# Conveniently Pre-Tagged and Pre-Packaged: Extended Molecular Identification and Metagenomics Using Complete Metazoan Mitochondrial Genomes

**DOI:** 10.1371/journal.pone.0051263

**Published:** 2012-12-14

**Authors:** Agnes Dettai, Cyril Gallut, Sophie Brouillet, Joel Pothier, Guillaume Lecointre, Régis Debruyne

**Affiliations:** 1 Muséum national d'Histoire naturelle, Département Systématique et Évolution, UMR 7138 Systématique, Adaptation, Évolution UPMC-CNRS-MNHN-IRD-ENS, Paris, France; 2 Université Pierre et Marie Curie, UMR 7138 Systématique, Adaptation, Évolution, UPMC-CNRS-MNHN-IRD-ENS, Paris, France; 3 Université Pierre et Marie Curie, UFR 927, Atelier de BioInformatique, Paris, France; 4 Muséum national d'Histoire naturelle, Département Systématique et Évolution, UMS 2700 CNRS, MNHN, Paris, France; Biodiversity Insitute of Ontario - University of Guelph, Canada

## Abstract

**Background:**

Researchers sorely need markers and approaches for biodiversity exploration (both specimen linked and metagenomics) using the full potential of next generation sequencing technologies (NGST). Currently, most studies rely on expensive multiple tagging, PCR primer universality and/or the use of few markers, sometimes with insufficient variability.

**Methodology/Principal Findings:**

We propose a novel approach for the isolation and sequencing of a universal, useful and popular marker across distant, non-model metazoans: the complete mitochondrial genome. It relies on the properties of metazoan mitogenomes for enrichment, on careful choice of the organisms to multiplex, as well as on the wide collection of accumulated mitochondrial reference datasets for post-sequencing sorting and identification instead of individual tagging. Multiple divergent organisms can be sequenced simultaneously, and their complete mitogenome obtained at a very low cost. We provide *in silico* testing of dataset assembly for a selected set of example datasets.

**Conclusions/Significance:**

This approach generates large mitogenome datasets. These sequences are useful for phylogenetics, molecular identification and molecular ecology studies, and are compatible with all existing projects or available datasets based on mitochondrial sequences, such as the Barcode of Life project. Our method can yield sequences both from identified samples and metagenomic samples. The use of the same datasets for both kinds of studies makes for a powerful approach, especially since the datasets have a high variability even at species level, and would be a useful complement to the less variable 18S rDNA currently prevailing in metagenomic studies.

## Introduction

For the last twenty years mitochondrial sequences, used on their own or along nuclear sequences, have been the workhorse of molecular systematics and underpinned thousands of publications. Now the mitochondrial genome might be one of the keys to biodiversity studies' transition from the slow and expensive Sanger sequencing to next generation sequencing technologies (NGST). The low cost and very high throughput of NGST has had an immense impact on the ease and scope of whole genome sequencing projects ([1], [2]), for conservation genetics [3] as well as for molecular ecology ([4], [5], [6]). Yet molecular identification and phylogeny have largely missed out on this progress. NGST can generate a very high number of sequence reads for complex samples. But molecular systematics studies require the acquisition of orthologous markers for a large number of specimens, as well as a way to assign the sequences to their original specimens. While the sequencing of metazoan complete nuclear genomes is becoming straightforward, it is not yet possible in the large numbers of specimens necessary for biodiversity studies. Moreover, while the cost of sequencing has gone down steadily, the assembly and comparison of genomes remains a costly endeavour in both computation and manpower ([7], [8]) because of their size and complexity. The complex genomes of the samples in species and samples-rich datasets should therefore be restricted to one or a few orthologous markers to be tractable. This is generally done by PCR or capture and has yielded metagenomic studies of very high interest ([4], [5], [6]). However, a number of problems remain. Technical issues, like PCR primer universality or reference dataset completeness are a limiting factor ([9], [6]). Moreover, the sequenced specimen needs to be linked to the sequence by tagging the PCR or original library, or by physically dividing the sequencing run. Both approaches are work intensive and more expensive than undivided, untagged runs, and often limited in the number of specimens that can be run in parallel.

We propose here an approach for biodiversity studies based on the intrinsic properties of the metazoan mitochondrial DNA and long established molecular techniques for marker isolation, followed by multiplex sequencing of divergent organisms. Its design permits to link the sequences to the specimen they were obtained from without additional experimental steps, while allowing multiplexing to fully exploit the power of the NGST.

There are several desirable properties for a marker used in large scale sequencing in metazoan systematics.

- The marker has to be easily enrichable or amplifiable.
- The marker must be variable enough to yield information for closely related species, even taking into account the non-negligible sequencing error rates encountered with some NGSTs.
- The marker needs to be comparable across taxa with a reasonable shot at orthology, and if possible ease of alignment. To this needs to be added the technical aspect of finding a reliable way to recover the link between the specimen and the sequence.
- The marker needs to be long enough to add some interest above the results achievable by Sanger sequencing.
- Using markers with known properties and already existing datasets provides access to current knowledge on their molecular evolution, and a possibility to integrate the new datasets with previous projects. By developing an approach that can be used for both metagenomics and molecular systematics studies, we can refine the protocols and conduct powerful joint analyses of the datasets.
- Markers with a large available reference library are useful for identification in metagenomic studies without having to generate a new library.

**Figure 1:**
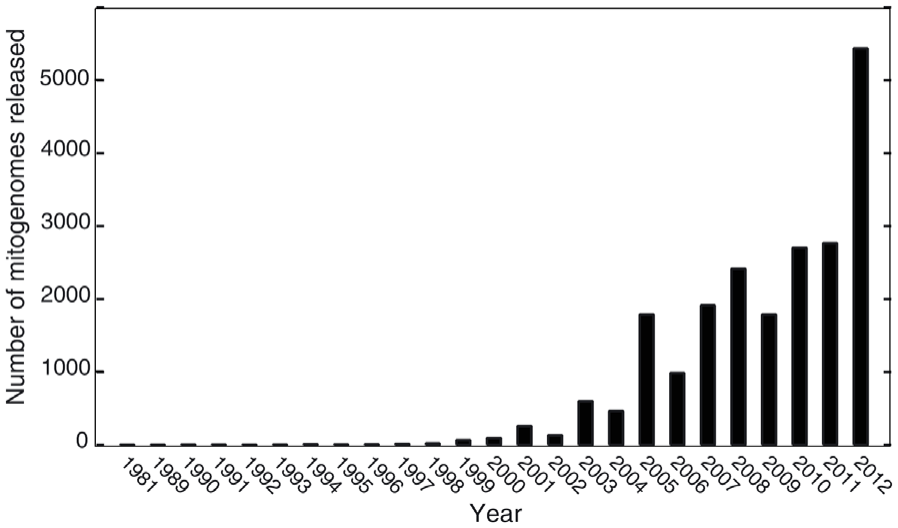
Release of mitogenome sequences in GenBank. The number of released mitogenomes has been plotted against the year of release (2012 covers only the first 9 month of the year, until the date of analysis).

The complete mitochondrial genome of metazoans fulfils all these criteria. Its small and relatively conserved size makes it isolable based solely on its physical properties, without having to resort to knowledge about its sequence. In Metazoans, it is on average ten times more variable than the nuclear genome ([10], [11]). However, the substitution rates of mitochondrial genes vary relative to each other across lineages ([12], [13], [14]), providing an array of markers with different divergence levels that can be used at different phylogenetic scales ([15], [16], [17]). The marker composition of the mitogenome varies little in Metazoa, and at least the coding ones are easy to align even between distantly-related species. The mode of inheritance in most metazoans is relatively straightforward, with only low rates of recombination despite a high copy number per cell [18]. As for the availability of reference datasets, hundreds of thousands of identified sequences for several mitochondrial markers populate EMBL, GenBank, and the Barcode of Life database [19]. Lately, complete mitochondrial genomes have become increasingly popular, and their rate of accumulation in GenBank has increased dramatically in 2012 (fig. 1). Yet for all its qualities, a practical approach to exploit it for a wide array of taxa using NGST is missing. Mitogenomes are still mostly being sequenced using Sanger technology and a very large number of primers ([20], [21]), or NGST using tags [22] or one sequencing run per mitogenome [23]. All these solutions are costly in time and reagents, limited to single specimens and make large datasets for distant species in phylogenetic and biodiversity studies complex to obtain. This has tremendous negative effects, as processing campaign samples takes months on end (leading to massive backlogs after specimen capture). We estimate that the method we propose could reduce the necessary time to sequence a sample by days, and divide the cost by up to a 100 times.

### Brief description of the approach (fig. 2.)

**Figure 2:**
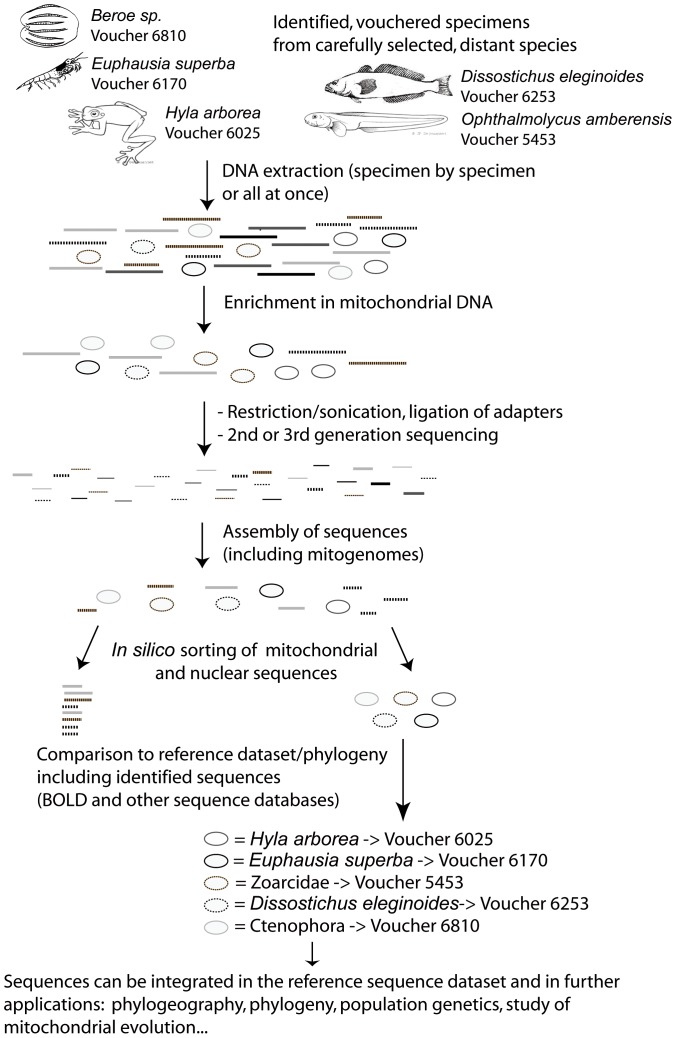
Summary of the approach for identified and individualized metazoan samples. Samples are selected so the mitogenomes can be separated at a later step.

Divergent identified specimens are selected for a single pooled sequencing run so that there is sufficient divergence all along their mitochondrial sequences to be able to separate them after sequencing. Their total genomic DNA is extracted, and enriched in mitochondrial DNA. The enriched extract is sequenced directly or amplified and sequenced. Individual mitochondrial genomes can then be assembled, as they are divergent enough to be distinct along their whole length. The assembled mitogenomes are linked to their specimen of origin by comparing their COI and/or other mitochondrial marker with the identified sequences present in a reference database, effectively using the sequences themselves as tags for sorting.

We describe here the approach, the type of sequence data it generates, the procedure to recover mitochondrial genomes without external tagging, and some potential uses. We perform an in-silico validation test based on the analysis of a simulated dataset with read lengths of two different sizes to represent average read length of three 2^nd^ generation desktop sequencing platforms, Illumina Mi-Seq, 454 GS junior and Ion Torrent PGM. Thus we can contrast their relative efficiencies for the experimental protocol proposed here.

## Materials and Methods

### Sliding window analyses

These analyses aim to evaluate the variability of sequence divergence along mitogenomes. In this way we can ascertain whether it would be possible from partial sequences to predict useable dissimilarity, i.e. the absence of identical stretches of sequences across species. Metazoan complete mitochondrial genomes were downloaded from GenBank and separated according to family (family and genus were used to fast-select sequences more related to each other than they are to the other available sequences). Mitogenome sequences were prepared so that the start of the Cytochrome oxidase I sequence was the first position. For families where several sequences were available for the same genus, alignments were performed using ClustalW [24]. Analyses were limited to these closely related species to limit mutational saturation and bias in the comparisons, as well as for ease of alignment. Very divergent sequences posing alignment problems were not considered in the analyses, such divergent taxa being deemed irrelevant to test the power of the approach. Indeed, the goal here is to identify the lower limits of divergence beyond which observed differences are sufficient all along the mitogenome length to allow for their unambiguous post-sequencing demultiplexing via straight assembly. P-distances were calculated on the Folmer fragment of the COI (position 50–700 of the COI sequence), to evaluate whether this value can be used as a proxy to determine if the specimens can then be combined. Sliding window analyses were performed for 150 bp and 450 bp windows, with a step of 15 and 45 base pairs respectively, in order to test the importance of sequence length in NGST acquisition of distinguishable mitochondrial sequences. These sequence lengths are compatible with all available desktop platforms: the 454 GS Junior (450 bp) and the Ion Torrent PGM and Illumina Mi-Seq (150 bp). Platform choice for the comparison was based on sequencing run size (moderate run size for easier multiplexing and sample separation), and wider accessibility to non-model species projects) and generated sequence length (comparison of longer and shorter sequences).

### Simulated datasets and assembly

In order to assess how mitogenomes could be assembled in real experiments, we ran simulations on datasets assembled from available complete sequences. As a general procedure, reads were generated from mitogenomes, pooled, and the pooled reads were assembled.

A 100 sequence dataset was assembled on the basis of the p-distance values from the previous analysis (File S1). The dataset was selected from a single taxonomic group (Actinopterygian fish) to replicate a realistic sequencing for a phylogenetic or systematics study in a specialised laboratory. It contains only species from different families with all COI p-distances above 15%. Simulations of reads and assemblies of pooled reads were carried out on an Intel Macintosh QuadCore with 14 Go RAM. Simulations of ‘Titanium’ 454 reads from mitogenomes were obtained using 454 sim [25]. Ion Torrent and Illumina reads were simulated using dwgsim, which is based upon wgsim from SAMtools package [26]. Both single- and paired-end reads were generated. Ion Torrent and Illumina read simulations were generated with errors rate of 0.02 per base, mutation rate of 0.001 (1/10 of mutations are indels and probability that an indel is extended is 0.3) and probability that a random DNA read occurs is 0.05. Two mean length of reads were simulated, 150 and 300 bp (only 150 pb for paired-end), to evaluate the importance of sequence length within the same type of data. 454 reads were simulated using the default parameters of 454 sim, with Titanium error profiles. Reads were then assembled with MIRA 3.9.1 [27] with 454, Illumina or Ion Torrent parameters as needed. Assembly results were visualised and compared to the original dataset using Mauve [28] with non-progressive alignment.

Two different types of read datasets were assembled.

First, exact reads were generated from genomes using a simple home-made script, taking into account the circularity of the genomes but with uniform coverages. These reads included a variable number of sequences from the dataset (10, 20, 30, 50 and 100, list given in supplementary material).

Second, ignoring genome circularity, 454, Illumina and Ion Torrent read simulations were carried out on 10 to 100 fish mitochondrial genomes, with uniform or various coverages over all mitochondrial genomes. Uniform coverages always provide better results than variable coverage at the assembly stage, but it is unlikely that equimolarity of all mixed mitogenome DNAs will be reached, so variable coverage should be more realistic. Mean coverage from 10× to 68× (or more) by step of 2× was simulated for Ion Torrent and Illumina reads for 30 fish mitogenomes (i.e. the coverage the first mitogenome is 10× while the coverage of the thirtieth is 68×). Two different mean read length (150 and 300 bp) have been tested for single paired reads (only 150 bp for paired end reads. Before the assembly step with MIRA, all simulated reads from the 30 mitogenomes were pooled.

### Comparison of 18S and mitochondrial markers variability

For five groups, datasets with both 18S and mitochondrial sequences available for the same specimens were identified, either through the Barcode of Life Data system (BOLD) project listing (all projects including several 18S rDNA sequences: [29], [30], [31]) or through publications databases ([32], [33]). Pairwise distances for species from the same genus or between genera in the same family were estimated either using the BOLD distance summary tool or the results of the publications. When this was not possible or available, we aligned the sequences using clustalW and calculated the distances using Mega4 ([34]). As with the sliding window analyses, only relatively closely related species (within genus and within family) were compared to try to avoid mutational saturation and bias in the divergence comparisons across markers.

## Results and Discussion

### Sample pooling selection

Our approach is based on local dissimilarity for post-sequencing de-multiplexing of the sequence pool, and therefore requires a careful selection of specimens before multiplexing runs. The goal is to pool samples possessing sufficient divergence all along their mitochondrial genomes, so that every individual sequence can be singled out at the sequence assembly stage, and then identified using the reference data as one of the samples included in the run. The selection is tied to the sequencing platform used, as both error rates and sequence length are important for the correct assembly.

**Figure 3:**
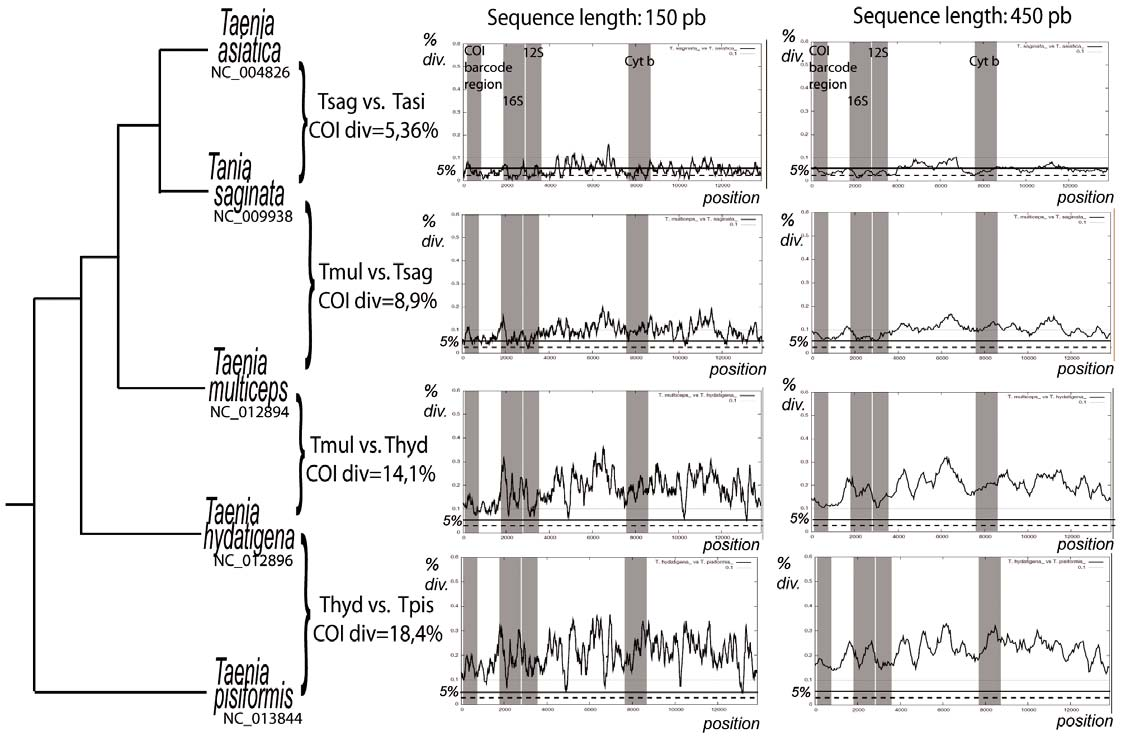
Sliding window analysis for checking mitogenome divergence before multiplexing. COI (or other mitochondrial marker) divergence can serve as a proxy to estimate sequence divergence over the whole mitogenome for fragments of the same length as generated by the NGST used. In the absence of sequence data, taxonomy can serve the same purpose, albeit less efficiently. There is much more variation between small fragment divergences than large fragment divergences, so while only distant species might be multiplexed when using short fragments generating sequencers, less distant species might be multiplexed if the sequences are longer. Some species are too similar to be multiplexed at all, except if additional tagging is used. Phylogeny follows Knapp et al. 2011.

Variability in the levels of divergence along mitogenomes can be visualized using the sliding window analyses for a given pair of sequences. Examples for several different sequence divergence and two window sequence lengths are presented in fig. 3 for genus *Taenia.* Results for 113 families from 10 phyla across metazoan diversity are available in the additional material (Files S2, S3, S4). In general, sequence divergence is in the same range of magnitude for all windows for the longer window length (450 bp) no matter where the window is located along the genome. Not surprisingly, for the shorter windows (150 bp), there is much more variability between the observed divergences. As the mitochondrial genome includes alternating variable and more conserved regions, some sequence stretches might be identical across co-sequenced taxa if the multiplexed taxa are not divergent enough. However, longer fragments “bridge over” the conserved regions and also include parts of more divergent regions. Most mitogenomes with a COI 5′ region divergence above 15% do not have a single 450 pb window with a divergence below 5%. The same is true for the 150 pb window length for a slightly higher COI divergence. Some species pairs did not follow this pattern, and there were areas with 0% divergence on the sliding window figures. However, checking the alignments for the analyses presenting regions with very low divergence for otherwise highly divergent species pairs revealed that in these regions of the alignment only one of the two sequences was present due to a deletion in the second. These are therefore an artefact of the sliding window visualization rather than truly identical areas which could be troublesome at the demultiplexing stage. The analyses for Chimaeridae or Protopteridae are good examples of such problems. There are a few exceptions, like a 200 base pair region with no divergence in the 16S rDNA of the two compared species of *Balanoglossus* (Hemichordata). The results of these analyses thus provide a rough guideline for the minimal divergence of sequences that can be safely multiplexed. Sequences that do not diverge much from each other (for instance in fig. 3, *T. saginata* vs. *T. asiatica*) include many stretches with no difference at all. These should not be combined in a single sequencing run with the current sequence length and error rates, but with new sequencing techniques with low error rates and very long sequences, it might become possible to sort them.

The relative variability of mitochondrial markers also varies across taxa. For most taxonomic groups, the two rDNAs represent the least variable segment of the mitogenome. COI generally is neither the least nor the most variable (fig. 3), although in a few groups (several gastropod families like Planorbidae, Conidae and Vermetidae, as well as Tetranychidae and Schistosomatidae), it is among the most conserved regions of the mitogenome. As the divergence level across mitochondrial genes is variable, but does not differ by orders of magnitude ([13], [14], present study), approximate divergence of any part of the genome can be roughly estimated from the largely available mitochondrial markers (such as ribosomal markers or cytochrome oxidase 1), using the divergence of the shorter mitochondrial sequences as a proxy to assess the overall divergence. Previous studies had shown that it is also possible to get a preview of mitogenome composition using COI [35], so biased compositions that might represent a problem for some sequencing approaches might also be detected in advance. If the data is available, however, a sliding window analysis along the whole mitogenome of the taxa of interest or of closely related groups will give more precise results and pinpoint areas of lesser divergence that might pose problem at the assembly stage. The recent wealth of partial mitochondrial sequences and complete mitogenomes (fig. 1) provides sufficient comparative data to make an optimal selection for many taxonomic groups even for relatively closely related species (table 1) without having to acquire new sequences. For groups where no sequence data is available, multiplexing with species from very distant taxonomic groups to acquire preliminary data is probably the best approach.

**Table 1:** Size and number of mitogenome sequences available, in relationship with number of described species and COI barcodes available.

Taxonomic group	Average length	Shortest	Largest	Mt genome nb in GenBank	Nb of COI sequences in BOLD	BOLD species with barcodes	Described species nb [36]
**Acanthocephala**	13888	13888	13888	1	163	13	1150
**Annelida**	15565	14426	17217	9	17000	1912	16763
**Arthropoda**							
Hexapoda	15720	**8118** *	19517	269	1060682	115353	>1 000 000
Arachnida	14855	13075	**24961**	47	42798	5010	102248
Pycnogonida and Merostomata	15083	14681	15474	6	1645	168	1340
Crustacea	15767	14113	18372	63	41406	4676	47000
Myriapoda	14976	14637	15695	8	1274	198	16072
**Brachiopoda**	14586	14017	15451	3	99	44	350
**Bryozoa**	14092	13026	15433	4	406	108	5700
**Chaetognatha**	11708	11121	12631	5	123	21	121
**Cephalochordata**	15065	14975	15102	8	251	10	33
**Chordata**							
Tunicata	14945	13648	16351	12	934	138	2760
Petromyzontiformes and Myxiniformes	16980	16159	18909	5	486	59	88
Chondrichthyes	18055	16697	**24889**	17	14012	964	1160
Actinopterygii	16650	15564	**22217**	864	99819	11204	30841
Coelacanthiformes and Dipnoi	16391	15873	16646	6	30	8	11
Amphibia	17492	15897	**22874**	111	12623	1291	6515
Testudines	17120	16439	19438	40	556	233	313
Archosauria	17106	14935	**22737**	171	28809	4221	10013
Lepidosauria	17442	15181	**25972**	106	4168	870	8396
Mammalia	16587	15289	17734	342	48827	1990	5487
**Cnidaria**							9795
Anthozoa	18118	14853	**21376**	39	1314	429	
Hydrozoa	10731	7686	16314	3	319	110	
Scyphozoa	16937			1	448	49	
**Ctenophora**	10326			1	3	1	166
**Echinodermata**	16211	14837	17538	29	17897	1391	7003
**Echiura**	15437	15113	15761	2	9	4	176
**Entoprocta**	15093	14862	15323	2	3	3	170
**Hemichordata**	16067	15708	17037	4	9	4	108
**Mollusca**							≈85000
Aplacophora	**21008**			1	7	6	
Bivalvia	18103	15712	**32115**	43	8526	966	
Cephalopoda	17631	15617	**20331**	14	2864	380	
Gastropoda	15268	13670	**26835**	42	31742	5116	
Polyplacophora	15532			1	1788	114	
Scaphopoda	14212	13932	14492	2	98	21	
**Nematoda**	14972	12626	**26194**	53	2231	329	<25000
**Nemertea**	15511	14558	16296	4	513	90	1200
**Onychophora**	14481	13673	15347	4	210	52	165
**Placozoa**	**37262**	**32661**	**43079**	5	2	1	1
**Platyhelminthes**	14130	13387	16901	37	4134	340	20000
**Porifera**	19977	16414	**28958**	42	615	180	≈6000
**Priapulida**	14919			1	9	1	16
**Rotifera**	13048	11153	15319	3	2425	248	2180
**Sipuncula**	15498	15494	15502	2	114	47	144
**Tardigrada**	15108	15047	15169	2	297	9	1045
**Xenoturbellida**	15234			1	2	1	2

Broken up by Metazoan group, number of available complete mitochondrial genomes (GenBank, dec 2011) with average, smallest and largest length, sequences and sequenced species available for COI in BOLD, and number of described species for the group (“World described accepted” from [36]). Genomes below 10 kb or above 20 kb are in **bold**.

* Anaticola crassicornis (Phthiraptera) is the only species available with a genome below 12 900 bp.

The selection for multiplexing also relies on the reference database coverage of a group (table 1), so that the sequences can easily be identified *a posteriori* by sequence comparison with available reference datasets. Molecular identification ease and quality relies on the representation in a reference dataset ([9], [6]). Taxa with good reference coverage will pose little problem, while others with no dense references might need to be sequenced separately from others from their taxonomic group until more data is available (fig. 2, [36]) and they can be fished-out easily from the sequence pool. Alternatively, a few sequences for partial COI or ribosomal markers could be sequenced trough Sanger technique for species with no reference in databases to serve as reference for demultiplexing.

### Number of samples per run

The number of samples that can be combined in a single run depends on the characteristics of the sequencer (necessary coverage depth, sequence length, error rates), as well as on the efficiency of the enrichment process (proportion of mitochondrial sequences vs by-product nuclear sequences). Table 2 presents a rough calculation of the number of genomes that can theoretically be sequenced per run for several currently available sequencers, for different coverage depths, using minimal throughput values from [37] and [38]. The recommended minimal sequence coverage (average number of times a base is read) for good quality sequences depends on the sequencer used. Prices are estimated for coverage values recommended for these sequencers. The real throughput is considerably higher than this (2–3 times, [37]), and progress is extremely fast, so the sequence length and prices are respectively under and overestimates for future studies. Even with an efficiency and a number of mitogenomes per sequencing one order of magnitude lower than what we calculated here, the cost of a complete mitogenome would be considerably lower than the current PCR and Sanger-sequencing based approach. Even sequencers generating a relatively small amount of data (454 Junior, Ion Torrent PGM) mean a considerable economy, and permit multiplexing of a large, yet tractable number of specimens in parallel. Sequencers with very high-throughput (such as Illumina HiSeq), while producing considerably cheaper sequences theoretically, also require the multiplexing of a very high number of specimens. In these cases, tagging smaller pools of multiplexed mitogenomes would involve tractable numbers of samples without having to tag thousands of samples separately.

**Table 2:** Complete mitogenome output for two next generation sequencers, with rough estimate of the cost.

			Nb of mitochondrial genomes in a single run			Approximate cost in $ [37], [38]		
		Minimum throughput	20×	50×	100×	per sequencing	per complete mitochondrial sequence	
454 GS Junior		35000000	51	21	10	1100	21,37	in 20×
Ion Torrent PGM	314 chip	10000000	15	6	3	225	38,25	in 50×
Ion Torrent PGM	316 chip	100000000	147	59	29	425	7,23	in 50×
Ion Torrent PGM	318 chip	1000000000	1471	588	294	625	1,06	in 50×
Illumina MiSeq		1500000000	2206	882	441	750	0,85	in 50×
Illumina HiSeq2000	1 run	600000000000	882353	352941	176471	42000	0,24	in 100×
Illumina HiSeq2000	1/15 run	40000000000	58824	23529	11765	2800	0,24	in 100×
Sanger sequencing		800 bp seq, so nb of reaction/mitogenome >40–50				384	192	2 to 2,5×

Minimum throughput and cost per sequencing follows [37], [38]. The calculation of the number of complete mitochondrial genomes considers an average length of 17 000 bp per genome, and an enrichment process yielding a sample containing 50% of mitochondrial DNA and equimolarity of the samples.

However, as will be discussed later, with the current assembly options, lower numbers of combined genomes yield better results at the assembly stage.

### Enrichment

The mitochondrial genome, although it is present in multiple copies, only represents about one percent of the total DNA content of cells. While whole extractions have been used for mitogenome sequencing with no further treatment, the mitochondrial sequences recovered represent only a small proportion of the total sequences (around or below 0.5%, [23]). Without more efficient targeting for sequencing, obtaining mitogenomes through high throughput sequencing is not cost nor effort efficient. Most current enrichment methods rely on complementary hybridization of the targeted DNA sequences with single-stranded probes to capture target sequences [39] [22]. While this might be appropriate for known sequences with at least some highly conserved fragments, it is far too sequence-specific to target highly divergent mitochondrial genomes, especially from little known or very divergent groups. However, processes for enrichment or specific isolation of mitochondrial DNA versus nuclear DNA have been mastered for decades, using the physical properties of the mitochondrial genomes, especially size and composition. When fresh tissues are available, there are commercial kits targeted at the extraction of mitochondria, and crude mitochondrial pellets can also be obtained on sucrose gradient [40] as a preliminary step to DNA extraction.

Samples highly enriched in mitochondrial DNA mean more usable sequences per run. However complete purity is not a requirement here because the high number of sequences generated per run allows for loss, and so complex methods yielding high purity [41] are not a requirement. Simple methods based on size can already provide a considerable enrichment. Plasmids of a size similar to that of the mitochondrial genome are isolated every day in many labs around the world, by simple size sorting through migration of total DNA on standard 0.75 or 0.5% agarose gels in TAE, followed by purification using electroelution or gel extraction kits available from most of the major biotech manufacturers and often based on ancient techniques ([42],[43]). For size based selection methods, having some information on the mitogenome size for the specific group of interest allows to better target the purification. While most mitogenomes vary relatively little in size, for a few taxonomic groups they are well above 20 kb or below 12 kb (table 1). Direct current electrophoresis on agarose gels is already used as a step in purification of soil DNA for metagenomics in some protocols [44].

As the sorting is performed on the sequences, combining samples from the extraction, and performing batch mitochondrial enrichments would reduce both the manpower and reagents necessary per sample. While obtaining approximate equimolarity is no trivial problem, sample preparation and measure with a spectrophotometer before pooling yielded good sequencing results in pooled and tagged human complete mitochondrial genomes [22].

### Sequencing

There are good reviews of the advantages and inconvenients of the sequencers themselves (for instance ([37], [38]). However they are outdated almost faster than they can be published due to the fast developments in the sequencers (see [45]). Both hardware and reagents are updated constantly, and it is difficult or impossible to make recommendations for the design of future studies based on even recent results. Table 2 is yet an attempt to provide estimates for the number of samples that can be multiplexed and the assorted cost using current running costs and protocols. While most NGST can be used, the length of the generated sequences is key to the choice of relevant taxa to multiplex (fig. 3). Very short sequences have a higher chance of being located entirely within a conserved region, so the choice of the platform might reveal crucial based on the scope of the project to achieve. Combination of extremely divergent samples is the most forgiving technique, while more caution must be exercised for less divergent samples. Error characteristics of the sequencers can also play a role, although both comparison with reference sequences and coding sequence control can help.

### Assembly

Assembly can be based either on the existing complete mitogenome datasets [27], or using de novo assemblers ([27], [46], [47]), depending on the type of sequence output. The risk of recovering chimeric sequences combining several mitogenomes is low, first because of the preliminary choice of the specimens, then because considerable work has been done on allele separation and identification for diploid genomes [7], and settings can be fine tuned to get the best results.

For the analyses based on the exact read assemblies that were built taking into account circularity of the genomes, the fact that the assembly program does not account for the circularity posed problems. These genomes were very often assembled in two contigs, one corresponding to the last part of the reference genome, while the second covered the first part (data not shown).

**Figure 4:**
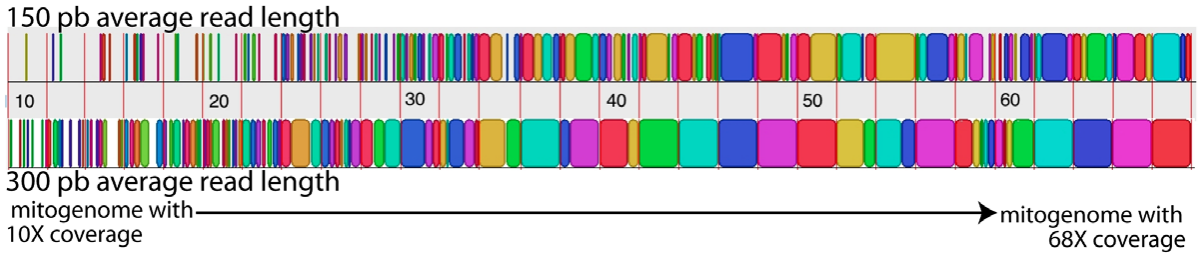
Comparison of the 30 original mitogenomes with the contigs assembled from the Ion Torrent simulations. Upper part represents contigs resulting from reads with 150 pb mean length, lower part contigs assembled from reads of 300 bp mean length. Orange lines indicate the limits of the mitogenomes; these are ordered from the one with least coverage (10×, on the left) to the most covered mitogenome (58×, on the right). Coverage of the mitogenomes are by steps of an increment of 2× (scale indicates the coverage). The same dataset was used to generate the 150 and the 300 bp reads. Contig colors have no meaning beyond ease of visualization. Generated using outputs of the genome alignement program MAUVE [28].

**Figure 5:**
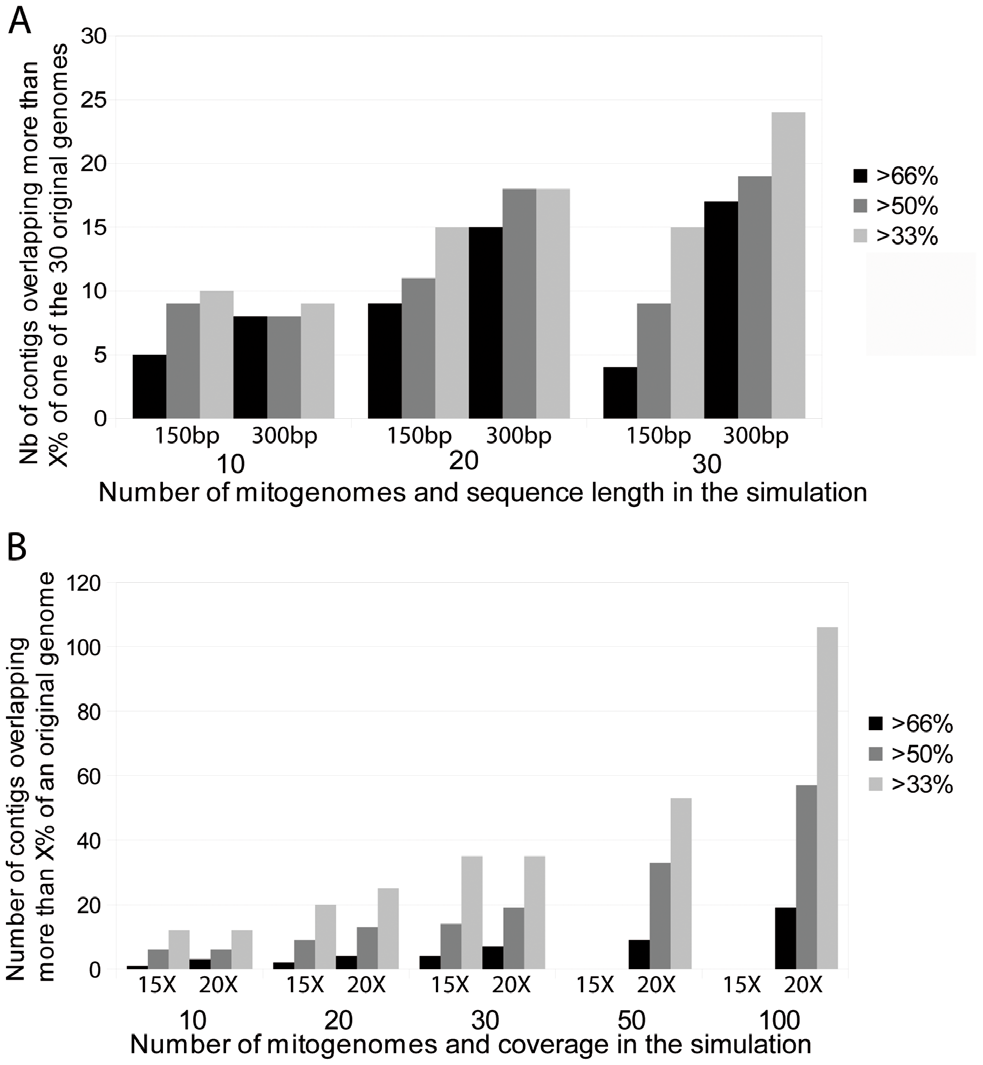
Number of contigs overlapping >X% against the number of mitogenomes. a. for two different sequence length (Ion Torrent simulations) e.g. in the simulation with a mean read length of 300 bp, there are 19 contigs overlapping more than 50% of 19 of the 30 mitogenomes. Two contigs overlapping a mitogenome by about 33% can belong to the same mitogenome, while there is only one contig that can cover 50% or 66% of a mitogenome. Overlap % calculated against an average mitogenome size (16 400 bp). b. for two different coverages (454 simulations) e.g. For 15× coverage, there are 9 contigs overlapping more than 50% of 9 of the 20 mitogenomes). Overlap % calculated against an average mitogenome size (16400 bp). The mean coverage of 15× correspond to coverages varying between 10 and 21 for the simulated individual mitogenome, and those of 20× correspond to coverages between 19 and 21. Note: in the simulation for 50 and 100 mitogenomes, the 15× coverage is not sufficient for MIRA to achieve the computation.

For the variable coverage analyses, fig. 4 displays the correspondence of the original mitogenomes with the contigs resulting from the assembly with MIRA. Not surprisingly, assembly for genomes with lower coverage provides a few short contigs, while higher coverage yields fewer long contigs recovering a large part of the mitogenomes. Sequence length also plays a role: when the mean length of reads is 150 bp, a coverage of at least 40× is necessary to obtain large contigs overlapping a noticeable fraction of the mitochondrial genome. When the mean read length is 300 bp, coverages above 30× might be sufficient (fig. 4). For simulations including only 10 mitogenomes, even at 150 bp read length, 30× coverage is sufficient to recover large contigs (fig. 5a). Figure 5a gives the fractions of contigs overlapping one of the original mitogenomes by more than 66, 50, or 33%, for mean read lengths of 150 bp and 300 bp (simulations with the same sequencer, Ion Torrent PGM) including 10, 20 or 30 mitogenomes (variable coverage from 10× to 68×). For 300 bp long reads and 30 mitogenomes, a contig of more than 50% of the total mitogenome length is retrieved for 19 of the 30 specimens. For 17 of these, the contig actually covers more than 2/3 s of the length of the genome. When only 20 mitogenomes are included, contigs overlapping more than 50% of the sequence length are recovered for 18 of the 20 specimens. Even with 150 bp mean length reads, one third of the mitogenomes are overlapped by contigs covering more than 50% of the genome. As coverage varies from 10× to 68×, this corresponds mostly to the third of the genomes that have a coverage above 50×. These coverage values are coherent with the coverage cited for other types of genome sequencing. The simulations thus suggest that, with good coverage, it is possible to recover large contigs for most of the sequenced mitogenomes, even without strict equimolarity of the samples.

Small increases in coverage actually have a small positive effect in assembly. Figure 5b gives the results for the 454 simulations for 10, 20, 30, 50 and 100 mitogenomes, with mean coverages of 15× (from a minimum of 10× to a maximum of 21×) and 20× (from a minimum of 19× to a maximum of 21×) and a mean read length of 400 bp. At 20× coverage, all simulations give contigs overlapping more than half of the total length of the mitogenome sequence for about 2/3 of the sequences.

**Figure 6:**
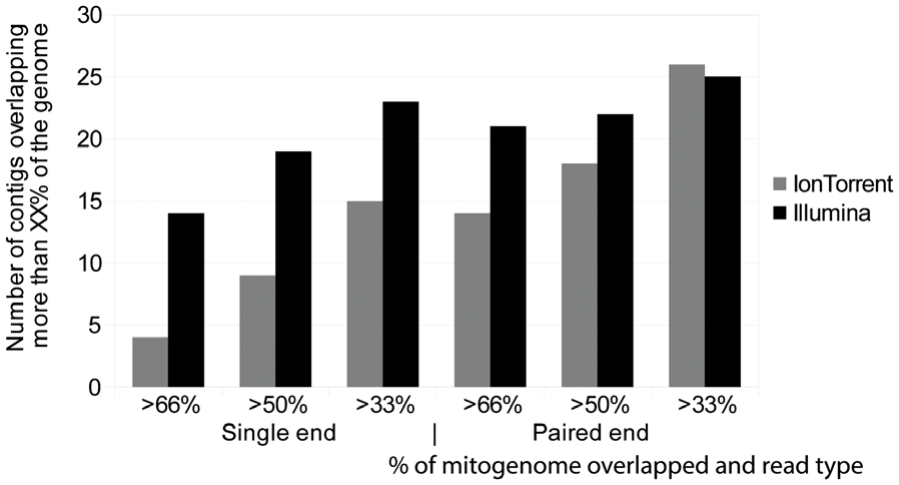
Comparison of paired-end and single sequences for two platforms (Illumina MiSeq vs. Ion Torrent PGM) and 30 mitogenomes. Number of contigs overlapping more than X% against number of mitogenomes. Overlap % calculated against an average mitogenome size (16400 bp).

As expected, using paired end instead of single end (with the same coverage) improves the assembly results considerably for both sequencing platforms simulated (Fig. 6). Due to apparent sequencing platform effect however, the comparison of paired-end reads is less straightforward. It is however obvious though that the construction of paired-end sequences should be favoured whenever possible to further facilitate the demultiplexing and assembly.

Building contigs overlapping large parts, or even whole mitogenomes, appears to be a reachable goal when coverage is sufficient. These coverage values are in agreement with the coverage cited for other types of genome sequencing. Multiple contigs might then be assigned to a single mitogenome using similarity searches on multiple mitochondrial markers. Additional techniques to enhance the discrimination of the read at the demultiplexing stage might bring some ameliorations. We tested one, the construction of a self-organizing map of tetranucleotide frequency [48]. It revealed very little structuring in the datasets analyzed in this study (data not shown).

While these simulations, intended to demonstrate a practical use in a specialised lab, used a dataset of actinopterygian teleost genomes, we are confident that similar results would be obtained using sequences from other groups, as the assembly depends chiefly on sequence divergence (explored in the sliding window analyses for a large diversity of phylums) and sequence length, which is imposed by the choice of sequencer rather than of the taxa.

### Annotation and quality control

Annotation of mitochondrial genomes is performed routinely in laboratories all over the world for all studies regarding mitochondrial genome evolution and phylogenetics. It can be performed using dedicated tools (for instance MOSAS, [49]) and/or using comparisons to already available related mitogenomes, mitochondrial gene sequences (for instance using alignment against profile HMMs [50]) or mRNA and tRNA structure ([21], [51]). Sequence alignment and comparison with closely related species is possible for many groups due to the amount of already known mitogenomes. This provides a good opportunity of quality control for the assembled sequences, including chimaeric sequences and assemblies. Additionally, a large part of the mitogenome sequence is composed of coding genes. These can be checked for reading frame shifts, stop codons, and length variations. This is especially important as errors are consistently associated to homopolymer misreads for some sequencers ([37]), creating artefactual indels in the raw sequences.

### Sorting and attribution to the samples

Biodiversity studies need to maintain the link between the sequences and the species they were obtained from, in order to discuss the results in relationship with hundreds of years of accumulated biological knowledge and not just based on sequence diversity within the dataset [52]. Linking the sequences with a precise specimen is advisable not only for the general quality of the data, but also so that the sequences can be used for intraspecific studies, as well as when cryptic species might be present [53]. There has been in recent years a wealth of studies on the criteria necessary for successful molecular identification [54] from which experience can be drawn. Here, if the choice of the multiplexed samples has been done carefully, we stand in an ideal configuration. All the samples to identify are distant from each other, the list is known *a priori*, and existence of sequences for identification has been checked beforehand. Molecular identification can take advantage of the vast effort in recent years resulting from the Barcode of Life initiative, with its unprecedented sequence reference library and quality standard [53]. The accumulated well-identified sequences can provide identification for many taxa, even if the coverage of some groups remains biased (see table 1). Tools for batch identification are available on both GenBank and BOLD.

Moreover, there are tens of thousands of species represented for not only COI, but also other mitochondrial markers, chiefly cytochrome b, 12S and 16S rRNA. These can be used to corroborate assignation if the mitochondrial genome has been assembled in a single contig. If the assembly was incomplete, identification of the contigs using these other markers located in different areas of the mitogenome can help piece it together.

More complex situations like the presence of mitochondrial heteroplasmy or doubly parental inheritance [55] can be taken into account. Mitochondrial heteroplasmy has been detected in quite a few metazoan groups ([56], [57]), sometimes with deep divergences between the two genomes. While representing an issue for mitochondrial sequence-based ID of specimens, it is less of a problem when it has been identified and if appropriate techniques are applied [57]. Due to its principle, our approach is ideal to pinpoint many more cases of heteroplasmy or unusual inheritance than currently known, as the different alleles are both accessed directly by the sequencing. However, when such cases are suspected, divergence of pooled samples must be adjusted so that both mitochondrial genomes differ enough from the other pooled samples to allow for good post-sequencing demultiplexing of the sequence pool.

### Advantages of the approach and of the wealth of new datasets

The analysis methods of the datasets can go from the use of partial sequence analysis for identification purposes (barcoding approaches) to fully fledged phylogenetic analyses on long sequence alignments, gene order coding studies and functional comparative analyses. The usefulness of the mitochondrial markers, and of the complete mitogenome for systematics has been recurrently demonstrated over the years ([13], [21], [58], [59], [60]). In the first nine month of 2012, there are already 42 publications available in PUBMED for “complete mitochondrial genome” and “phylogeny” (while there were only 38 for the whole of 2011).

The large number of mitochondrial datasets generated through the approach would of course vastly increase our knowledge about the evolution of this small genome, which is currently very limited for many groups (table 1). In recent years, complete mitochondrial genome sequences have become important tools for the study of genome architecture, phylogeny, and molecular evolution ([59], [61], [62], [20], [63]).

As will be further developed, the complete mitogenomes can themselves be used as reference datasets for metagenomic studies, as they can serve as reference for any mitochondrial fragment.

However, this is far from the only advantage of acquiring whole mitogenome data.

While single mitochondrial markers might not provide well-supported and reliable results for deeper relationships ([64], [65]), combined analysis of multiple mitochondrial markers has better results ([59], [60] [66], [20]). At a larger phylogenetic scale, complete or almost complete mitochondrial genomes provide decent quality phylogenetic signal for some taxa when analyzed with proper phylogenetic approaches ([13], [59], [60], [66]), although, as for any other marker, it also has downsides [65]. A more efficient method to obtain mitogenomes would eliminate the need of having to choose between a large number of terminals and long sequences, and very probably considerably improve results obtained for these datasets ([66], [20]).

At a smaller scale, each mitochondrial marker might in itself contain an insufficient amount of information for population genetics studies or for very closely related organisms. However, a few studies using the whole mitogenome have revealed that it contains a number of Single Nucleotide Polymorphisms [67], [68], [69] sufficient for some types of fine grained analyses of populations.

The approach itself presents multiple advantages for some currently problematic situations.

Amplification success issues plague all larger scale studies based on PCR approaches, especially when the sequences are very divergent. Non-binding or low binding efficiency of primers to some target DNAs because of sequence divergence is a serious problem for all mitochondrial markers, jeopardizing amplification and study of some samples and some groups and requiring multiple primers combinations ([70], [71], [20], [6]). The non-targeted approach proposed here avoids this step altogether and still allows for the recovery of orthologous sequence data.

Transfers of mitochondrial sequences to the nucleus are commonplace accross Metazoa, occurring recurrently and independently in many groups [72]. This causes multiple problems for studies relying on the amplification of fragments, as nuclear copies can be amplified instead of the mitochondrial ones, and impede in the identifications, biodiversity evaluations, or phylogenies [73]. However, the enrichment in mitogenomes prior to sequencing in our approach effectively gets rid of an important part of the nuclear genomes, and the odds of recovering the nuclear copies are lowered significantly.

### Limits of the new datasets

The datasets produced through this approach have all the assets brought by longer sequences and diverse substitution rates. However, they will not provide an universal answer to problems in systematics. Except for very rare exceptions, all the mitochondrial markers are physically linked and inherited as a single unit. They do not provide independent corroboration for the inference of the history of the species ([74], [66]), as the comparison of mitochondrial and nuclear markers would. Acquiring multiple mitogenomes is just a step on the road towards an integrative taxonomy, and these datasets need to be complemented by other sources of data [75].

There are also some metazoan groups with very low mitochondrial divergence. For some Porifera [76], Anthozoa [77], and Ctenophora, the rate of mitochondrial evolution compared to other Metazoans is lower by a factor 10–20, and a single marker can prove insufficient to discriminate between closely related species [78]. Moreover, in some of these taxa, the mitochondrial genome is heavily reorganized and/or contains introns. While using mitochondrial sequences might not be the best approach to resolve the fine phylogenetic relationships within these groups [79], it would still be useful to study deeper relationships [77]. However, multiplexing taxa with low divergence raises problem for the present approach. This can currently only be solved by adding tags for the sequencing, or combining with distant samples from other sequencing projects.

### Extension to environment DNA studies and complex marine samples

Another potential use for the method we outline here, is to improve classical metagenomic studies (i.e., without artificial assemblage of specimens). The complexity of species assemblages in zooplankton or interstitial fauna, as well as their sensitivity to changes in their environment makes them extremely reactive indicators, and so they are being increasingly used ([5], [80]). However, precise species identification for these samples is of utmost importance if we want a shot at describing accurately their diversity and distribution, and from there, correctly assess changes in community composition or biogeography. Yet the incredible diversity of marine groups, and the sometimes considerable difference between life stages of a same organism makes analysis of planktonic samples very complex [81]. The precision of identification within such samples can sometimes be only down to the family, the phylum or not even that, even with good conditions and specialists, and molecular identification can help [82]. There are multiple projects currently focusing on the molecular identification of zooplankton, within the Census of Marine Zooplankton and other projects ([81], [83], [84]) that have established extensive reference data, and determined on-board protocols to immediately treat the samples for best DNA quality. However, many studies to date have relied either on cloning (time consuming, expensive, and biased) or on the more easily amplified nuclear 18S and mitochondrial 16S rDNAs. But PCR-based methods also present problems. For all markers, there are more or less important problems of primer universality (lesser for 16S and 18S rDNA), restricted amplicon length, and availability of reference sequences in the databases ([82], [4]).

The most basic prerequisite for the use of a marker for identification is the existence of distinct sequences: two species with the exact same sequence cannot be distinguished with this approach. A number of examples have been published of species sharing sequences for a mitochondrial marker, although the problem is more widespread for 16S rDNA than for Cytochrome b or COI ([32], [16], [17]). 18S rDNA, while more useful for taxonomic assignment where no closely related sequences are available ([85], [86]) is more conserved than either 16S or COI in some taxonomic groups (table 3), and sometimes shows little or no divergence between some closely related species (table 3 and [82], [87]) distinguishable by mitochondrial markers (table 3 and [32]). When taking into account the error rates of the sequencing techniques, such low divergences can present serious pitfalls for differentiation of clades, as sequencing errors impose divergence thresholds to lower the risk of considering two clusters as distinct due solely to sequencing errors [88].

**Table 3:** Resolutive power of 18SrDNA versus mitochondrial markers.

					18S rDNA				mitochondrial			
Taxonomic group	Dataset reference	BOLD project name	nb of seqs	nb of species	seq length (bp)	nb of sp. sharing a sequence	pairwise intrageneric distances in the dataset	pairwise intergeneric distances in the dataset	Marker name: seq length (bp)	nb of sp. sharing a sequence	pairwise intrageneric distances in the dataset	Pairwise intergeneric distances in the dataset
Lepidoptera	[31]	LGC	70	68	655	20	0–0.65	0–1.43	COI: 655	0	4.28–12.68	4.59–15.44
Amphibia	[30]	CBAM	81	14	570	7	0	0–1.09	COI: 655	2	0–20.38	14.97–21.92
Ascidiacea	[29]	ASCAN	30	22	800	6	0–11.12	0–9.74	COI: 550	0	0.17–34.29	6.53–26.74
Nematoda	[33]		15	7	1708	3	0–1.07	0.06–1.13	12S: 499	0	0–12.37	7.5–25.9
Teleostei	[32]		160	71	1800	14	0–1.33	0–1.39	12S: 390	0	0–12.56	

Only specimens for which both markers were available were included in the comparison. The values are min-max of the pairwise divergences. Intergeneric distances are calculated on specimens belonging to the same family.

Additionally, at least in some taxa not all the 18S rDNA copies within an individual are identical ([89], [90]). The level of intra-individual divergence can be quite high among 18S rDNA sequences in some groups, in some cases actually higher than interspecific divergences ([89], [32]), so more refined approaches like phylogenetic placement methods [91] and secondary structure analyses [92] can be needed. Mitochondrial sequences present generally high levels of divergence, and studies have repeatedly shown that even single mitochondrial markers generally have distinct sequences by species, and sometimes even by population. This means that although there is still a risk of confusing two closely related species using our approach, this risk is much lower than using current protocols.

**Figure 7:**
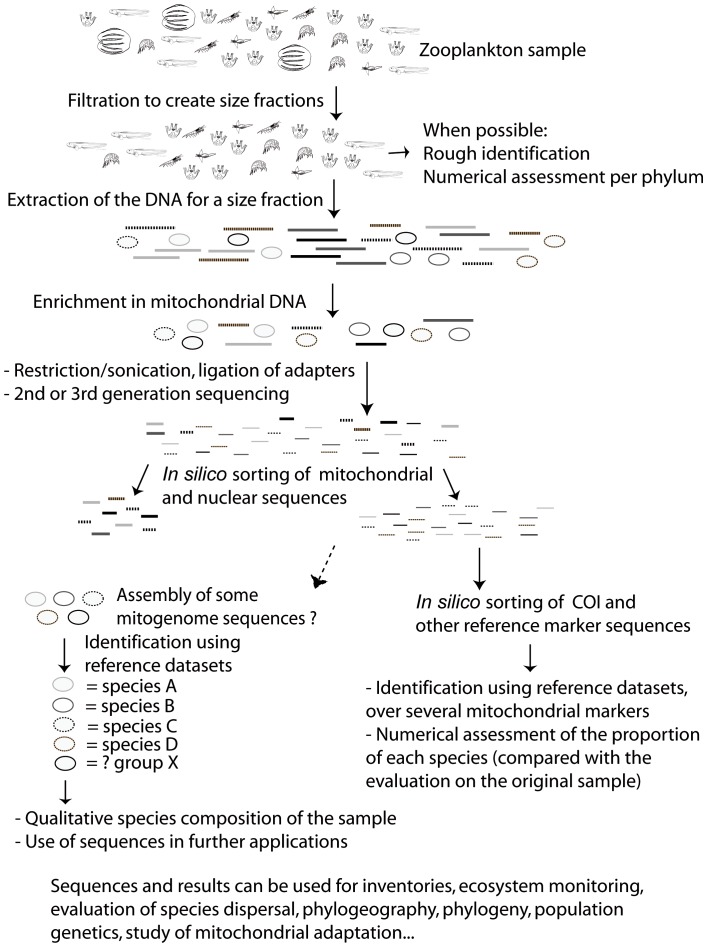
Summary of the approach for complex samples of unknown content (for instance zooplankton). For complex samples, the most valuable part of the approach is the isolation of mitogenomic DNA based on its properties, precluding PCR biases. This is followed by a cluster analysis on multiple mitochondrial markers. Complete mitogenome reconstruction might be possible only in some limited cases, as there might be a mix of closely related species in the sample.

Applying the technique we propose here to plankton samples, or other environment DNA samples, would open up a level of detail never reached before, using any or all mitochondrial markers for identification and further analyses, and providing the complete mitogenomes obtained in studies based on identified specimens as reference datasets (fig. 7). Both closed-reference and open-reference OTU clustering [5] can be used on mitochondrial datasets. These datasets can also be acquired in parallel with the 18S data, and combining and comparing the results of both will yield a better estimate of the diversity in the sample [85]. As, depending on the sequencer used, completely PCR-free sequencing techniques can be applied, this also has the potential to reduce the number of problems due to chimeric sequences, and open the way to quantitative datasets that are not biased by taxonomic group-dependent PCR efficiency.

## Conclusion

The approach we propose here has the potential to open or speed up very wide fields of research, harnessing new technologies to benefit biodiversity studies. A number of questions can now be explored. The best enrichment techniques in various settings need to be determined. With good coverage, it is possible to recover large contigs for a number of the sequenced mitogenomes, at least in the simulations, even without equimolarity of the samples. However, our simulations and assemblies show that existing programs for genome assembly lack some important features when applied to mitochondrial genomes. The circularity of mitochondrial genomes is not yet taken into account, which could lead to reconstruction problems. For complex, unknown samples, it would be interesting to use profiles from known mitochondrial genomes to assemble reads on these profiles - the number of different species/contigs could then be estimated from counts of different reads/contigs within a sliding window along the profile(s), or even without any bioinformatics consolidation at all [93]. However such assembly tools have yet to be developed.

While this approach is not appropriate for all situations, it provides a solution for a large number of cases that were until now technically problematic. There are also economic considerations, as the approach does not require sample tagging, which can represent a considerable part of the sequencing cost. The approach is not sequencer or kit specific, and can be adapted to the availability of each, although the sequencers generating longer sequences will give better results even at lower coverages, as will the use of paired ends. One requirement, however, is the use of well-preserved biological samples, containing complete mitochondrial molecules.

The need to mix numerous samples with divergent sequences makes our proposal of little interest for research groups working on a small number of specimens and/or closely related taxa. However, many laboratories, especially in the phylogenetics and biodiversity field, include a number of researchers working on multiple different taxa, or have set up projects to characterize the molecular diversity of multiple taxonomic groups in large numbers. Pooling very distinct taxonomic entities at the sequencing stage generates large mixed libraries with highly divergent mitochondrial sequences, ideal for *a posteriori* sorting and unequivocal assignment. Pricewise, complete mitogenomes with all their versatility could be generated for a price barely above that of a two directional Sanger read for a single PCR (table 2), or maybe even lower.

However, this approach is also highly interesting for metagenomic studies when high quality DNA is available, as is the case for recent plankton and interstitial fauna studies, and it would provide a welcome complement to 18S rDNA based assessments, providing a second marker with different evolutionary speed and heritability, using already constituted large reference datasets. For studies based on degraded DNA, availability of reference identification datasets covering the whole mitogenome would provide sequence data to explore alternative markers more suited to each group, and help in the development of primers for divergent groups [93].

## Supporting Information

File S1 **Mitogenome sequence dataset used in the assembly simulations.**
(TXT)

File S2 **Sliding window analyses for Annelida, Cnidaria, Crustacea, Echinodermata, Mollusca, Nematoda, Nemertea, Platyhelminthes and Porifera.** For each family, the folder contains the aligned sequences as well as the sliding window analyses by species pair and for all species pair on a single figure.
(ZIP)

File S3 **Sliding window analyses for Mammalia and Lissamphibia.** For each family, the folder contains the aligned sequences as well as the sliding window analyses by species pair and for all species pair on a single figure.
(ZIP)

File S4 **Sliding window analyses for Sauropsida, Aves, Hemichordata, Coelacanthimorpha, Dipnoi, Chondrichthyes and Cephalochordata.** For each family, the folder contains the aligned sequences as well as the sliding window analyses by species pair and for all species pair on a single figure.
(ZIP)
