# Correction: Conveniently Pre-Tagged and Pre-Packaged: Extended Molecular Identification and Metagenomics Using Complete Metazoan Mitochondrial Genomes

**DOI:** 10.1371/annotation/020848d7-0453-42df-bf94-40eb39f1b27a

**Published:** 2013-03-11

**Authors:** Agnes Dettai, Cyril Gallut, Sophie Brouillet, Joel Pothier, Guillaume Lecointre, Régis Debruyne

Since the publication of our article, we have been made aware by Prof. Eisen of a publication by Pollock and colleagues as early as 2000 in Molecular Biology and Evolution with which our approach shows some remarkable points of convergence, the publication details are below:

Pollock DD, Eisen JA, Doggett NA, Cummings MP. 2000. A case for evolutionary genomics and the comprehensive examination of sequence biodiversity. Mol Biol Evol 7: 1776-1788.

We also had overlooked a publication by Timmermans and colleagues (2010) in Nucleic Acids Research describing a case study based on similar principles regarding Coleoptera:

Timmermans MJ, Dodsworth S, Culverwell CL, Bocak L, Ahrens D, Littlewood DT, Pons J, Vogler AP (2010) Why barcode? High-throughput multiplex sequencing of mitochondrial genomes for molecular systematics. Nucleic Acids Res 38(21): e197. doi: 10.1093/nar/gkq807.

Both publications should have been cited and we apologize for missing them. However, our work presents an in-depth exploration of the approach for Metazoans, giving as many leads as possible on its limits and pitfalls as well as covering a large array of sequencing platforms and taxonomic groups, and so there are numerous developments addressed in our article that are not present in the other two.

In the light of the previous publications by Pollock et al. and Timmermans et al., the occurrences of "our approach" in the text should be revised to read "the approach", in order to recognize that although we developed it independently, we were not the first to do so. Like us, both Pollock et al. and Timmermans et al. promote an approach based on pooling independent organisms prior to amplification and sequencing without any external individual tagging. It then relies on diverging sequence content to computationally demultiplex the sequencing products. Both publications concur with the approach we presented in the article.

The article of Pollock and colleagues (2000) describes the principle and provides some in silico analyses for the 69 vertebrate mitogenomes available at the time, and is based on that period's technologies (cloning, high throughput sequencing, and automation). For the last decade however, the field of genomics has seen a huge methodological shift towards massive parallel sequencing of short, clonally amplified, genome fragments using NGS technologies. Our current study does not only rest on the original underlying concept, but on an operational in-silico validation test of the methodological approach based on the currently available next-generation sequencing desktop platforms, using their specificity to address their relative relevance in this framework. We also extend the work to all of Metazoans using the thousands of available mitochondrial genomes and knowledge accumulated on their evolution and phylogenetic use in the last twelve years.

The case study of Timmermans and colleagues (2010) does provide a proof of concept of the approach using a single taxonomic group, a single sequencing platform (454/Roche) and a single sequence selection technique (long PCRs). We made in silico comparisons of several platforms to provide some elements for an optimal platform choice based on their characteristics, including sequence volume and price. We compared mitochondrial genome sequences for a large range of Metazoan groups to make a systematic evaluation of the level of divergence necessary for correct demultiplexing, and alternatives to PCR amplification for sequence selection.

The fact that the three studies report such convergent views on how to improve production of comparative genomics data and minimize the amount of work and related costs over such a gap of technological availability provides support for the soundness of the approaches, which might improve our practice of sequencing technologies in the realm of comparative genomics of non-model organisms, as well as environmental samples. 

