# Supplementary figures and images for: Conveniently Pre-Tagged and Pre-Packaged: Extended Molecular Identification and Metagenomics Using Complete Metazoan Mitochondrial Genomes (part 1 of 3)
Source: PLoS One. 2012 Dec 14;7(12):e51263. doi: 10.1371/journal.pone.0051263 (PMC3522660; doi:10.1371/journal.pone.0051263)

# Urechidae

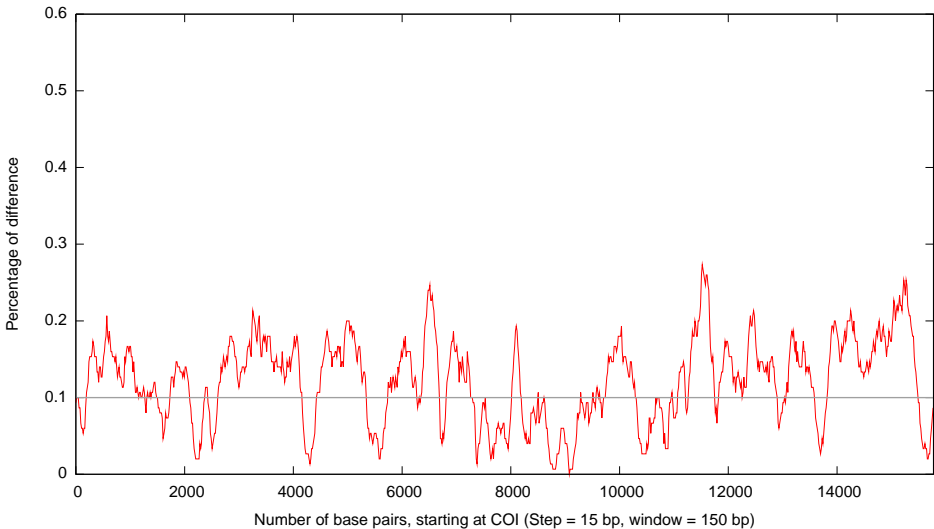

Supplement: File S2 — Sliding window analyses for Annelida, Cnidaria, Crustacea, Echinodermata, Mollusca, Nematoda, Nemertea, Platyhelminthes and Porifera. For each family, the folder contains the aligned sequences as well as the sliding window analyses by species pair and for all species pair on a single figure. (ZIP) [file pone.0051263.s002.zip › Annelida/Urechidae/15_150/allCurves.pdf]

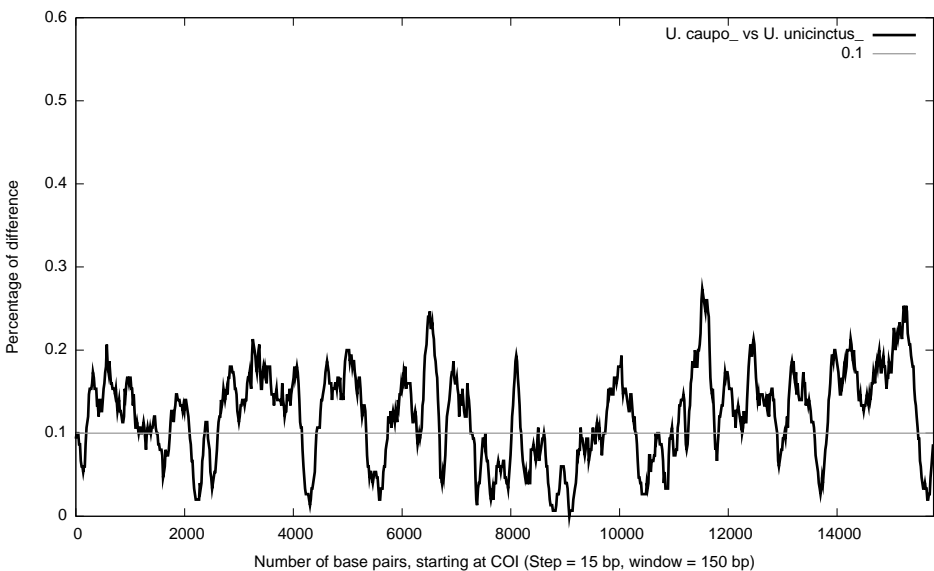

Supplement: File S2 — Sliding window analyses for Annelida, Cnidaria, Crustacea, Echinodermata, Mollusca, Nematoda, Nemertea, Platyhelminthes and Porifera. For each family, the folder contains the aligned sequences as well as the sliding window analyses by species pair and for all species pair on a single figure. (ZIP) [file pone.0051263.s002.zip › Annelida/Urechidae/15_150/Urechis_caupo_NC_006379_Urechis_unicinctus_NC_012768.pdf]

# Urechidae

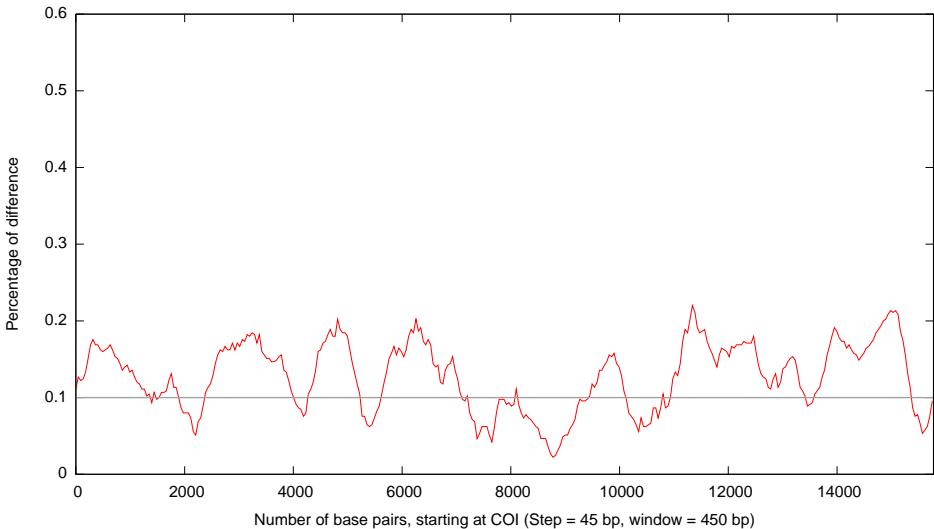

Supplement: File S2 — Sliding window analyses for Annelida, Cnidaria, Crustacea, Echinodermata, Mollusca, Nematoda, Nemertea, Platyhelminthes and Porifera. For each family, the folder contains the aligned sequences as well as the sliding window analyses by species pair and for all species pair on a single figure. (ZIP) [file pone.0051263.s002.zip › Annelida/Urechidae/45_450/allCurves.pdf]

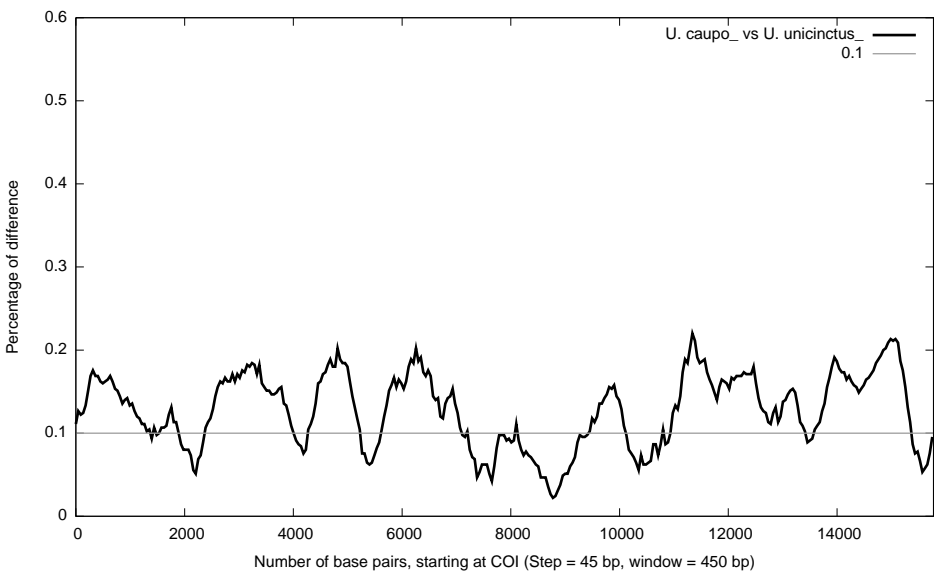

Supplement: File S2 — Sliding window analyses for Annelida, Cnidaria, Crustacea, Echinodermata, Mollusca, Nematoda, Nemertea, Platyhelminthes and Porifera. For each family, the folder contains the aligned sequences as well as the sliding window analyses by species pair and for all species pair on a single figure. (ZIP) [file pone.0051263.s002.zip › Annelida/Urechidae/45_450/Urechis_caupo_NC_006379_Urechis_unicinctus_NC_012768.pdf]

# Faviidae

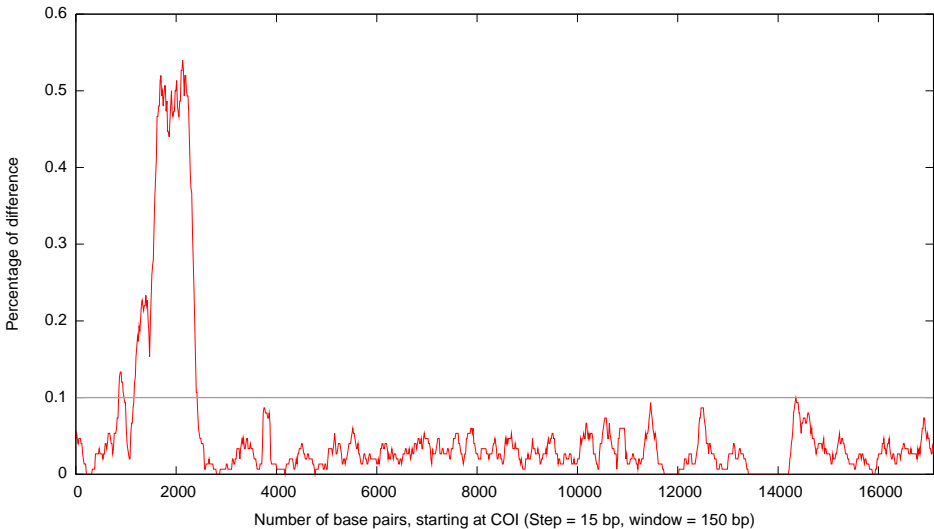

Supplement: File S2 — Sliding window analyses for Annelida, Cnidaria, Crustacea, Echinodermata, Mollusca, Nematoda, Nemertea, Platyhelminthes and Porifera. For each family, the folder contains the aligned sequences as well as the sliding window analyses by species pair and for all species pair on a single figure. (ZIP) [file pone.0051263.s002.zip › Cnidaria/Faviidae/15_150/allCurves.pdf]

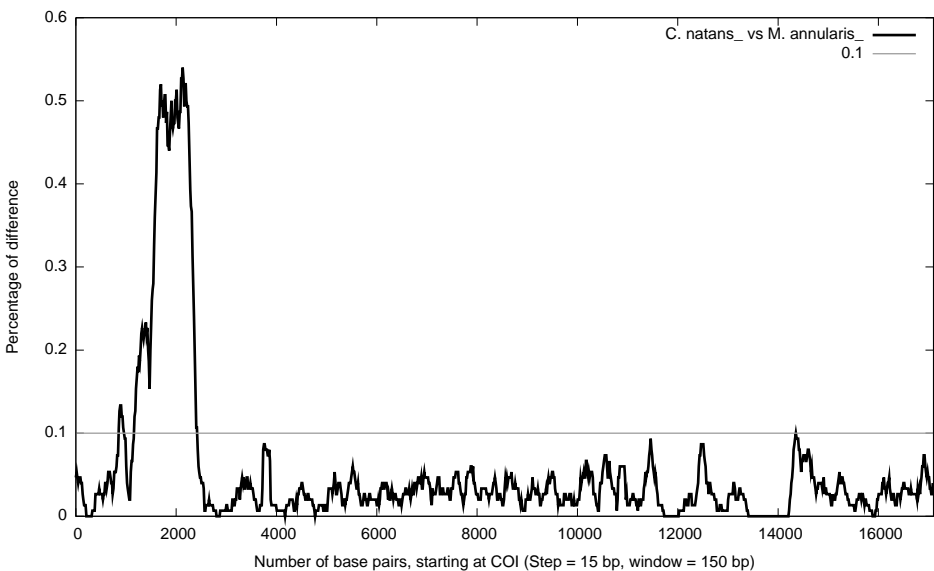

Supplement: File S2 — Sliding window analyses for Annelida, Cnidaria, Crustacea, Echinodermata, Mollusca, Nematoda, Nemertea, Platyhelminthes and Porifera. For each family, the folder contains the aligned sequences as well as the sliding window analyses by species pair and for all species pair on a single figure. (ZIP) [file pone.0051263.s002.zip › Cnidaria/Faviidae/15_150/Colpophyllia_natans_NC_008162_Montastraea_annularis_NC_007224.pdf]

# Faviidae

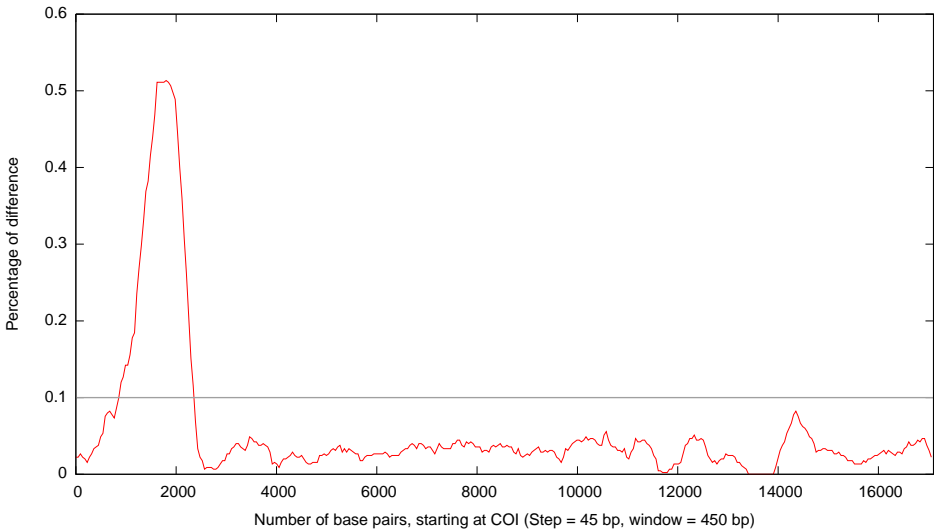

Supplement: File S2 — Sliding window analyses for Annelida, Cnidaria, Crustacea, Echinodermata, Mollusca, Nematoda, Nemertea, Platyhelminthes and Porifera. For each family, the folder contains the aligned sequences as well as the sliding window analyses by species pair and for all species pair on a single figure. (ZIP) [file pone.0051263.s002.zip › Cnidaria/Faviidae/45_450/allCurves.pdf]

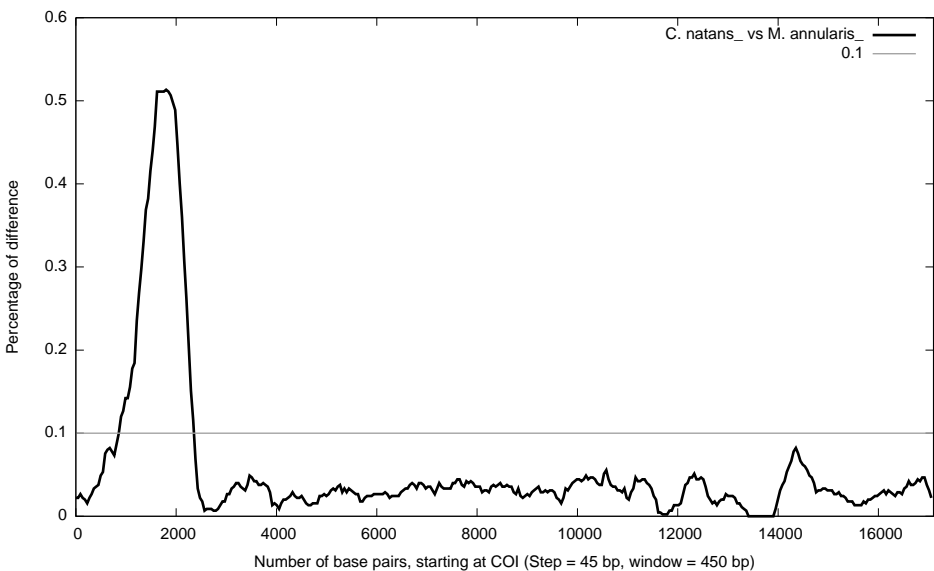

Supplement: File S2 — Sliding window analyses for Annelida, Cnidaria, Crustacea, Echinodermata, Mollusca, Nematoda, Nemertea, Platyhelminthes and Porifera. For each family, the folder contains the aligned sequences as well as the sliding window analyses by species pair and for all species pair on a single figure. (ZIP) [file pone.0051263.s002.zip › Cnidaria/Faviidae/45_450/Colpophyllia_natans_NC_008162_Montastraea_annularis_NC_007224.pdf]

# Pocilloporidae

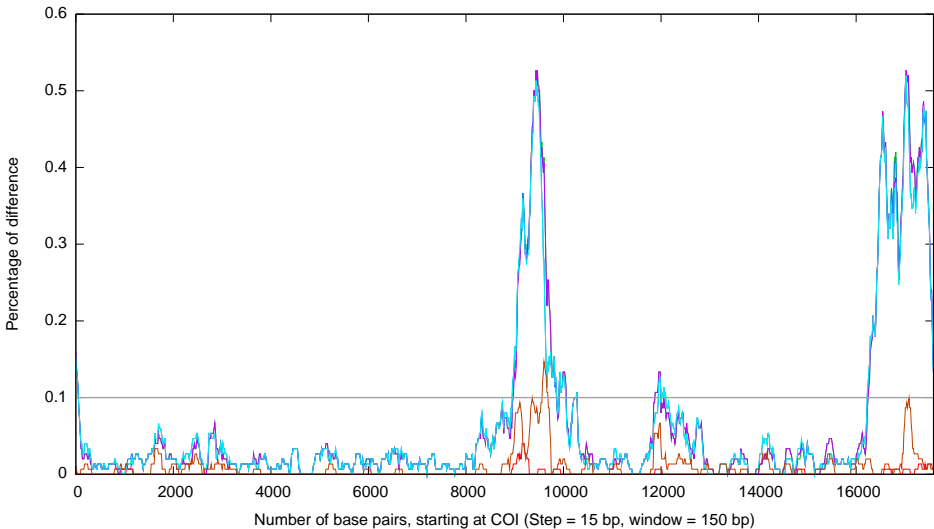

Supplement: File S2 — Sliding window analyses for Annelida, Cnidaria, Crustacea, Echinodermata, Mollusca, Nematoda, Nemertea, Platyhelminthes and Porifera. For each family, the folder contains the aligned sequences as well as the sliding window analyses by species pair and for all species pair on a single figure. (ZIP) [file pone.0051263.s002.zip › Cnidaria/Pocilloporidae/15_150/allCurves.pdf]

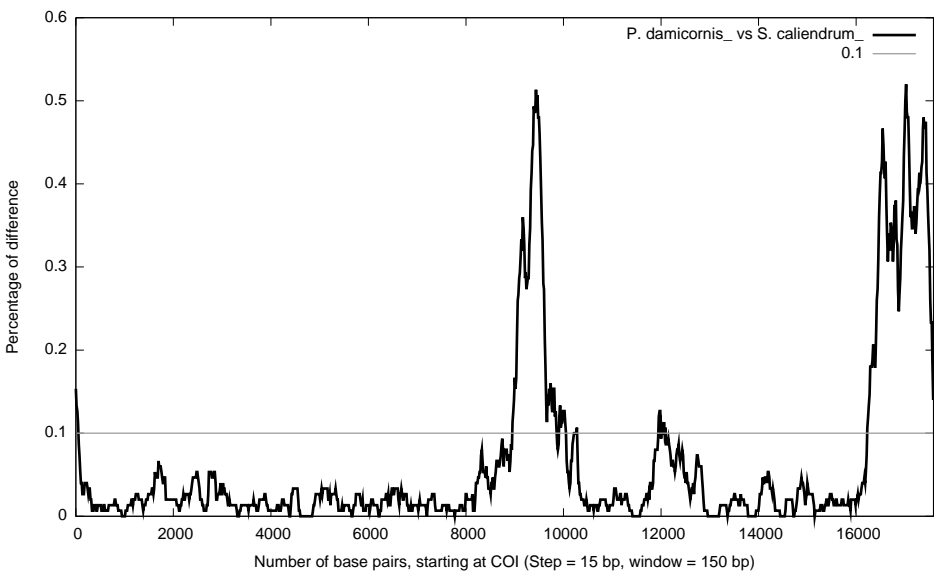

Supplement: File S2 — Sliding window analyses for Annelida, Cnidaria, Crustacea, Echinodermata, Mollusca, Nematoda, Nemertea, Platyhelminthes and Porifera. For each family, the folder contains the aligned sequences as well as the sliding window analyses by species pair and for all species pair on a single figure. (ZIP) [file pone.0051263.s002.zip › Cnidaria/Pocilloporidae/15_150/Pocillopora_damicornis_NC_009797_Seriatopora_caliendrum_NC_010245.pdf]

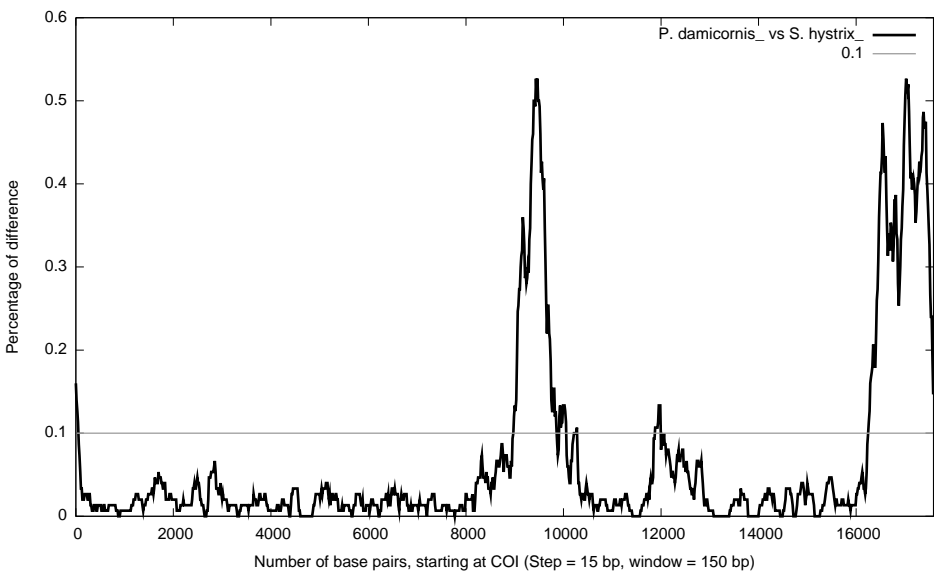

Supplement: File S2 — Sliding window analyses for Annelida, Cnidaria, Crustacea, Echinodermata, Mollusca, Nematoda, Nemertea, Platyhelminthes and Porifera. For each family, the folder contains the aligned sequences as well as the sliding window analyses by species pair and for all species pair on a single figure. (ZIP) [file pone.0051263.s002.zip › Cnidaria/Pocilloporidae/15_150/Pocillopora_damicornis_NC_009797_Seriatopora_hystrix_NC_010244.pdf]

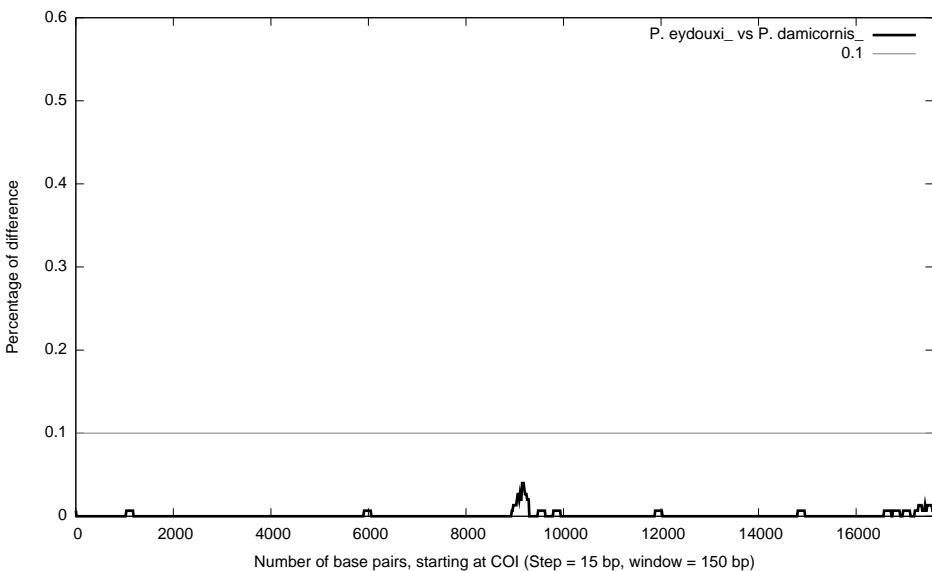

Supplement: File S2 — Sliding window analyses for Annelida, Cnidaria, Crustacea, Echinodermata, Mollusca, Nematoda, Nemertea, Platyhelminthes and Porifera. For each family, the folder contains the aligned sequences as well as the sliding window analyses by species pair and for all species pair on a single figure. (ZIP) [file pone.0051263.s002.zip › Cnidaria/Pocilloporidae/15_150/Pocillopora_eydouxi_NC_009798_Pocillopora_damicornis_NC_009797.pdf]

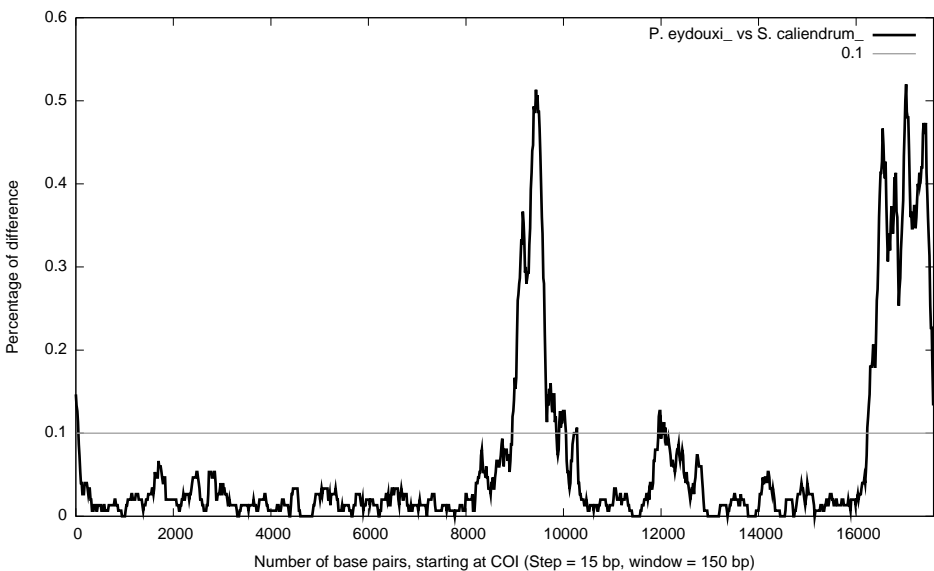

Supplement: File S2 — Sliding window analyses for Annelida, Cnidaria, Crustacea, Echinodermata, Mollusca, Nematoda, Nemertea, Platyhelminthes and Porifera. For each family, the folder contains the aligned sequences as well as the sliding window analyses by species pair and for all species pair on a single figure. (ZIP) [file pone.0051263.s002.zip › Cnidaria/Pocilloporidae/15_150/Pocillopora_eydouxi_NC_009798_Seriatopora_caliendrum_NC_010245.pdf]

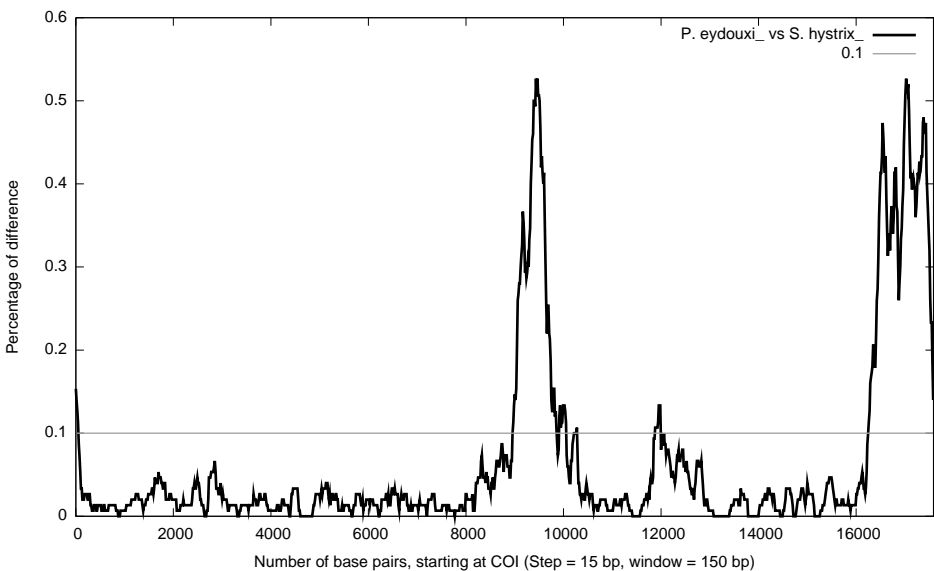

Supplement: File S2 — Sliding window analyses for Annelida, Cnidaria, Crustacea, Echinodermata, Mollusca, Nematoda, Nemertea, Platyhelminthes and Porifera. For each family, the folder contains the aligned sequences as well as the sliding window analyses by species pair and for all species pair on a single figure. (ZIP) [file pone.0051263.s002.zip › Cnidaria/Pocilloporidae/15_150/Pocillopora_eydouxi_NC_009798_Seriatopora_hystrix_NC_010244.pdf]

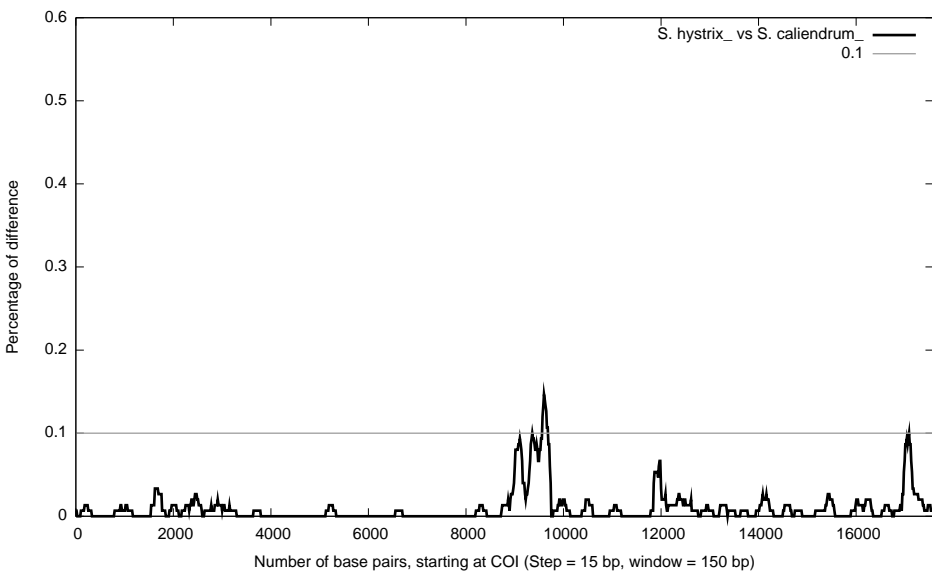

Supplement: File S2 — Sliding window analyses for Annelida, Cnidaria, Crustacea, Echinodermata, Mollusca, Nematoda, Nemertea, Platyhelminthes and Porifera. For each family, the folder contains the aligned sequences as well as the sliding window analyses by species pair and for all species pair on a single figure. (ZIP) [file pone.0051263.s002.zip › Cnidaria/Pocilloporidae/15_150/Seriatopora_hystrix_NC_010244_Seriatopora_caliendrum_NC_010245.pdf]

# Pocilloporidae

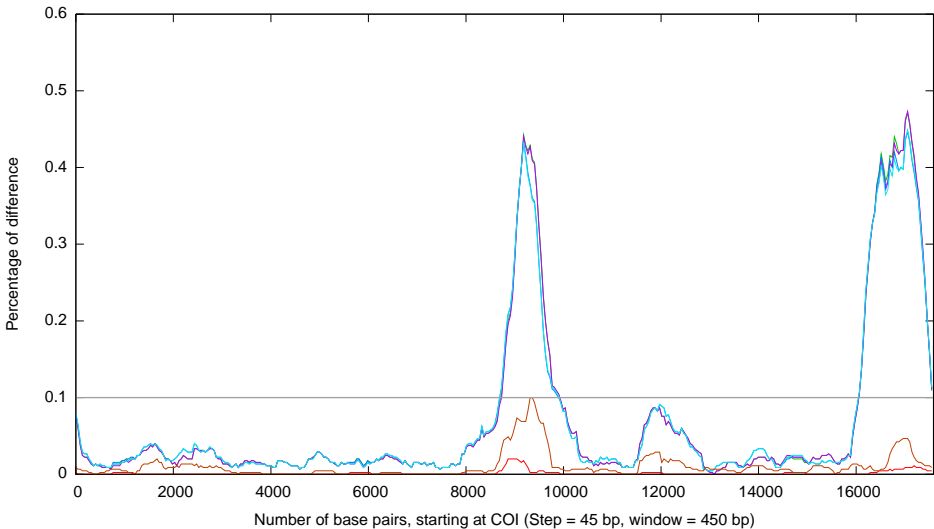

Supplement: File S2 — Sliding window analyses for Annelida, Cnidaria, Crustacea, Echinodermata, Mollusca, Nematoda, Nemertea, Platyhelminthes and Porifera. For each family, the folder contains the aligned sequences as well as the sliding window analyses by species pair and for all species pair on a single figure. (ZIP) [file pone.0051263.s002.zip › Cnidaria/Pocilloporidae/45_450/allCurves.pdf]

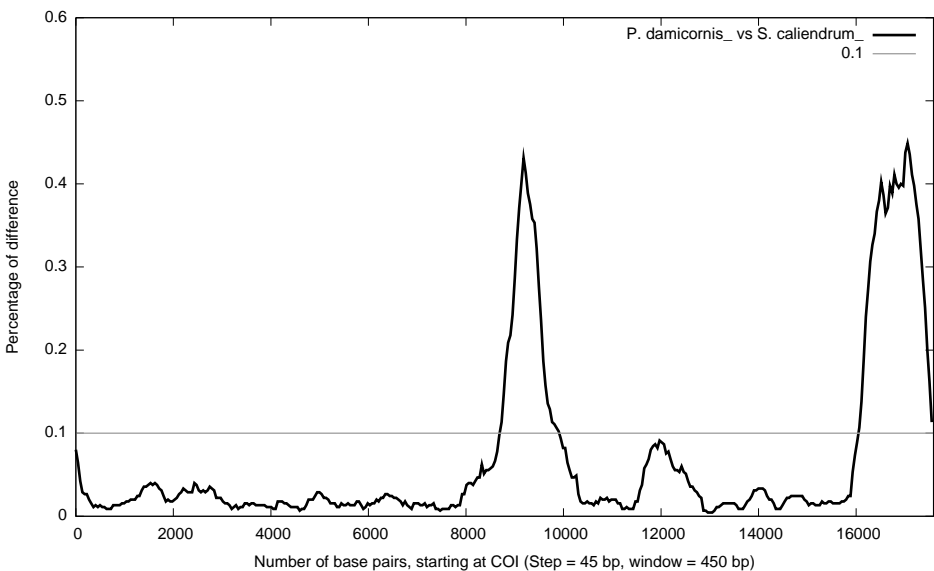

Supplement: File S2 — Sliding window analyses for Annelida, Cnidaria, Crustacea, Echinodermata, Mollusca, Nematoda, Nemertea, Platyhelminthes and Porifera. For each family, the folder contains the aligned sequences as well as the sliding window analyses by species pair and for all species pair on a single figure. (ZIP) [file pone.0051263.s002.zip › Cnidaria/Pocilloporidae/45_450/Pocillopora_damicornis_NC_009797_Seriatopora_caliendrum_NC_010245.pdf]

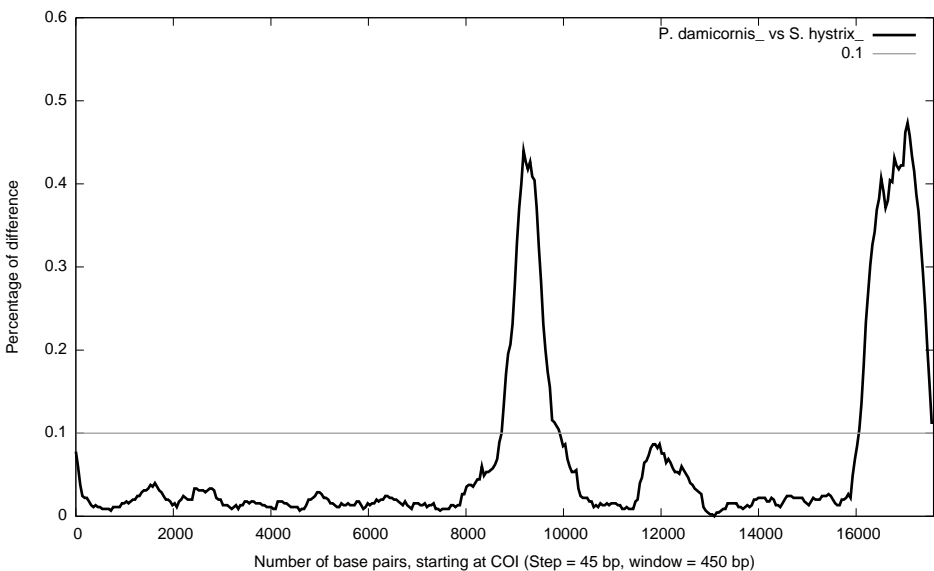

Supplement: File S2 — Sliding window analyses for Annelida, Cnidaria, Crustacea, Echinodermata, Mollusca, Nematoda, Nemertea, Platyhelminthes and Porifera. For each family, the folder contains the aligned sequences as well as the sliding window analyses by species pair and for all species pair on a single figure. (ZIP) [file pone.0051263.s002.zip › Cnidaria/Pocilloporidae/45_450/Pocillopora_damicornis_NC_009797_Seriatopora_hystrix_NC_010244.pdf]

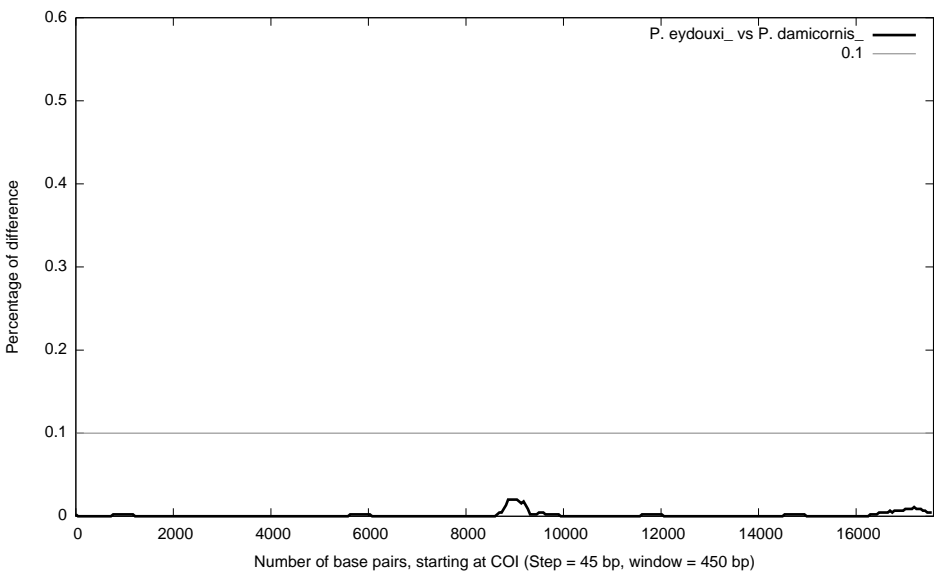

Supplement: File S2 — Sliding window analyses for Annelida, Cnidaria, Crustacea, Echinodermata, Mollusca, Nematoda, Nemertea, Platyhelminthes and Porifera. For each family, the folder contains the aligned sequences as well as the sliding window analyses by species pair and for all species pair on a single figure. (ZIP) [file pone.0051263.s002.zip › Cnidaria/Pocilloporidae/45_450/Pocillopora_eydouxi_NC_009798_Pocillopora_damicornis_NC_009797.pdf]

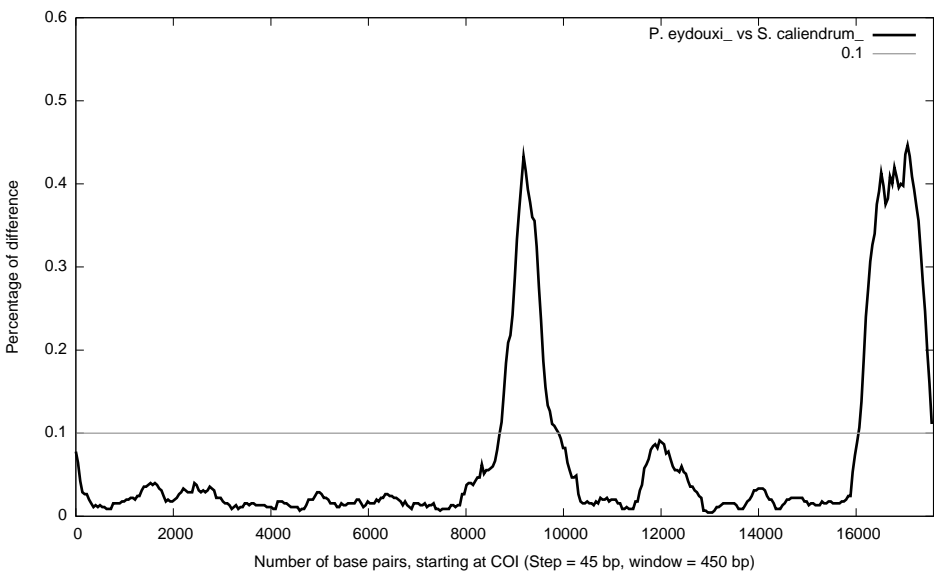

Supplement: File S2 — Sliding window analyses for Annelida, Cnidaria, Crustacea, Echinodermata, Mollusca, Nematoda, Nemertea, Platyhelminthes and Porifera. For each family, the folder contains the aligned sequences as well as the sliding window analyses by species pair and for all species pair on a single figure. (ZIP) [file pone.0051263.s002.zip › Cnidaria/Pocilloporidae/45_450/Pocillopora_eydouxi_NC_009798_Seriatopora_caliendrum_NC_010245.pdf]

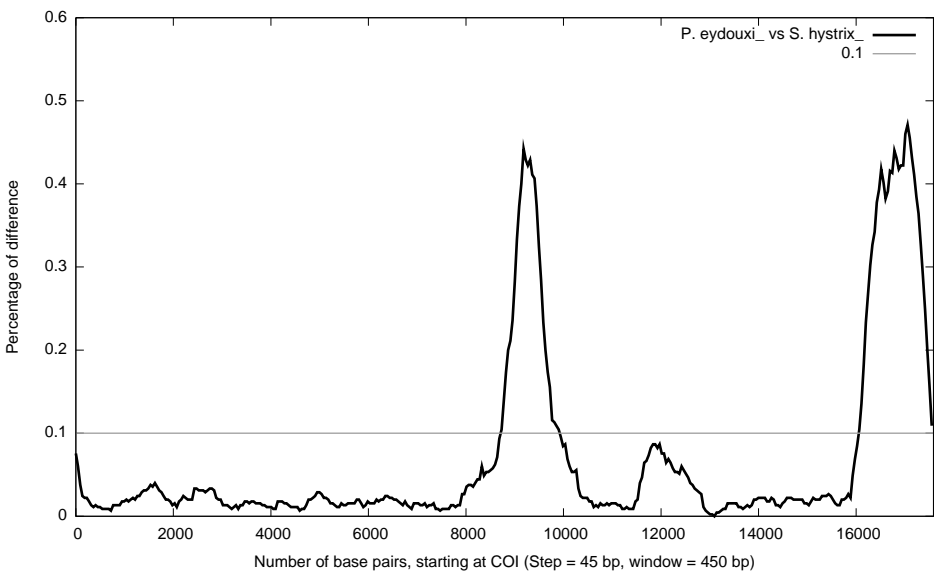

Supplement: File S2 — Sliding window analyses for Annelida, Cnidaria, Crustacea, Echinodermata, Mollusca, Nematoda, Nemertea, Platyhelminthes and Porifera. For each family, the folder contains the aligned sequences as well as the sliding window analyses by species pair and for all species pair on a single figure. (ZIP) [file pone.0051263.s002.zip › Cnidaria/Pocilloporidae/45_450/Pocillopora_eydouxi_NC_009798_Seriatopora_hystrix_NC_010244.pdf]

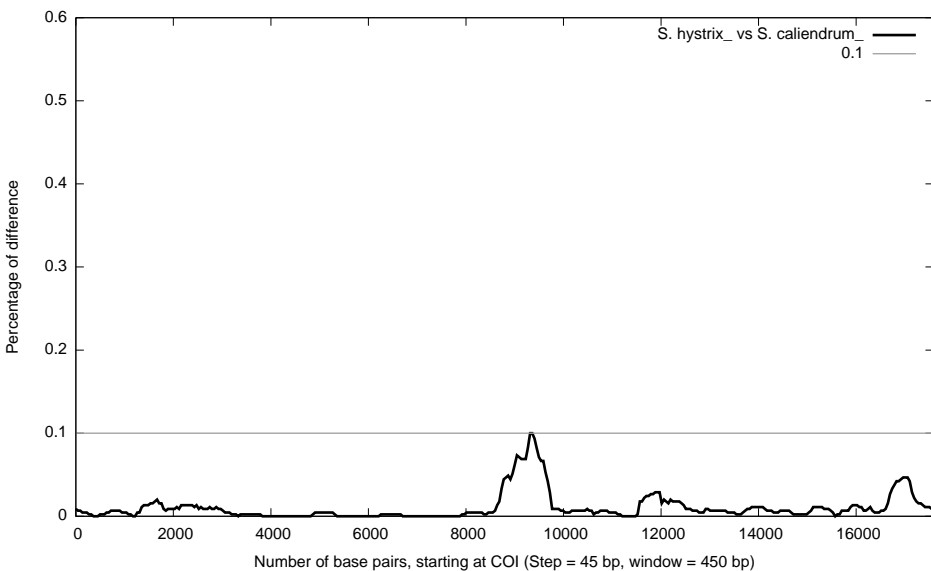

Supplement: File S2 — Sliding window analyses for Annelida, Cnidaria, Crustacea, Echinodermata, Mollusca, Nematoda, Nemertea, Platyhelminthes and Porifera. For each family, the folder contains the aligned sequences as well as the sliding window analyses by species pair and for all species pair on a single figure. (ZIP) [file pone.0051263.s002.zip › Cnidaria/Pocilloporidae/45_450/Seriatopora_hystrix_NC_010244_Seriatopora_caliendrum_NC_010245.pdf]

# Poritidae

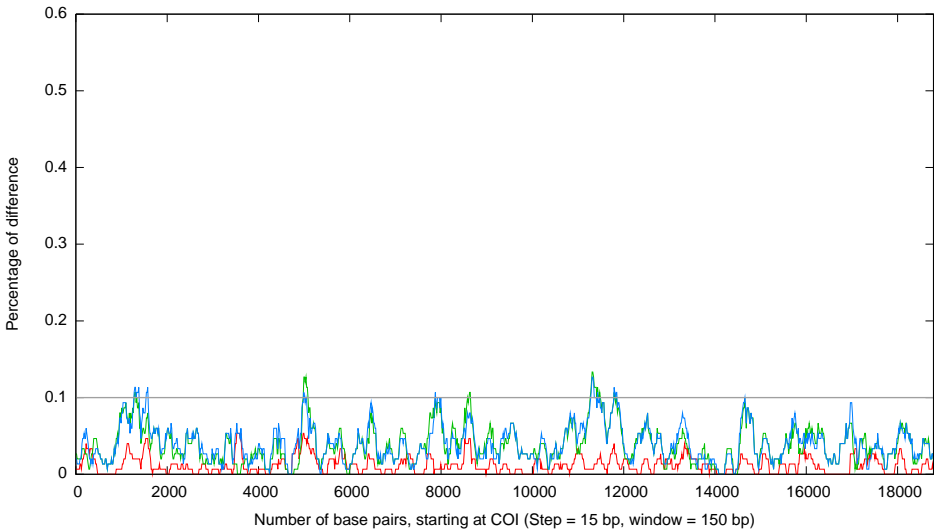

Supplement: File S2 — Sliding window analyses for Annelida, Cnidaria, Crustacea, Echinodermata, Mollusca, Nematoda, Nemertea, Platyhelminthes and Porifera. For each family, the folder contains the aligned sequences as well as the sliding window analyses by species pair and for all species pair on a single figure. (ZIP) [file pone.0051263.s002.zip › Cnidaria/Poritidae/15_150/allCurves.pdf]

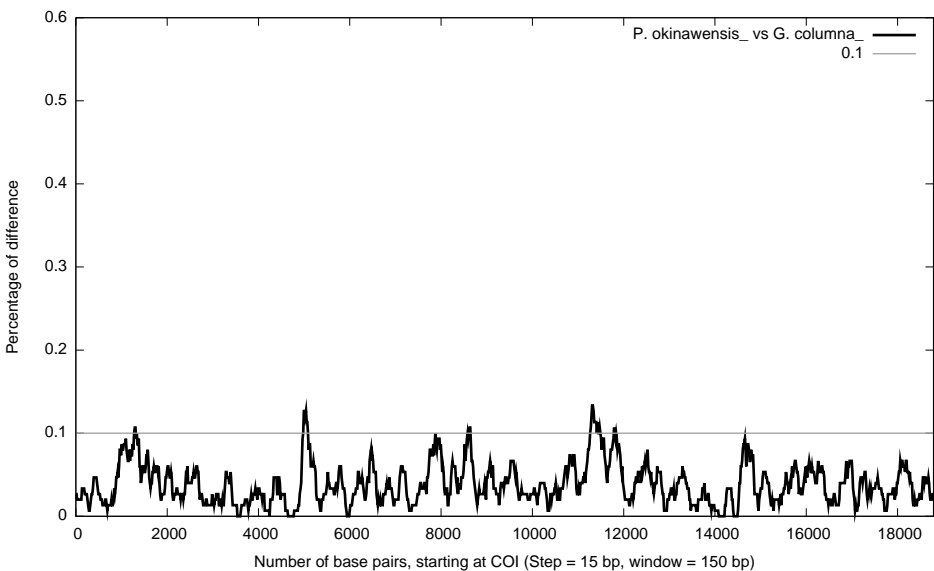

Supplement: File S2 — Sliding window analyses for Annelida, Cnidaria, Crustacea, Echinodermata, Mollusca, Nematoda, Nemertea, Platyhelminthes and Porifera. For each family, the folder contains the aligned sequences as well as the sliding window analyses by species pair and for all species pair on a single figure. (ZIP) [file pone.0051263.s002.zip › Cnidaria/Poritidae/15_150/Porites_okinawensis_NC_015644_Goniopora_columna_NC_015643.pdf]

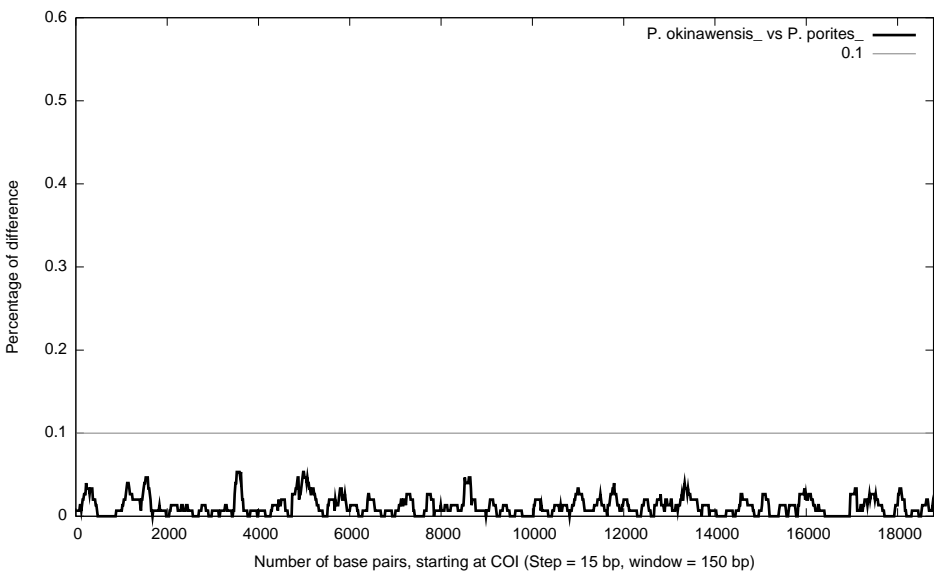

Supplement: File S2 — Sliding window analyses for Annelida, Cnidaria, Crustacea, Echinodermata, Mollusca, Nematoda, Nemertea, Platyhelminthes and Porifera. For each family, the folder contains the aligned sequences as well as the sliding window analyses by species pair and for all species pair on a single figure. (ZIP) [file pone.0051263.s002.zip › Cnidaria/Poritidae/15_150/Porites_okinawensis_NC_015644_Porites_porites_NC_008166.pdf]

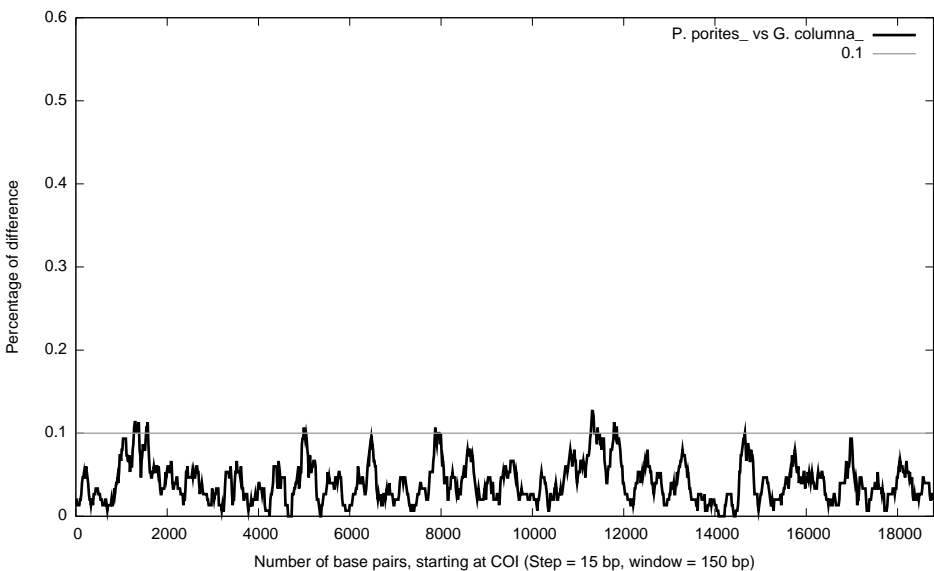

Supplement: File S2 — Sliding window analyses for Annelida, Cnidaria, Crustacea, Echinodermata, Mollusca, Nematoda, Nemertea, Platyhelminthes and Porifera. For each family, the folder contains the aligned sequences as well as the sliding window analyses by species pair and for all species pair on a single figure. (ZIP) [file pone.0051263.s002.zip › Cnidaria/Poritidae/15_150/Porites_porites_NC_008166_Goniopora_columna_NC_015643.pdf]

# Poritidae

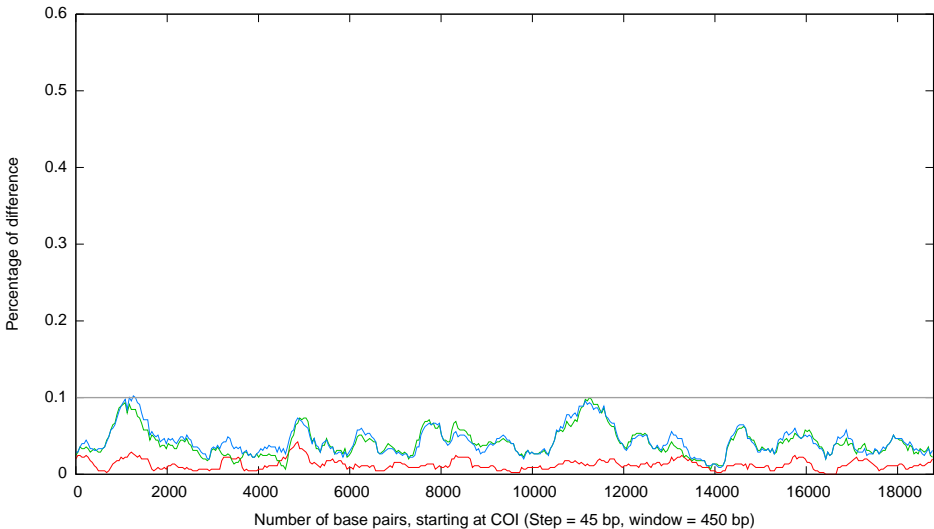

Supplement: File S2 — Sliding window analyses for Annelida, Cnidaria, Crustacea, Echinodermata, Mollusca, Nematoda, Nemertea, Platyhelminthes and Porifera. For each family, the folder contains the aligned sequences as well as the sliding window analyses by species pair and for all species pair on a single figure. (ZIP) [file pone.0051263.s002.zip › Cnidaria/Poritidae/45_450/allCurves.pdf]

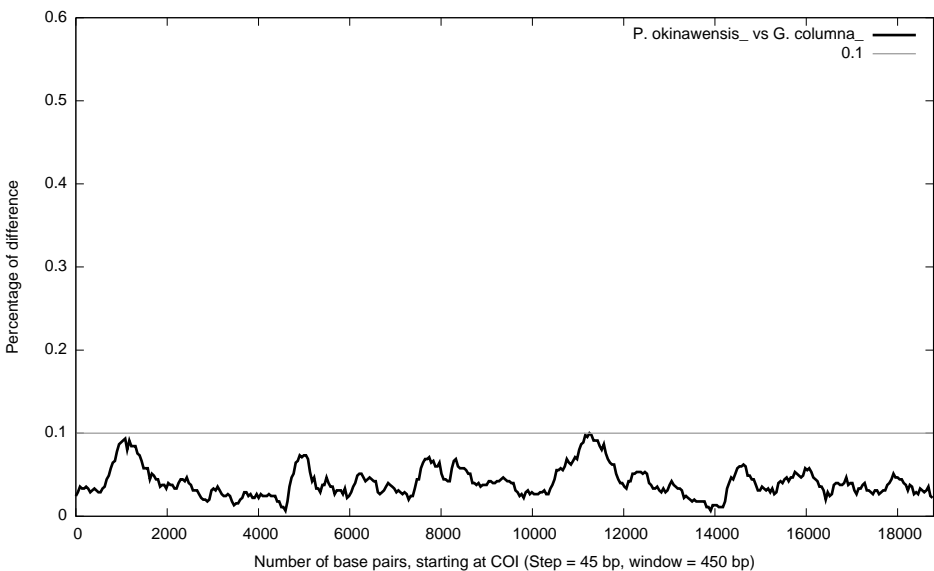

Supplement: File S2 — Sliding window analyses for Annelida, Cnidaria, Crustacea, Echinodermata, Mollusca, Nematoda, Nemertea, Platyhelminthes and Porifera. For each family, the folder contains the aligned sequences as well as the sliding window analyses by species pair and for all species pair on a single figure. (ZIP) [file pone.0051263.s002.zip › Cnidaria/Poritidae/45_450/Porites_okinawensis_NC_015644_Goniopora_columna_NC_015643.pdf]

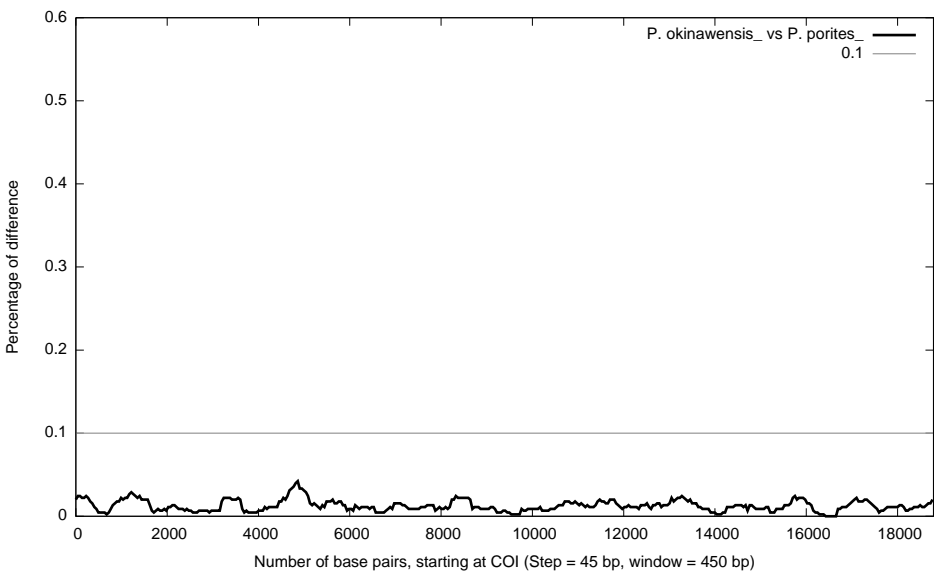

Supplement: File S2 — Sliding window analyses for Annelida, Cnidaria, Crustacea, Echinodermata, Mollusca, Nematoda, Nemertea, Platyhelminthes and Porifera. For each family, the folder contains the aligned sequences as well as the sliding window analyses by species pair and for all species pair on a single figure. (ZIP) [file pone.0051263.s002.zip › Cnidaria/Poritidae/45_450/Porites_okinawensis_NC_015644_Porites_porites_NC_008166.pdf]

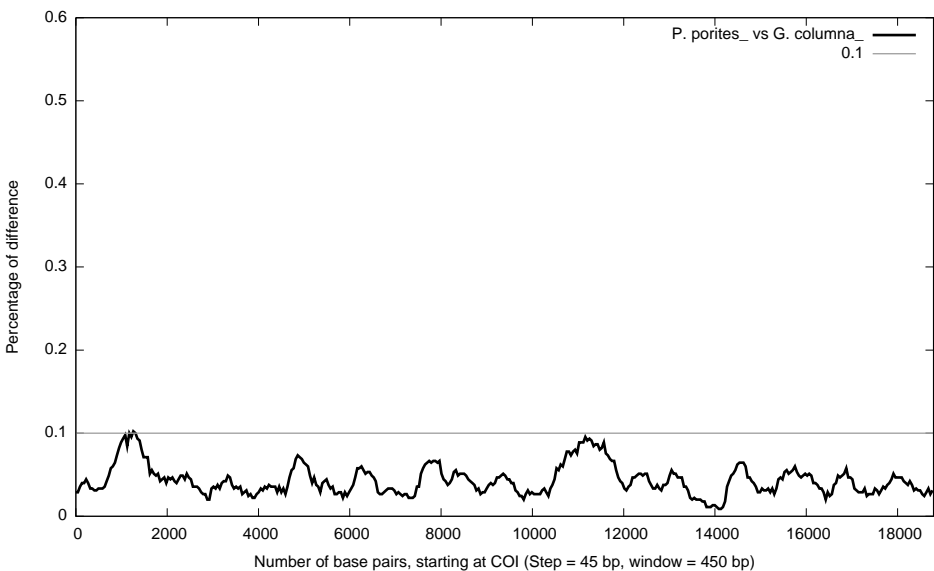

Supplement: File S2 — Sliding window analyses for Annelida, Cnidaria, Crustacea, Echinodermata, Mollusca, Nematoda, Nemertea, Platyhelminthes and Porifera. For each family, the folder contains the aligned sequences as well as the sliding window analyses by species pair and for all species pair on a single figure. (ZIP) [file pone.0051263.s002.zip › Cnidaria/Poritidae/45_450/Porites_porites_NC_008166_Goniopora_columna_NC_015643.pdf]

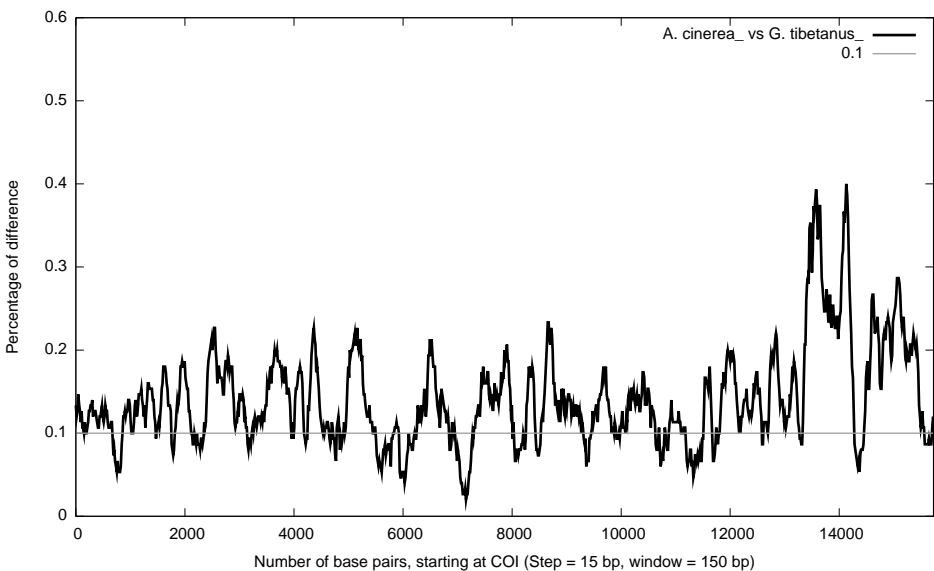

Supplement: File S2 — Sliding window analyses for Annelida, Cnidaria, Crustacea, Echinodermata, Mollusca, Nematoda, Nemertea, Platyhelminthes and Porifera. For each family, the folder contains the aligned sequences as well as the sliding window analyses by species pair and for all species pair on a single figure. (ZIP) [file pone.0051263.s002.zip › Crustacea/Acrididae/15_150/Acrida_cinerea_NC_014887_Gomphocerus_sibiricus_tibetanus_NC_015478.pdf]

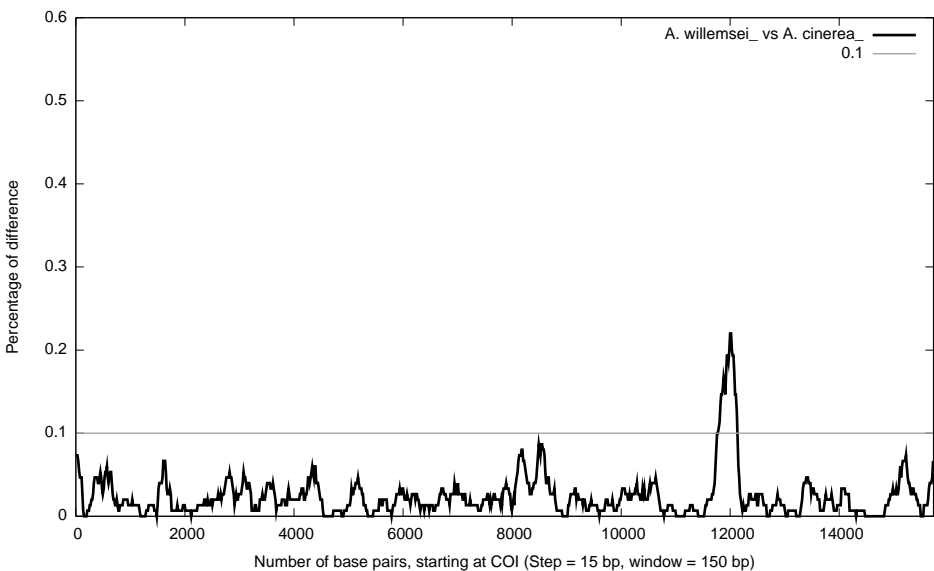

Supplement: File S2 — Sliding window analyses for Annelida, Cnidaria, Crustacea, Echinodermata, Mollusca, Nematoda, Nemertea, Platyhelminthes and Porifera. For each family, the folder contains the aligned sequences as well as the sliding window analyses by species pair and for all species pair on a single figure. (ZIP) [file pone.0051263.s002.zip › Crustacea/Acrididae/15_150/Acrida_willemsei_NC_011303_Acrida_cinerea_NC_014887.pdf]

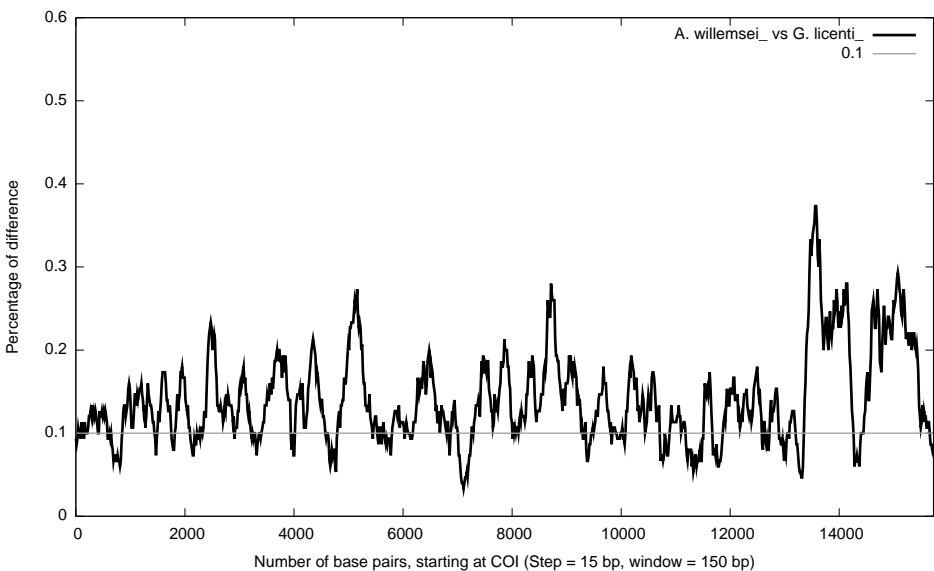

Supplement: File S2 — Sliding window analyses for Annelida, Cnidaria, Crustacea, Echinodermata, Mollusca, Nematoda, Nemertea, Platyhelminthes and Porifera. For each family, the folder contains the aligned sequences as well as the sliding window analyses by species pair and for all species pair on a single figure. (ZIP) [file pone.0051263.s002.zip › Crustacea/Acrididae/15_150/Acrida_willemsei_NC_011303_Gomphocerus_licenti_NC_013847.pdf]

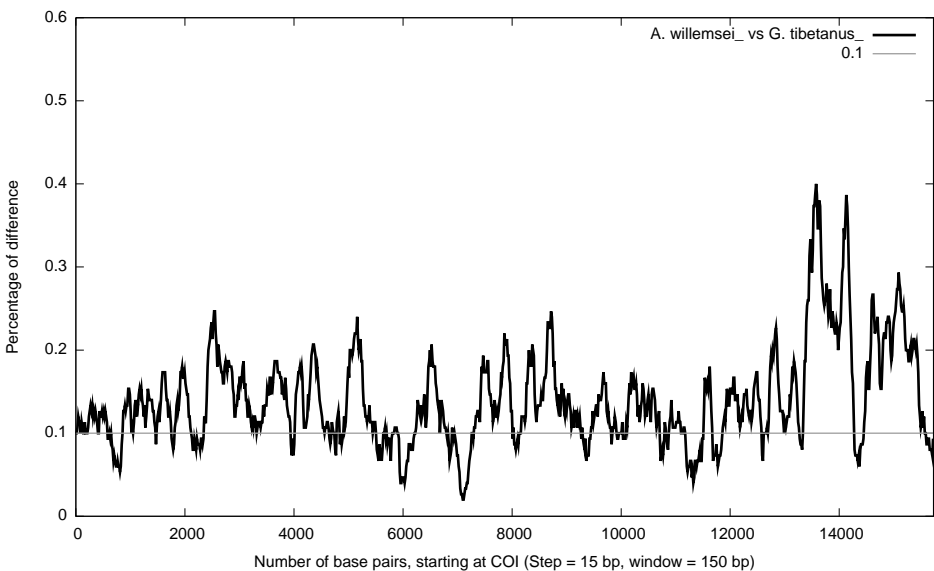

Supplement: File S2 — Sliding window analyses for Annelida, Cnidaria, Crustacea, Echinodermata, Mollusca, Nematoda, Nemertea, Platyhelminthes and Porifera. For each family, the folder contains the aligned sequences as well as the sliding window analyses by species pair and for all species pair on a single figure. (ZIP) [file pone.0051263.s002.zip › Crustacea/Acrididae/15_150/Acrida_willemsei_NC_011303_Gomphocerus_sibiricus_tibetanus_NC_015478.pdf]

# Acrididae

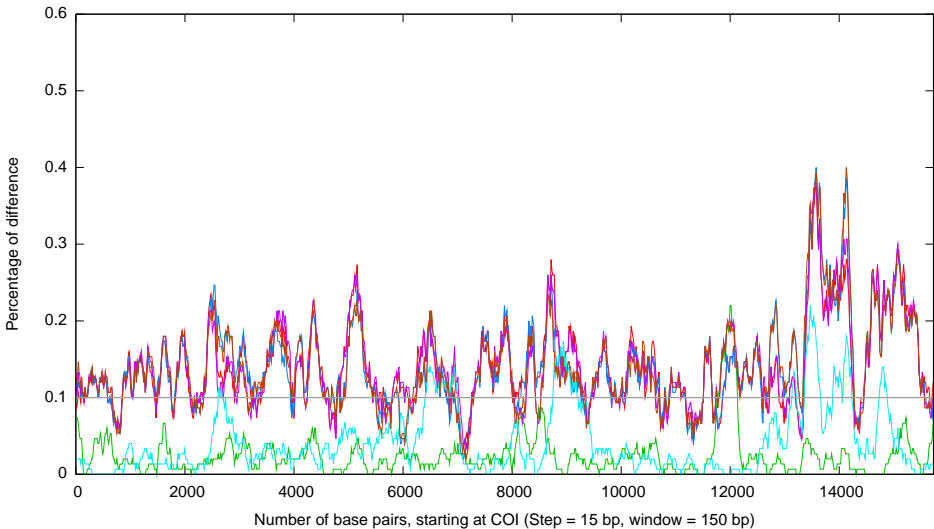

Supplement: File S2 — Sliding window analyses for Annelida, Cnidaria, Crustacea, Echinodermata, Mollusca, Nematoda, Nemertea, Platyhelminthes and Porifera. For each family, the folder contains the aligned sequences as well as the sliding window analyses by species pair and for all species pair on a single figure. (ZIP) [file pone.0051263.s002.zip › Crustacea/Acrididae/15_150/allCurves.pdf]

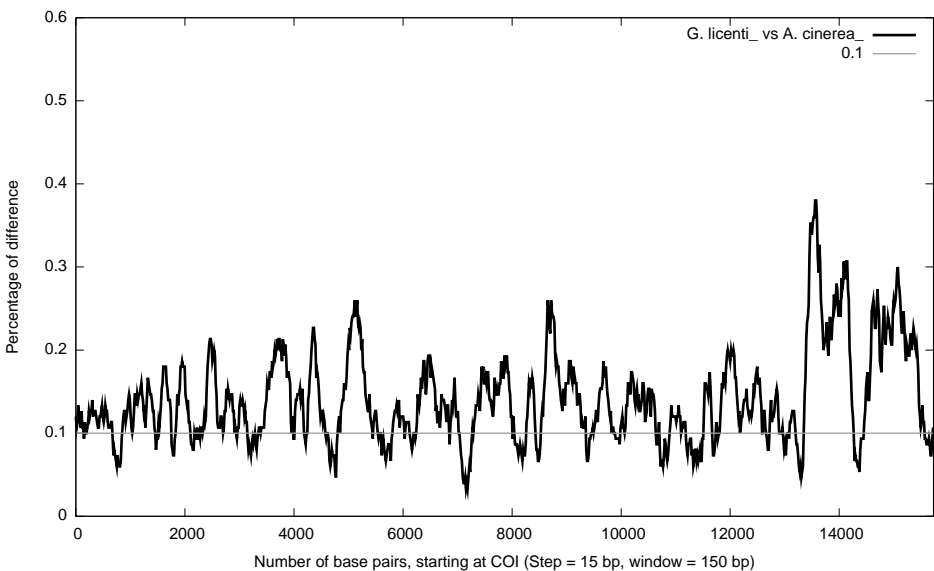

Supplement: File S2 — Sliding window analyses for Annelida, Cnidaria, Crustacea, Echinodermata, Mollusca, Nematoda, Nemertea, Platyhelminthes and Porifera. For each family, the folder contains the aligned sequences as well as the sliding window analyses by species pair and for all species pair on a single figure. (ZIP) [file pone.0051263.s002.zip › Crustacea/Acrididae/15_150/Gomphocerus_licenti_NC_013847_Acrida_cinerea_NC_014887.pdf]

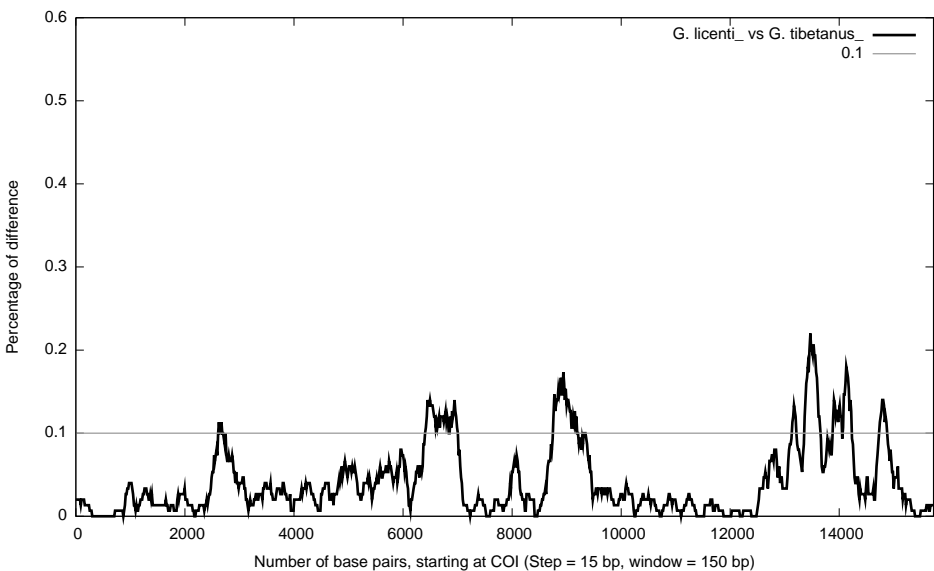

Supplement: File S2 — Sliding window analyses for Annelida, Cnidaria, Crustacea, Echinodermata, Mollusca, Nematoda, Nemertea, Platyhelminthes and Porifera. For each family, the folder contains the aligned sequences as well as the sliding window analyses by species pair and for all species pair on a single figure. (ZIP) [file pone.0051263.s002.zip › Crustacea/Acrididae/15_150/Gomphocerus_licenti_NC_013847_Gomphocerus_sibiricus_tibetanus_NC_015478.pdf]

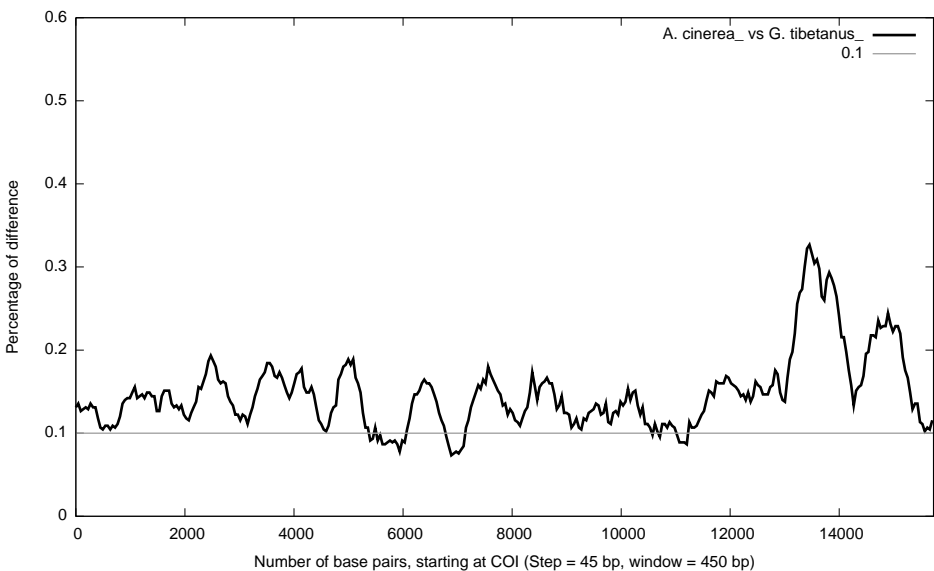

Supplement: File S2 — Sliding window analyses for Annelida, Cnidaria, Crustacea, Echinodermata, Mollusca, Nematoda, Nemertea, Platyhelminthes and Porifera. For each family, the folder contains the aligned sequences as well as the sliding window analyses by species pair and for all species pair on a single figure. (ZIP) [file pone.0051263.s002.zip › Crustacea/Acrididae/45_450/Acrida_cinerea_NC_014887_Gomphocerus_sibiricus_tibetanus_NC_015478.pdf]

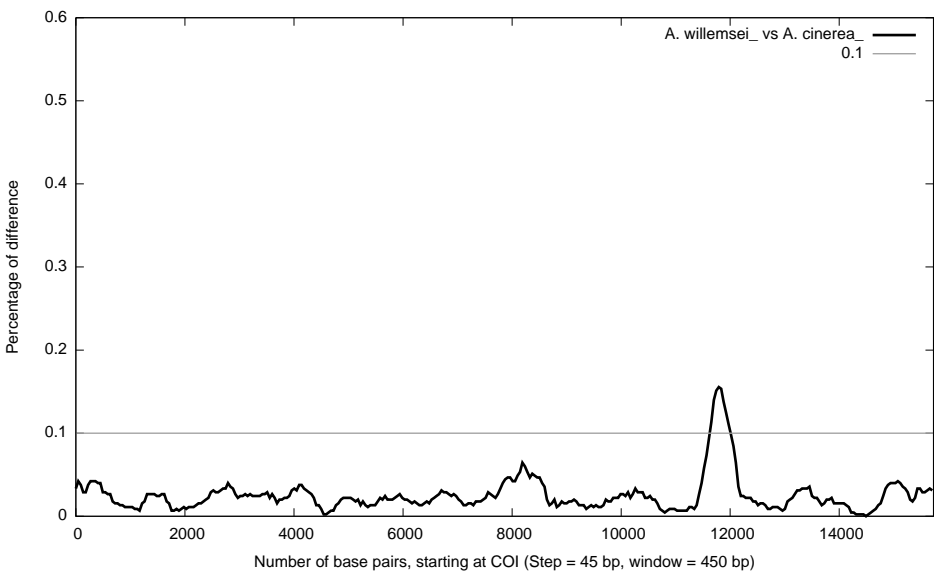

Supplement: File S2 — Sliding window analyses for Annelida, Cnidaria, Crustacea, Echinodermata, Mollusca, Nematoda, Nemertea, Platyhelminthes and Porifera. For each family, the folder contains the aligned sequences as well as the sliding window analyses by species pair and for all species pair on a single figure. (ZIP) [file pone.0051263.s002.zip › Crustacea/Acrididae/45_450/Acrida_willemsei_NC_011303_Acrida_cinerea_NC_014887.pdf]

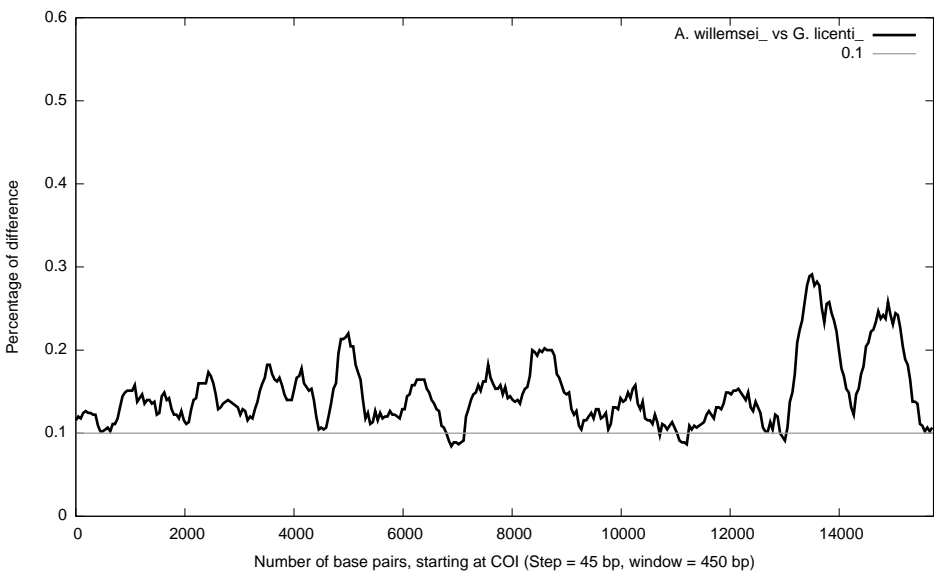

Supplement: File S2 — Sliding window analyses for Annelida, Cnidaria, Crustacea, Echinodermata, Mollusca, Nematoda, Nemertea, Platyhelminthes and Porifera. For each family, the folder contains the aligned sequences as well as the sliding window analyses by species pair and for all species pair on a single figure. (ZIP) [file pone.0051263.s002.zip › Crustacea/Acrididae/45_450/Acrida_willemsei_NC_011303_Gomphocerus_licenti_NC_013847.pdf]

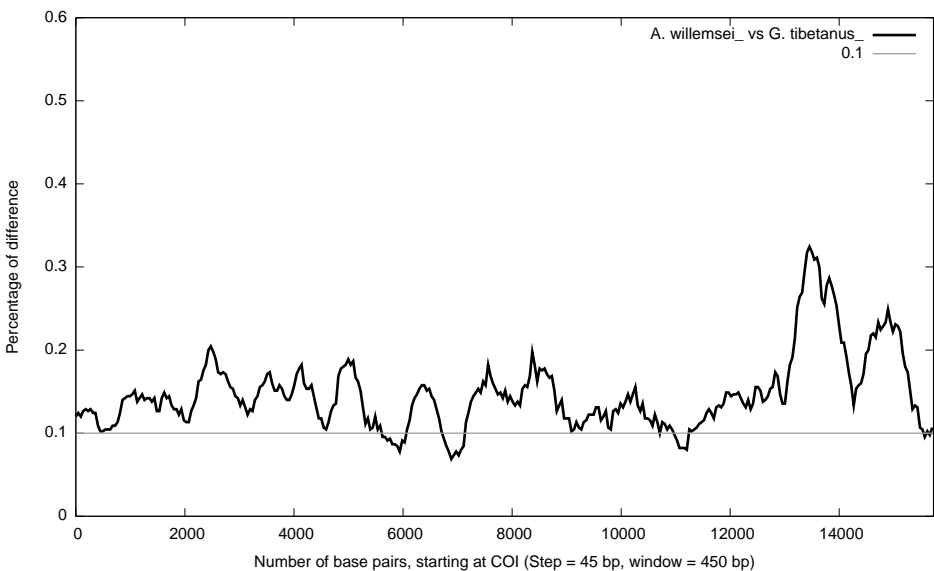

Supplement: File S2 — Sliding window analyses for Annelida, Cnidaria, Crustacea, Echinodermata, Mollusca, Nematoda, Nemertea, Platyhelminthes and Porifera. For each family, the folder contains the aligned sequences as well as the sliding window analyses by species pair and for all species pair on a single figure. (ZIP) [file pone.0051263.s002.zip › Crustacea/Acrididae/45_450/Acrida_willemsei_NC_011303_Gomphocerus_sibiricus_tibetanus_NC_015478.pdf]

Acrididae

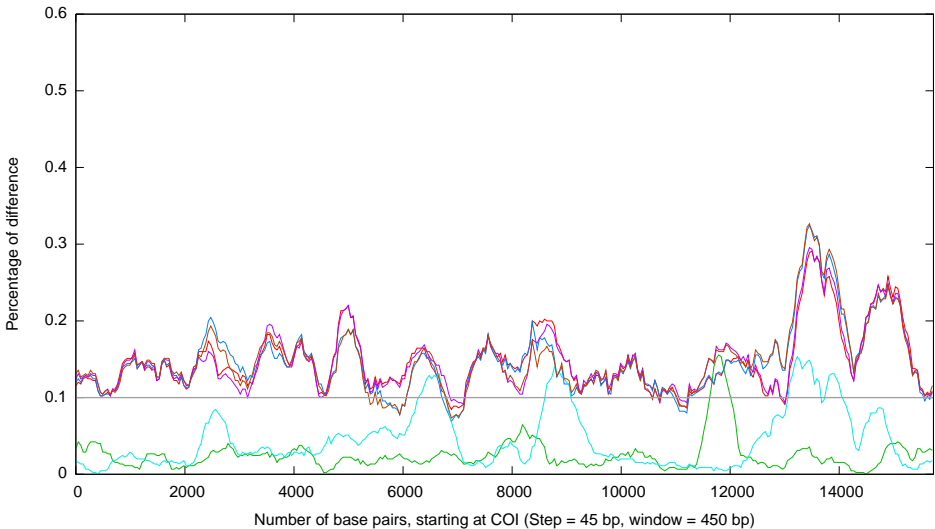

Supplement: File S2 — Sliding window analyses for Annelida, Cnidaria, Crustacea, Echinodermata, Mollusca, Nematoda, Nemertea, Platyhelminthes and Porifera. For each family, the folder contains the aligned sequences as well as the sliding window analyses by species pair and for all species pair on a single figure. (ZIP) [file pone.0051263.s002.zip › Crustacea/Acrididae/45_450/allCurves.pdf]

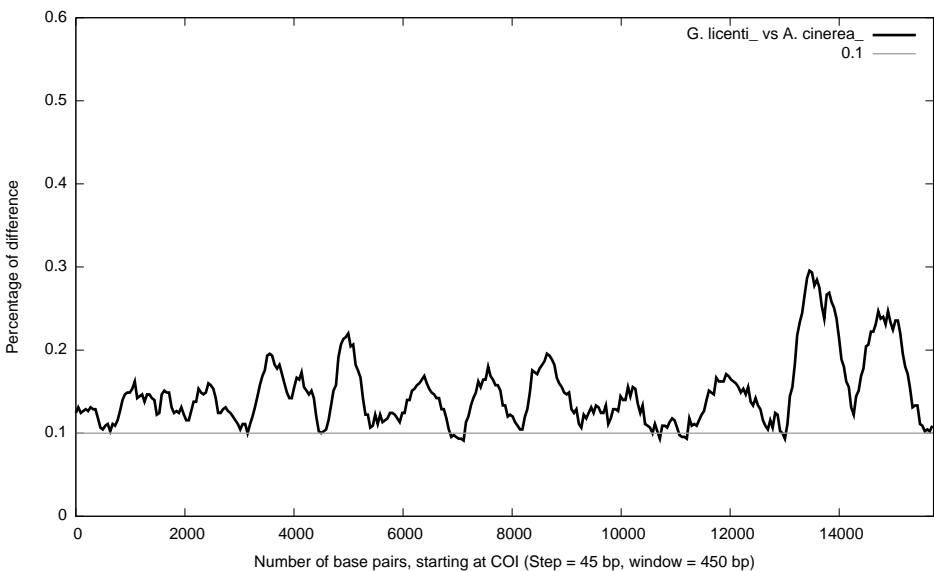

Supplement: File S2 — Sliding window analyses for Annelida, Cnidaria, Crustacea, Echinodermata, Mollusca, Nematoda, Nemertea, Platyhelminthes and Porifera. For each family, the folder contains the aligned sequences as well as the sliding window analyses by species pair and for all species pair on a single figure. (ZIP) [file pone.0051263.s002.zip › Crustacea/Acrididae/45_450/Gomphocerus_licenti_NC_013847_Acrida_cinerea_NC_014887.pdf]

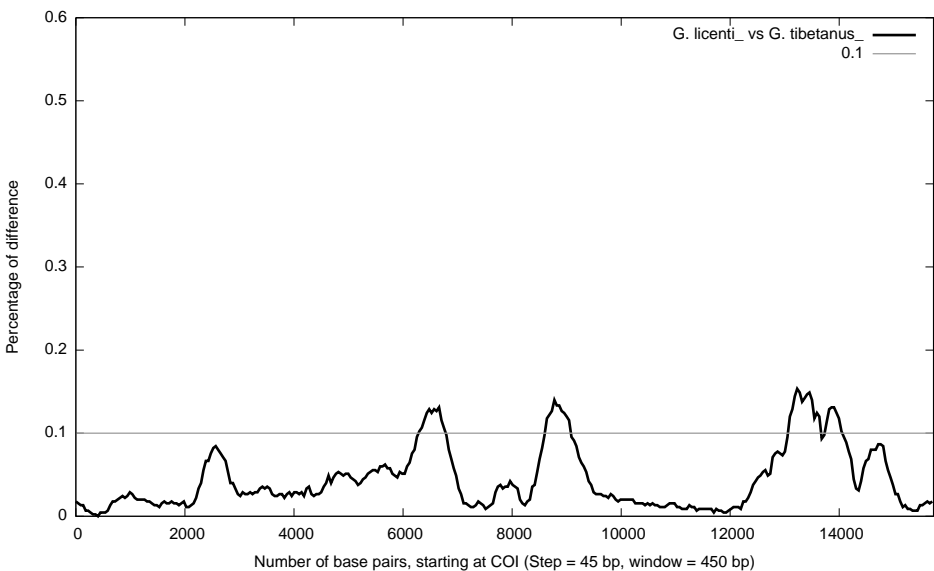

Supplement: File S2 — Sliding window analyses for Annelida, Cnidaria, Crustacea, Echinodermata, Mollusca, Nematoda, Nemertea, Platyhelminthes and Porifera. For each family, the folder contains the aligned sequences as well as the sliding window analyses by species pair and for all species pair on a single figure. (ZIP) [file pone.0051263.s002.zip › Crustacea/Acrididae/45_450/Gomphocerus_licenti_NC_013847_Gomphocerus_sibiricus_tibetanus_NC_015478.pdf]

# Agromyzidae

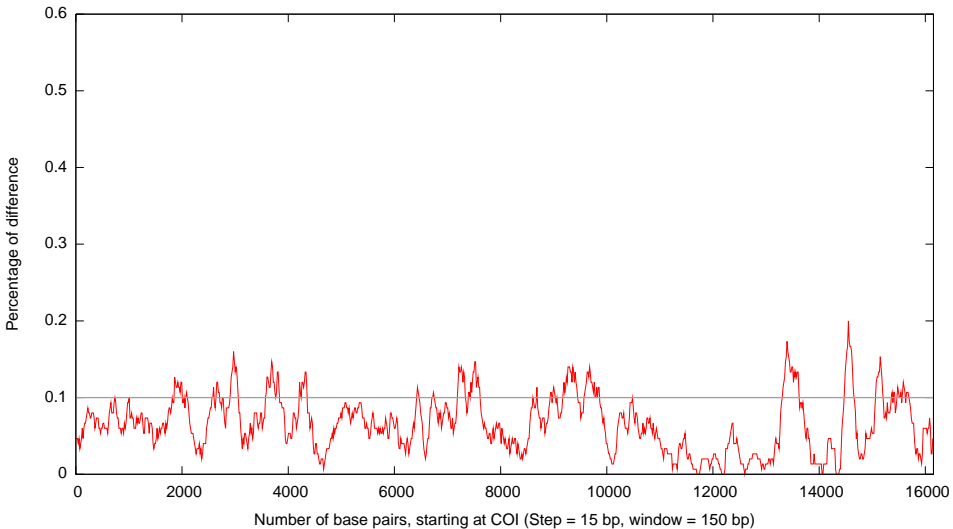

Supplement: File S2 — Sliding window analyses for Annelida, Cnidaria, Crustacea, Echinodermata, Mollusca, Nematoda, Nemertea, Platyhelminthes and Porifera. For each family, the folder contains the aligned sequences as well as the sliding window analyses by species pair and for all species pair on a single figure. (ZIP) [file pone.0051263.s002.zip › Crustacea/Agromyzidae/15_150/allCurves.pdf]

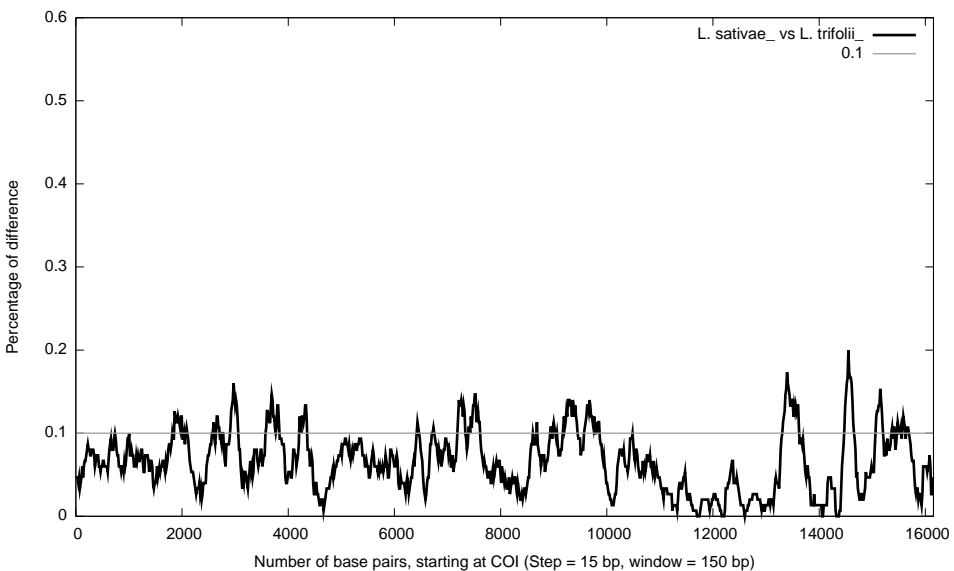

Supplement: File S2 — Sliding window analyses for Annelida, Cnidaria, Crustacea, Echinodermata, Mollusca, Nematoda, Nemertea, Platyhelminthes and Porifera. For each family, the folder contains the aligned sequences as well as the sliding window analyses by species pair and for all species pair on a single figure. (ZIP) [file pone.0051263.s002.zip › Crustacea/Agromyzidae/15_150/Liriomyza_sativae_NC_015926_Liriomyza_trifolii_NC_014283.pdf]

# Agromyzidae

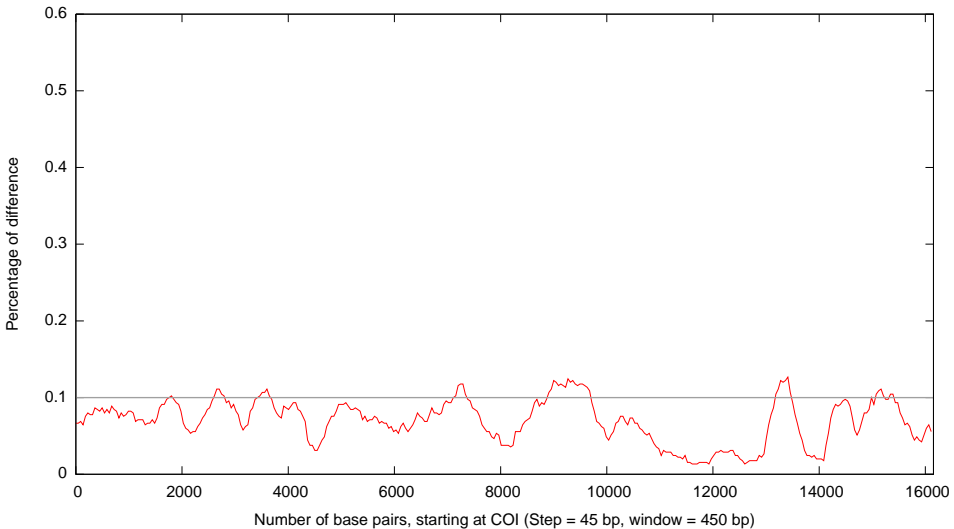

Supplement: File S2 — Sliding window analyses for Annelida, Cnidaria, Crustacea, Echinodermata, Mollusca, Nematoda, Nemertea, Platyhelminthes and Porifera. For each family, the folder contains the aligned sequences as well as the sliding window analyses by species pair and for all species pair on a single figure. (ZIP) [file pone.0051263.s002.zip › Crustacea/Agromyzidae/45_450/allCurves.pdf]

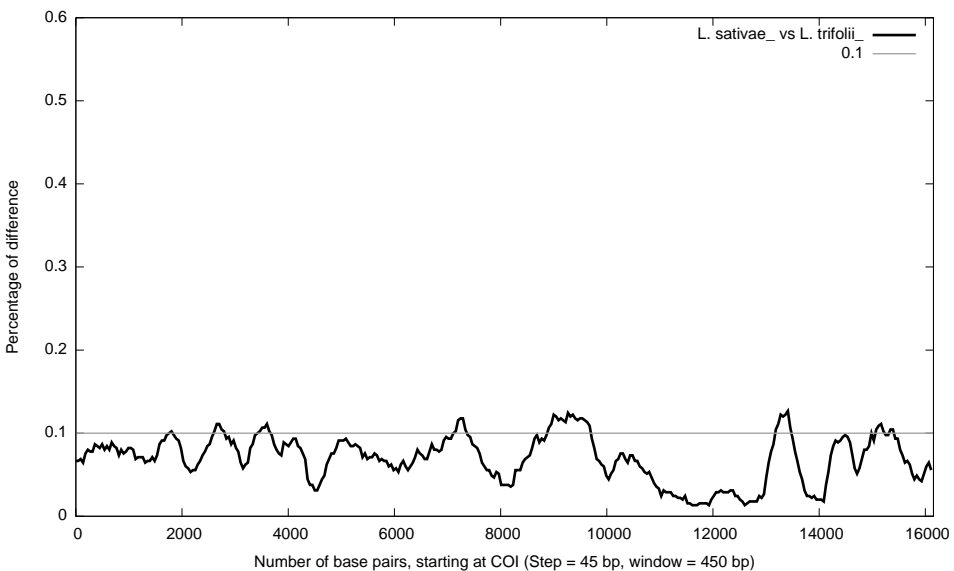

Supplement: File S2 — Sliding window analyses for Annelida, Cnidaria, Crustacea, Echinodermata, Mollusca, Nematoda, Nemertea, Platyhelminthes and Porifera. For each family, the folder contains the aligned sequences as well as the sliding window analyses by species pair and for all species pair on a single figure. (ZIP) [file pone.0051263.s002.zip › Crustacea/Agromyzidae/45_450/Liriomyza_sativae_NC_015926_Liriomyza_trifolii_NC_014283.pdf]

# Apidae

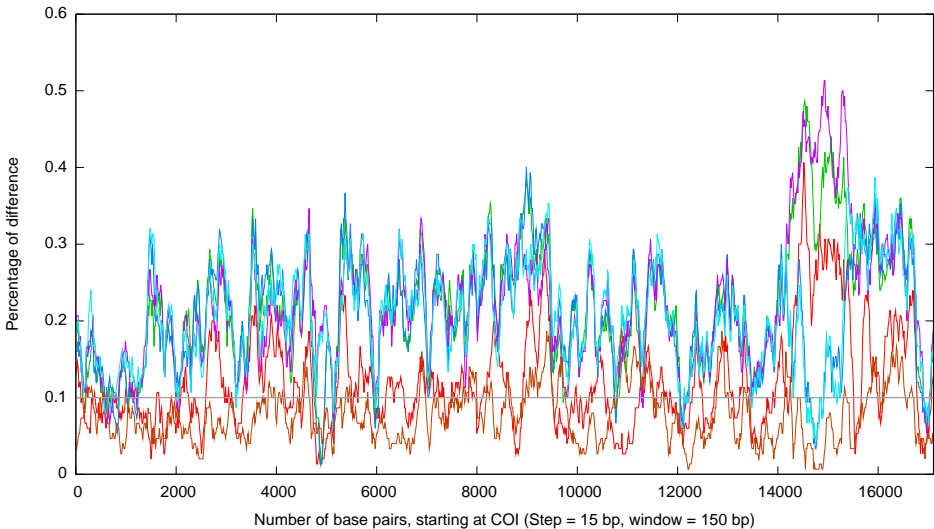

Supplement: File S2 — Sliding window analyses for Annelida, Cnidaria, Crustacea, Echinodermata, Mollusca, Nematoda, Nemertea, Platyhelminthes and Porifera. For each family, the folder contains the aligned sequences as well as the sliding window analyses by species pair and for all species pair on a single figure. (ZIP) [file pone.0051263.s002.zip › Crustacea/Apidae/15_150/allCurves.pdf]

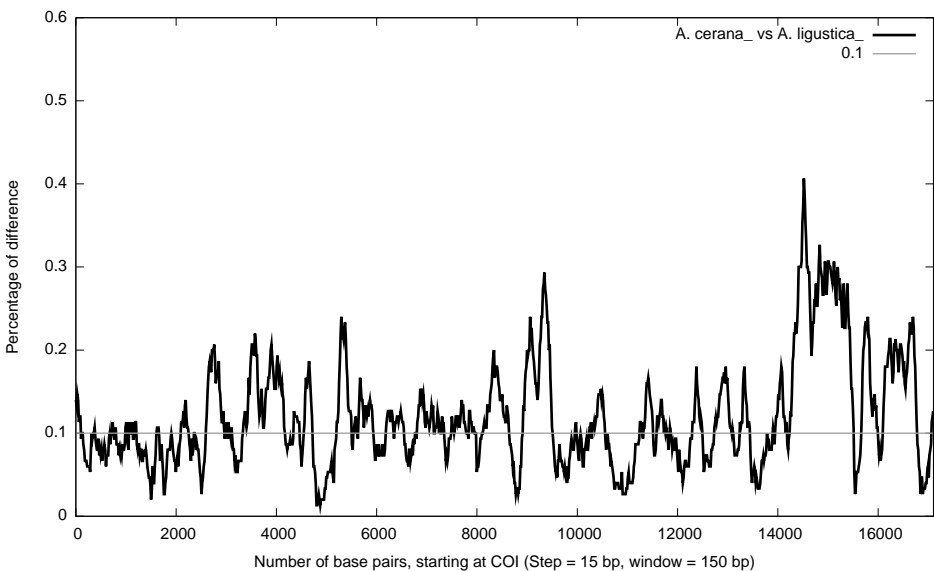

Supplement: File S2 — Sliding window analyses for Annelida, Cnidaria, Crustacea, Echinodermata, Mollusca, Nematoda, Nemertea, Platyhelminthes and Porifera. For each family, the folder contains the aligned sequences as well as the sliding window analyses by species pair and for all species pair on a single figure. (ZIP) [file pone.0051263.s002.zip › Crustacea/Apidae/15_150/Apis_cerana_NC_014295_Apis_mellifera_ligustica_NC_001566.pdf]

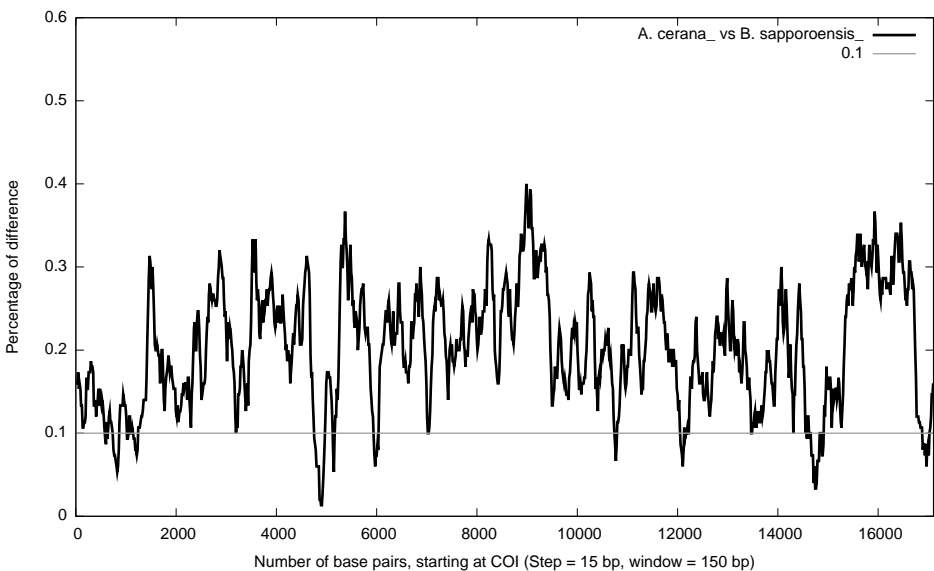

Supplement: File S2 — Sliding window analyses for Annelida, Cnidaria, Crustacea, Echinodermata, Mollusca, Nematoda, Nemertea, Platyhelminthes and Porifera. For each family, the folder contains the aligned sequences as well as the sliding window analyses by species pair and for all species pair on a single figure. (ZIP) [file pone.0051263.s002.zip › Crustacea/Apidae/15_150/Apis_cerana_NC_014295_Bombus_hypocrita_sapporoensis_NC_011923.pdf]

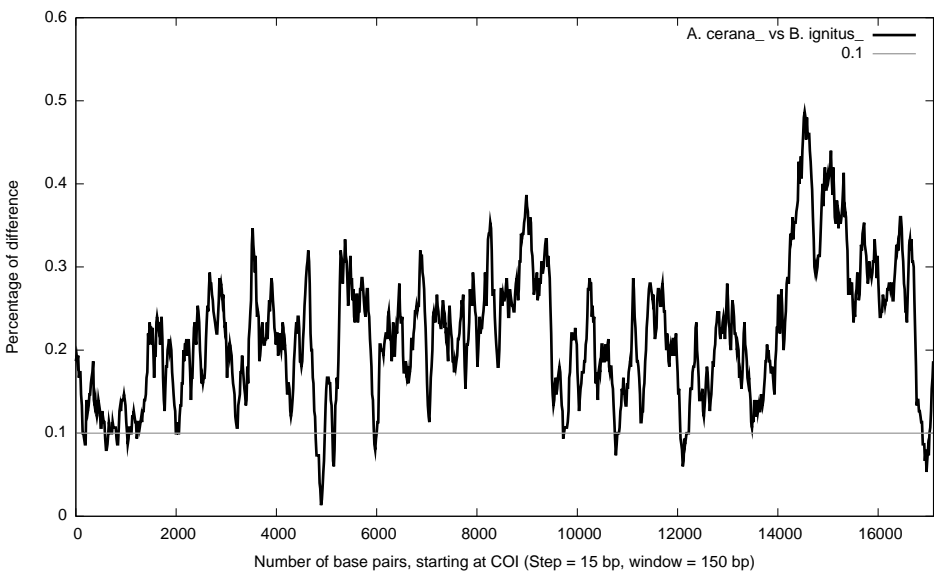

Supplement: File S2 — Sliding window analyses for Annelida, Cnidaria, Crustacea, Echinodermata, Mollusca, Nematoda, Nemertea, Platyhelminthes and Porifera. For each family, the folder contains the aligned sequences as well as the sliding window analyses by species pair and for all species pair on a single figure. (ZIP) [file pone.0051263.s002.zip › Crustacea/Apidae/15_150/Apis_cerana_NC_014295_Bombus_ignitus_NC_010967.pdf]

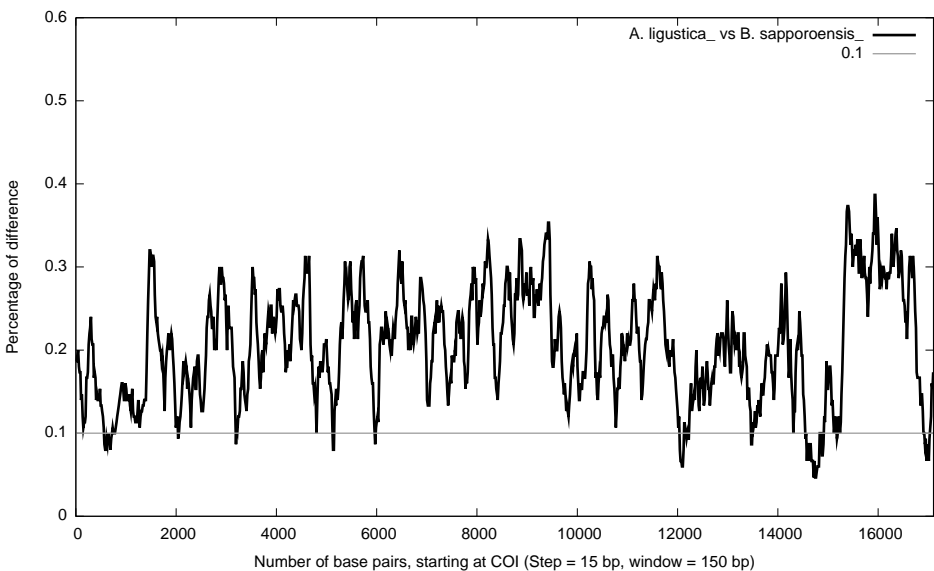

Supplement: File S2 — Sliding window analyses for Annelida, Cnidaria, Crustacea, Echinodermata, Mollusca, Nematoda, Nemertea, Platyhelminthes and Porifera. For each family, the folder contains the aligned sequences as well as the sliding window analyses by species pair and for all species pair on a single figure. (ZIP) [file pone.0051263.s002.zip › Crustacea/Apidae/15_150/Apis_mellifera_ligustica_NC_001566_Bombus_hypocrita_sapporoensis_NC_011923.pdf]

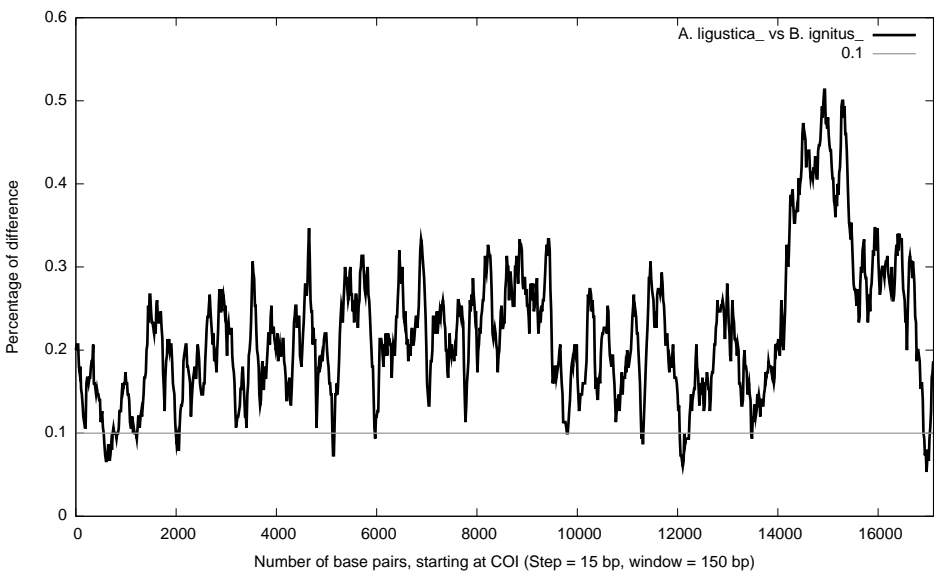

Supplement: File S2 — Sliding window analyses for Annelida, Cnidaria, Crustacea, Echinodermata, Mollusca, Nematoda, Nemertea, Platyhelminthes and Porifera. For each family, the folder contains the aligned sequences as well as the sliding window analyses by species pair and for all species pair on a single figure. (ZIP) [file pone.0051263.s002.zip › Crustacea/Apidae/15_150/Apis_mellifera_ligustica_NC_001566_Bombus_ignitus_NC_010967.pdf]

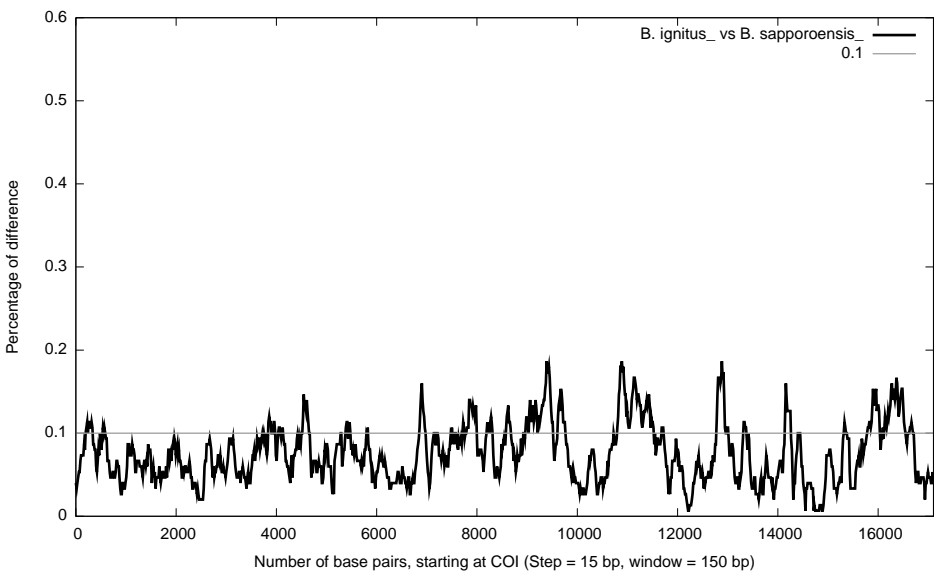

Supplement: File S2 — Sliding window analyses for Annelida, Cnidaria, Crustacea, Echinodermata, Mollusca, Nematoda, Nemertea, Platyhelminthes and Porifera. For each family, the folder contains the aligned sequences as well as the sliding window analyses by species pair and for all species pair on a single figure. (ZIP) [file pone.0051263.s002.zip › Crustacea/Apidae/15_150/Bombus_ignitus_NC_010967_Bombus_hypocrita_sapporoensis_NC_011923.pdf]

# Apidae

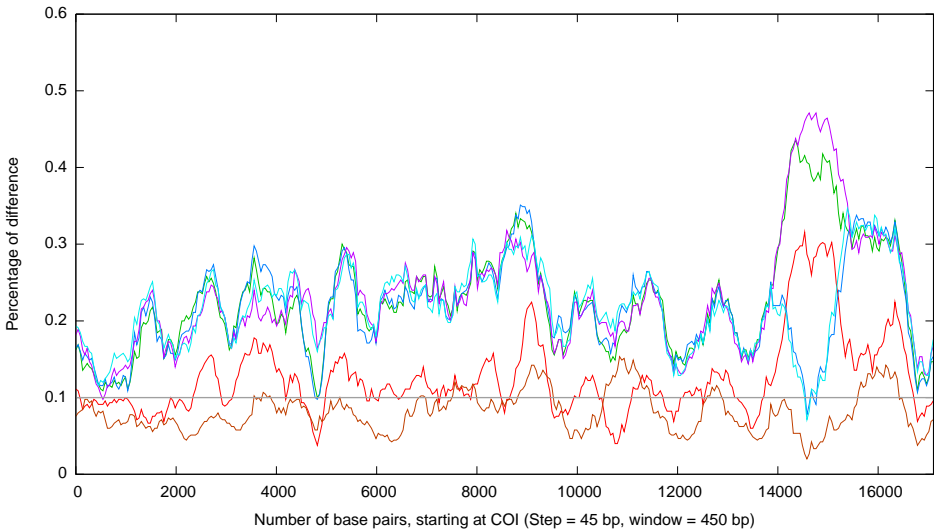

Supplement: File S2 — Sliding window analyses for Annelida, Cnidaria, Crustacea, Echinodermata, Mollusca, Nematoda, Nemertea, Platyhelminthes and Porifera. For each family, the folder contains the aligned sequences as well as the sliding window analyses by species pair and for all species pair on a single figure. (ZIP) [file pone.0051263.s002.zip › Crustacea/Apidae/45_450/allCurves.pdf]

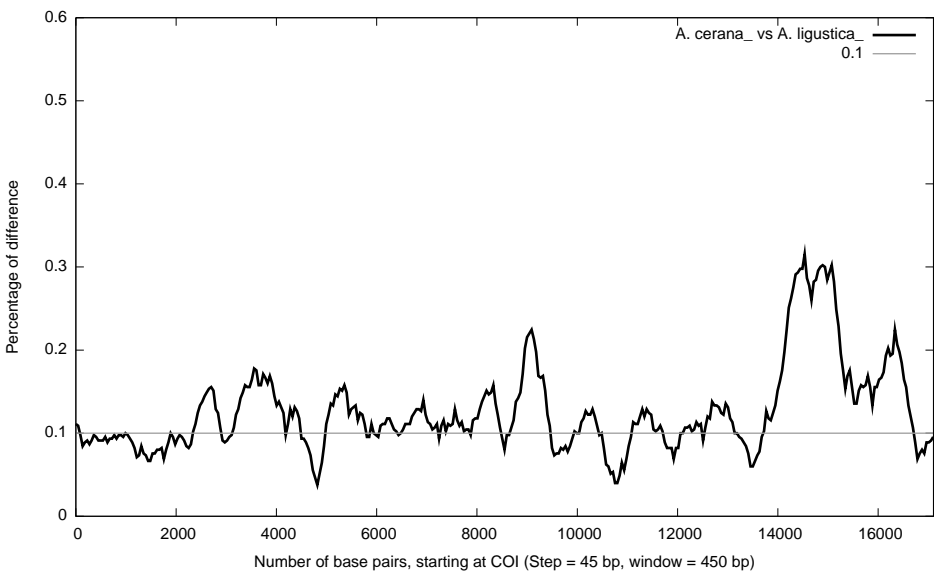

Supplement: File S2 — Sliding window analyses for Annelida, Cnidaria, Crustacea, Echinodermata, Mollusca, Nematoda, Nemertea, Platyhelminthes and Porifera. For each family, the folder contains the aligned sequences as well as the sliding window analyses by species pair and for all species pair on a single figure. (ZIP) [file pone.0051263.s002.zip › Crustacea/Apidae/45_450/Apis_cerana_NC_014295_Apis_mellifera_ligustica_NC_001566.pdf]

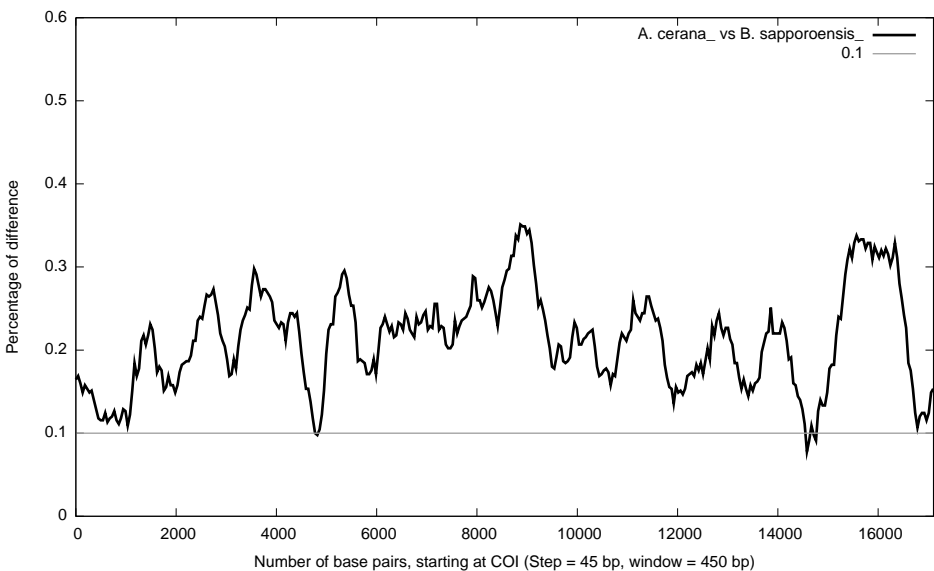

Supplement: File S2 — Sliding window analyses for Annelida, Cnidaria, Crustacea, Echinodermata, Mollusca, Nematoda, Nemertea, Platyhelminthes and Porifera. For each family, the folder contains the aligned sequences as well as the sliding window analyses by species pair and for all species pair on a single figure. (ZIP) [file pone.0051263.s002.zip › Crustacea/Apidae/45_450/Apis_cerana_NC_014295_Bombus_hypocrita_sapporoensis_NC_011923.pdf]

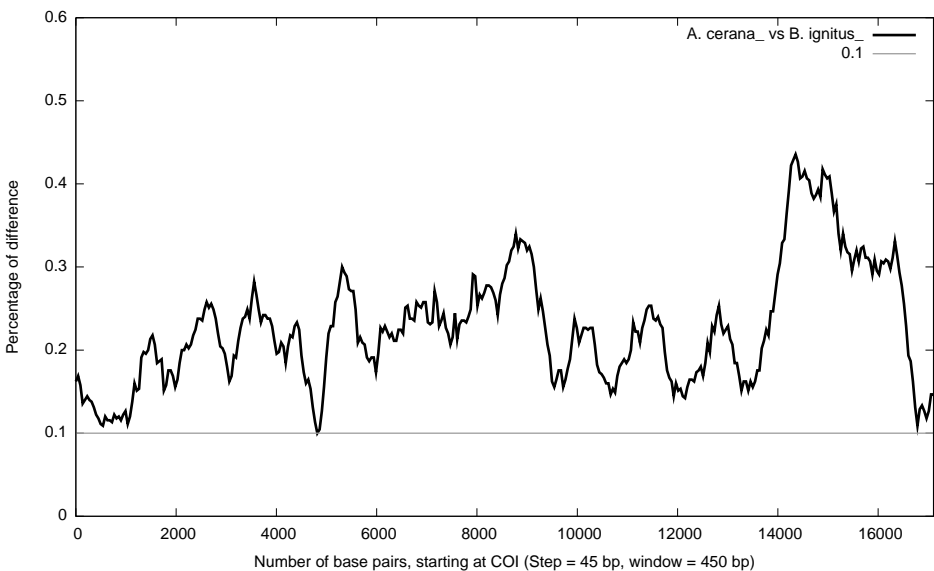

Supplement: File S2 — Sliding window analyses for Annelida, Cnidaria, Crustacea, Echinodermata, Mollusca, Nematoda, Nemertea, Platyhelminthes and Porifera. For each family, the folder contains the aligned sequences as well as the sliding window analyses by species pair and for all species pair on a single figure. (ZIP) [file pone.0051263.s002.zip › Crustacea/Apidae/45_450/Apis_cerana_NC_014295_Bombus_ignitus_NC_010967.pdf]

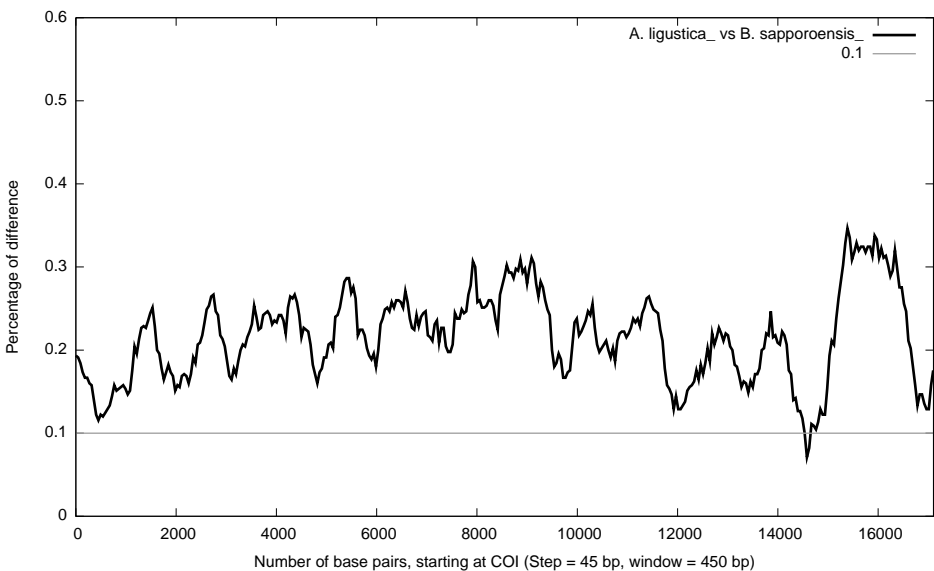

Supplement: File S2 — Sliding window analyses for Annelida, Cnidaria, Crustacea, Echinodermata, Mollusca, Nematoda, Nemertea, Platyhelminthes and Porifera. For each family, the folder contains the aligned sequences as well as the sliding window analyses by species pair and for all species pair on a single figure. (ZIP) [file pone.0051263.s002.zip › Crustacea/Apidae/45_450/Apis_mellifera_ligustica_NC_001566_Bombus_hypocrita_sapporoensis_NC_011923.pdf]

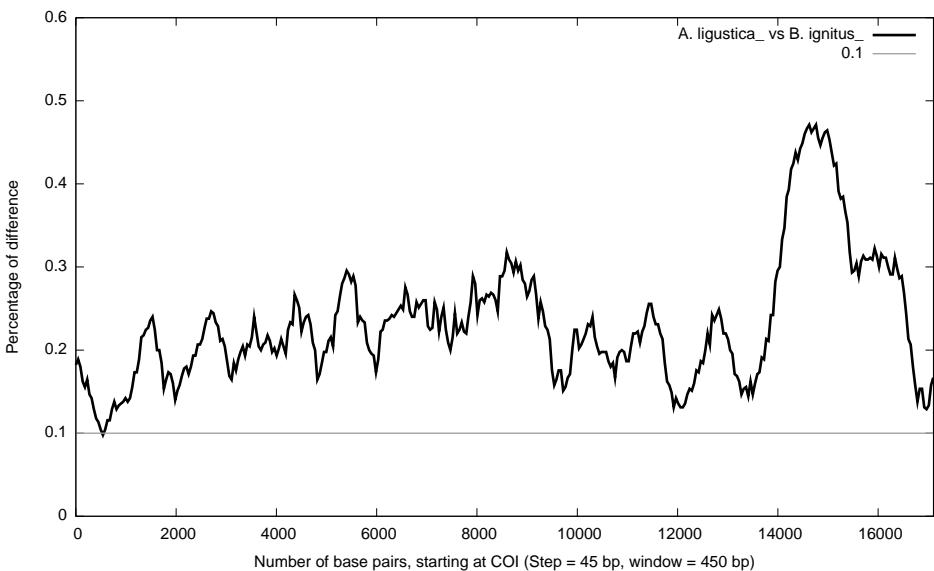

Supplement: File S2 — Sliding window analyses for Annelida, Cnidaria, Crustacea, Echinodermata, Mollusca, Nematoda, Nemertea, Platyhelminthes and Porifera. For each family, the folder contains the aligned sequences as well as the sliding window analyses by species pair and for all species pair on a single figure. (ZIP) [file pone.0051263.s002.zip › Crustacea/Apidae/45_450/Apis_mellifera_ligustica_NC_001566_Bombus_ignitus_NC_010967.pdf]

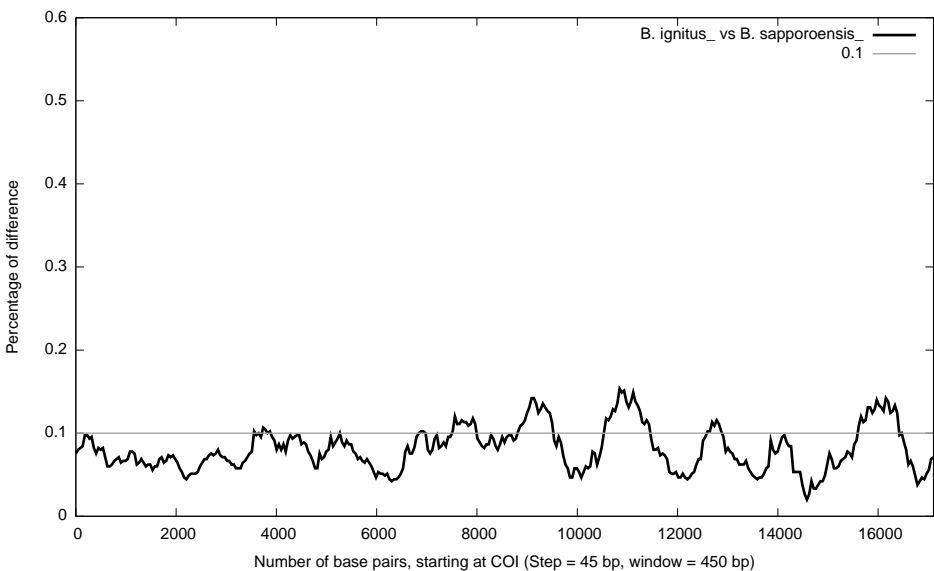

Supplement: File S2 — Sliding window analyses for Annelida, Cnidaria, Crustacea, Echinodermata, Mollusca, Nematoda, Nemertea, Platyhelminthes and Porifera. For each family, the folder contains the aligned sequences as well as the sliding window analyses by species pair and for all species pair on a single figure. (ZIP) [file pone.0051263.s002.zip › Crustacea/Apidae/45_450/Bombus_ignitus_NC_010967_Bombus_hypocrita_sapporoensis_NC_011923.pdf]

# Argasidae

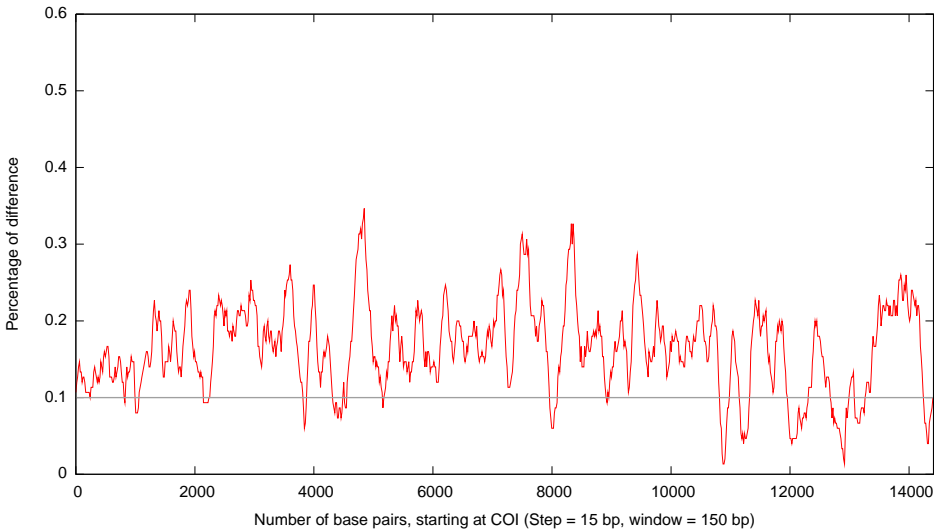

Supplement: File S2 — Sliding window analyses for Annelida, Cnidaria, Crustacea, Echinodermata, Mollusca, Nematoda, Nemertea, Platyhelminthes and Porifera. For each family, the folder contains the aligned sequences as well as the sliding window analyses by species pair and for all species pair on a single figure. (ZIP) [file pone.0051263.s002.zip › Crustacea/Argasidae/15_150/allCurves.pdf]

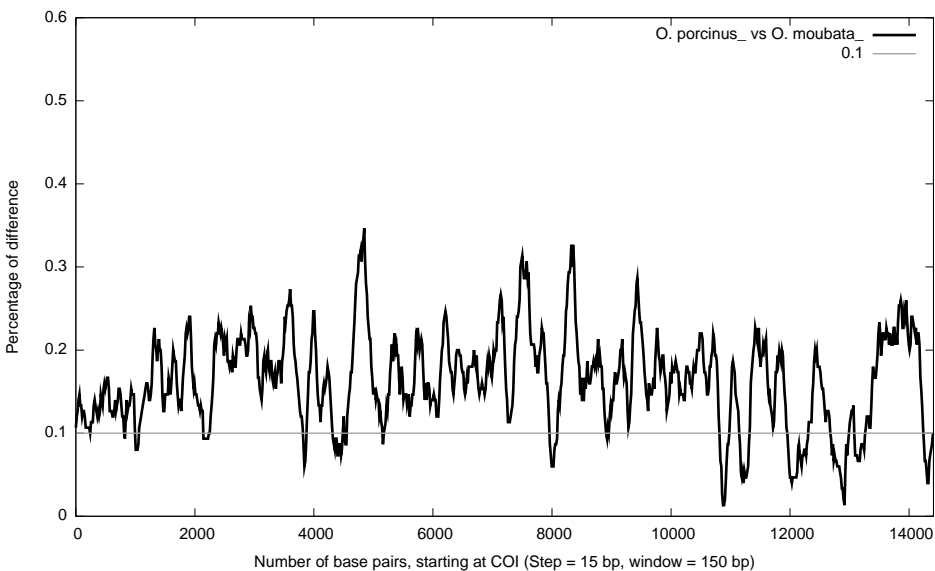

Supplement: File S2 — Sliding window analyses for Annelida, Cnidaria, Crustacea, Echinodermata, Mollusca, Nematoda, Nemertea, Platyhelminthes and Porifera. For each family, the folder contains the aligned sequences as well as the sliding window analyses by species pair and for all species pair on a single figure. (ZIP) [file pone.0051263.s002.zip › Crustacea/Argasidae/15_150/Ornithodoros_porcinus_NC_005820_Ornithodoros_moubata_NC_004357.pdf]

# Ambystomatidae

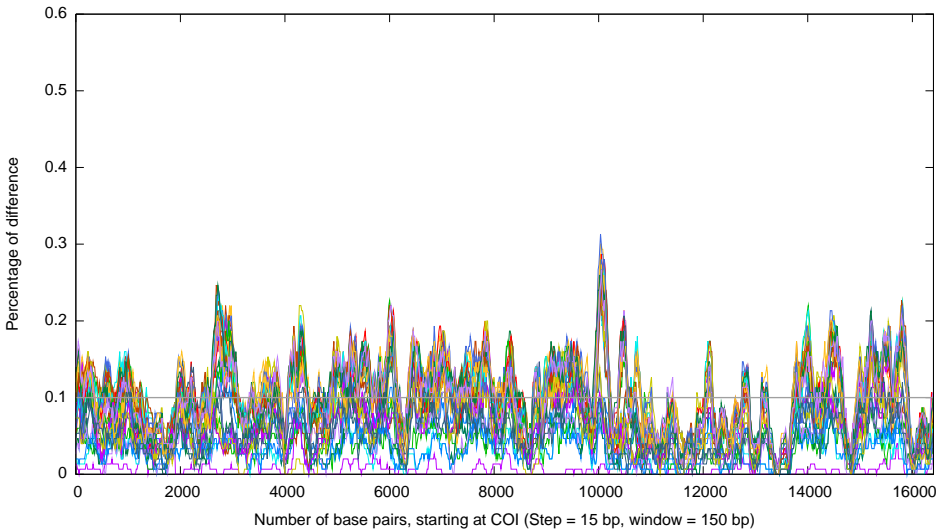

Supplement: File S3 — Sliding window analyses for Mammalia and Lissamphibia. For each family, the folder contains the aligned sequences as well as the sliding window analyses by species pair and for all species pair on a single figure. (ZIP) [file pone.0051263.s003.zip › Lissamphibia/Ambystomatidae/15_150/allCurves.pdf]

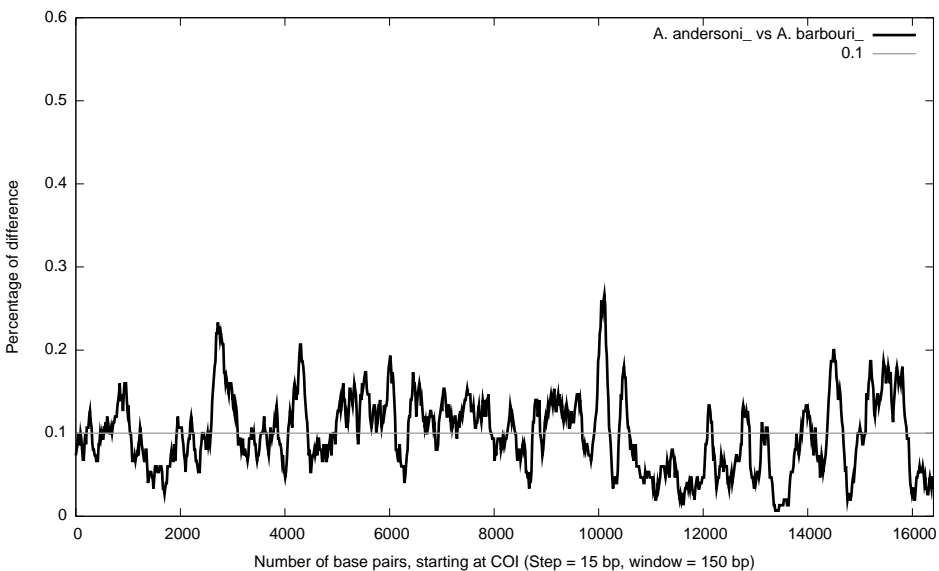

Supplement: File S3 — Sliding window analyses for Mammalia and Lissamphibia. For each family, the folder contains the aligned sequences as well as the sliding window analyses by species pair and for all species pair on a single figure. (ZIP) [file pone.0051263.s003.zip › Lissamphibia/Ambystomatidae/15_150/Ambystoma_andersoni_NC_006888_Ambystoma_barbouri_NC_014568.pdf]

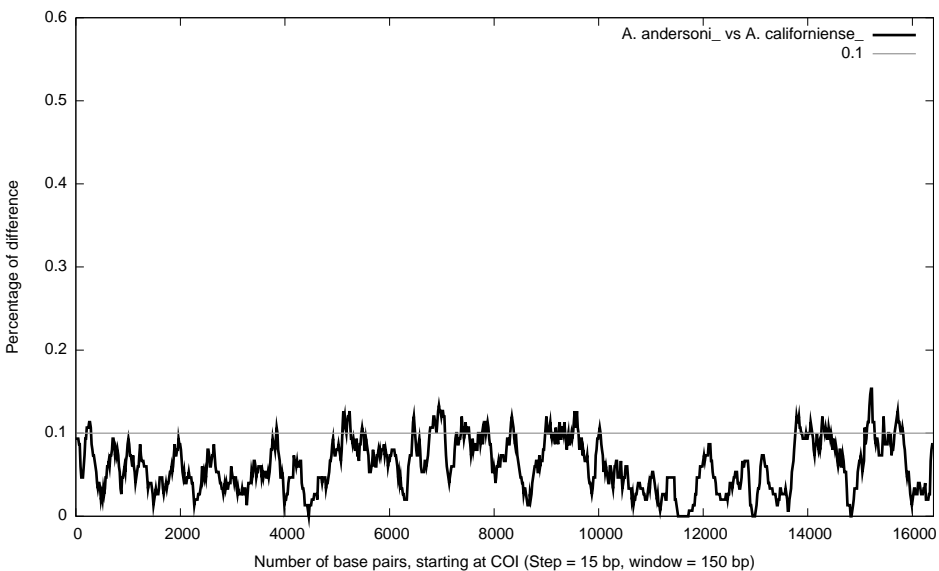

Supplement: File S3 — Sliding window analyses for Mammalia and Lissamphibia. For each family, the folder contains the aligned sequences as well as the sliding window analyses by species pair and for all species pair on a single figure. (ZIP) [file pone.0051263.s003.zip › Lissamphibia/Ambystomatidae/15_150/Ambystoma_andersoni_NC_006888_Ambystoma_californiense_NC_006890.pdf]

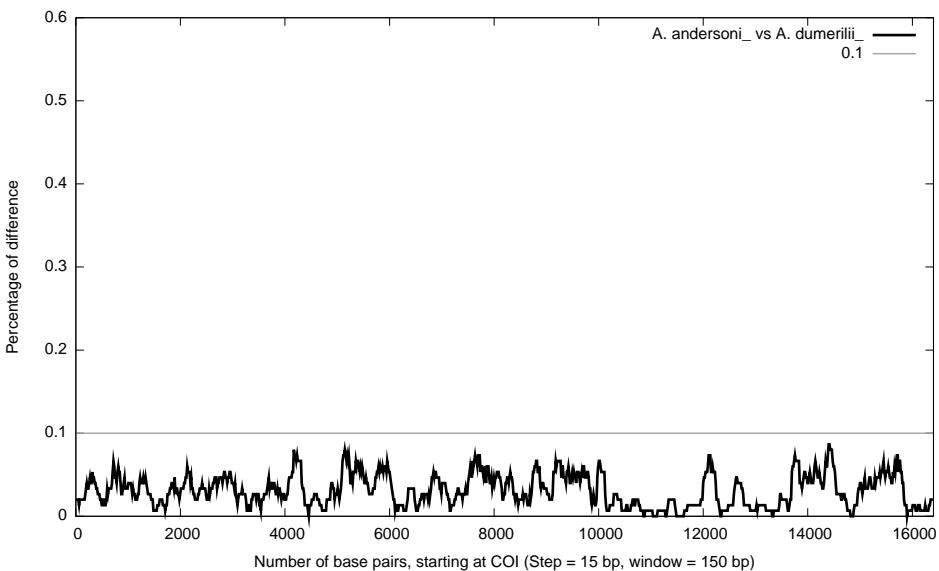

Supplement: File S3 — Sliding window analyses for Mammalia and Lissamphibia. For each family, the folder contains the aligned sequences as well as the sliding window analyses by species pair and for all species pair on a single figure. (ZIP) [file pone.0051263.s003.zip › Lissamphibia/Ambystomatidae/15_150/Ambystoma_andersoni_NC_006888_Ambystoma_dumerilii_NC_006889.pdf]

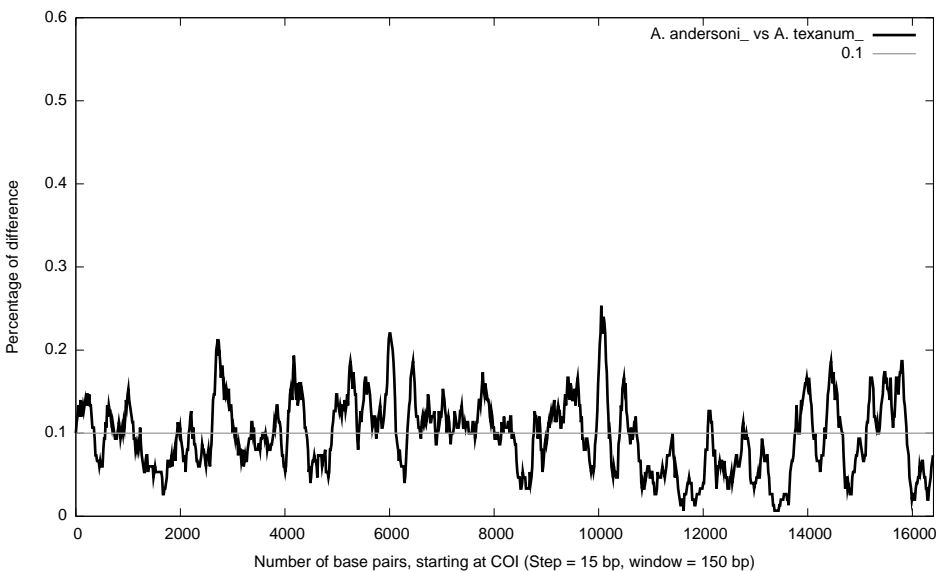

Supplement: File S3 — Sliding window analyses for Mammalia and Lissamphibia. For each family, the folder contains the aligned sequences as well as the sliding window analyses by species pair and for all species pair on a single figure. (ZIP) [file pone.0051263.s003.zip › Lissamphibia/Ambystomatidae/15_150/Ambystoma_andersoni_NC_006888_Ambystoma_texanum_NC_014571.pdf]

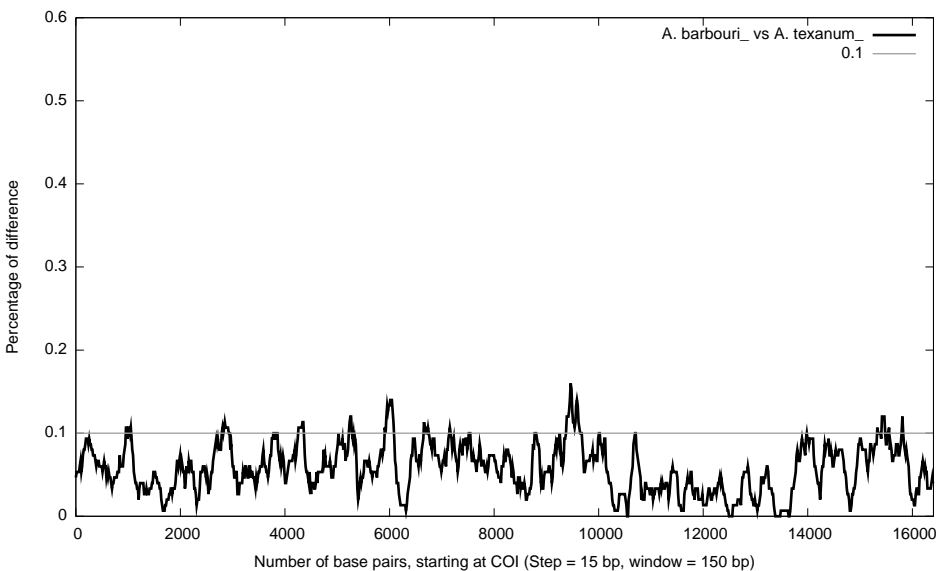

Supplement: File S3 — Sliding window analyses for Mammalia and Lissamphibia. For each family, the folder contains the aligned sequences as well as the sliding window analyses by species pair and for all species pair on a single figure. (ZIP) [file pone.0051263.s003.zip › Lissamphibia/Ambystomatidae/15_150/Ambystoma_barbouri_NC_014568_Ambystoma_texanum_NC_014571.pdf]

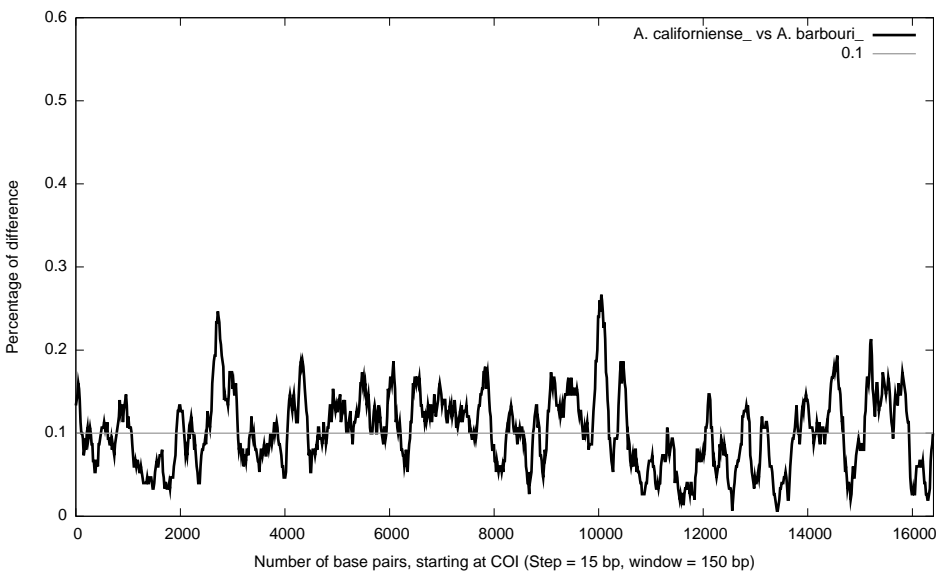

Supplement: File S3 — Sliding window analyses for Mammalia and Lissamphibia. For each family, the folder contains the aligned sequences as well as the sliding window analyses by species pair and for all species pair on a single figure. (ZIP) [file pone.0051263.s003.zip › Lissamphibia/Ambystomatidae/15_150/Ambystoma_californiense_NC_006890_Ambystoma_barbouri_NC_014568.pdf]

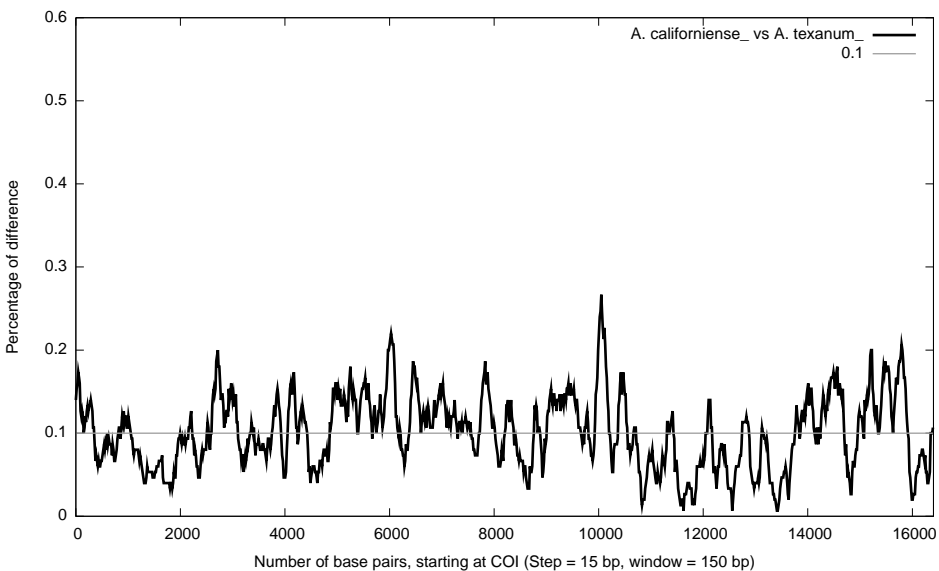

Supplement: File S3 — Sliding window analyses for Mammalia and Lissamphibia. For each family, the folder contains the aligned sequences as well as the sliding window analyses by species pair and for all species pair on a single figure. (ZIP) [file pone.0051263.s003.zip › Lissamphibia/Ambystomatidae/15_150/Ambystoma_californiense_NC_006890_Ambystoma_texanum_NC_014571.pdf]

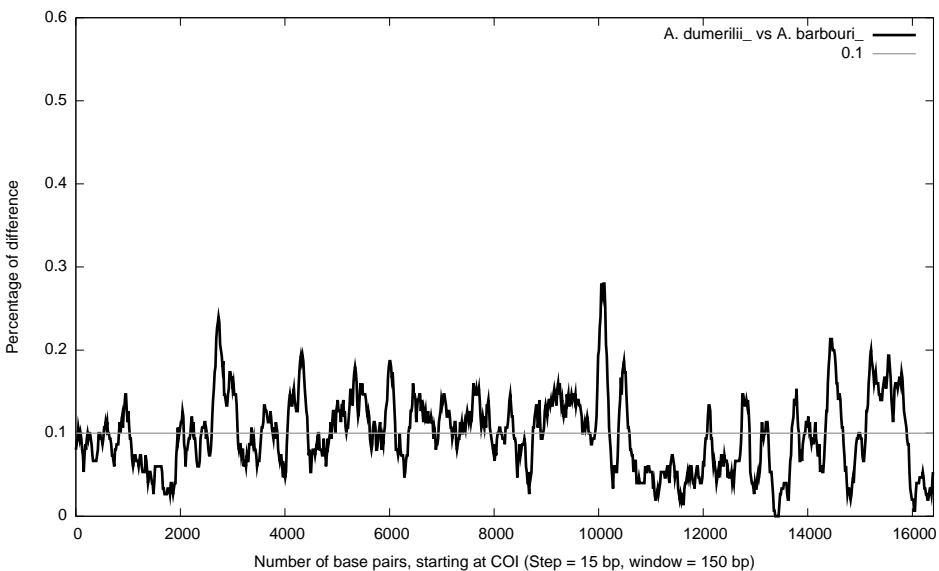

Supplement: File S3 — Sliding window analyses for Mammalia and Lissamphibia. For each family, the folder contains the aligned sequences as well as the sliding window analyses by species pair and for all species pair on a single figure. (ZIP) [file pone.0051263.s003.zip › Lissamphibia/Ambystomatidae/15_150/Ambystoma_dumerilii_NC_006889_Ambystoma_barbouri_NC_014568.pdf]

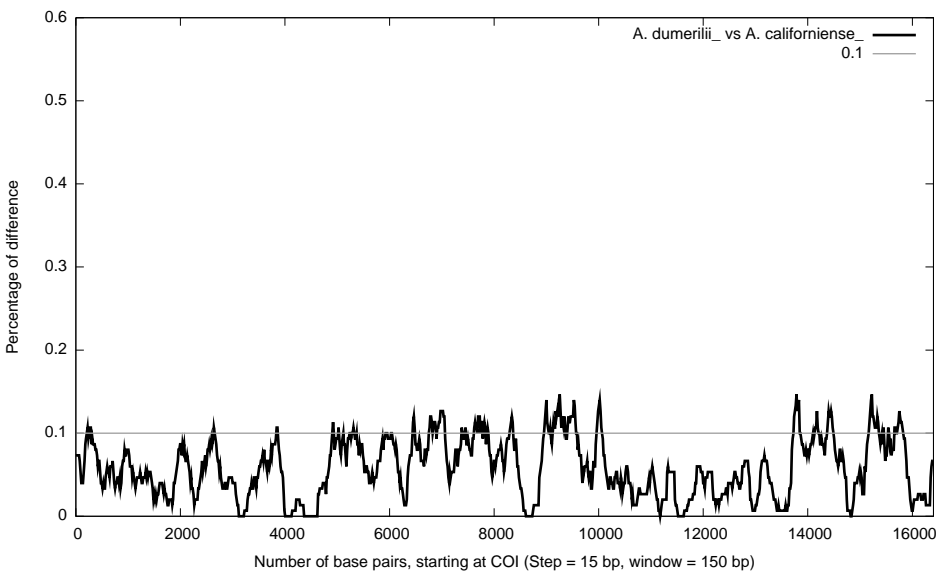

Supplement: File S3 — Sliding window analyses for Mammalia and Lissamphibia. For each family, the folder contains the aligned sequences as well as the sliding window analyses by species pair and for all species pair on a single figure. (ZIP) [file pone.0051263.s003.zip › Lissamphibia/Ambystomatidae/15_150/Ambystoma_dumerilii_NC_006889_Ambystoma_californiense_NC_006890.pdf]

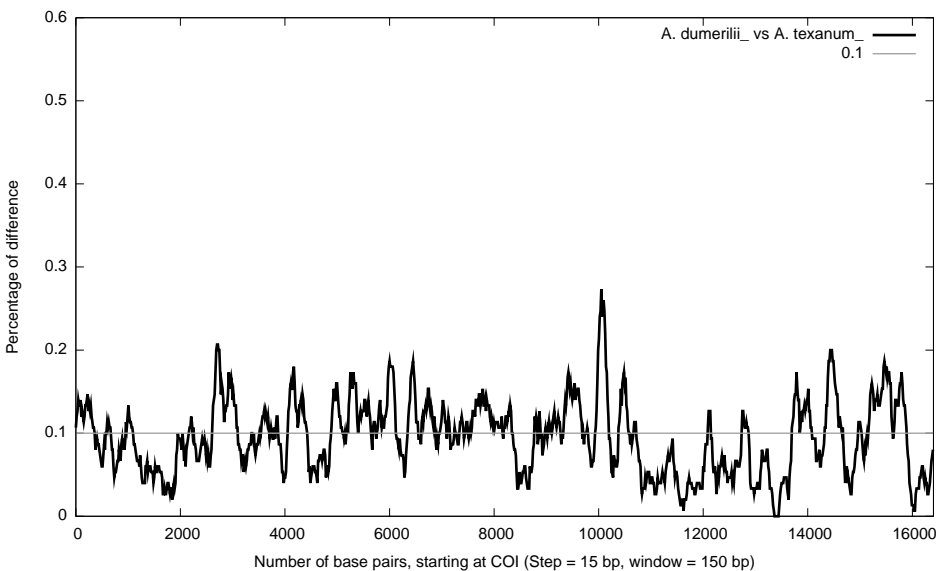

Supplement: File S3 — Sliding window analyses for Mammalia and Lissamphibia. For each family, the folder contains the aligned sequences as well as the sliding window analyses by species pair and for all species pair on a single figure. (ZIP) [file pone.0051263.s003.zip › Lissamphibia/Ambystomatidae/15_150/Ambystoma_dumerilii_NC_006889_Ambystoma_texanum_NC_014571.pdf]

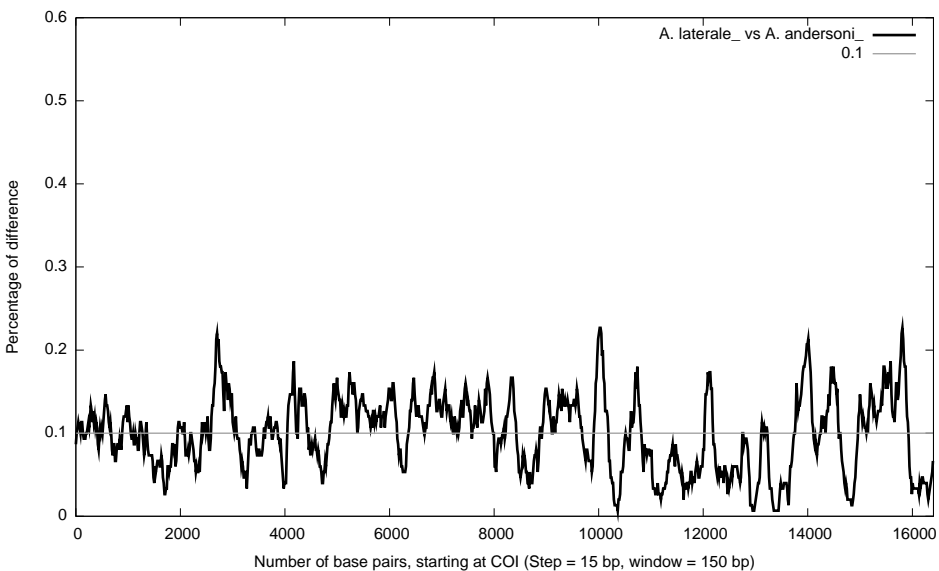

Supplement: File S3 — Sliding window analyses for Mammalia and Lissamphibia. For each family, the folder contains the aligned sequences as well as the sliding window analyses by species pair and for all species pair on a single figure. (ZIP) [file pone.0051263.s003.zip › Lissamphibia/Ambystomatidae/15_150/Ambystoma_laterale_NC_006330_Ambystoma_andersoni_NC_006888.pdf]

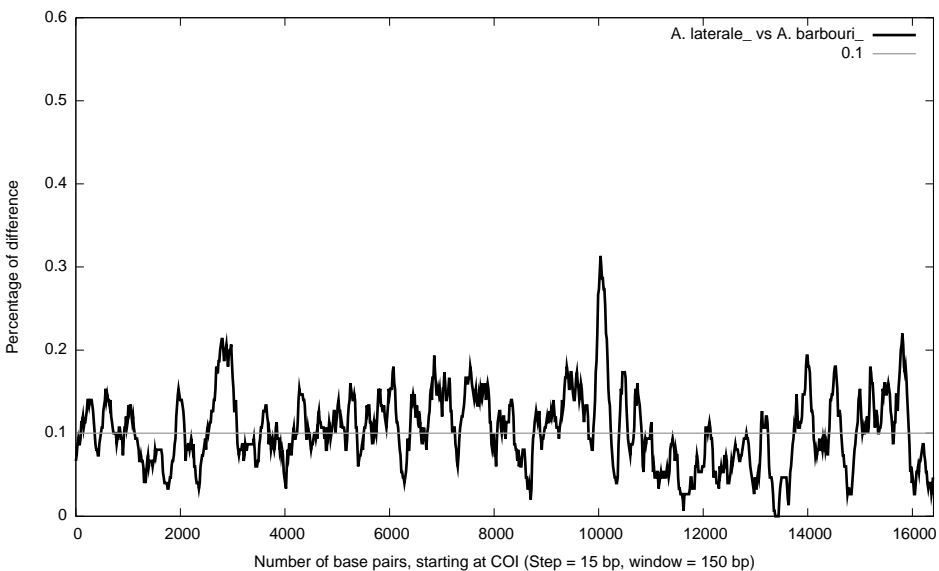

Supplement: File S3 — Sliding window analyses for Mammalia and Lissamphibia. For each family, the folder contains the aligned sequences as well as the sliding window analyses by species pair and for all species pair on a single figure. (ZIP) [file pone.0051263.s003.zip › Lissamphibia/Ambystomatidae/15_150/Ambystoma_laterale_NC_006330_Ambystoma_barbouri_NC_014568.pdf]

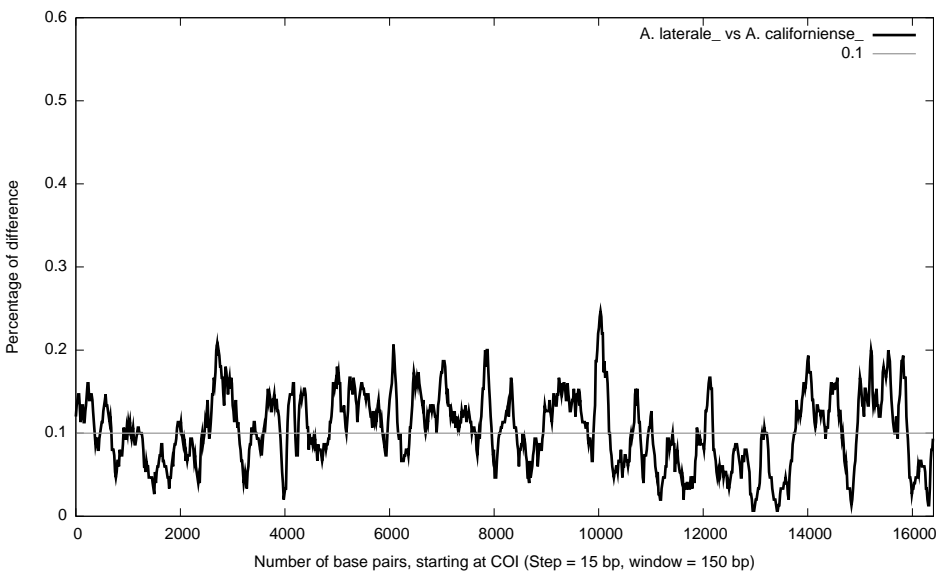

Supplement: File S3 — Sliding window analyses for Mammalia and Lissamphibia. For each family, the folder contains the aligned sequences as well as the sliding window analyses by species pair and for all species pair on a single figure. (ZIP) [file pone.0051263.s003.zip › Lissamphibia/Ambystomatidae/15_150/Ambystoma_laterale_NC_006330_Ambystoma_californiense_NC_006890.pdf]

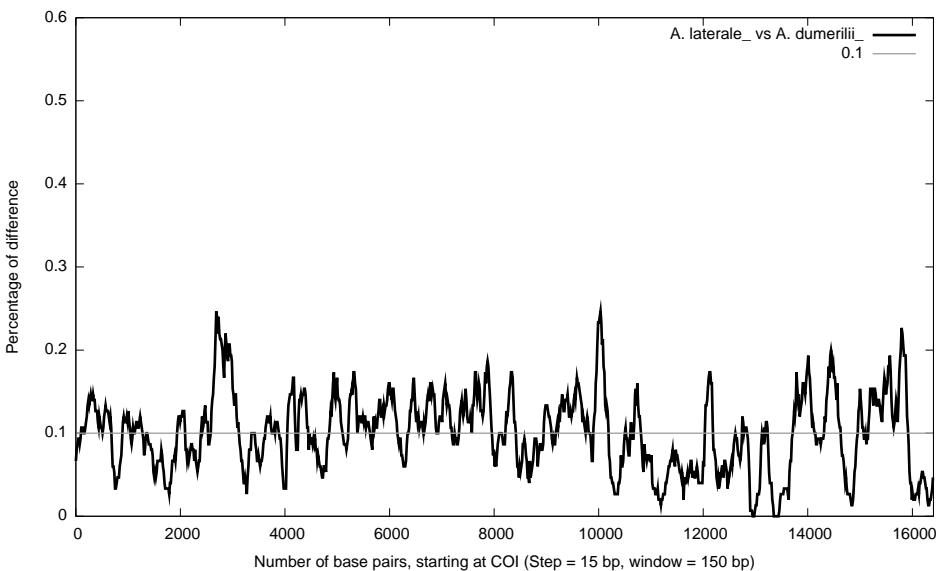

Supplement: File S3 — Sliding window analyses for Mammalia and Lissamphibia. For each family, the folder contains the aligned sequences as well as the sliding window analyses by species pair and for all species pair on a single figure. (ZIP) [file pone.0051263.s003.zip › Lissamphibia/Ambystomatidae/15_150/Ambystoma_laterale_NC_006330_Ambystoma_dumerilii_NC_006889.pdf]

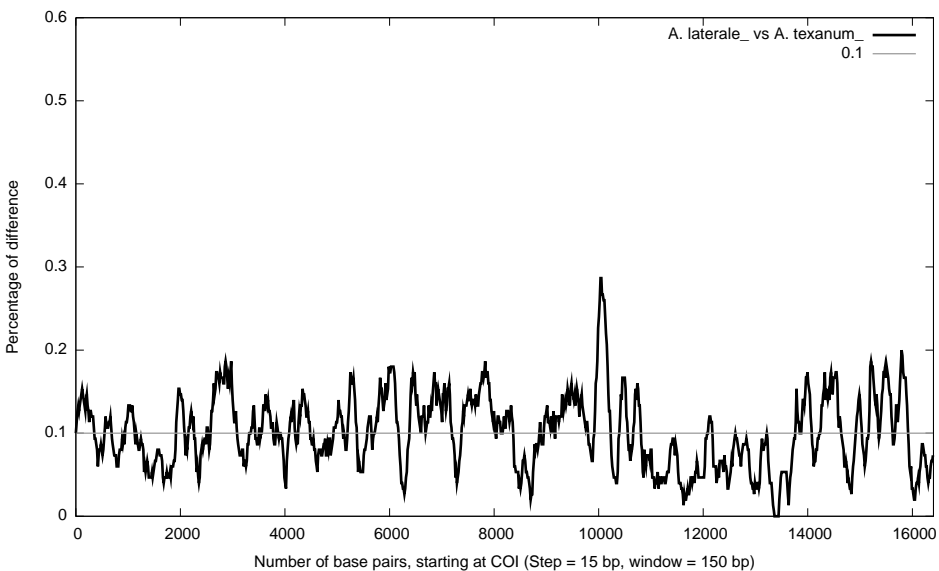

Supplement: File S3 — Sliding window analyses for Mammalia and Lissamphibia. For each family, the folder contains the aligned sequences as well as the sliding window analyses by species pair and for all species pair on a single figure. (ZIP) [file pone.0051263.s003.zip › Lissamphibia/Ambystomatidae/15_150/Ambystoma_laterale_NC_006330_Ambystoma_texanum_NC_014571.pdf]

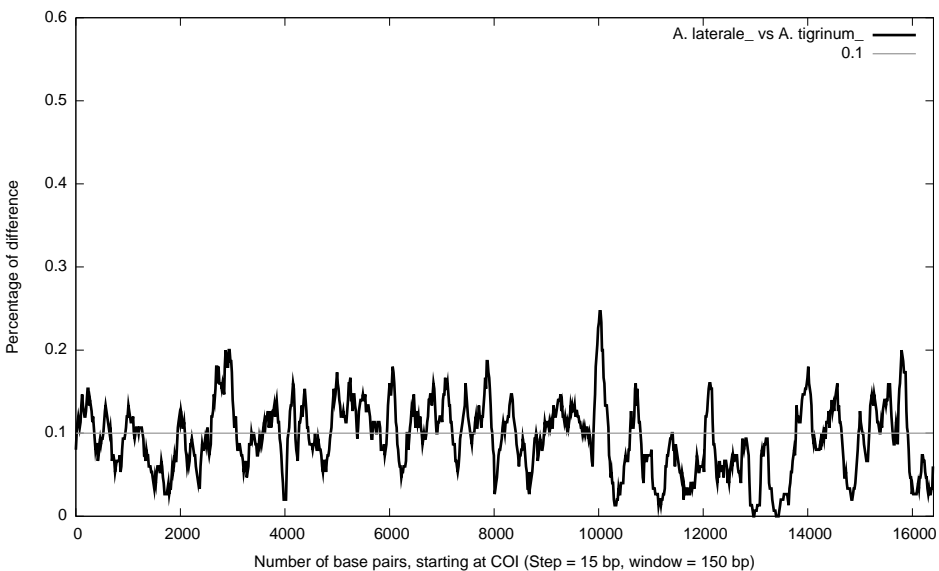

Supplement: File S3 — Sliding window analyses for Mammalia and Lissamphibia. For each family, the folder contains the aligned sequences as well as the sliding window analyses by species pair and for all species pair on a single figure. (ZIP) [file pone.0051263.s003.zip › Lissamphibia/Ambystomatidae/15_150/Ambystoma_laterale_NC_006330_Ambystoma_tigrinum_tigrinum_NC_006887.pdf]

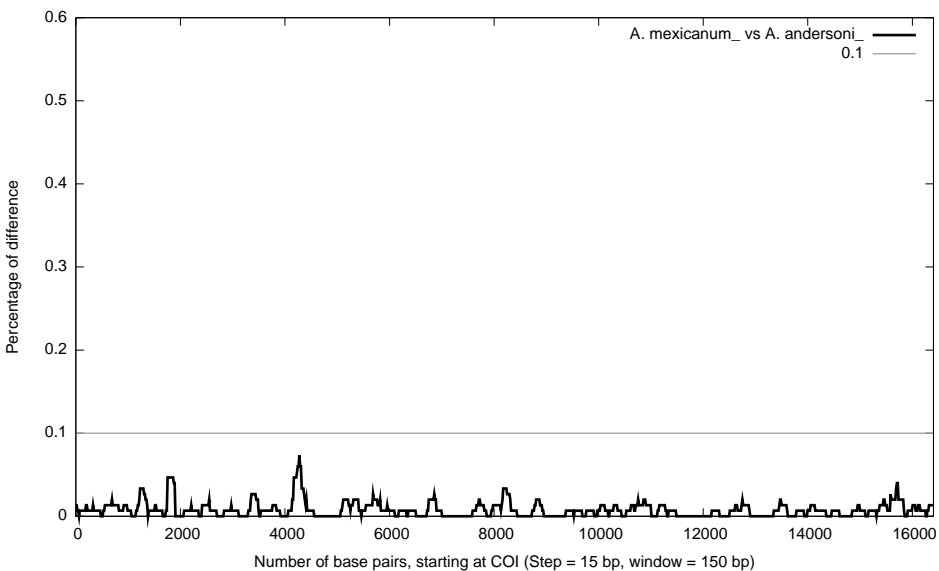

Supplement: File S3 — Sliding window analyses for Mammalia and Lissamphibia. For each family, the folder contains the aligned sequences as well as the sliding window analyses by species pair and for all species pair on a single figure. (ZIP) [file pone.0051263.s003.zip › Lissamphibia/Ambystomatidae/15_150/Ambystoma_mexicanum_NC_005797_Ambystoma_andersoni_NC_006888.pdf]

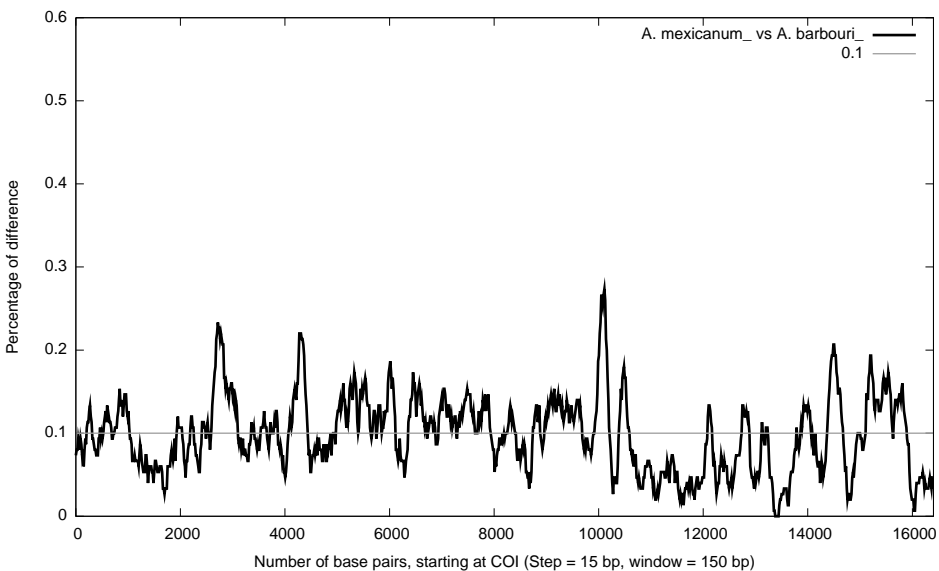

Supplement: File S3 — Sliding window analyses for Mammalia and Lissamphibia. For each family, the folder contains the aligned sequences as well as the sliding window analyses by species pair and for all species pair on a single figure. (ZIP) [file pone.0051263.s003.zip › Lissamphibia/Ambystomatidae/15_150/Ambystoma_mexicanum_NC_005797_Ambystoma_barbouri_NC_014568.pdf]

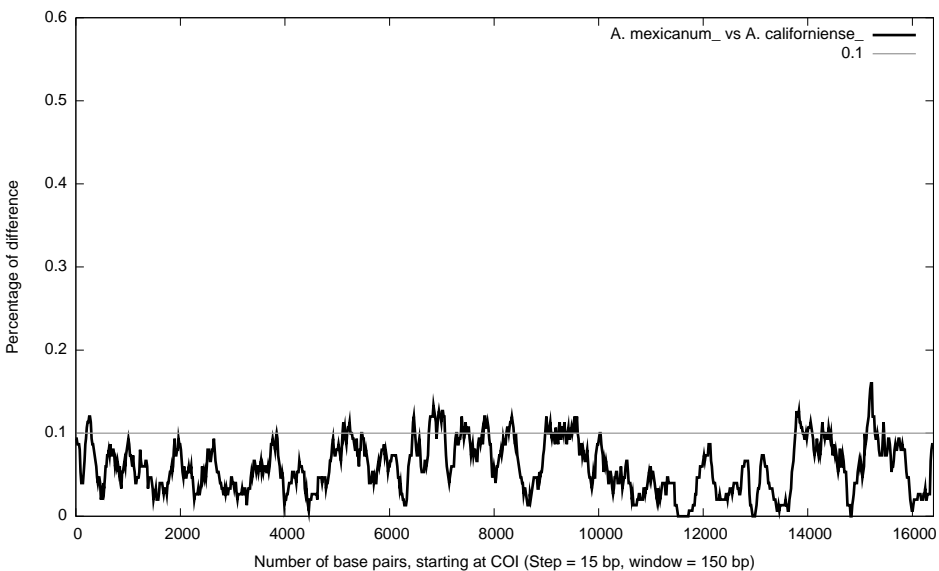

Supplement: File S3 — Sliding window analyses for Mammalia and Lissamphibia. For each family, the folder contains the aligned sequences as well as the sliding window analyses by species pair and for all species pair on a single figure. (ZIP) [file pone.0051263.s003.zip › Lissamphibia/Ambystomatidae/15_150/Ambystoma_mexicanum_NC_005797_Ambystoma_californiense_NC_006890.pdf]

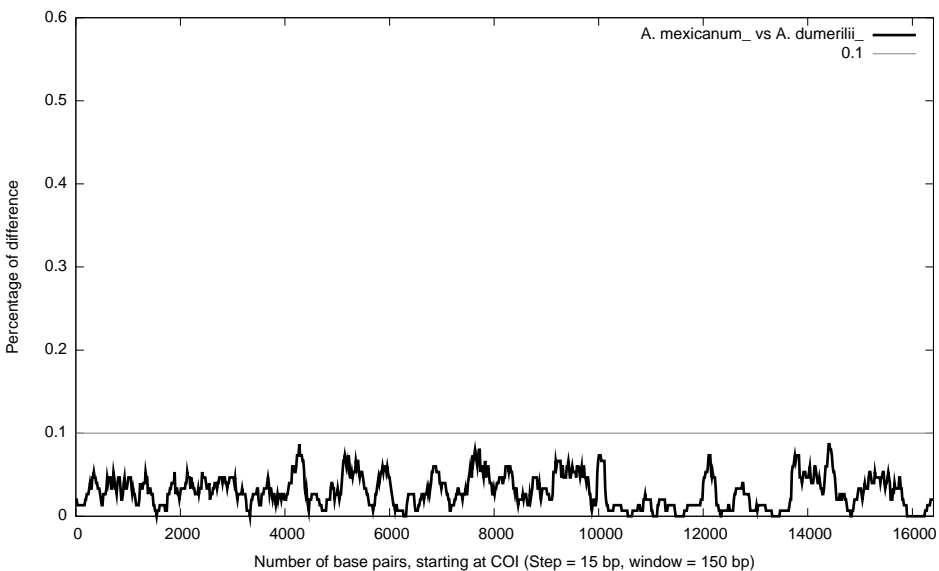

Supplement: File S3 — Sliding window analyses for Mammalia and Lissamphibia. For each family, the folder contains the aligned sequences as well as the sliding window analyses by species pair and for all species pair on a single figure. (ZIP) [file pone.0051263.s003.zip › Lissamphibia/Ambystomatidae/15_150/Ambystoma_mexicanum_NC_005797_Ambystoma_dumerilii_NC_006889.pdf]

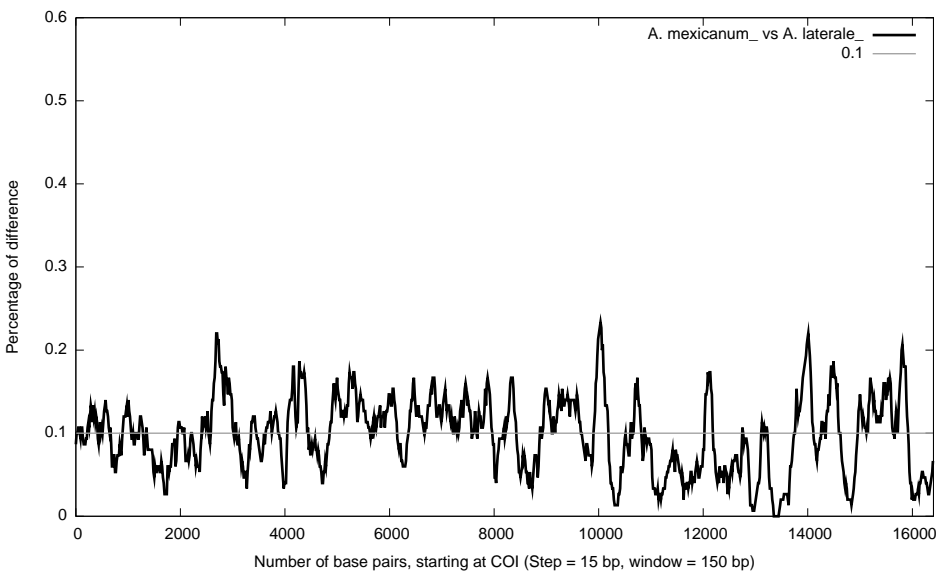

Supplement: File S3 — Sliding window analyses for Mammalia and Lissamphibia. For each family, the folder contains the aligned sequences as well as the sliding window analyses by species pair and for all species pair on a single figure. (ZIP) [file pone.0051263.s003.zip › Lissamphibia/Ambystomatidae/15_150/Ambystoma_mexicanum_NC_005797_Ambystoma_laterale_NC_006330.pdf]

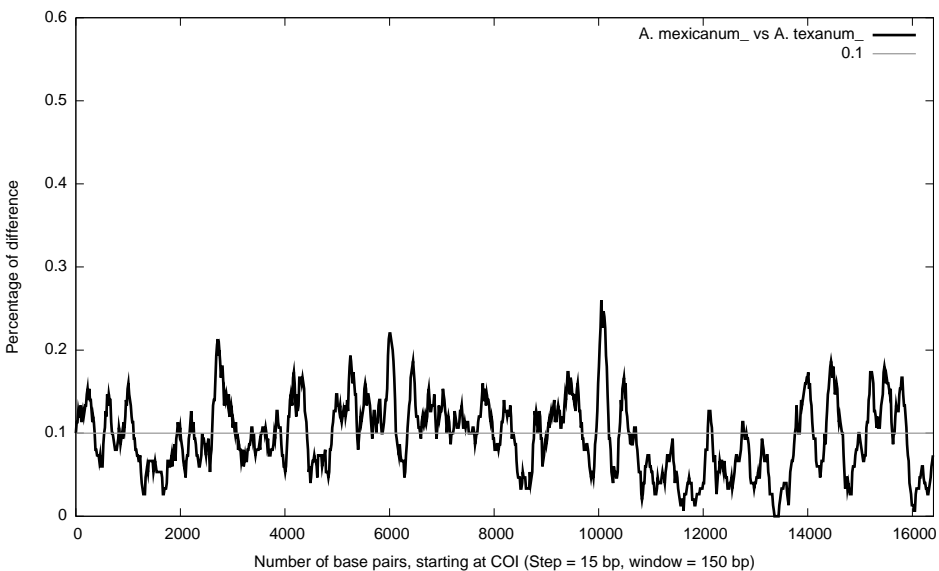

Supplement: File S3 — Sliding window analyses for Mammalia and Lissamphibia. For each family, the folder contains the aligned sequences as well as the sliding window analyses by species pair and for all species pair on a single figure. (ZIP) [file pone.0051263.s003.zip › Lissamphibia/Ambystomatidae/15_150/Ambystoma_mexicanum_NC_005797_Ambystoma_texanum_NC_014571.pdf]

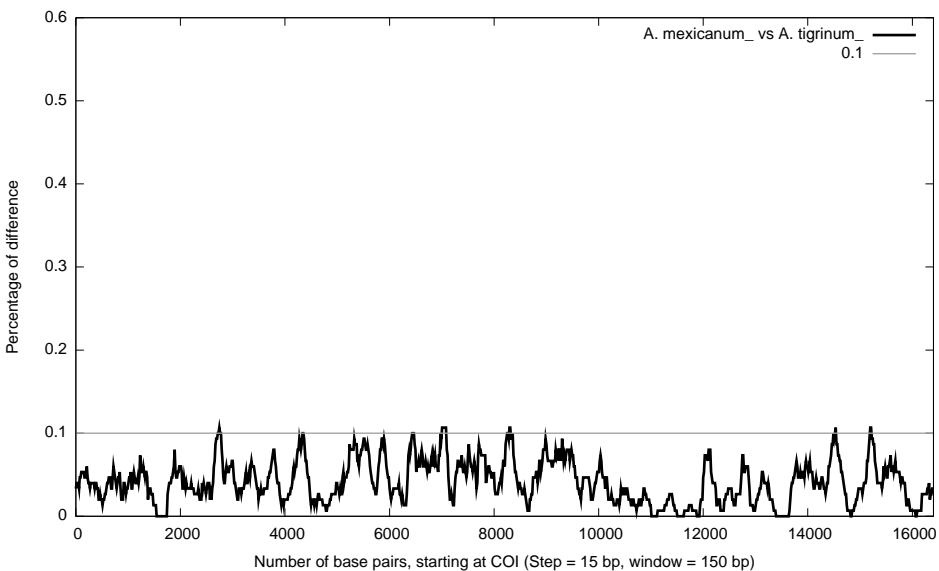

Supplement: File S3 — Sliding window analyses for Mammalia and Lissamphibia. For each family, the folder contains the aligned sequences as well as the sliding window analyses by species pair and for all species pair on a single figure. (ZIP) [file pone.0051263.s003.zip › Lissamphibia/Ambystomatidae/15_150/Ambystoma_mexicanum_NC_005797_Ambystoma_tigrinum_tigrinum_NC_006887.pdf]

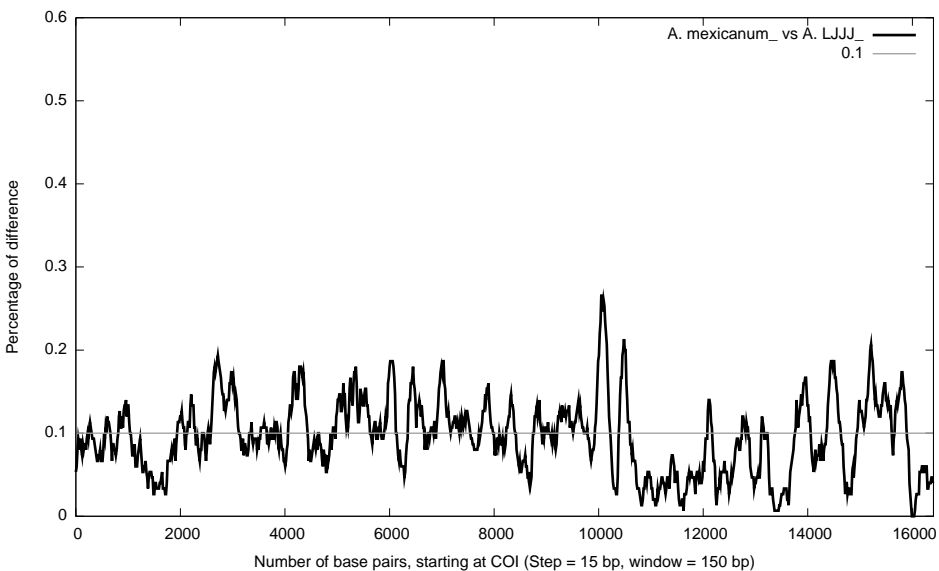

Supplement: File S3 — Sliding window analyses for Mammalia and Lissamphibia. For each family, the folder contains the aligned sequences as well as the sliding window analyses by species pair and for all species pair on a single figure. (ZIP) [file pone.0051263.s003.zip › Lissamphibia/Ambystomatidae/15_150/Ambystoma_mexicanum_NC_005797_Ambystoma_unisexual_lineage_LJJJ_NC_014572.pdf]

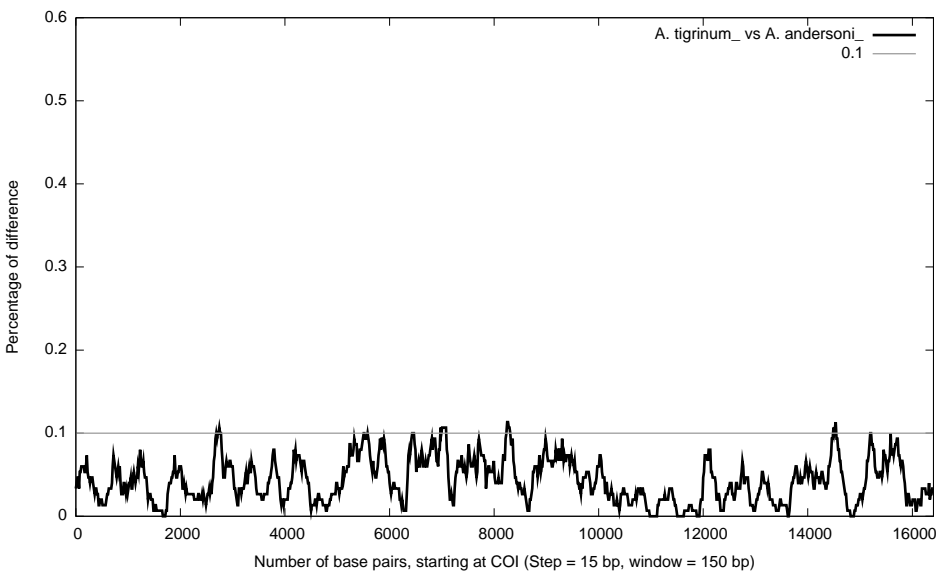

Supplement: File S3 — Sliding window analyses for Mammalia and Lissamphibia. For each family, the folder contains the aligned sequences as well as the sliding window analyses by species pair and for all species pair on a single figure. (ZIP) [file pone.0051263.s003.zip › Lissamphibia/Ambystomatidae/15_150/Ambystoma_tigrinum_tigrinum_NC_006887_Ambystoma_andersoni_NC_006888.pdf]

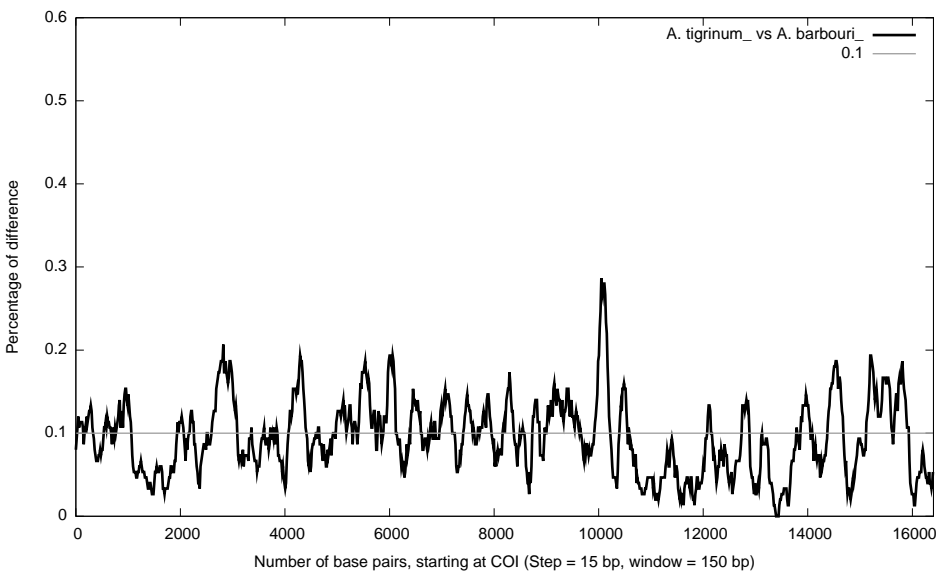

Supplement: File S3 — Sliding window analyses for Mammalia and Lissamphibia. For each family, the folder contains the aligned sequences as well as the sliding window analyses by species pair and for all species pair on a single figure. (ZIP) [file pone.0051263.s003.zip › Lissamphibia/Ambystomatidae/15_150/Ambystoma_tigrinum_tigrinum_NC_006887_Ambystoma_barbouri_NC_014568.pdf]

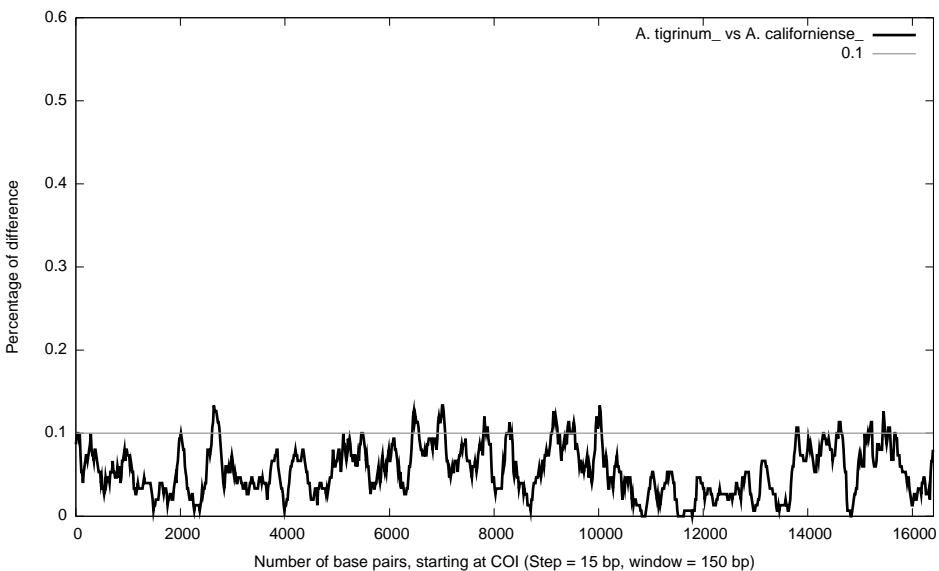

Supplement: File S3 — Sliding window analyses for Mammalia and Lissamphibia. For each family, the folder contains the aligned sequences as well as the sliding window analyses by species pair and for all species pair on a single figure. (ZIP) [file pone.0051263.s003.zip › Lissamphibia/Ambystomatidae/15_150/Ambystoma_tigrinum_tigrinum_NC_006887_Ambystoma_californiense_NC_006890.pdf]

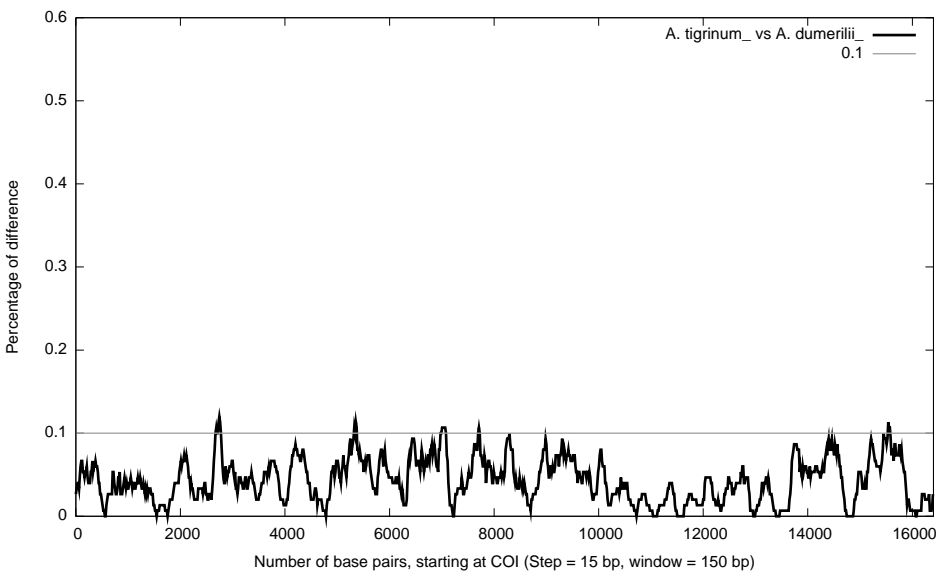

Supplement: File S3 — Sliding window analyses for Mammalia and Lissamphibia. For each family, the folder contains the aligned sequences as well as the sliding window analyses by species pair and for all species pair on a single figure. (ZIP) [file pone.0051263.s003.zip › Lissamphibia/Ambystomatidae/15_150/Ambystoma_tigrinum_tigrinum_NC_006887_Ambystoma_dumerilii_NC_006889.pdf]

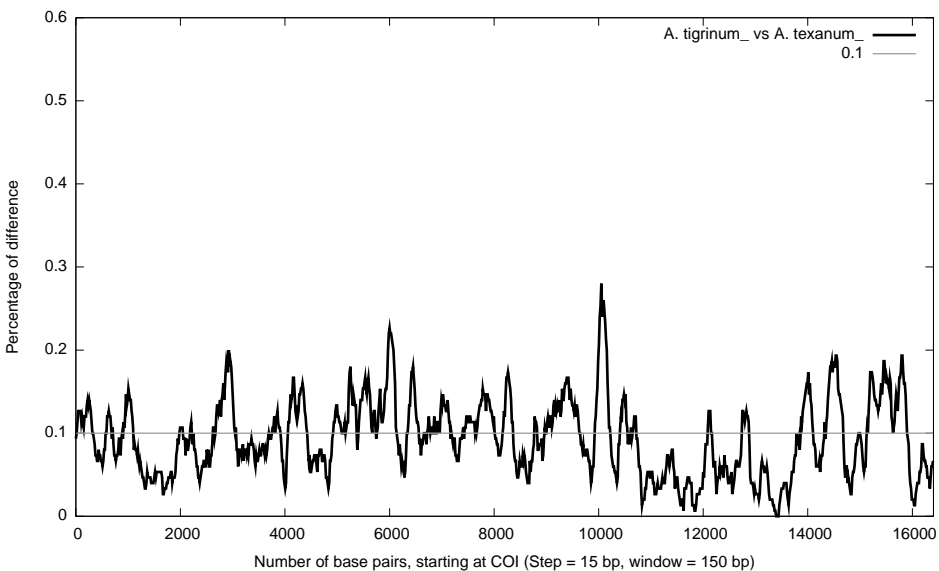

Supplement: File S3 — Sliding window analyses for Mammalia and Lissamphibia. For each family, the folder contains the aligned sequences as well as the sliding window analyses by species pair and for all species pair on a single figure. (ZIP) [file pone.0051263.s003.zip › Lissamphibia/Ambystomatidae/15_150/Ambystoma_tigrinum_tigrinum_NC_006887_Ambystoma_texanum_NC_014571.pdf]

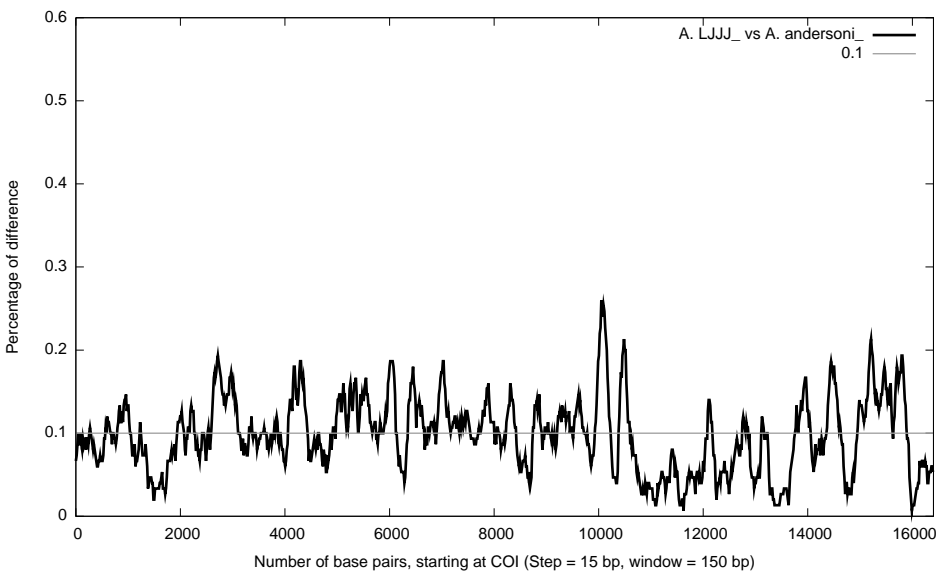

Supplement: File S3 — Sliding window analyses for Mammalia and Lissamphibia. For each family, the folder contains the aligned sequences as well as the sliding window analyses by species pair and for all species pair on a single figure. (ZIP) [file pone.0051263.s003.zip › Lissamphibia/Ambystomatidae/15_150/Ambystoma_unisexual_lineage_LJJJ_NC_014572_Ambystoma_andersoni_NC_006888.pdf]

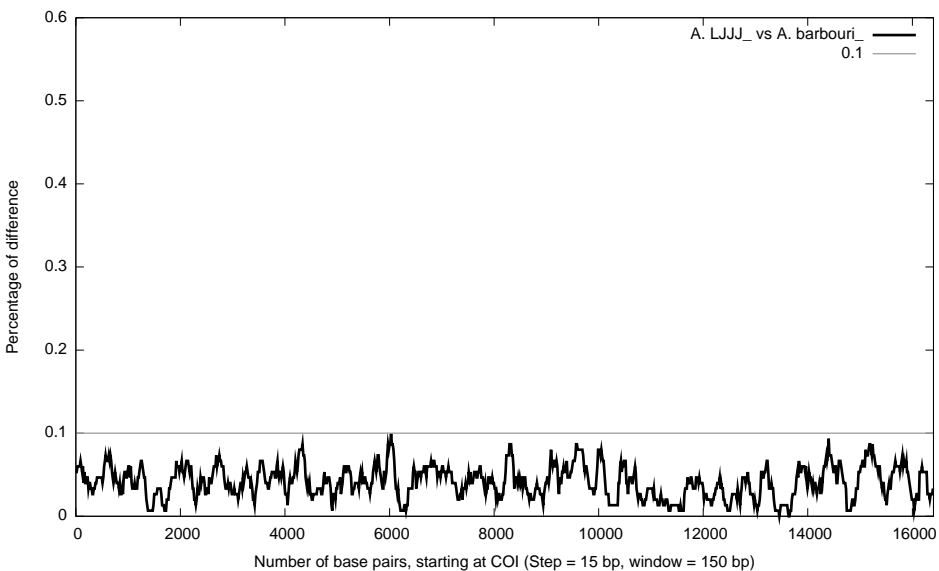

Supplement: File S3 — Sliding window analyses for Mammalia and Lissamphibia. For each family, the folder contains the aligned sequences as well as the sliding window analyses by species pair and for all species pair on a single figure. (ZIP) [file pone.0051263.s003.zip › Lissamphibia/Ambystomatidae/15_150/Ambystoma_unisexual_lineage_LJJJ_NC_014572_Ambystoma_barbouri_NC_014568.pdf]

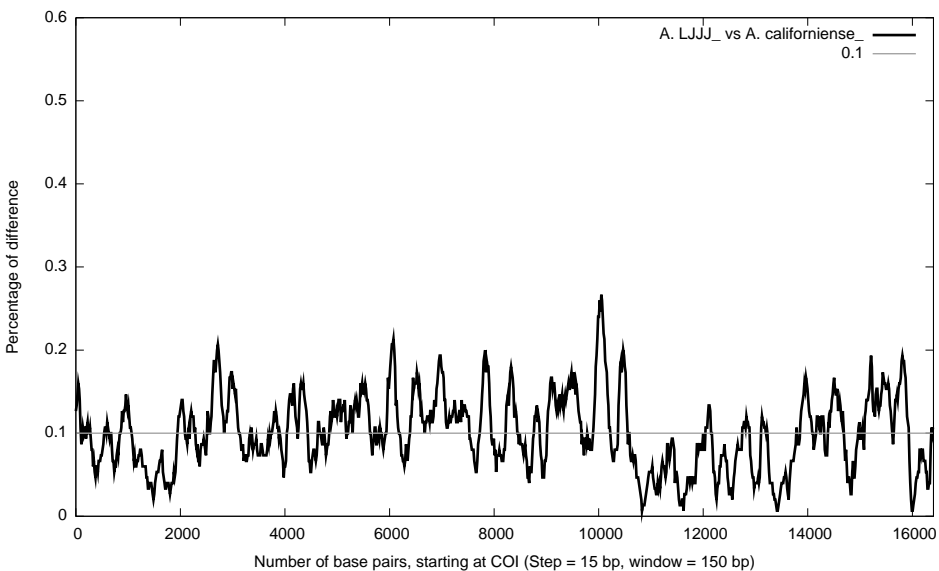

Supplement: File S3 — Sliding window analyses for Mammalia and Lissamphibia. For each family, the folder contains the aligned sequences as well as the sliding window analyses by species pair and for all species pair on a single figure. (ZIP) [file pone.0051263.s003.zip › Lissamphibia/Ambystomatidae/15_150/Ambystoma_unisexual_lineage_LJJJ_NC_014572_Ambystoma_californiense_NC_006890.pdf]

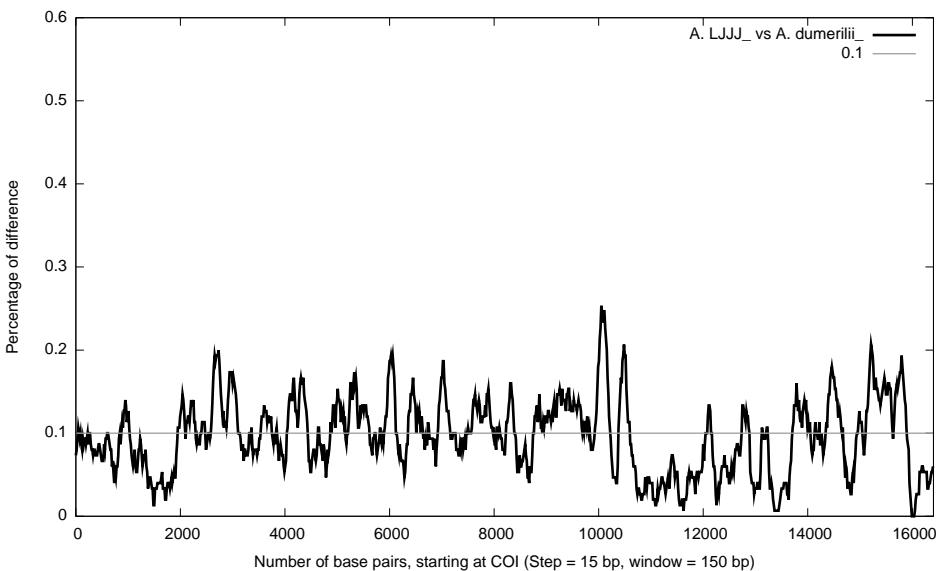

Supplement: File S3 — Sliding window analyses for Mammalia and Lissamphibia. For each family, the folder contains the aligned sequences as well as the sliding window analyses by species pair and for all species pair on a single figure. (ZIP) [file pone.0051263.s003.zip › Lissamphibia/Ambystomatidae/15_150/Ambystoma_unisexual_lineage_LJJJ_NC_014572_Ambystoma_dumerilii_NC_006889.pdf]

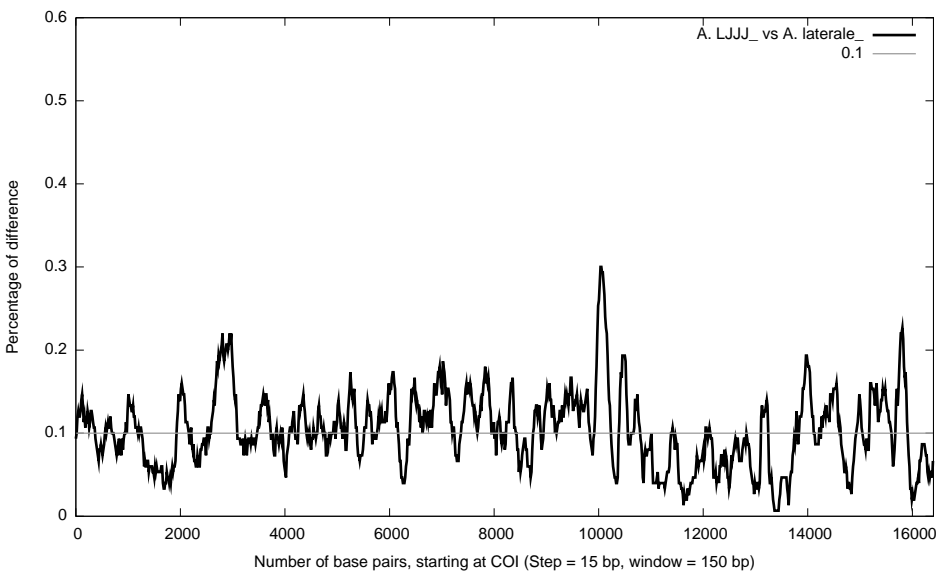

Supplement: File S3 — Sliding window analyses for Mammalia and Lissamphibia. For each family, the folder contains the aligned sequences as well as the sliding window analyses by species pair and for all species pair on a single figure. (ZIP) [file pone.0051263.s003.zip › Lissamphibia/Ambystomatidae/15_150/Ambystoma_unisexual_lineage_LJJJ_NC_014572_Ambystoma_laterale_NC_006330.pdf]

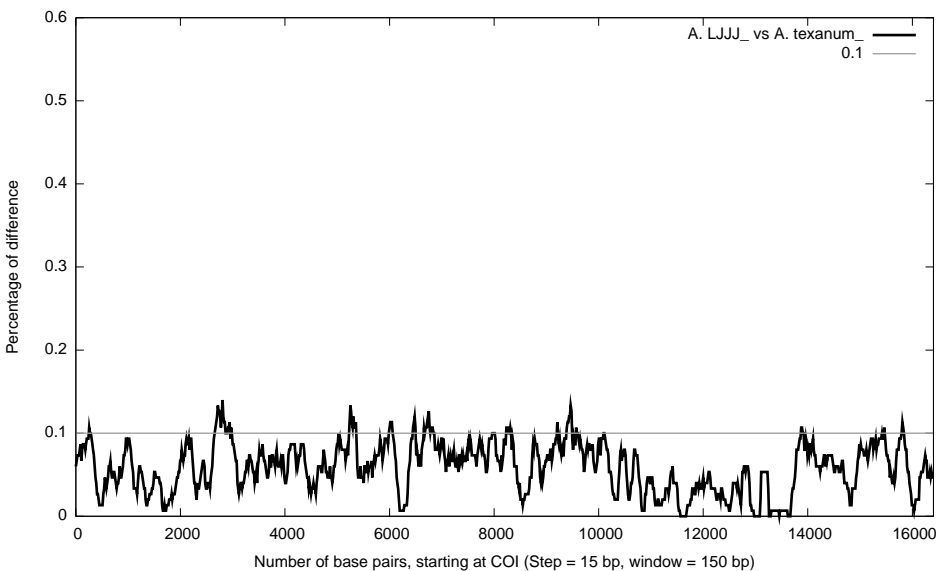

Supplement: File S3 — Sliding window analyses for Mammalia and Lissamphibia. For each family, the folder contains the aligned sequences as well as the sliding window analyses by species pair and for all species pair on a single figure. (ZIP) [file pone.0051263.s003.zip › Lissamphibia/Ambystomatidae/15_150/Ambystoma_unisexual_lineage_LJJJ_NC_014572_Ambystoma_texanum_NC_014571.pdf]
