# Supplementary figures and images for: Conveniently Pre-Tagged and Pre-Packaged: Extended Molecular Identification and Metagenomics Using Complete Metazoan Mitochondrial Genomes (part 2 of 3)
Source: PLoS One. 2012 Dec 14;7(12):e51263. doi: 10.1371/journal.pone.0051263 (PMC3522660; doi:10.1371/journal.pone.0051263)

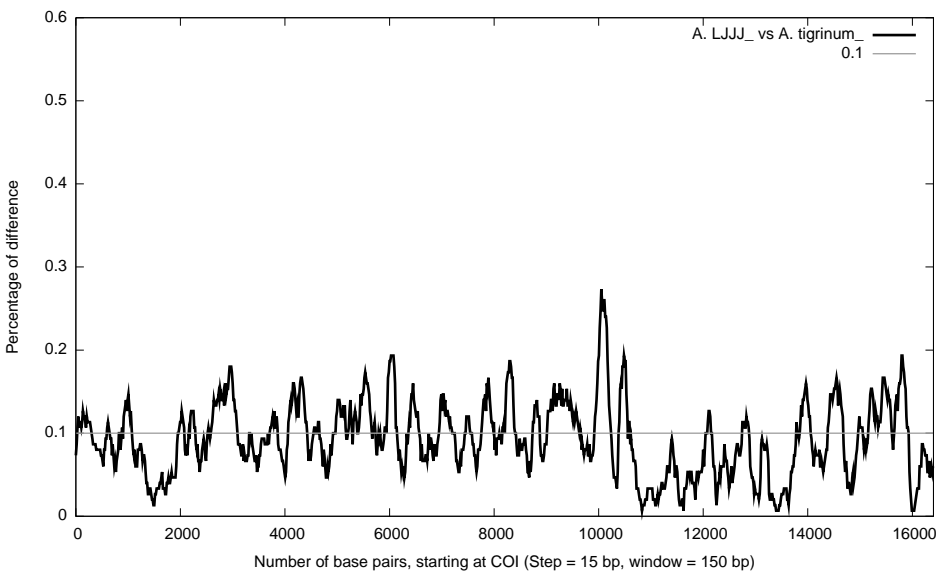

Supplement: File S3 — Sliding window analyses for Mammalia and Lissamphibia. For each family, the folder contains the aligned sequences as well as the sliding window analyses by species pair and for all species pair on a single figure. (ZIP) [file pone.0051263.s003.zip › Lissamphibia/Ambystomatidae/15_150/Ambystoma_unisexual_lineage_LJJJ_NC_014572_Ambystoma_tigrinum_tigrinum_NC_006887.pdf]

# Ambystomatidae

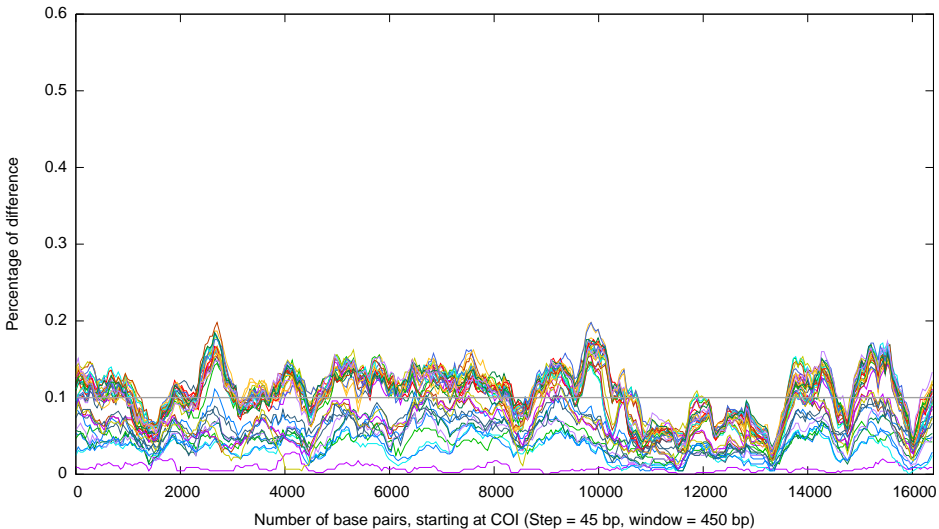

Supplement: File S3 — Sliding window analyses for Mammalia and Lissamphibia. For each family, the folder contains the aligned sequences as well as the sliding window analyses by species pair and for all species pair on a single figure. (ZIP) [file pone.0051263.s003.zip › Lissamphibia/Ambystomatidae/45_450/allCurves.pdf]

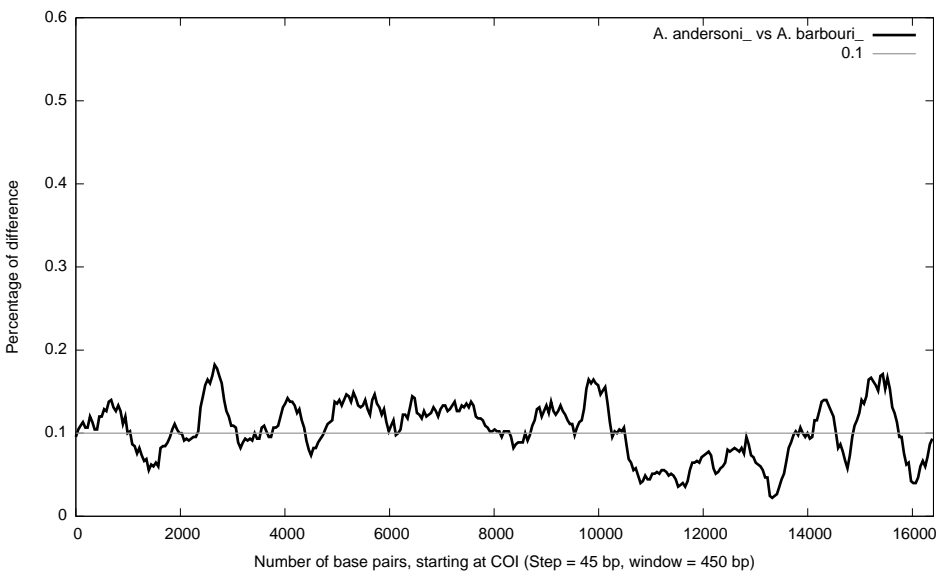

Supplement: File S3 — Sliding window analyses for Mammalia and Lissamphibia. For each family, the folder contains the aligned sequences as well as the sliding window analyses by species pair and for all species pair on a single figure. (ZIP) [file pone.0051263.s003.zip › Lissamphibia/Ambystomatidae/45_450/Ambystoma_andersoni_NC_006888_Ambystoma_barbouri_NC_014568.pdf]

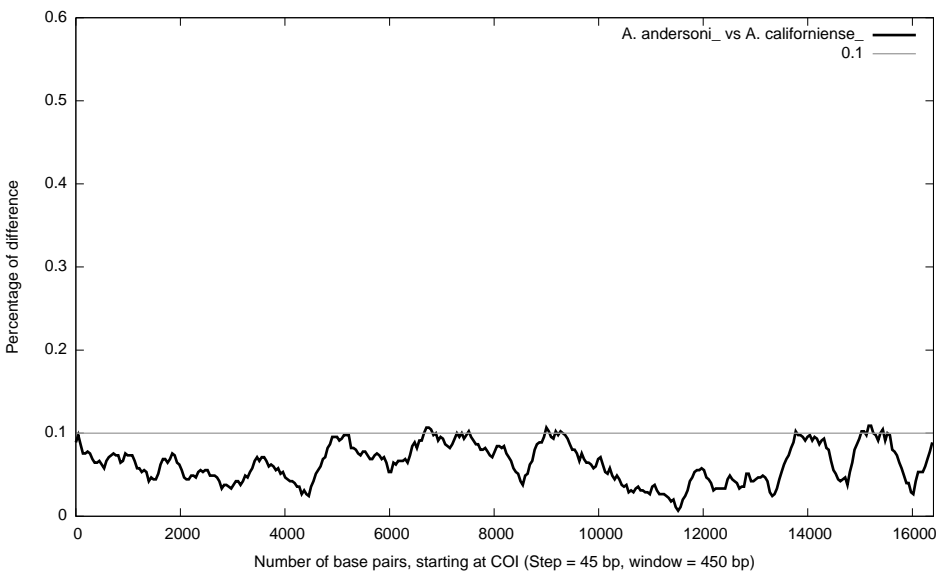

Supplement: File S3 — Sliding window analyses for Mammalia and Lissamphibia. For each family, the folder contains the aligned sequences as well as the sliding window analyses by species pair and for all species pair on a single figure. (ZIP) [file pone.0051263.s003.zip › Lissamphibia/Ambystomatidae/45_450/Ambystoma_andersoni_NC_006888_Ambystoma_californiense_NC_006890.pdf]

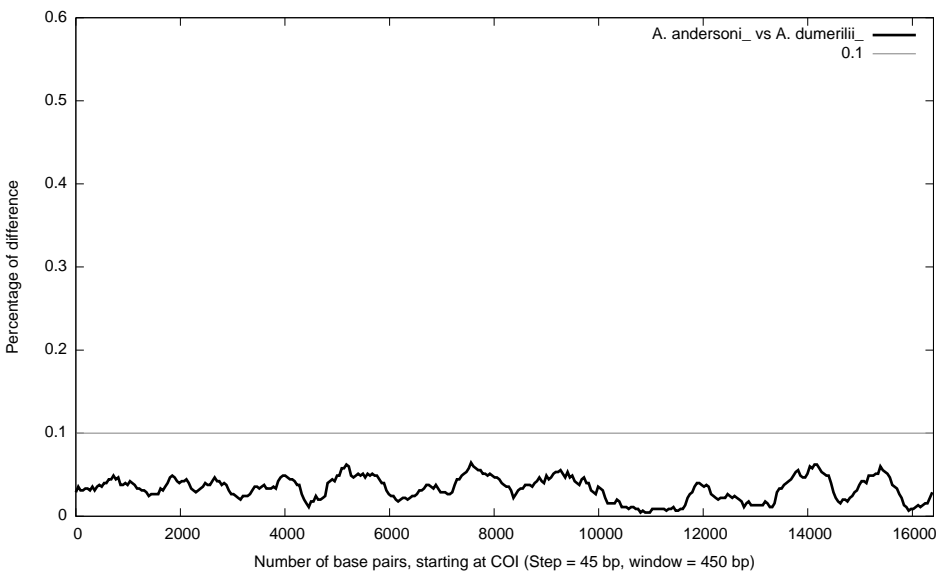

Supplement: File S3 — Sliding window analyses for Mammalia and Lissamphibia. For each family, the folder contains the aligned sequences as well as the sliding window analyses by species pair and for all species pair on a single figure. (ZIP) [file pone.0051263.s003.zip › Lissamphibia/Ambystomatidae/45_450/Ambystoma_andersoni_NC_006888_Ambystoma_dumerilii_NC_006889.pdf]

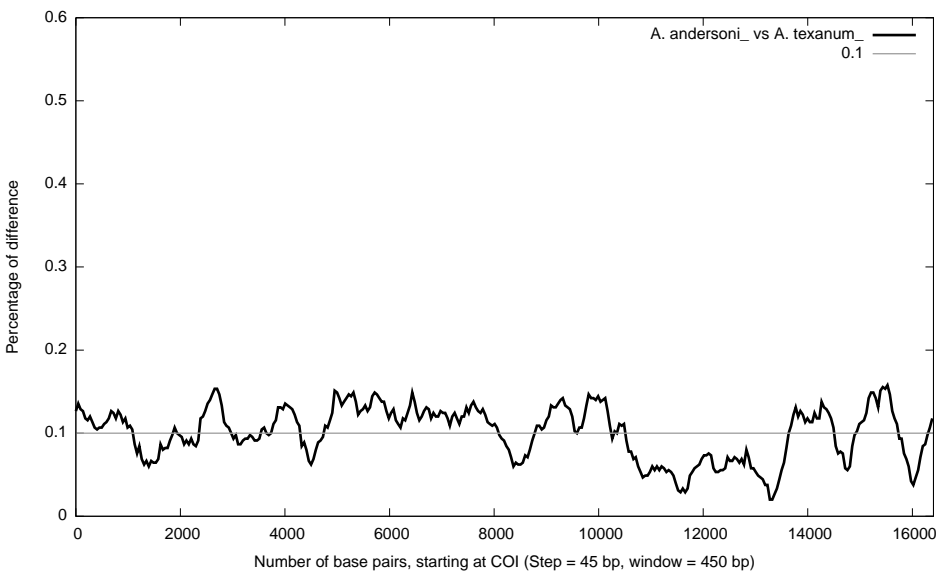

Supplement: File S3 — Sliding window analyses for Mammalia and Lissamphibia. For each family, the folder contains the aligned sequences as well as the sliding window analyses by species pair and for all species pair on a single figure. (ZIP) [file pone.0051263.s003.zip › Lissamphibia/Ambystomatidae/45_450/Ambystoma_andersoni_NC_006888_Ambystoma_texanum_NC_014571.pdf]

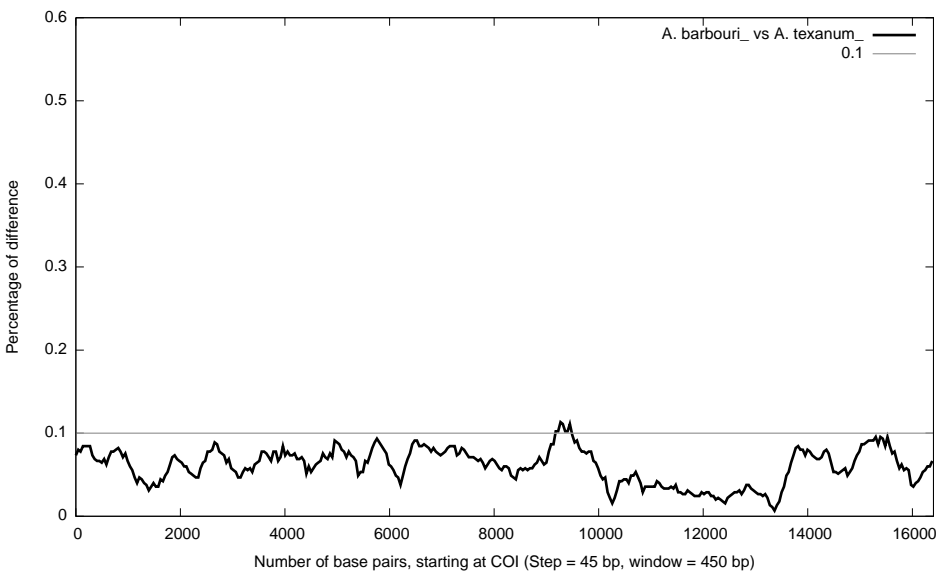

Supplement: File S3 — Sliding window analyses for Mammalia and Lissamphibia. For each family, the folder contains the aligned sequences as well as the sliding window analyses by species pair and for all species pair on a single figure. (ZIP) [file pone.0051263.s003.zip › Lissamphibia/Ambystomatidae/45_450/Ambystoma_barbouri_NC_014568_Ambystoma_texanum_NC_014571.pdf]

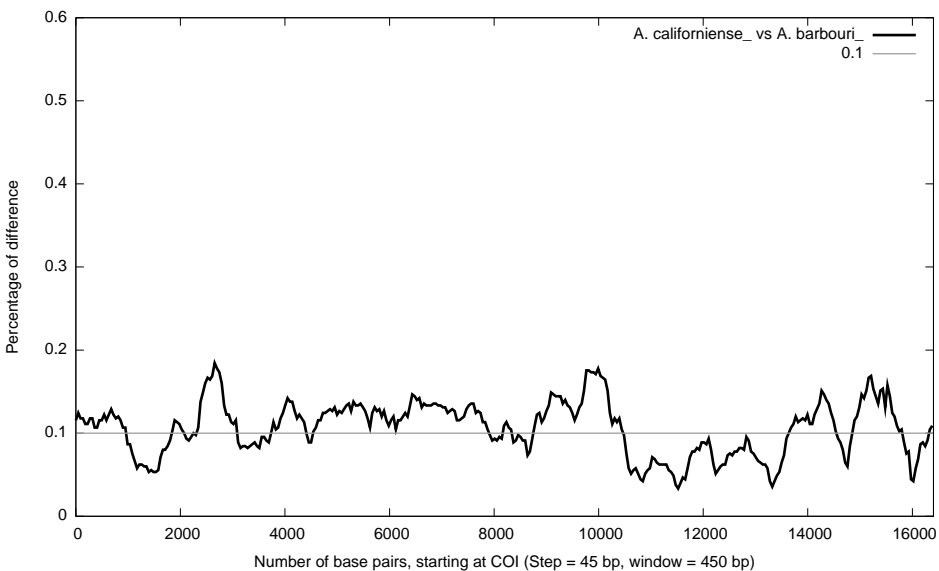

Supplement: File S3 — Sliding window analyses for Mammalia and Lissamphibia. For each family, the folder contains the aligned sequences as well as the sliding window analyses by species pair and for all species pair on a single figure. (ZIP) [file pone.0051263.s003.zip › Lissamphibia/Ambystomatidae/45_450/Ambystoma_californiense_NC_006890_Ambystoma_barbouri_NC_014568.pdf]

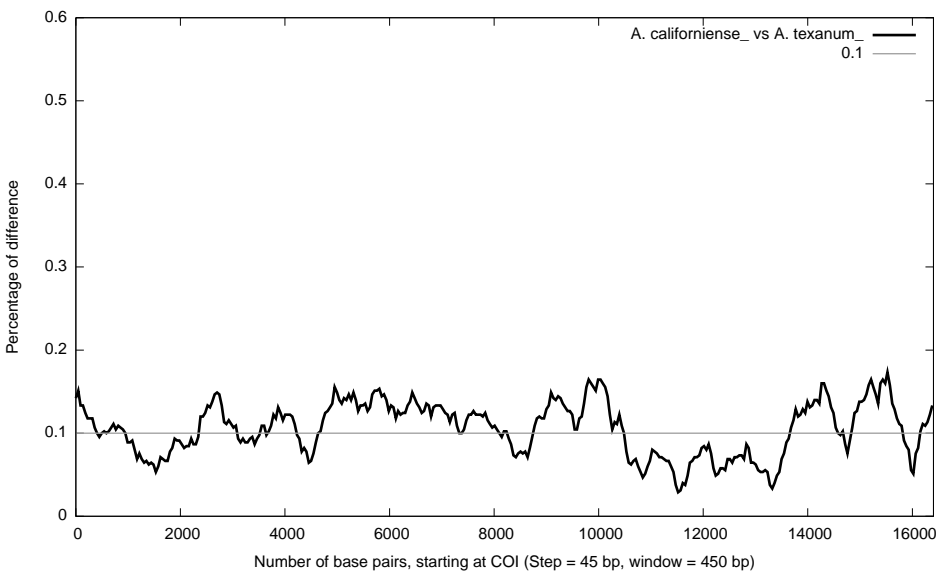

Supplement: File S3 — Sliding window analyses for Mammalia and Lissamphibia. For each family, the folder contains the aligned sequences as well as the sliding window analyses by species pair and for all species pair on a single figure. (ZIP) [file pone.0051263.s003.zip › Lissamphibia/Ambystomatidae/45_450/Ambystoma_californiense_NC_006890_Ambystoma_texanum_NC_014571.pdf]

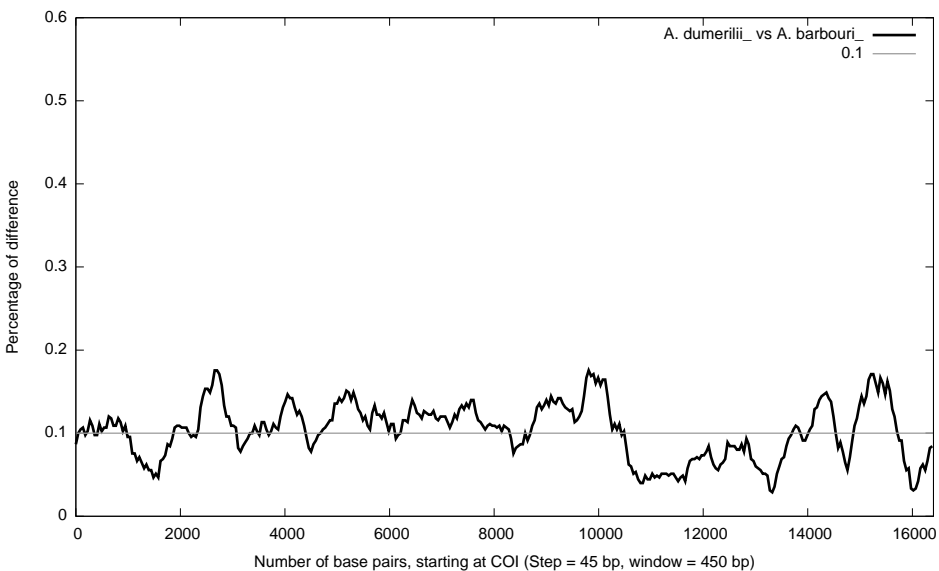

Supplement: File S3 — Sliding window analyses for Mammalia and Lissamphibia. For each family, the folder contains the aligned sequences as well as the sliding window analyses by species pair and for all species pair on a single figure. (ZIP) [file pone.0051263.s003.zip › Lissamphibia/Ambystomatidae/45_450/Ambystoma_dumerilii_NC_006889_Ambystoma_barbouri_NC_014568.pdf]

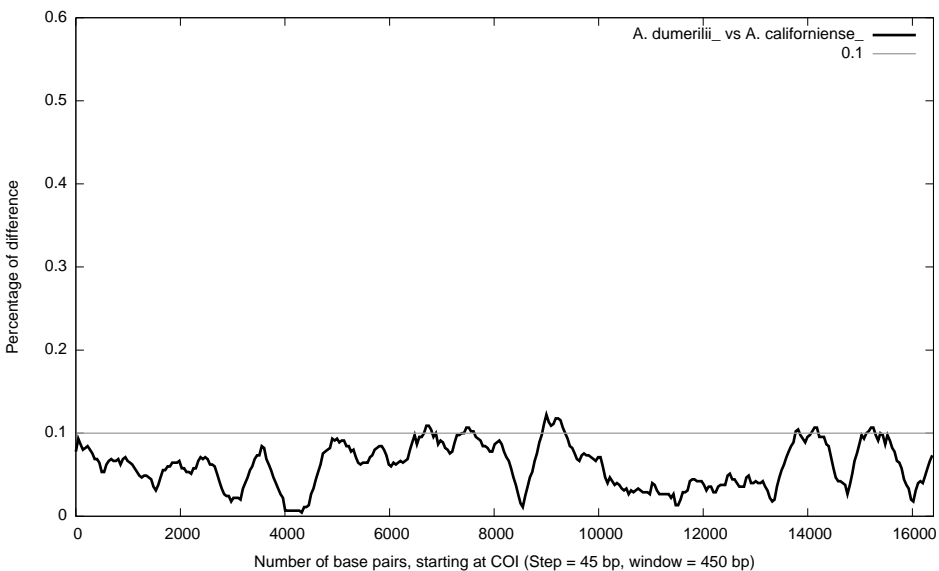

Supplement: File S3 — Sliding window analyses for Mammalia and Lissamphibia. For each family, the folder contains the aligned sequences as well as the sliding window analyses by species pair and for all species pair on a single figure. (ZIP) [file pone.0051263.s003.zip › Lissamphibia/Ambystomatidae/45_450/Ambystoma_dumerilii_NC_006889_Ambystoma_californiense_NC_006890.pdf]

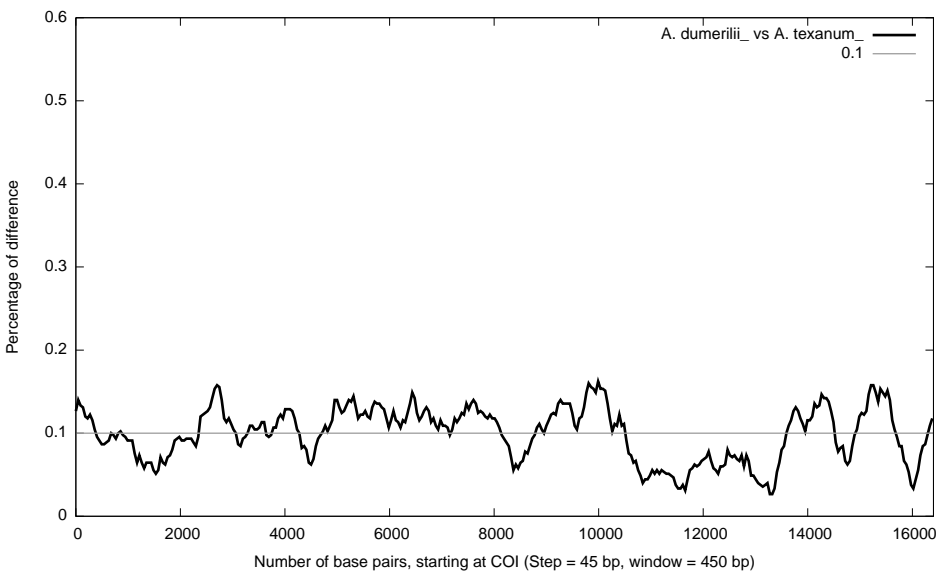

Supplement: File S3 — Sliding window analyses for Mammalia and Lissamphibia. For each family, the folder contains the aligned sequences as well as the sliding window analyses by species pair and for all species pair on a single figure. (ZIP) [file pone.0051263.s003.zip › Lissamphibia/Ambystomatidae/45_450/Ambystoma_dumerilii_NC_006889_Ambystoma_texanum_NC_014571.pdf]

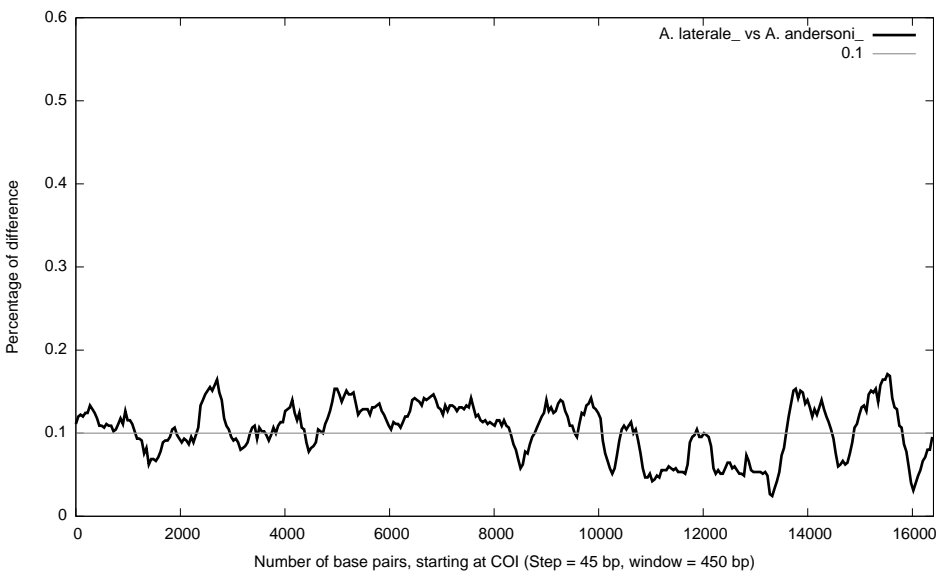

Supplement: File S3 — Sliding window analyses for Mammalia and Lissamphibia. For each family, the folder contains the aligned sequences as well as the sliding window analyses by species pair and for all species pair on a single figure. (ZIP) [file pone.0051263.s003.zip › Lissamphibia/Ambystomatidae/45_450/Ambystoma_laterale_NC_006330_Ambystoma_andersoni_NC_006888.pdf]

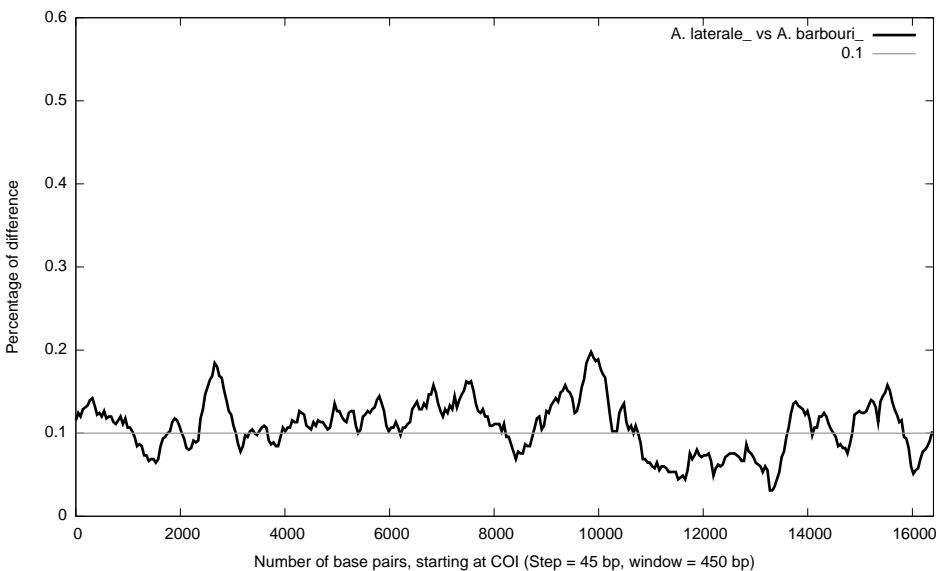

Supplement: File S3 — Sliding window analyses for Mammalia and Lissamphibia. For each family, the folder contains the aligned sequences as well as the sliding window analyses by species pair and for all species pair on a single figure. (ZIP) [file pone.0051263.s003.zip › Lissamphibia/Ambystomatidae/45_450/Ambystoma_laterale_NC_006330_Ambystoma_barbouri_NC_014568.pdf]

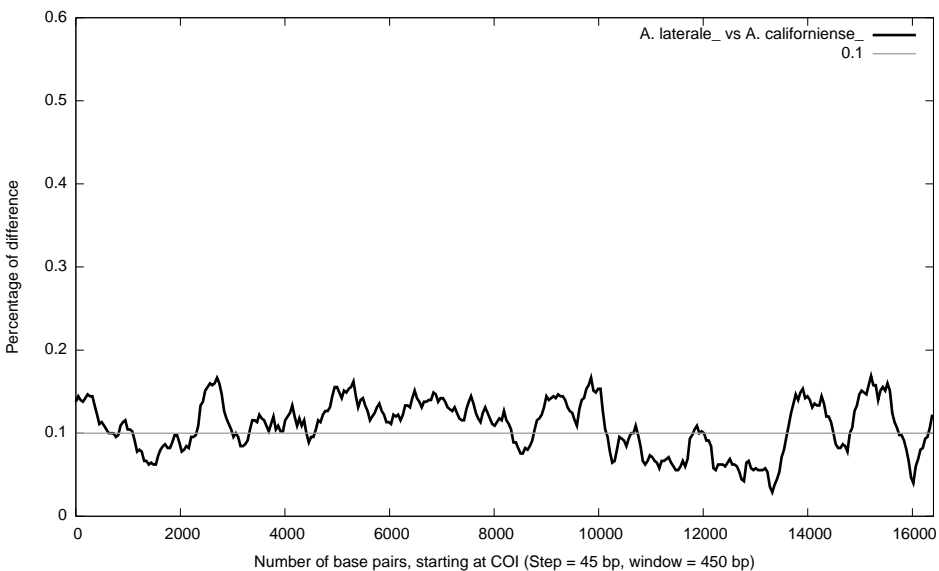

Supplement: File S3 — Sliding window analyses for Mammalia and Lissamphibia. For each family, the folder contains the aligned sequences as well as the sliding window analyses by species pair and for all species pair on a single figure. (ZIP) [file pone.0051263.s003.zip › Lissamphibia/Ambystomatidae/45_450/Ambystoma_laterale_NC_006330_Ambystoma_californiense_NC_006890.pdf]

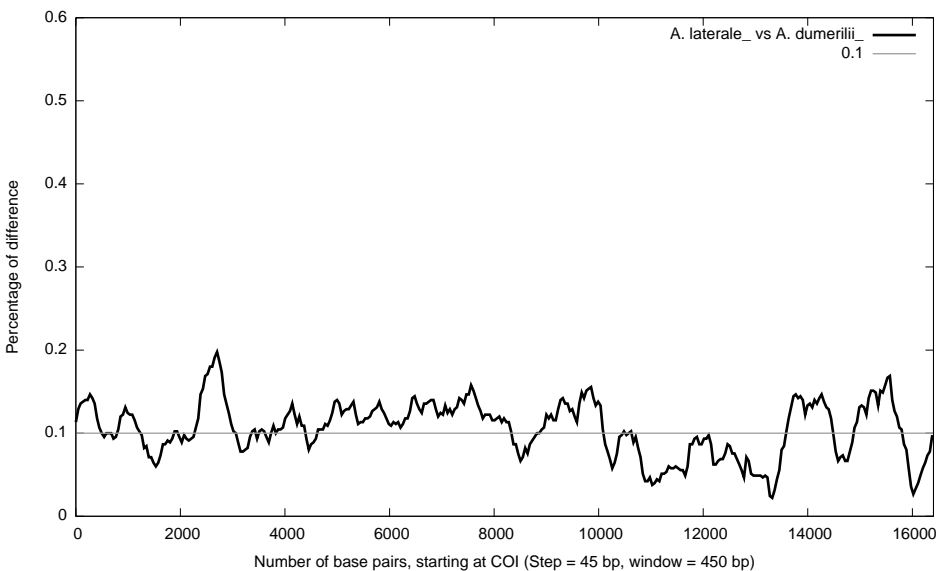

Supplement: File S3 — Sliding window analyses for Mammalia and Lissamphibia. For each family, the folder contains the aligned sequences as well as the sliding window analyses by species pair and for all species pair on a single figure. (ZIP) [file pone.0051263.s003.zip › Lissamphibia/Ambystomatidae/45_450/Ambystoma_laterale_NC_006330_Ambystoma_dumerilii_NC_006889.pdf]

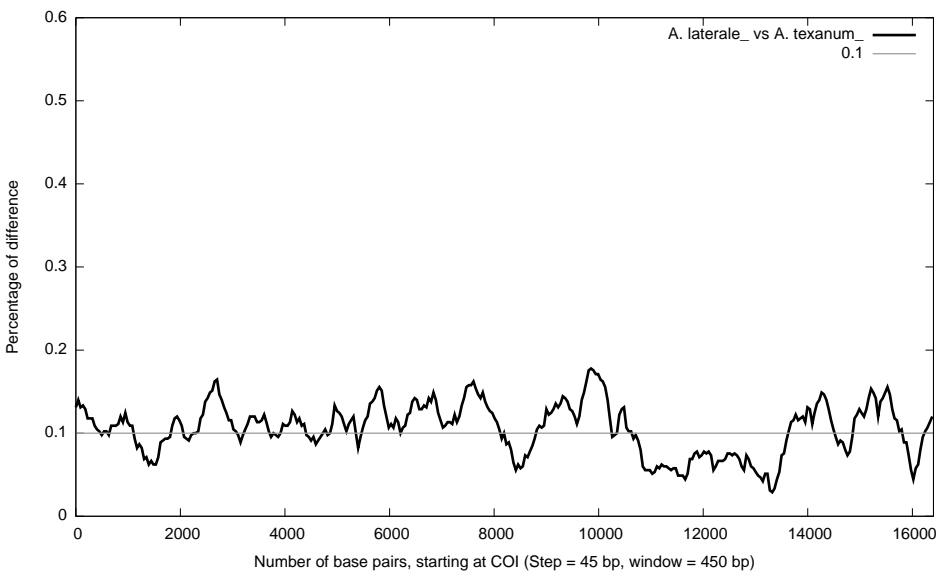

Supplement: File S3 — Sliding window analyses for Mammalia and Lissamphibia. For each family, the folder contains the aligned sequences as well as the sliding window analyses by species pair and for all species pair on a single figure. (ZIP) [file pone.0051263.s003.zip › Lissamphibia/Ambystomatidae/45_450/Ambystoma_laterale_NC_006330_Ambystoma_texanum_NC_014571.pdf]

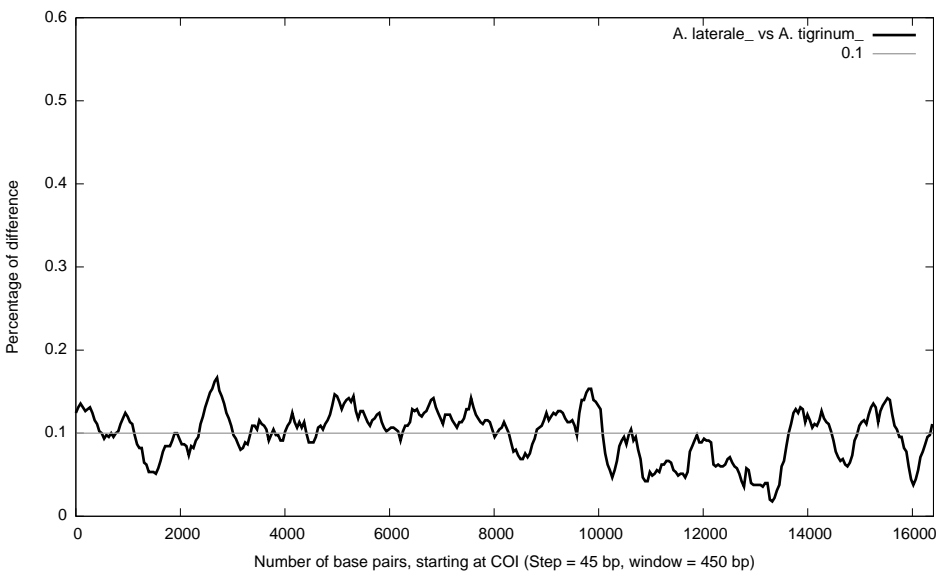

Supplement: File S3 — Sliding window analyses for Mammalia and Lissamphibia. For each family, the folder contains the aligned sequences as well as the sliding window analyses by species pair and for all species pair on a single figure. (ZIP) [file pone.0051263.s003.zip › Lissamphibia/Ambystomatidae/45_450/Ambystoma_laterale_NC_006330_Ambystoma_tigrinum_tigrinum_NC_006887.pdf]

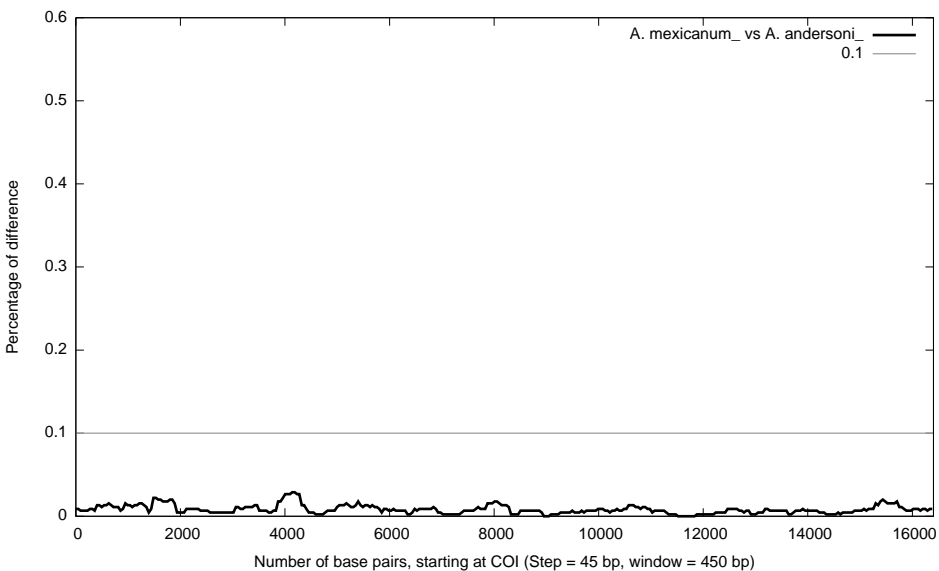

Supplement: File S3 — Sliding window analyses for Mammalia and Lissamphibia. For each family, the folder contains the aligned sequences as well as the sliding window analyses by species pair and for all species pair on a single figure. (ZIP) [file pone.0051263.s003.zip › Lissamphibia/Ambystomatidae/45_450/Ambystoma_mexicanum_NC_005797_Ambystoma_andersoni_NC_006888.pdf]

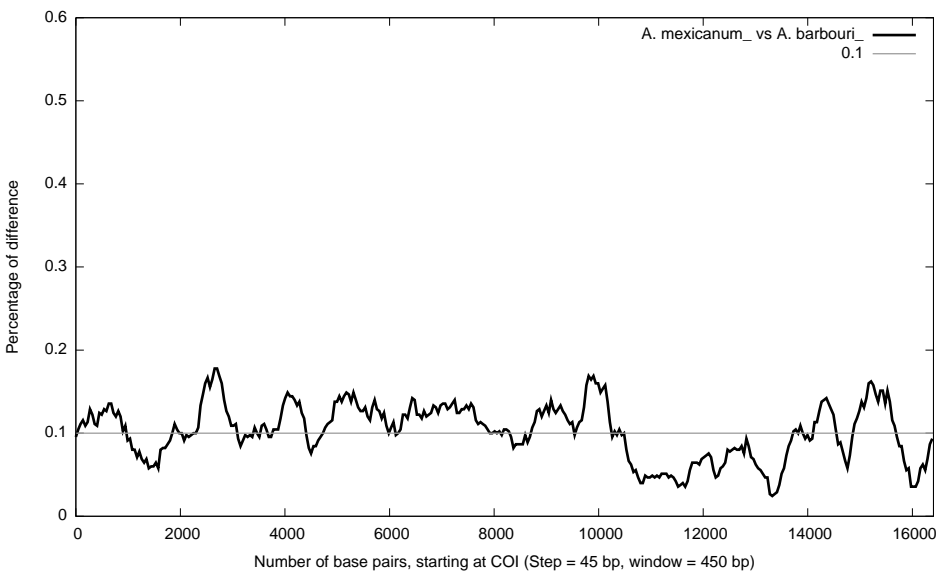

Supplement: File S3 — Sliding window analyses for Mammalia and Lissamphibia. For each family, the folder contains the aligned sequences as well as the sliding window analyses by species pair and for all species pair on a single figure. (ZIP) [file pone.0051263.s003.zip › Lissamphibia/Ambystomatidae/45_450/Ambystoma_mexicanum_NC_005797_Ambystoma_barbouri_NC_014568.pdf]

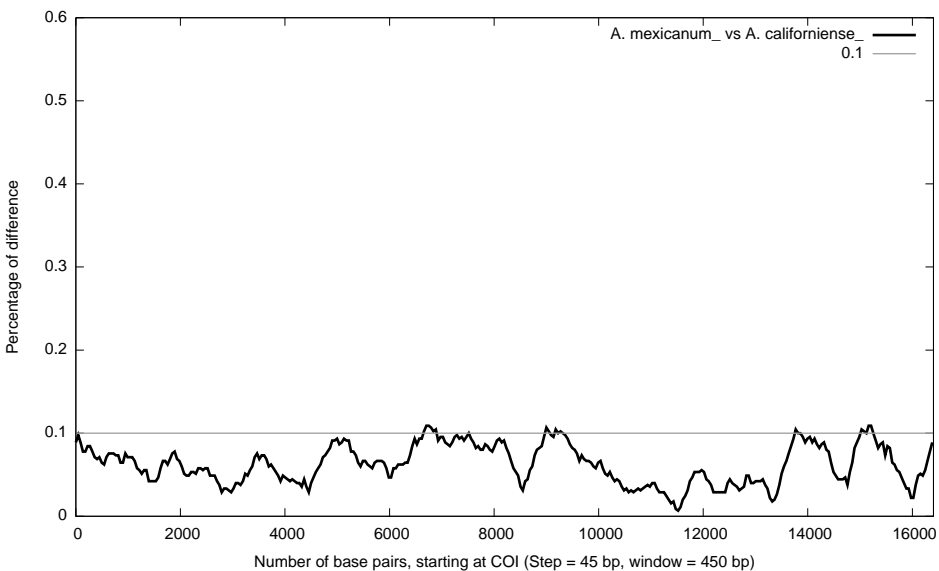

Supplement: File S3 — Sliding window analyses for Mammalia and Lissamphibia. For each family, the folder contains the aligned sequences as well as the sliding window analyses by species pair and for all species pair on a single figure. (ZIP) [file pone.0051263.s003.zip › Lissamphibia/Ambystomatidae/45_450/Ambystoma_mexicanum_NC_005797_Ambystoma_californiense_NC_006890.pdf]

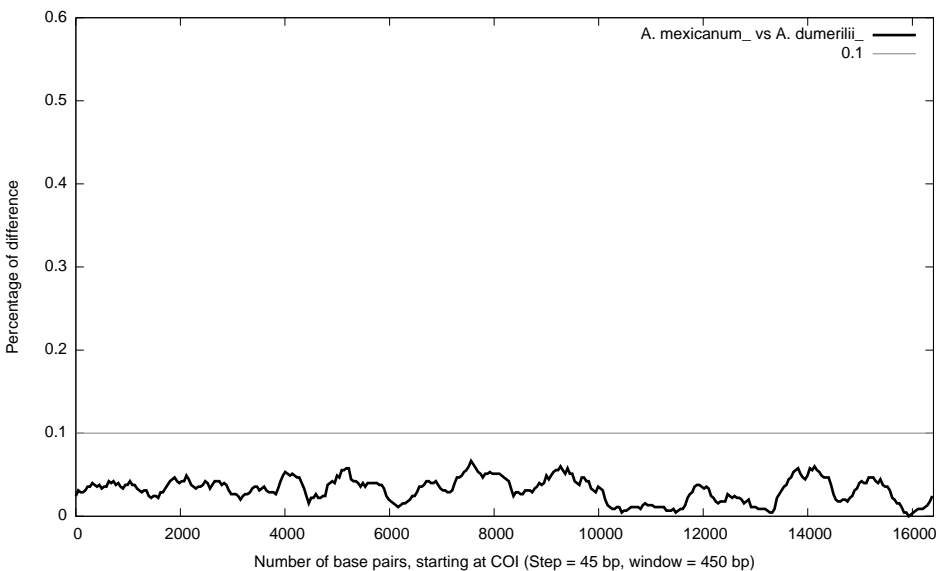

Supplement: File S3 — Sliding window analyses for Mammalia and Lissamphibia. For each family, the folder contains the aligned sequences as well as the sliding window analyses by species pair and for all species pair on a single figure. (ZIP) [file pone.0051263.s003.zip › Lissamphibia/Ambystomatidae/45_450/Ambystoma_mexicanum_NC_005797_Ambystoma_dumerilii_NC_006889.pdf]

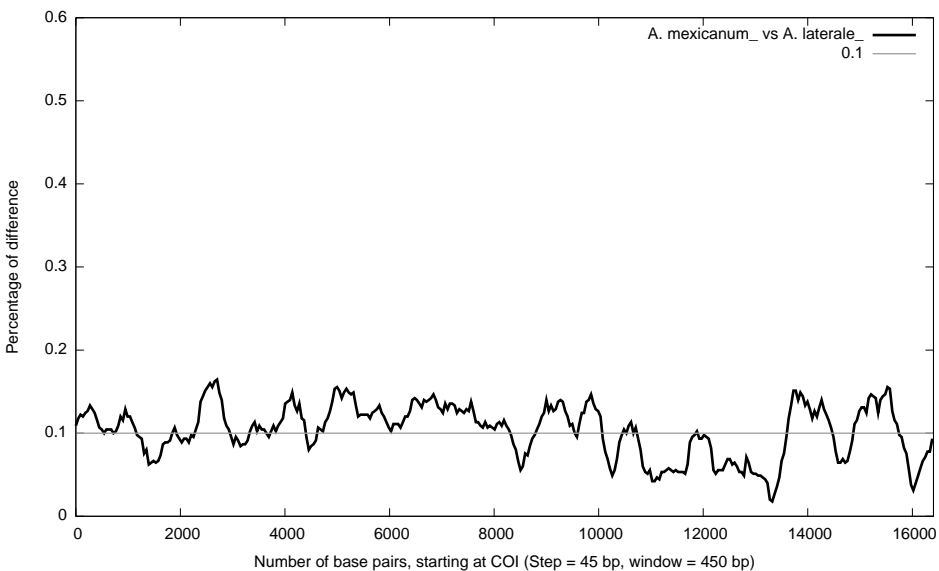

Supplement: File S3 — Sliding window analyses for Mammalia and Lissamphibia. For each family, the folder contains the aligned sequences as well as the sliding window analyses by species pair and for all species pair on a single figure. (ZIP) [file pone.0051263.s003.zip › Lissamphibia/Ambystomatidae/45_450/Ambystoma_mexicanum_NC_005797_Ambystoma_laterale_NC_006330.pdf]

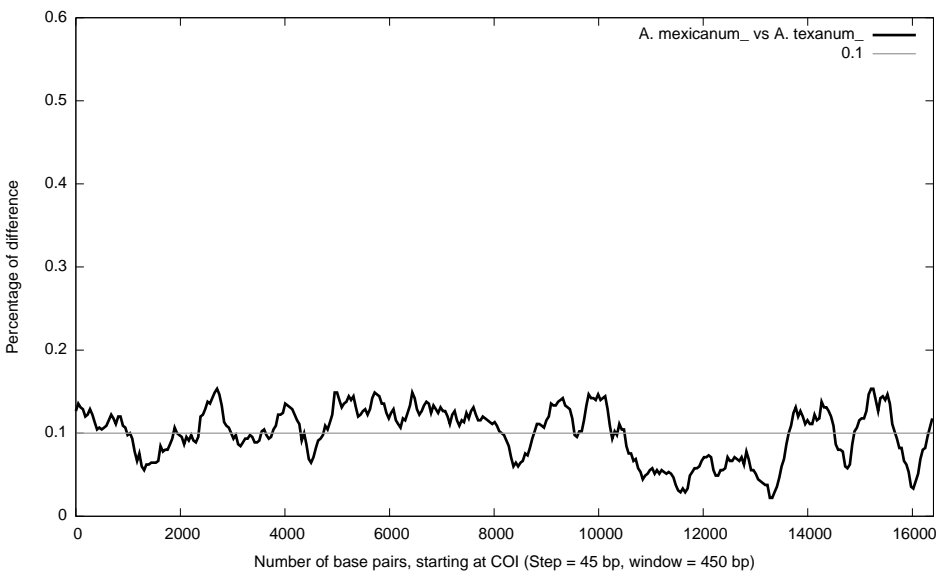

Supplement: File S3 — Sliding window analyses for Mammalia and Lissamphibia. For each family, the folder contains the aligned sequences as well as the sliding window analyses by species pair and for all species pair on a single figure. (ZIP) [file pone.0051263.s003.zip › Lissamphibia/Ambystomatidae/45_450/Ambystoma_mexicanum_NC_005797_Ambystoma_texanum_NC_014571.pdf]

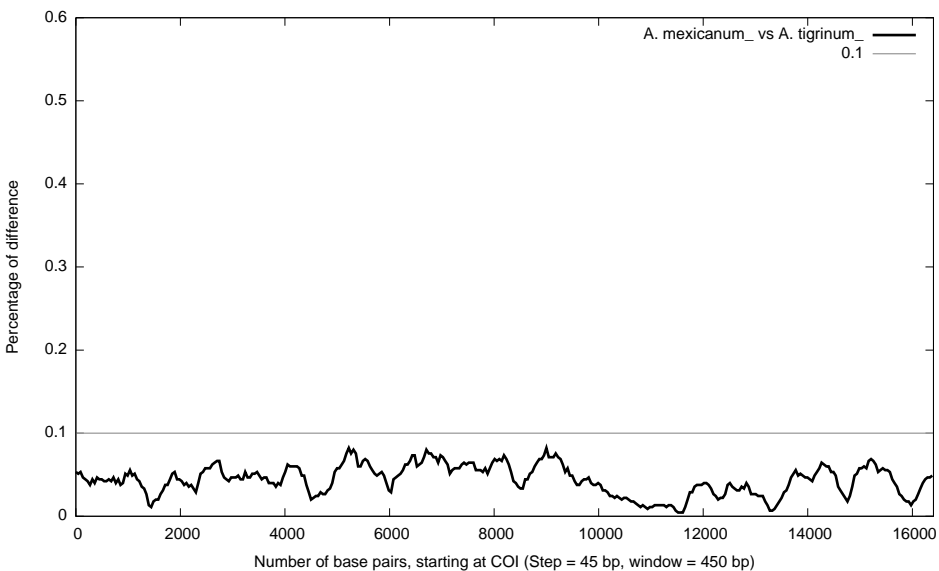

Supplement: File S3 — Sliding window analyses for Mammalia and Lissamphibia. For each family, the folder contains the aligned sequences as well as the sliding window analyses by species pair and for all species pair on a single figure. (ZIP) [file pone.0051263.s003.zip › Lissamphibia/Ambystomatidae/45_450/Ambystoma_mexicanum_NC_005797_Ambystoma_tigrinum_tigrinum_NC_006887.pdf]

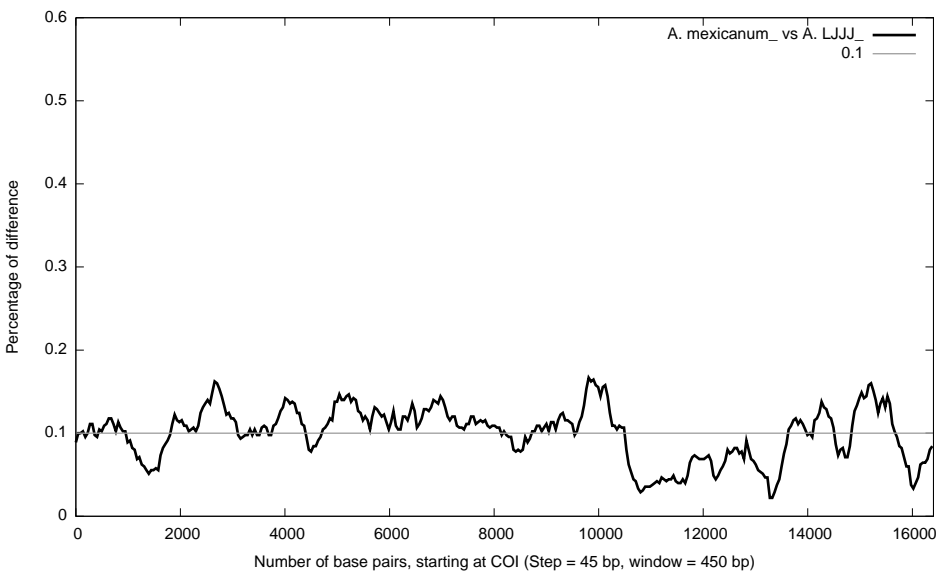

Supplement: File S3 — Sliding window analyses for Mammalia and Lissamphibia. For each family, the folder contains the aligned sequences as well as the sliding window analyses by species pair and for all species pair on a single figure. (ZIP) [file pone.0051263.s003.zip › Lissamphibia/Ambystomatidae/45_450/Ambystoma_mexicanum_NC_005797_Ambystoma_unisexual_lineage_LJJJ_NC_014572.pdf]

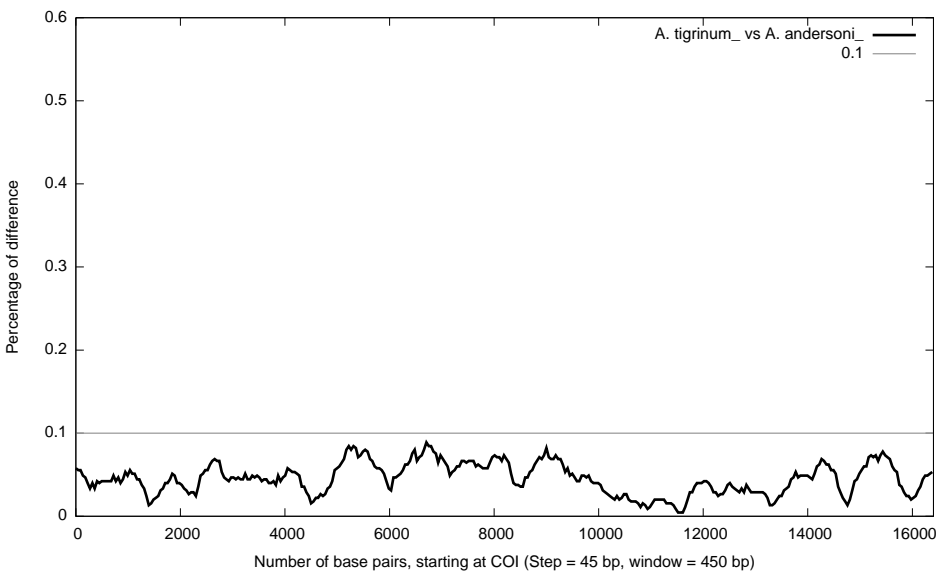

Supplement: File S3 — Sliding window analyses for Mammalia and Lissamphibia. For each family, the folder contains the aligned sequences as well as the sliding window analyses by species pair and for all species pair on a single figure. (ZIP) [file pone.0051263.s003.zip › Lissamphibia/Ambystomatidae/45_450/Ambystoma_tigrinum_tigrinum_NC_006887_Ambystoma_andersoni_NC_006888.pdf]

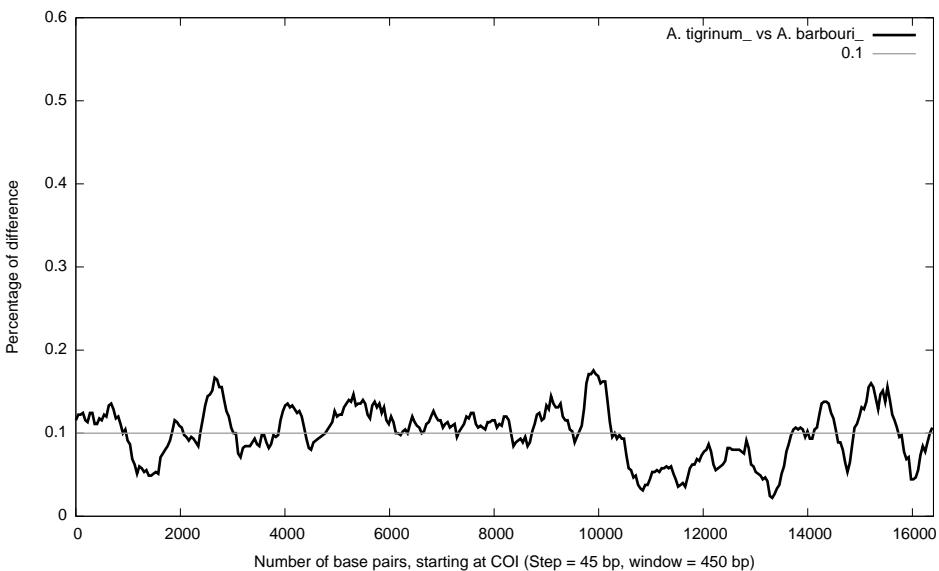

Supplement: File S3 — Sliding window analyses for Mammalia and Lissamphibia. For each family, the folder contains the aligned sequences as well as the sliding window analyses by species pair and for all species pair on a single figure. (ZIP) [file pone.0051263.s003.zip › Lissamphibia/Ambystomatidae/45_450/Ambystoma_tigrinum_tigrinum_NC_006887_Ambystoma_barbouri_NC_014568.pdf]

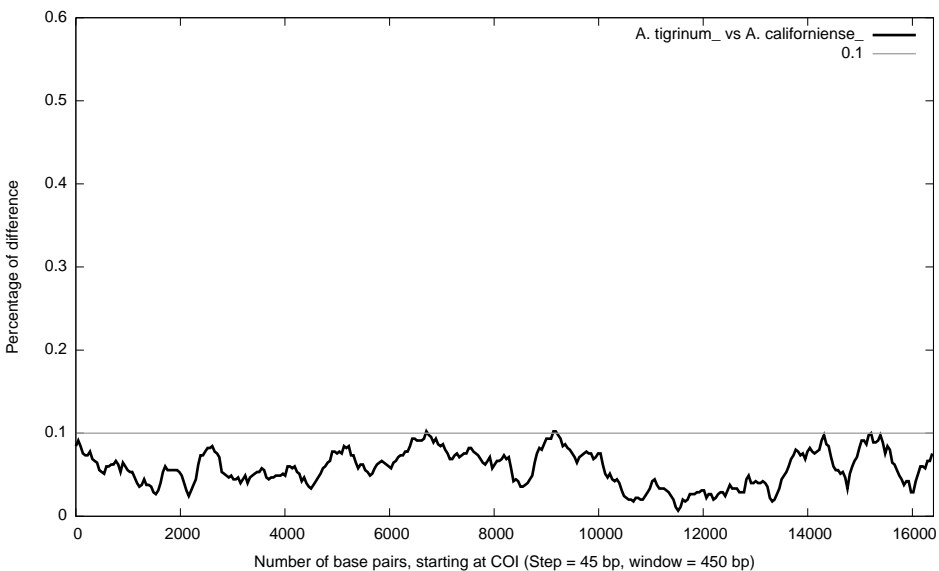

Supplement: File S3 — Sliding window analyses for Mammalia and Lissamphibia. For each family, the folder contains the aligned sequences as well as the sliding window analyses by species pair and for all species pair on a single figure. (ZIP) [file pone.0051263.s003.zip › Lissamphibia/Ambystomatidae/45_450/Ambystoma_tigrinum_tigrinum_NC_006887_Ambystoma_californiense_NC_006890.pdf]

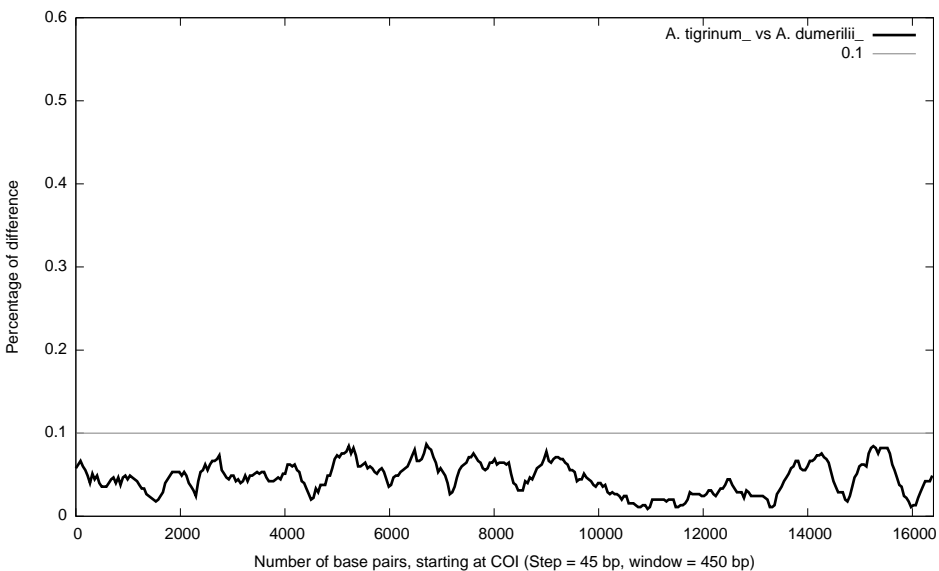

Supplement: File S3 — Sliding window analyses for Mammalia and Lissamphibia. For each family, the folder contains the aligned sequences as well as the sliding window analyses by species pair and for all species pair on a single figure. (ZIP) [file pone.0051263.s003.zip › Lissamphibia/Ambystomatidae/45_450/Ambystoma_tigrinum_tigrinum_NC_006887_Ambystoma_dumerilii_NC_006889.pdf]

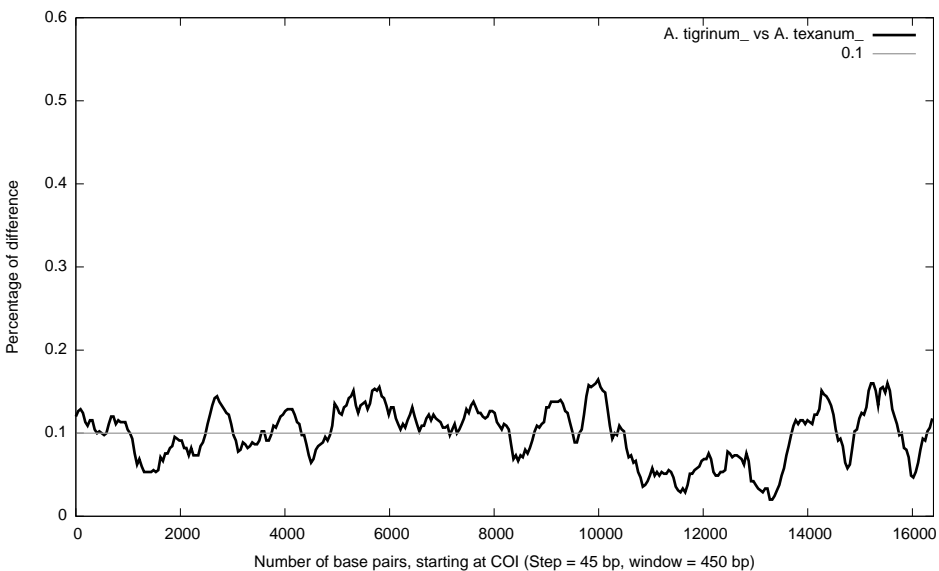

Supplement: File S3 — Sliding window analyses for Mammalia and Lissamphibia. For each family, the folder contains the aligned sequences as well as the sliding window analyses by species pair and for all species pair on a single figure. (ZIP) [file pone.0051263.s003.zip › Lissamphibia/Ambystomatidae/45_450/Ambystoma_tigrinum_tigrinum_NC_006887_Ambystoma_texanum_NC_014571.pdf]

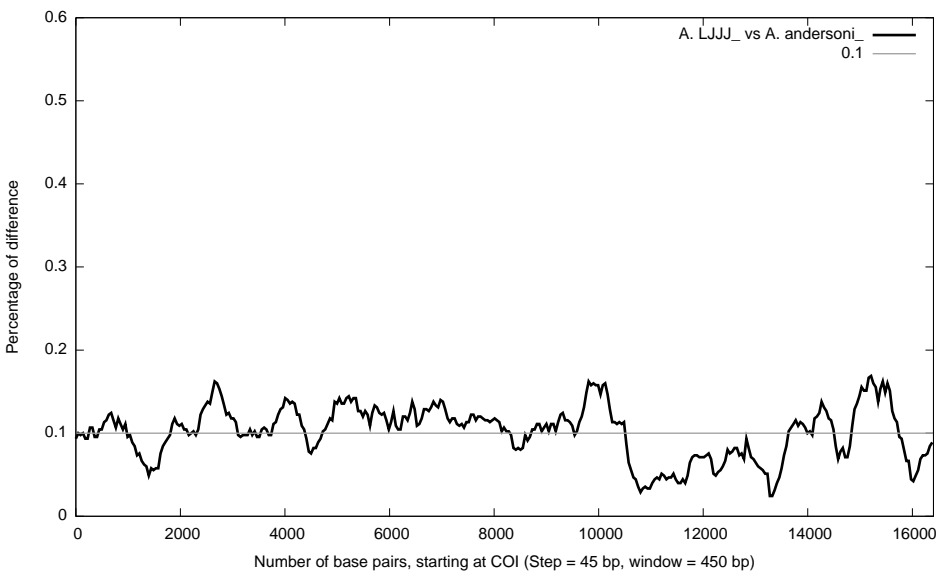

Supplement: File S3 — Sliding window analyses for Mammalia and Lissamphibia. For each family, the folder contains the aligned sequences as well as the sliding window analyses by species pair and for all species pair on a single figure. (ZIP) [file pone.0051263.s003.zip › Lissamphibia/Ambystomatidae/45_450/Ambystoma_unisexual_lineage_LJJJ_NC_014572_Ambystoma_andersoni_NC_006888.pdf]

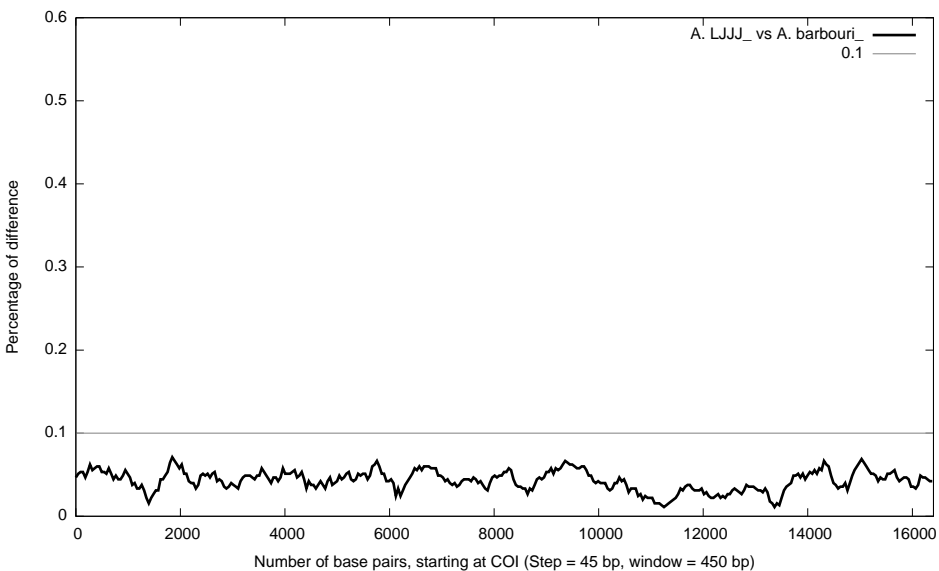

Supplement: File S3 — Sliding window analyses for Mammalia and Lissamphibia. For each family, the folder contains the aligned sequences as well as the sliding window analyses by species pair and for all species pair on a single figure. (ZIP) [file pone.0051263.s003.zip › Lissamphibia/Ambystomatidae/45_450/Ambystoma_unisexual_lineage_LJJJ_NC_014572_Ambystoma_barbouri_NC_014568.pdf]

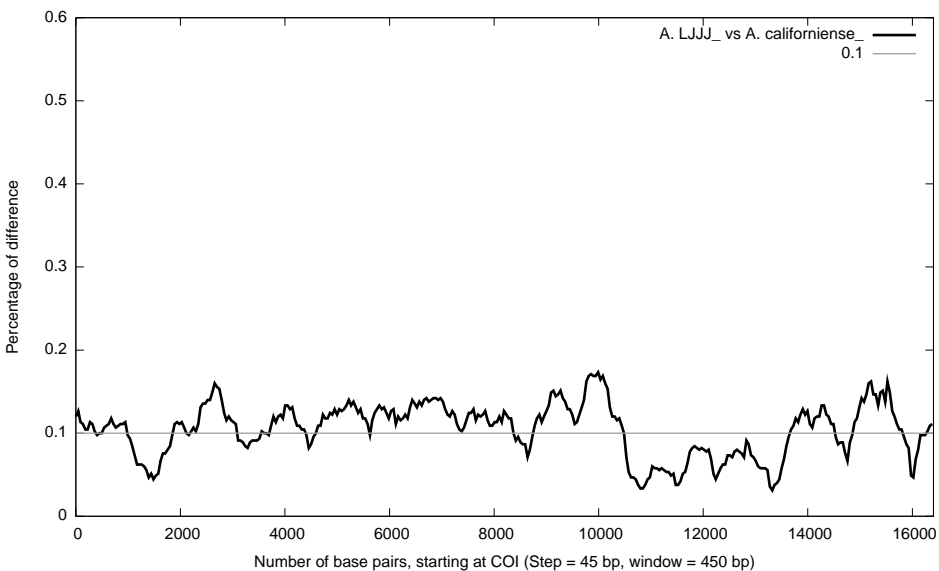

Supplement: File S3 — Sliding window analyses for Mammalia and Lissamphibia. For each family, the folder contains the aligned sequences as well as the sliding window analyses by species pair and for all species pair on a single figure. (ZIP) [file pone.0051263.s003.zip › Lissamphibia/Ambystomatidae/45_450/Ambystoma_unisexual_lineage_LJJJ_NC_014572_Ambystoma_californiense_NC_006890.pdf]

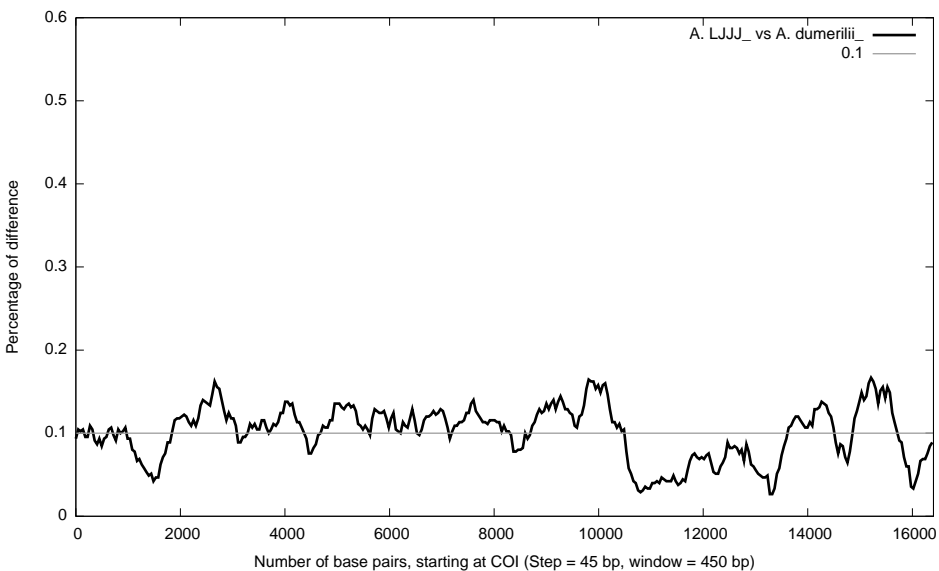

Supplement: File S3 — Sliding window analyses for Mammalia and Lissamphibia. For each family, the folder contains the aligned sequences as well as the sliding window analyses by species pair and for all species pair on a single figure. (ZIP) [file pone.0051263.s003.zip › Lissamphibia/Ambystomatidae/45_450/Ambystoma_unisexual_lineage_LJJJ_NC_014572_Ambystoma_dumerilii_NC_006889.pdf]

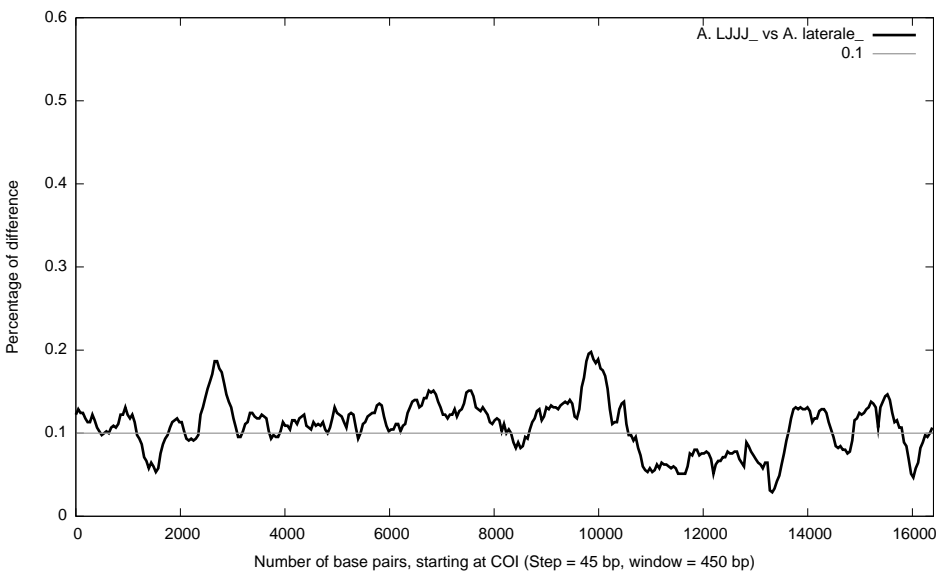

Supplement: File S3 — Sliding window analyses for Mammalia and Lissamphibia. For each family, the folder contains the aligned sequences as well as the sliding window analyses by species pair and for all species pair on a single figure. (ZIP) [file pone.0051263.s003.zip › Lissamphibia/Ambystomatidae/45_450/Ambystoma_unisexual_lineage_LJJJ_NC_014572_Ambystoma_laterale_NC_006330.pdf]

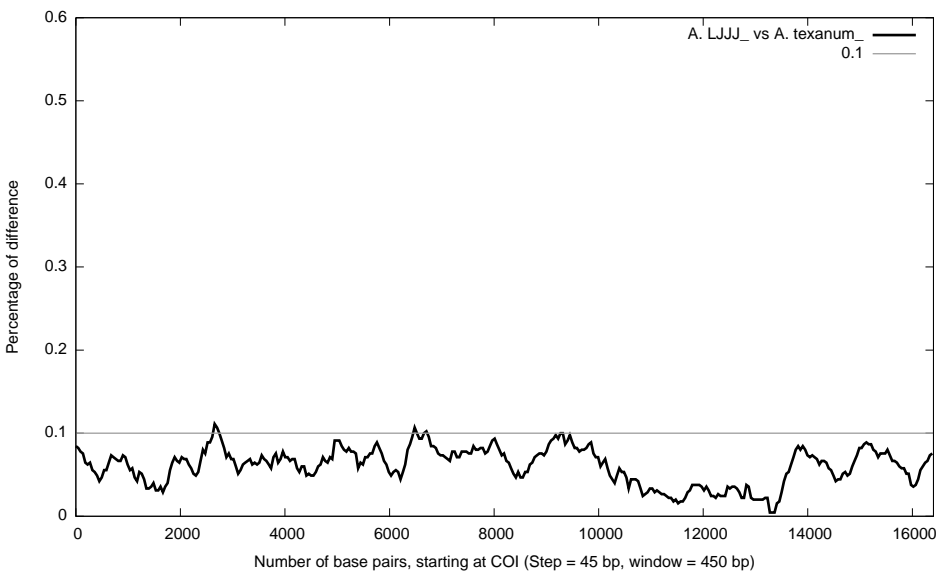

Supplement: File S3 — Sliding window analyses for Mammalia and Lissamphibia. For each family, the folder contains the aligned sequences as well as the sliding window analyses by species pair and for all species pair on a single figure. (ZIP) [file pone.0051263.s003.zip › Lissamphibia/Ambystomatidae/45_450/Ambystoma_unisexual_lineage_LJJJ_NC_014572_Ambystoma_texanum_NC_014571.pdf]

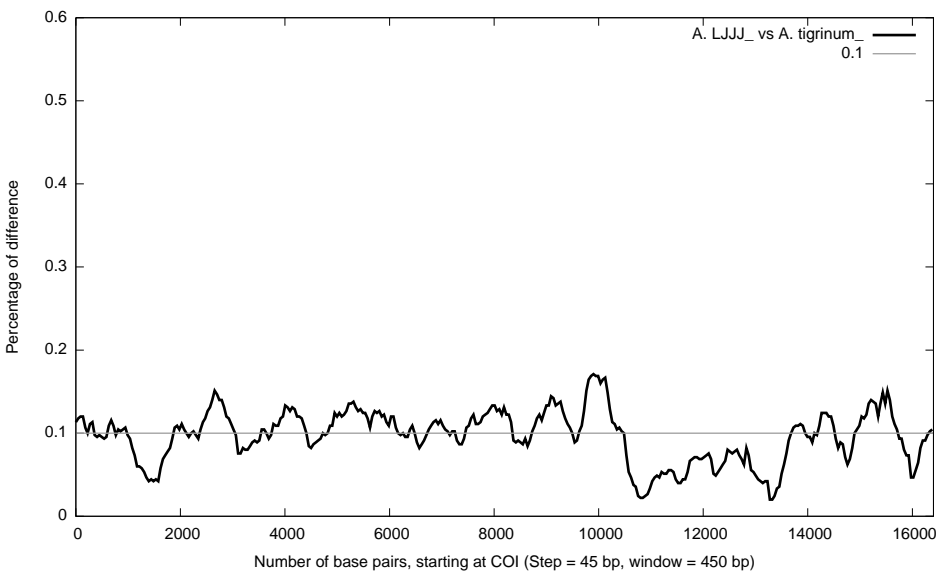

Supplement: File S3 — Sliding window analyses for Mammalia and Lissamphibia. For each family, the folder contains the aligned sequences as well as the sliding window analyses by species pair and for all species pair on a single figure. (ZIP) [file pone.0051263.s003.zip › Lissamphibia/Ambystomatidae/45_450/Ambystoma_unisexual_lineage_LJJJ_NC_014572_Ambystoma_tigrinum_tigrinum_NC_006887.pdf]

# Bombinatoridae

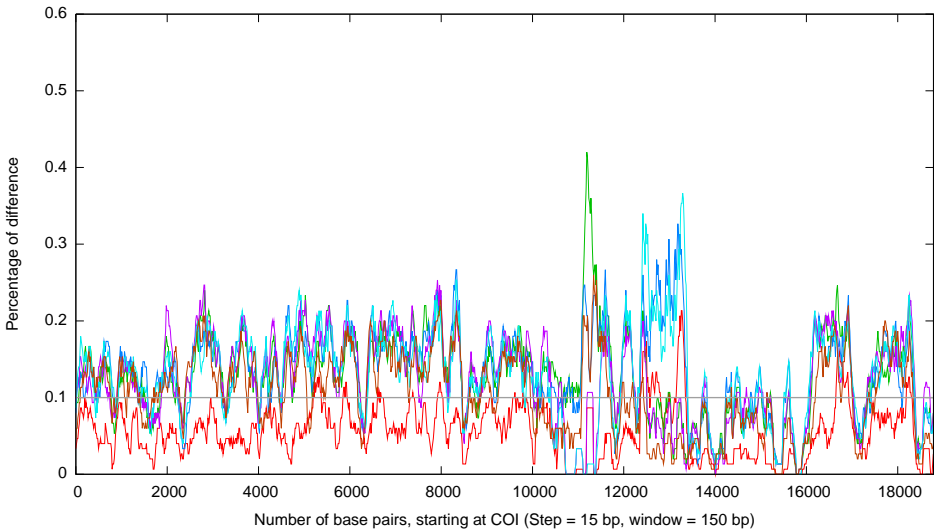

Supplement: File S3 — Sliding window analyses for Mammalia and Lissamphibia. For each family, the folder contains the aligned sequences as well as the sliding window analyses by species pair and for all species pair on a single figure. (ZIP) [file pone.0051263.s003.zip › Lissamphibia/Bombinatoridae/15_150/allCurves.pdf]

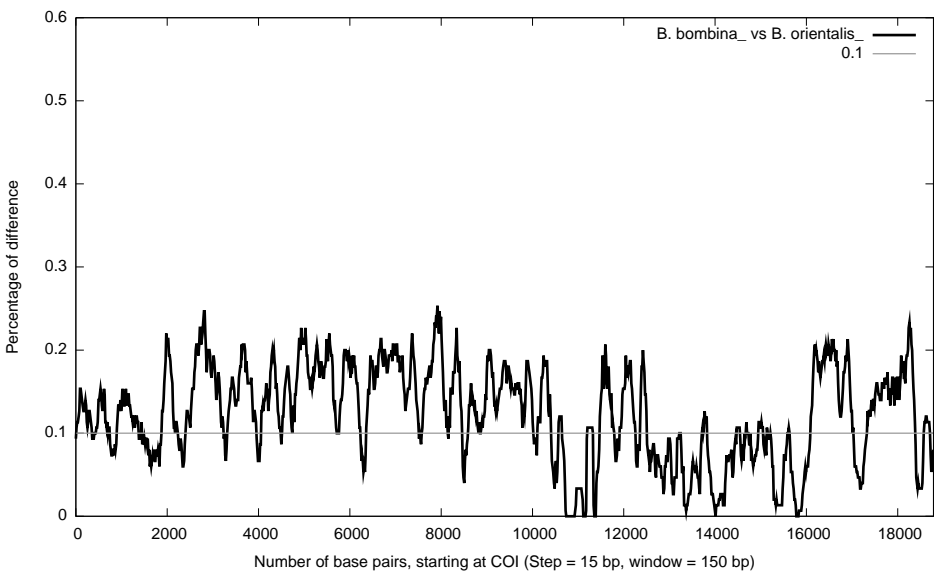

Supplement: File S3 — Sliding window analyses for Mammalia and Lissamphibia. For each family, the folder contains the aligned sequences as well as the sliding window analyses by species pair and for all species pair on a single figure. (ZIP) [file pone.0051263.s003.zip › Lissamphibia/Bombinatoridae/15_150/Bombina_bombina_NC_006402_Bombina_orientalis_NC_006689.pdf]

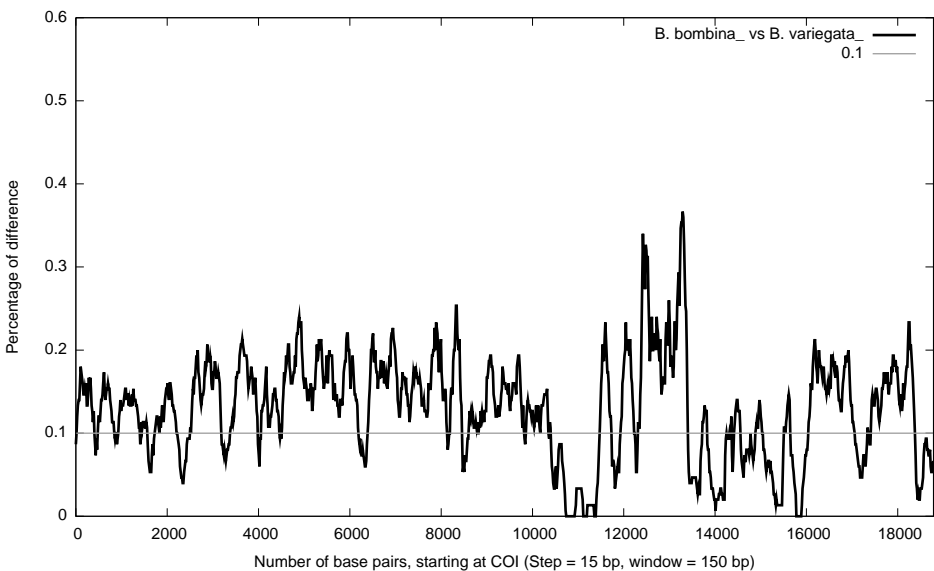

Supplement: File S3 — Sliding window analyses for Mammalia and Lissamphibia. For each family, the folder contains the aligned sequences as well as the sliding window analyses by species pair and for all species pair on a single figure. (ZIP) [file pone.0051263.s003.zip › Lissamphibia/Bombinatoridae/15_150/Bombina_bombina_NC_006402_Bombina_variegata_NC_009258.pdf]

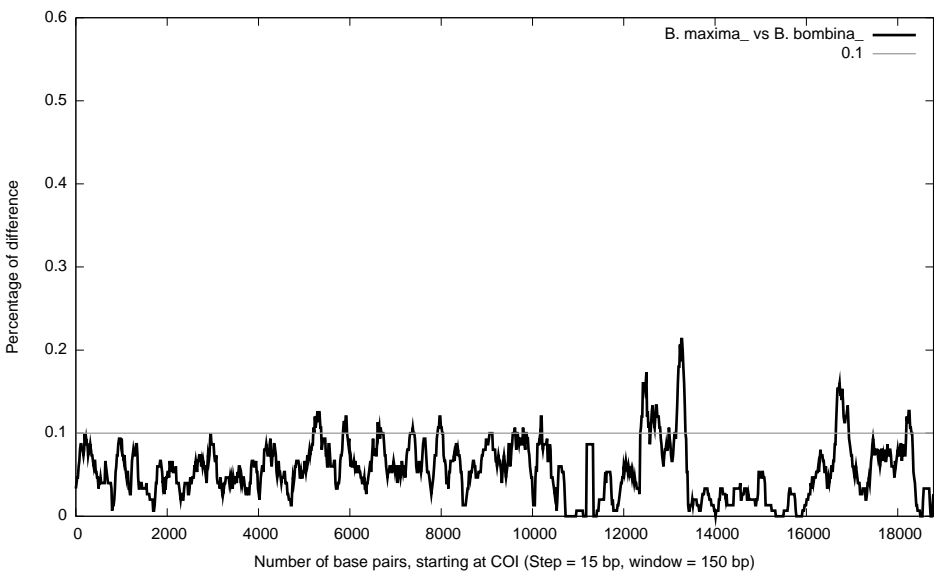

Supplement: File S3 — Sliding window analyses for Mammalia and Lissamphibia. For each family, the folder contains the aligned sequences as well as the sliding window analyses by species pair and for all species pair on a single figure. (ZIP) [file pone.0051263.s003.zip › Lissamphibia/Bombinatoridae/15_150/Bombina_maxima_NC_011049_Bombina_bombina_NC_006402.pdf]

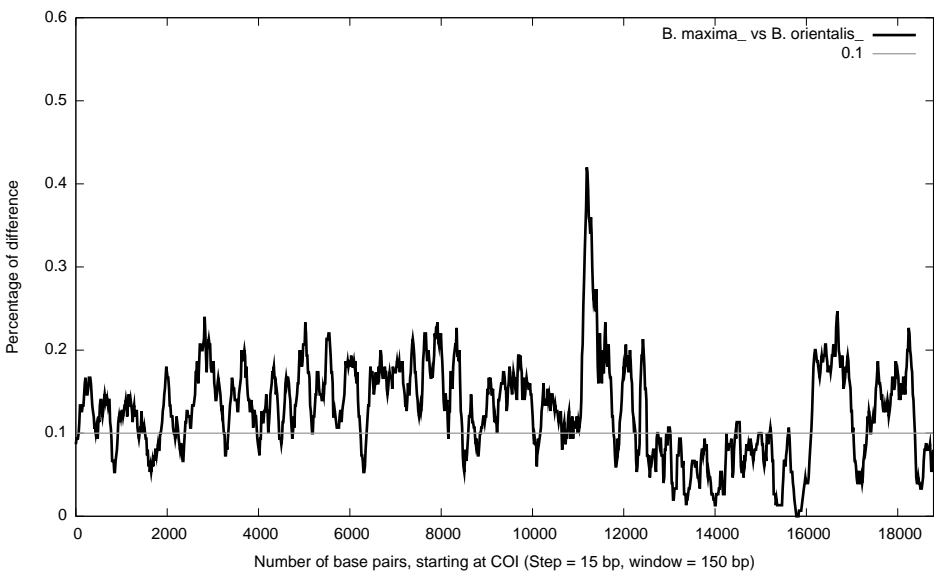

Supplement: File S3 — Sliding window analyses for Mammalia and Lissamphibia. For each family, the folder contains the aligned sequences as well as the sliding window analyses by species pair and for all species pair on a single figure. (ZIP) [file pone.0051263.s003.zip › Lissamphibia/Bombinatoridae/15_150/Bombina_maxima_NC_011049_Bombina_orientalis_NC_006689.pdf]

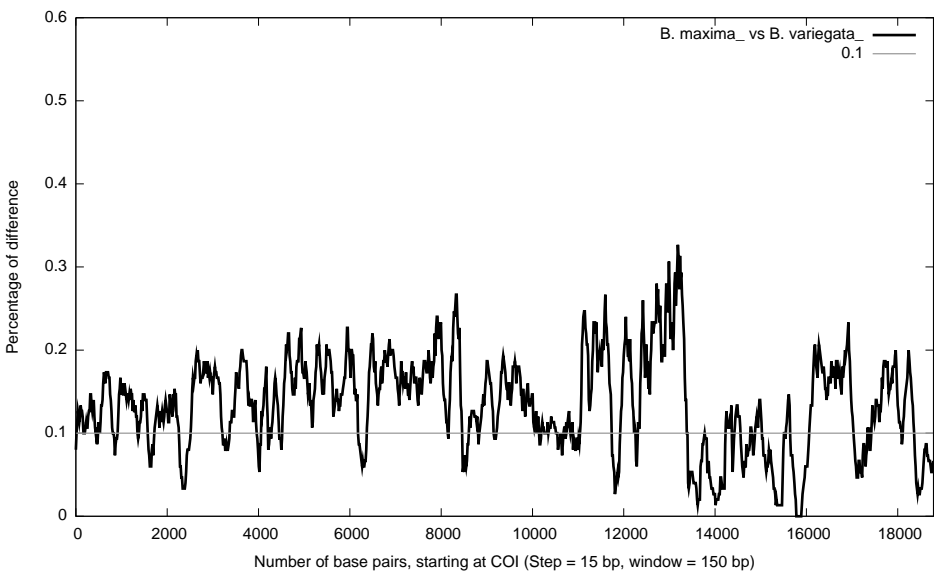

Supplement: File S3 — Sliding window analyses for Mammalia and Lissamphibia. For each family, the folder contains the aligned sequences as well as the sliding window analyses by species pair and for all species pair on a single figure. (ZIP) [file pone.0051263.s003.zip › Lissamphibia/Bombinatoridae/15_150/Bombina_maxima_NC_011049_Bombina_variegata_NC_009258.pdf]

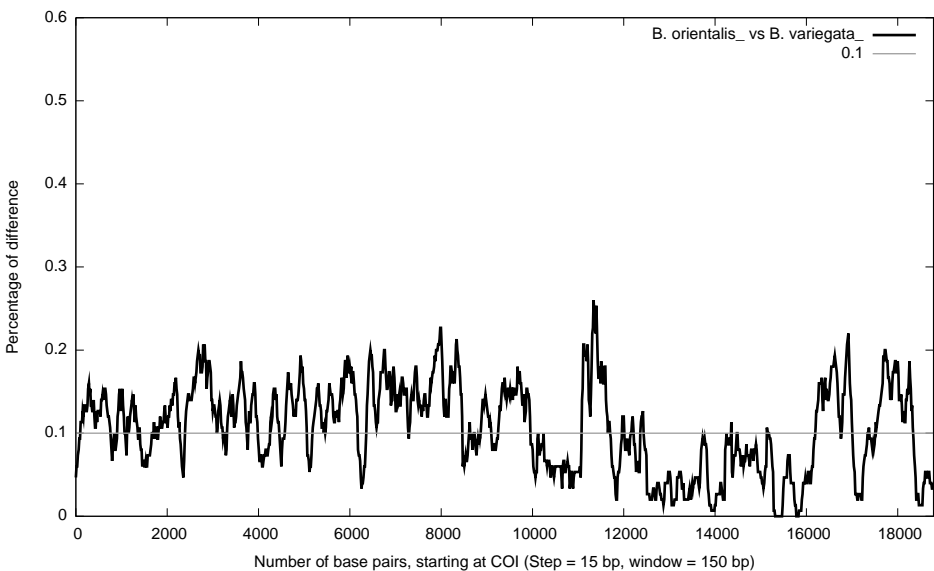

Supplement: File S3 — Sliding window analyses for Mammalia and Lissamphibia. For each family, the folder contains the aligned sequences as well as the sliding window analyses by species pair and for all species pair on a single figure. (ZIP) [file pone.0051263.s003.zip › Lissamphibia/Bombinatoridae/15_150/Bombina_orientalis_NC_006689_Bombina_variegata_NC_009258.pdf]

# Bombinatoridae

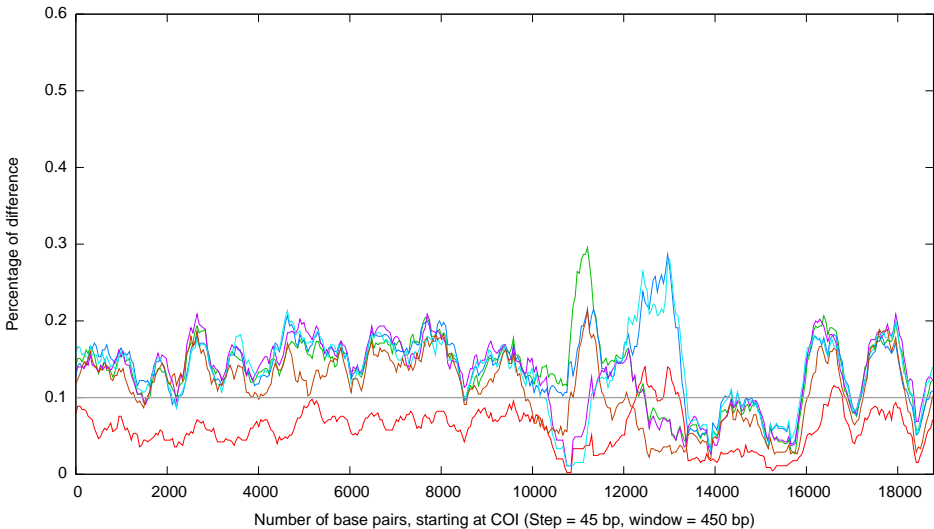

Supplement: File S3 — Sliding window analyses for Mammalia and Lissamphibia. For each family, the folder contains the aligned sequences as well as the sliding window analyses by species pair and for all species pair on a single figure. (ZIP) [file pone.0051263.s003.zip › Lissamphibia/Bombinatoridae/45_450/allCurves.pdf]

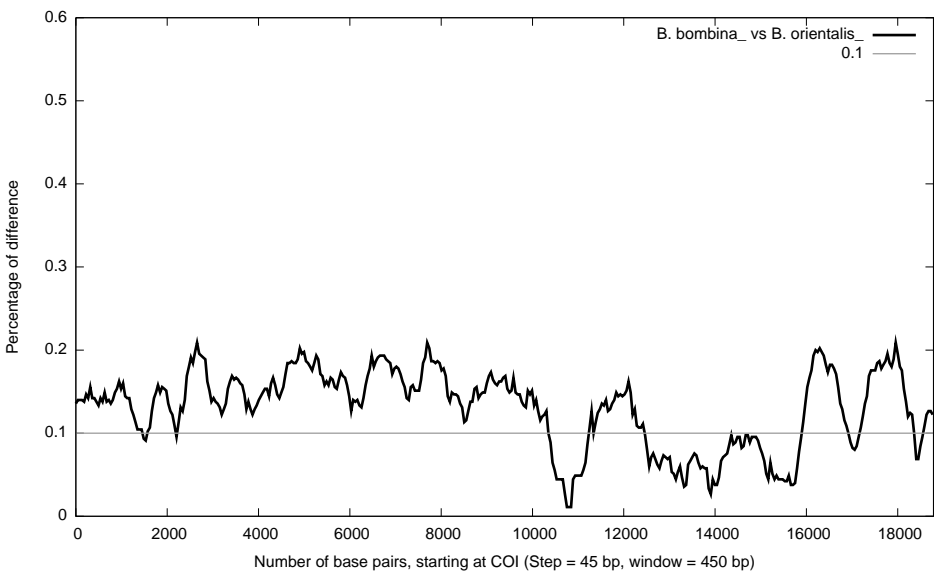

Supplement: File S3 — Sliding window analyses for Mammalia and Lissamphibia. For each family, the folder contains the aligned sequences as well as the sliding window analyses by species pair and for all species pair on a single figure. (ZIP) [file pone.0051263.s003.zip › Lissamphibia/Bombinatoridae/45_450/Bombina_bombina_NC_006402_Bombina_orientalis_NC_006689.pdf]

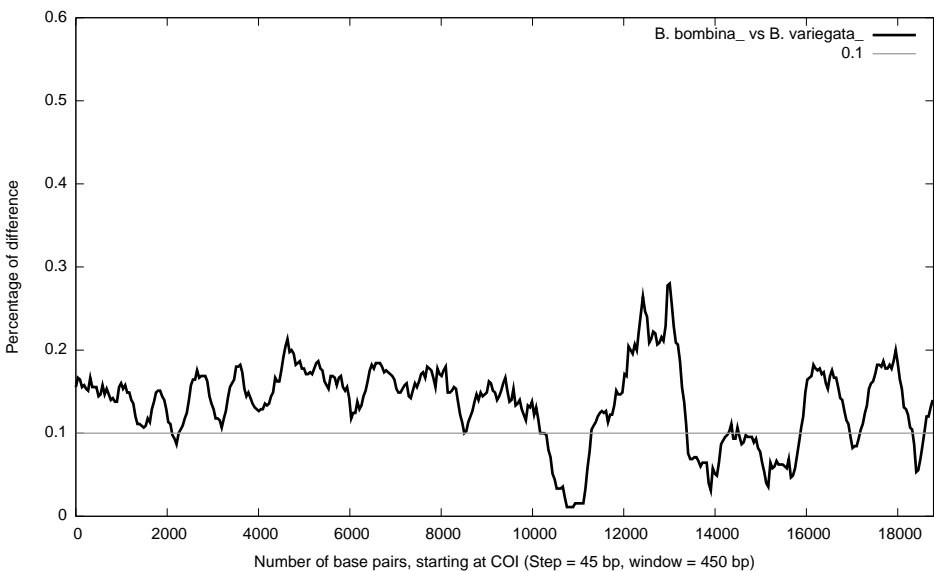

Supplement: File S3 — Sliding window analyses for Mammalia and Lissamphibia. For each family, the folder contains the aligned sequences as well as the sliding window analyses by species pair and for all species pair on a single figure. (ZIP) [file pone.0051263.s003.zip › Lissamphibia/Bombinatoridae/45_450/Bombina_bombina_NC_006402_Bombina_variegata_NC_009258.pdf]

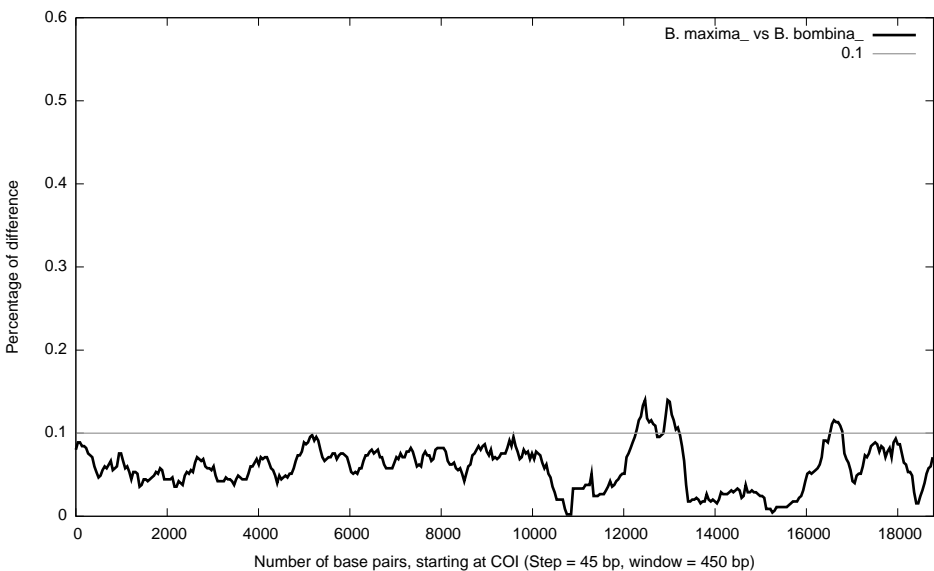

Supplement: File S3 — Sliding window analyses for Mammalia and Lissamphibia. For each family, the folder contains the aligned sequences as well as the sliding window analyses by species pair and for all species pair on a single figure. (ZIP) [file pone.0051263.s003.zip › Lissamphibia/Bombinatoridae/45_450/Bombina_maxima_NC_011049_Bombina_bombina_NC_006402.pdf]

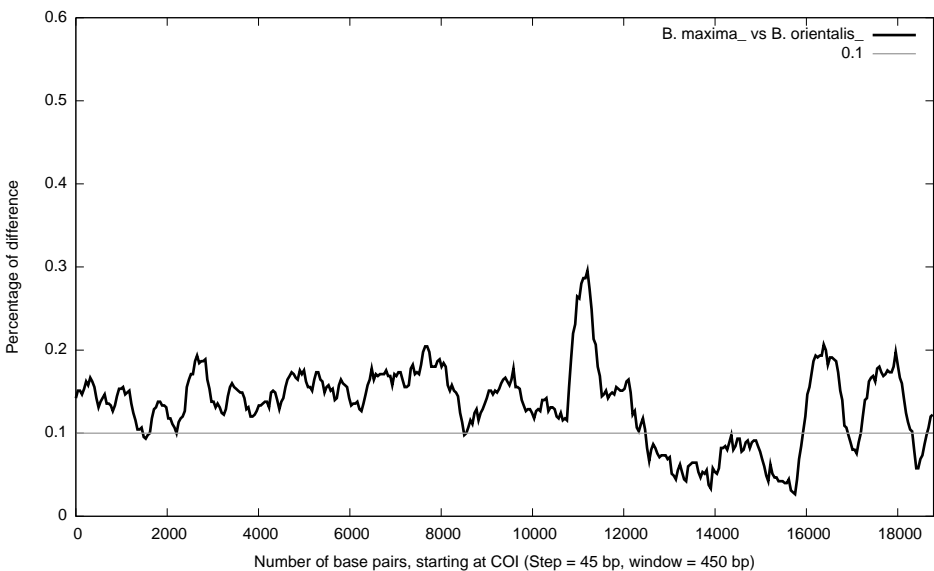

Supplement: File S3 — Sliding window analyses for Mammalia and Lissamphibia. For each family, the folder contains the aligned sequences as well as the sliding window analyses by species pair and for all species pair on a single figure. (ZIP) [file pone.0051263.s003.zip › Lissamphibia/Bombinatoridae/45_450/Bombina_maxima_NC_011049_Bombina_orientalis_NC_006689.pdf]

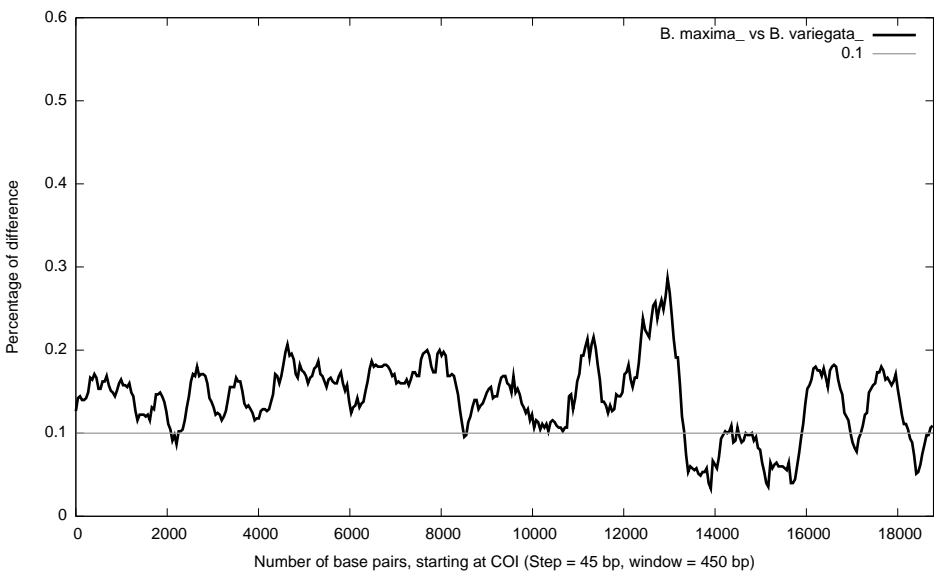

Supplement: File S3 — Sliding window analyses for Mammalia and Lissamphibia. For each family, the folder contains the aligned sequences as well as the sliding window analyses by species pair and for all species pair on a single figure. (ZIP) [file pone.0051263.s003.zip › Lissamphibia/Bombinatoridae/45_450/Bombina_maxima_NC_011049_Bombina_variegata_NC_009258.pdf]

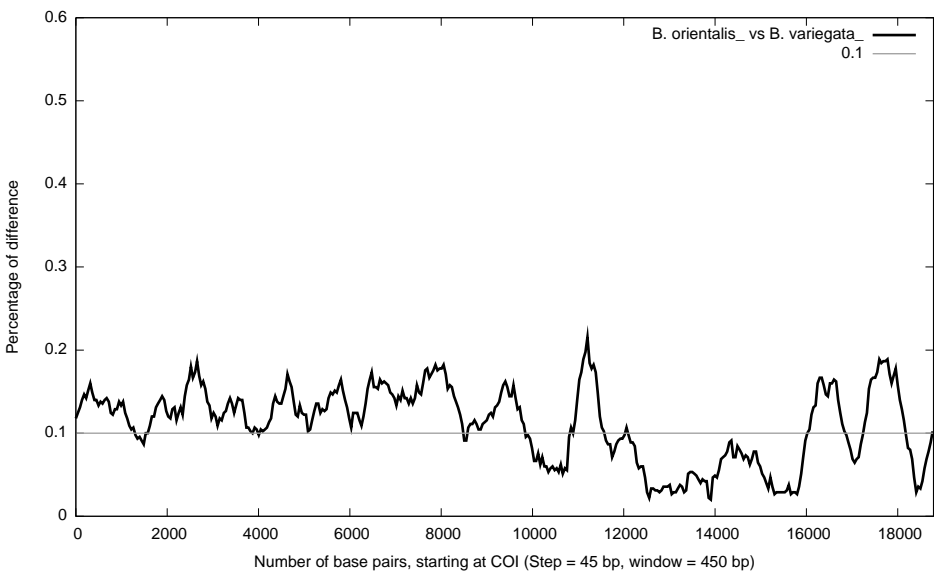

Supplement: File S3 — Sliding window analyses for Mammalia and Lissamphibia. For each family, the folder contains the aligned sequences as well as the sliding window analyses by species pair and for all species pair on a single figure. (ZIP) [file pone.0051263.s003.zip › Lissamphibia/Bombinatoridae/45_450/Bombina_orientalis_NC_006689_Bombina_variegata_NC_009258.pdf]

# Bufo

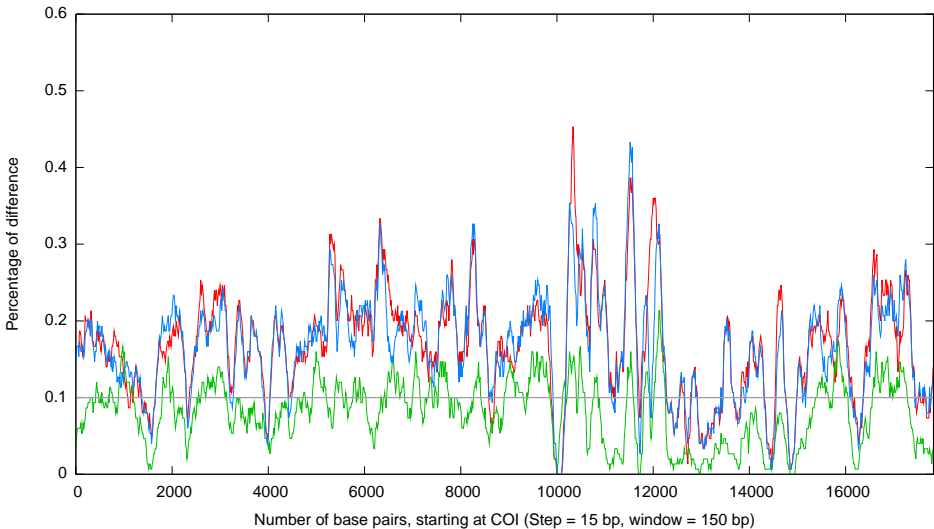

Supplement: File S3 — Sliding window analyses for Mammalia and Lissamphibia. For each family, the folder contains the aligned sequences as well as the sliding window analyses by species pair and for all species pair on a single figure. (ZIP) [file pone.0051263.s003.zip › Lissamphibia/Bufonidae/15_150/allCurves.pdf]

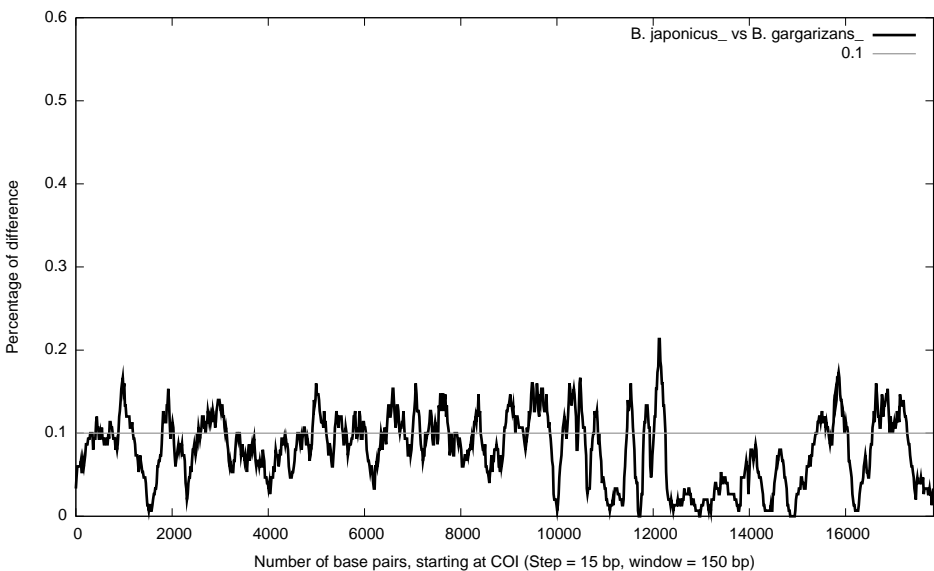

Supplement: File S3 — Sliding window analyses for Mammalia and Lissamphibia. For each family, the folder contains the aligned sequences as well as the sliding window analyses by species pair and for all species pair on a single figure. (ZIP) [file pone.0051263.s003.zip › Lissamphibia/Bufonidae/15_150/Bufo_japonicus_NC_009886_Bufo_gargarizans_NC_008410.pdf]

# Branchiostomidae

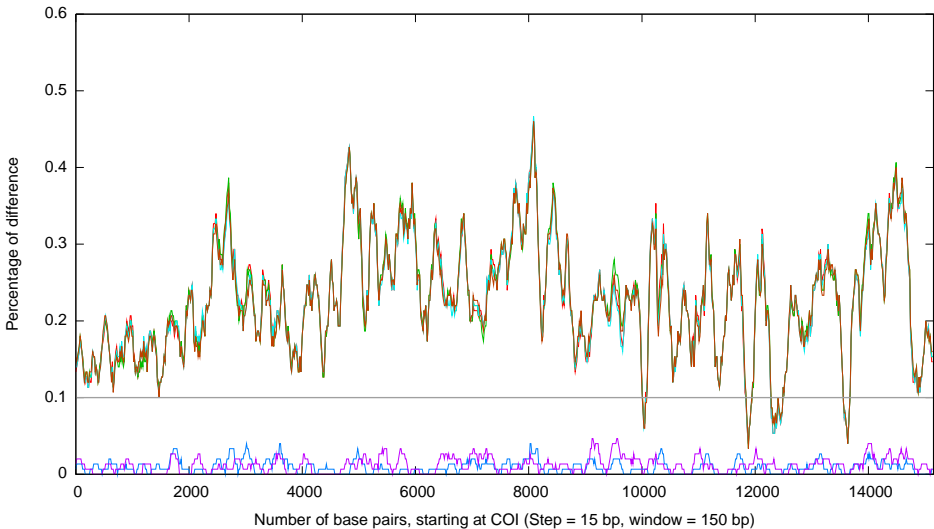

Supplement: File S4 — Sliding window analyses for Sauropsida, Aves, Hemichordata, Coelacanthimorpha, Dipnoi, Chondrichthyes and Cephalochordata. For each family, the folder contains the aligned sequences as well as the sliding window analyses by species pair and for all species pair on a single figure. (ZIP) [file pone.0051263.s004.zip › Cephalocordata/Branchiostomidae/15_150/allCurves.pdf]

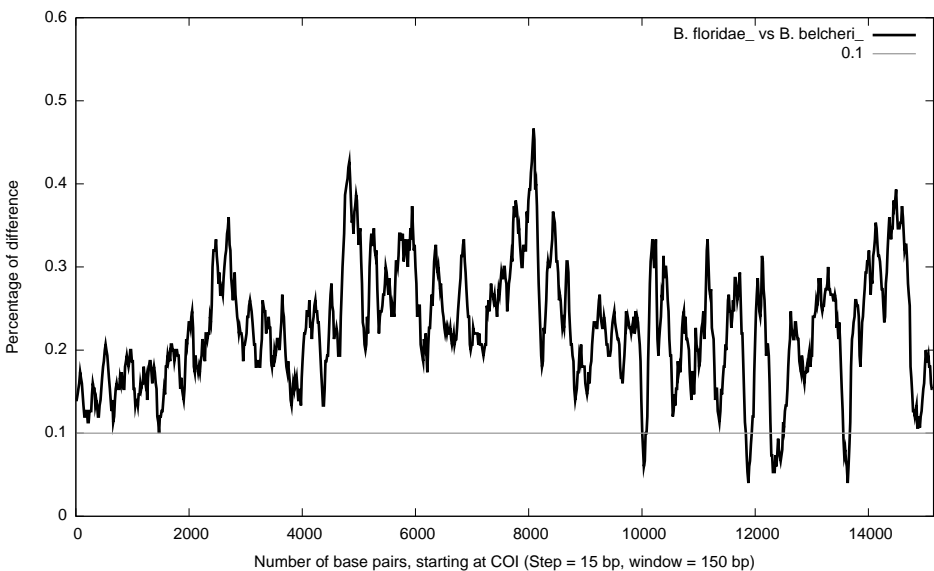

Supplement: File S4 — Sliding window analyses for Sauropsida, Aves, Hemichordata, Coelacanthimorpha, Dipnoi, Chondrichthyes and Cephalochordata. For each family, the folder contains the aligned sequences as well as the sliding window analyses by species pair and for all species pair on a single figure. (ZIP) [file pone.0051263.s004.zip › Cephalocordata/Branchiostomidae/15_150/Branchiostoma_floridae_NC_000834_Branchiostoma_belcheri_NC_004537.pdf]

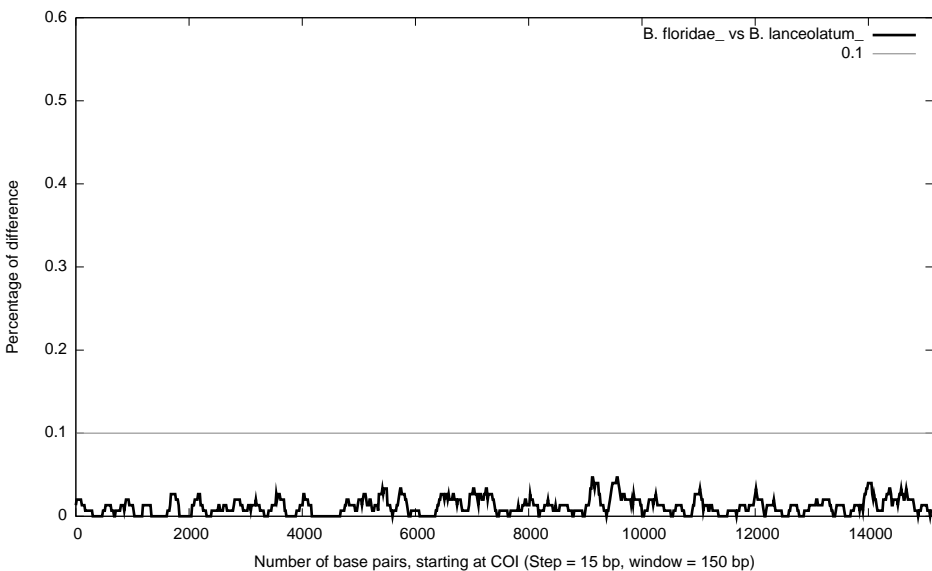

Supplement: File S4 — Sliding window analyses for Sauropsida, Aves, Hemichordata, Coelacanthimorpha, Dipnoi, Chondrichthyes and Cephalochordata. For each family, the folder contains the aligned sequences as well as the sliding window analyses by species pair and for all species pair on a single figure. (ZIP) [file pone.0051263.s004.zip › Cephalocordata/Branchiostomidae/15_150/Branchiostoma_floridae_NC_000834_Branchiostoma_lanceolatum_NC_001912.pdf]

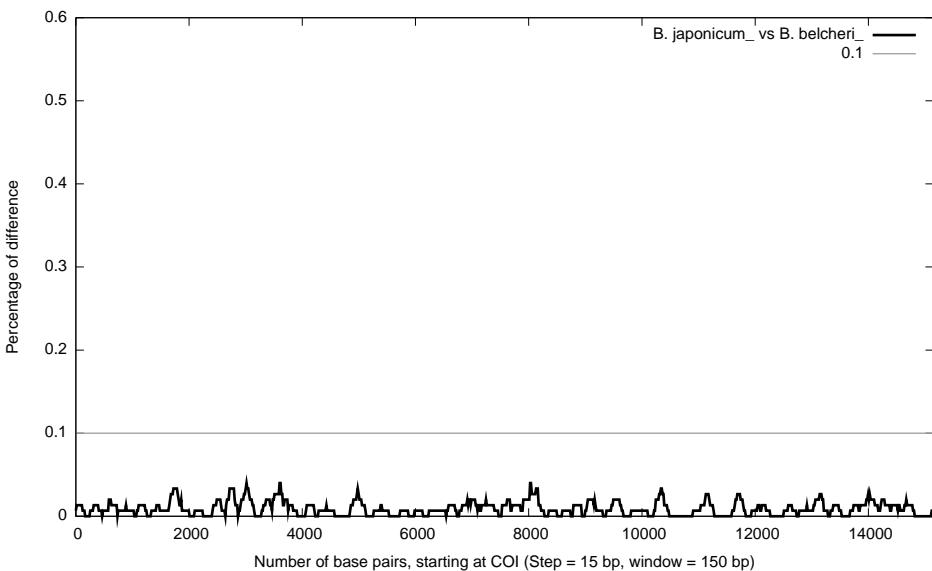

Supplement: File S4 — Sliding window analyses for Sauropsida, Aves, Hemichordata, Coelacanthimorpha, Dipnoi, Chondrichthyes and Cephalochordata. For each family, the folder contains the aligned sequences as well as the sliding window analyses by species pair and for all species pair on a single figure. (ZIP) [file pone.0051263.s004.zip › Cephalocordata/Branchiostomidae/15_150/Branchiostoma_japonicum_NC_008069_Branchiostoma_belcheri_NC_004537.pdf]

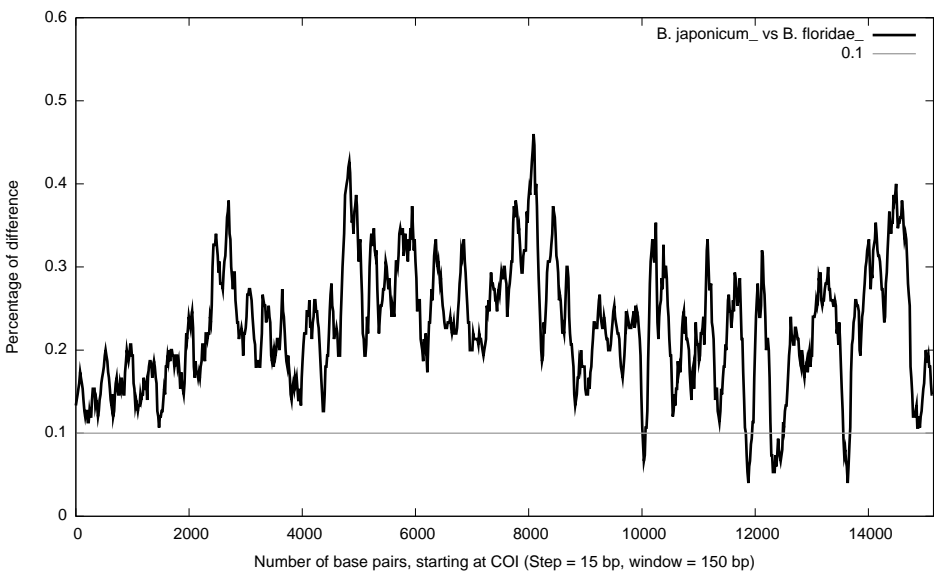

Supplement: File S4 — Sliding window analyses for Sauropsida, Aves, Hemichordata, Coelacanthimorpha, Dipnoi, Chondrichthyes and Cephalochordata. For each family, the folder contains the aligned sequences as well as the sliding window analyses by species pair and for all species pair on a single figure. (ZIP) [file pone.0051263.s004.zip › Cephalocordata/Branchiostomidae/15_150/Branchiostoma_japonicum_NC_008069_Branchiostoma_floridae_NC_000834.pdf]

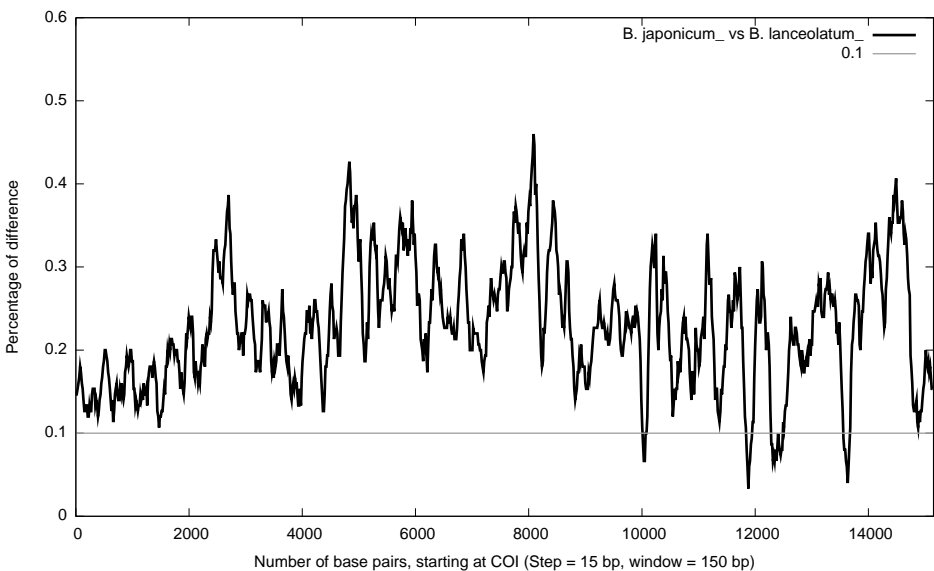

Supplement: File S4 — Sliding window analyses for Sauropsida, Aves, Hemichordata, Coelacanthimorpha, Dipnoi, Chondrichthyes and Cephalochordata. For each family, the folder contains the aligned sequences as well as the sliding window analyses by species pair and for all species pair on a single figure. (ZIP) [file pone.0051263.s004.zip › Cephalocordata/Branchiostomidae/15_150/Branchiostoma_japonicum_NC_008069_Branchiostoma_lanceolatum_NC_001912.pdf]

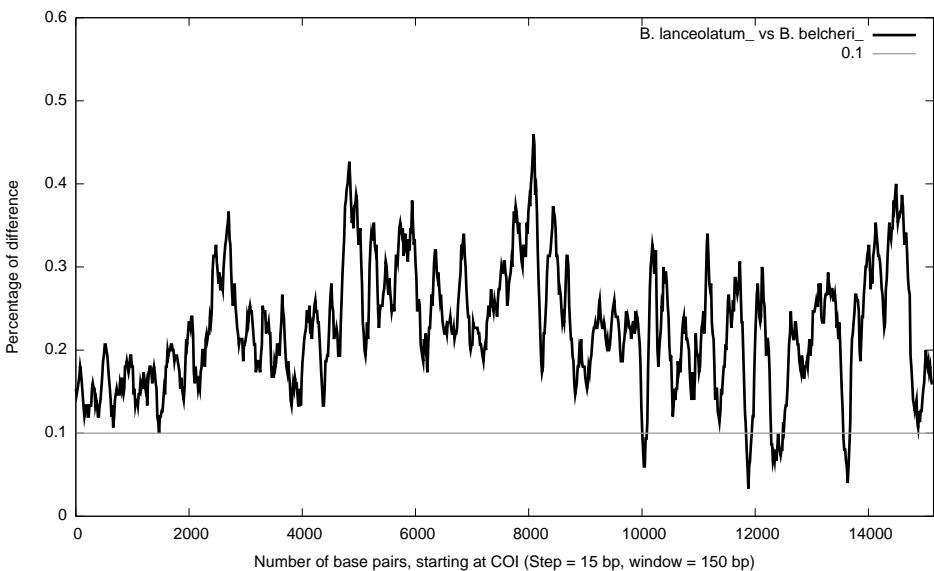

Supplement: File S4 — Sliding window analyses for Sauropsida, Aves, Hemichordata, Coelacanthimorpha, Dipnoi, Chondrichthyes and Cephalochordata. For each family, the folder contains the aligned sequences as well as the sliding window analyses by species pair and for all species pair on a single figure. (ZIP) [file pone.0051263.s004.zip › Cephalocordata/Branchiostomidae/15_150/Branchiostoma_lanceolatum_NC_001912_Branchiostoma_belcheri_NC_004537.pdf]

# Branchiostomidae

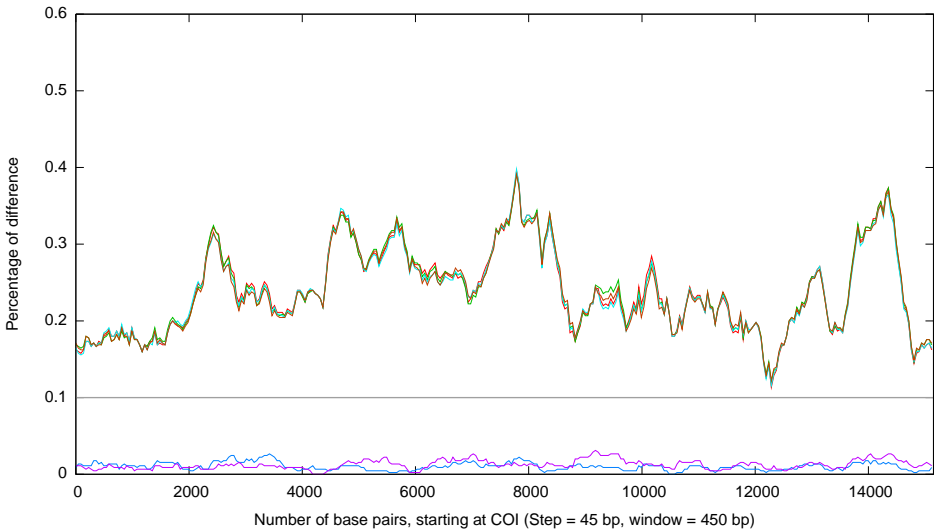

Supplement: File S4 — Sliding window analyses for Sauropsida, Aves, Hemichordata, Coelacanthimorpha, Dipnoi, Chondrichthyes and Cephalochordata. For each family, the folder contains the aligned sequences as well as the sliding window analyses by species pair and for all species pair on a single figure. (ZIP) [file pone.0051263.s004.zip › Cephalocordata/Branchiostomidae/45_450/allCurves.pdf]

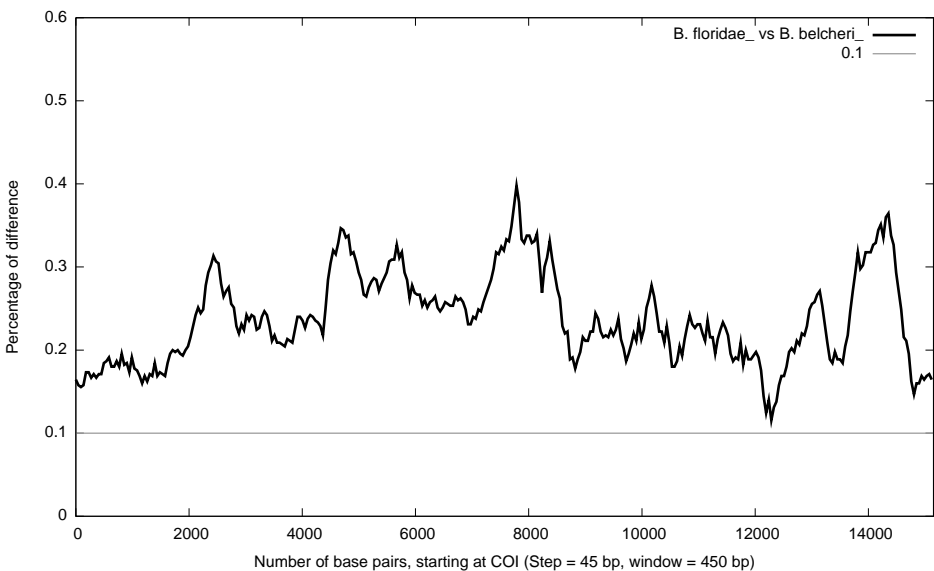

Supplement: File S4 — Sliding window analyses for Sauropsida, Aves, Hemichordata, Coelacanthimorpha, Dipnoi, Chondrichthyes and Cephalochordata. For each family, the folder contains the aligned sequences as well as the sliding window analyses by species pair and for all species pair on a single figure. (ZIP) [file pone.0051263.s004.zip › Cephalocordata/Branchiostomidae/45_450/Branchiostoma_floridae_NC_000834_Branchiostoma_belcheri_NC_004537.pdf]

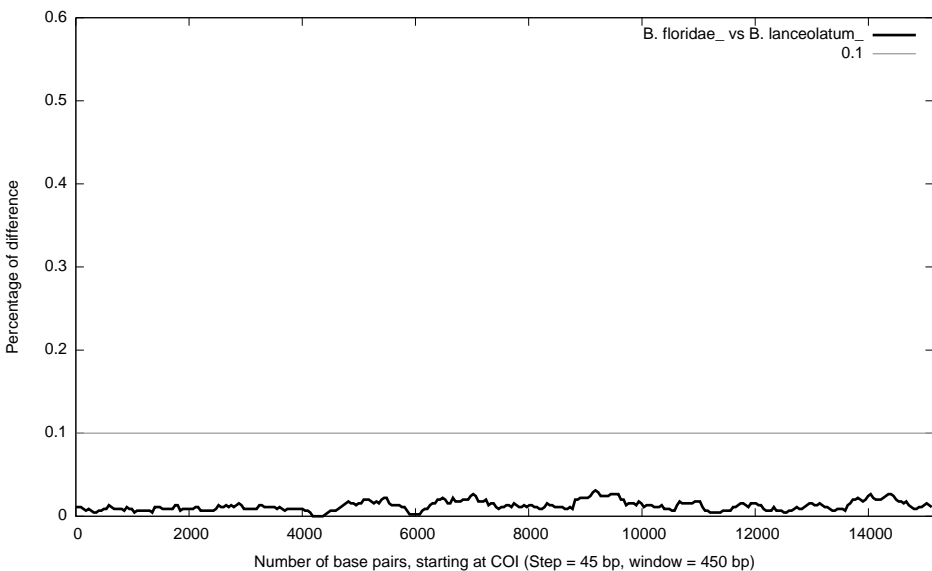

Supplement: File S4 — Sliding window analyses for Sauropsida, Aves, Hemichordata, Coelacanthimorpha, Dipnoi, Chondrichthyes and Cephalochordata. For each family, the folder contains the aligned sequences as well as the sliding window analyses by species pair and for all species pair on a single figure. (ZIP) [file pone.0051263.s004.zip › Cephalocordata/Branchiostomidae/45_450/Branchiostoma_floridae_NC_000834_Branchiostoma_lanceolatum_NC_001912.pdf]

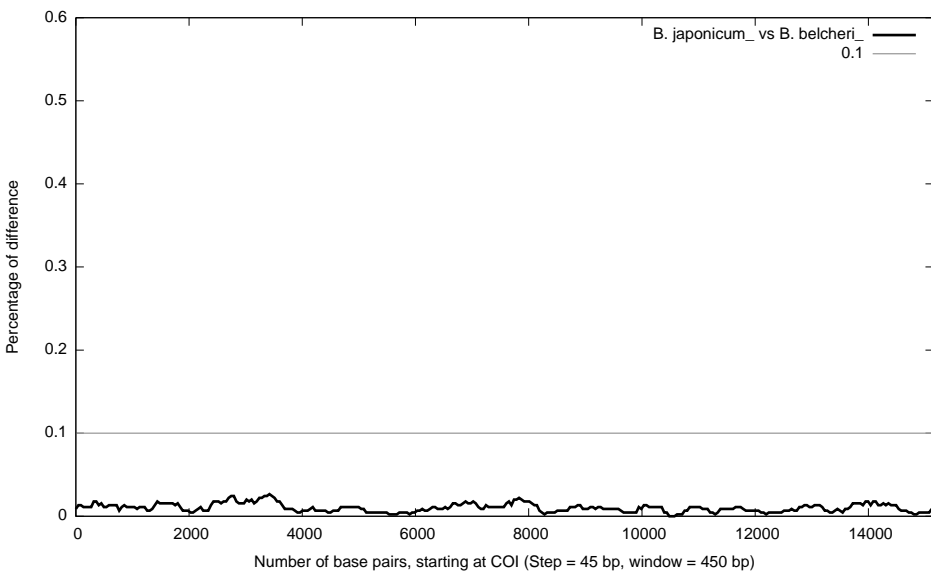

Supplement: File S4 — Sliding window analyses for Sauropsida, Aves, Hemichordata, Coelacanthimorpha, Dipnoi, Chondrichthyes and Cephalochordata. For each family, the folder contains the aligned sequences as well as the sliding window analyses by species pair and for all species pair on a single figure. (ZIP) [file pone.0051263.s004.zip › Cephalocordata/Branchiostomidae/45_450/Branchiostoma_japonicum_NC_008069_Branchiostoma_belcheri_NC_004537.pdf]

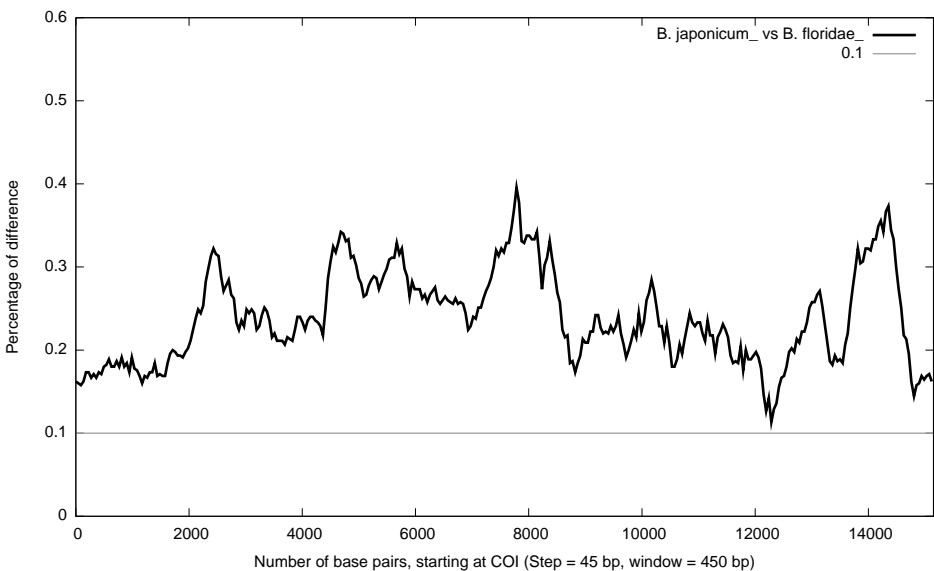

Supplement: File S4 — Sliding window analyses for Sauropsida, Aves, Hemichordata, Coelacanthimorpha, Dipnoi, Chondrichthyes and Cephalochordata. For each family, the folder contains the aligned sequences as well as the sliding window analyses by species pair and for all species pair on a single figure. (ZIP) [file pone.0051263.s004.zip › Cephalocordata/Branchiostomidae/45_450/Branchiostoma_japonicum_NC_008069_Branchiostoma_floridae_NC_000834.pdf]

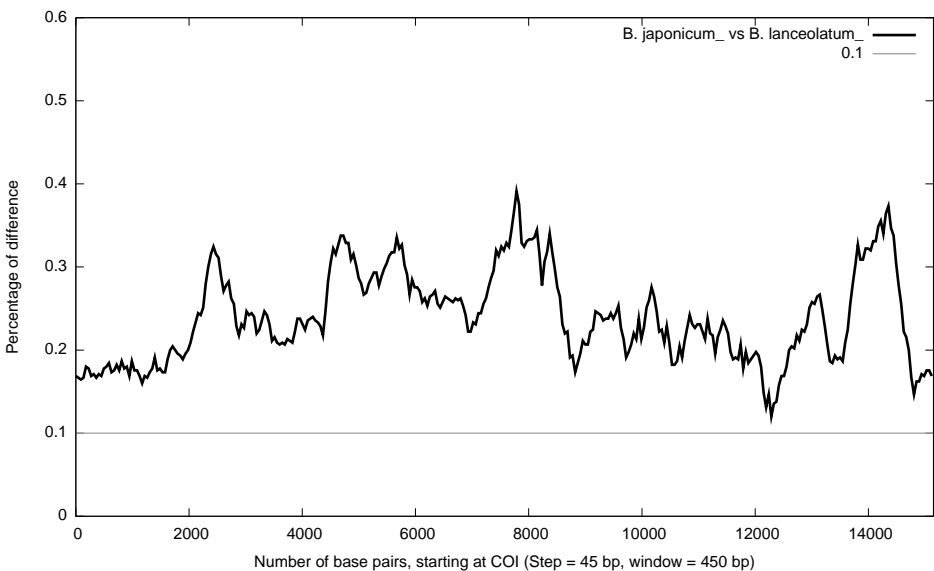

Supplement: File S4 — Sliding window analyses for Sauropsida, Aves, Hemichordata, Coelacanthimorpha, Dipnoi, Chondrichthyes and Cephalochordata. For each family, the folder contains the aligned sequences as well as the sliding window analyses by species pair and for all species pair on a single figure. (ZIP) [file pone.0051263.s004.zip › Cephalocordata/Branchiostomidae/45_450/Branchiostoma_japonicum_NC_008069_Branchiostoma_lanceolatum_NC_001912.pdf]

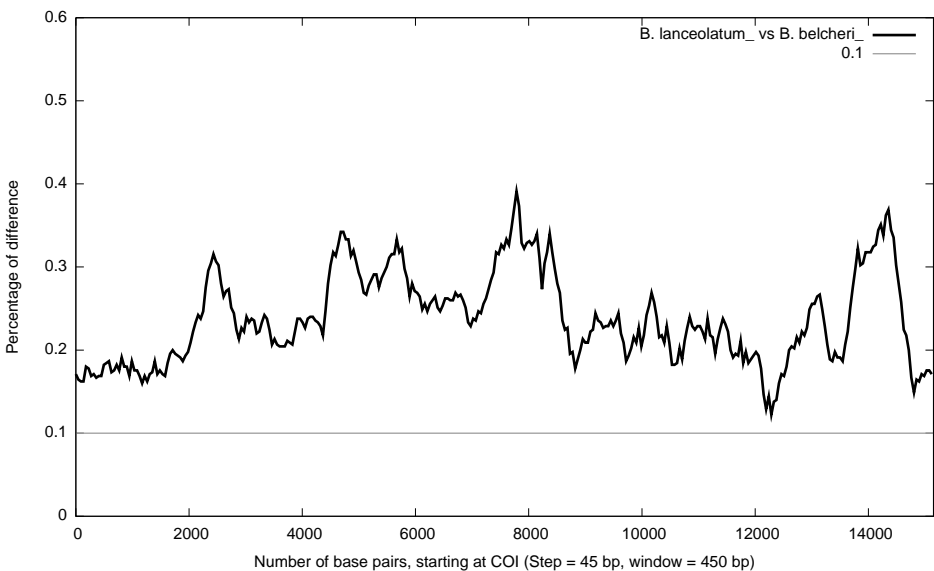

Supplement: File S4 — Sliding window analyses for Sauropsida, Aves, Hemichordata, Coelacanthimorpha, Dipnoi, Chondrichthyes and Cephalochordata. For each family, the folder contains the aligned sequences as well as the sliding window analyses by species pair and for all species pair on a single figure. (ZIP) [file pone.0051263.s004.zip › Cephalocordata/Branchiostomidae/45_450/Branchiostoma_lanceolatum_NC_001912_Branchiostoma_belcheri_NC_004537.pdf]

# Epigonichthyidae

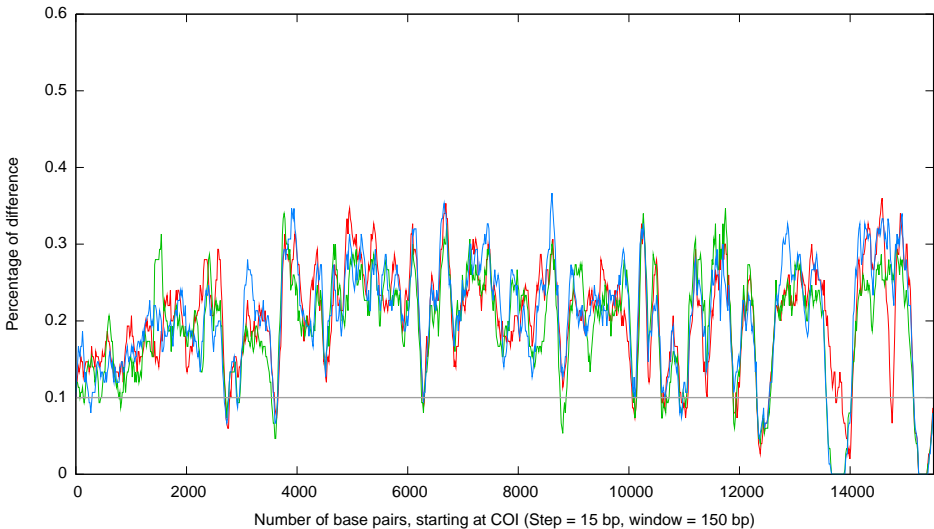

Supplement: File S4 — Sliding window analyses for Sauropsida, Aves, Hemichordata, Coelacanthimorpha, Dipnoi, Chondrichthyes and Cephalochordata. For each family, the folder contains the aligned sequences as well as the sliding window analyses by species pair and for all species pair on a single figure. (ZIP) [file pone.0051263.s004.zip › Cephalocordata/Epigonichthyidae/15_150/allCurves.pdf]

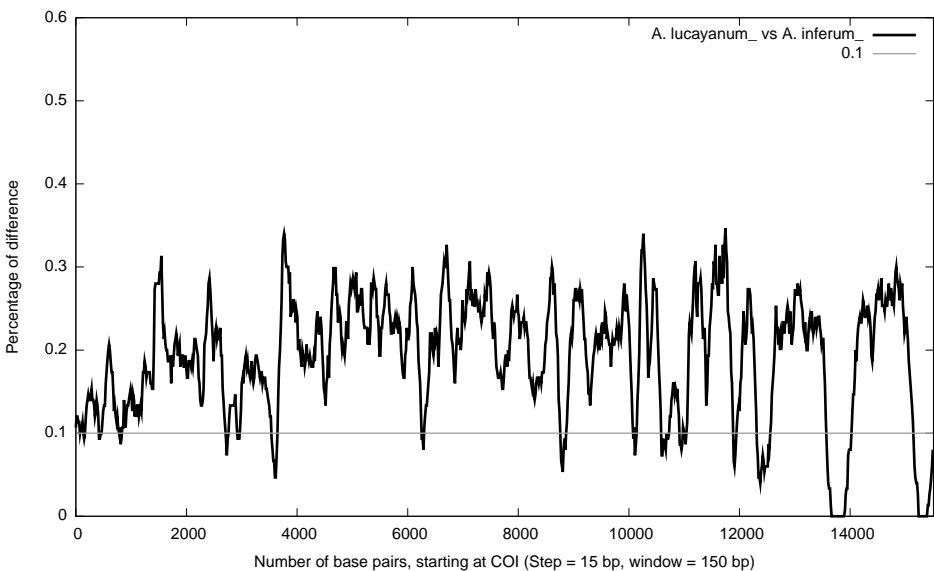

Supplement: File S4 — Sliding window analyses for Sauropsida, Aves, Hemichordata, Coelacanthimorpha, Dipnoi, Chondrichthyes and Cephalochordata. For each family, the folder contains the aligned sequences as well as the sliding window analyses by species pair and for all species pair on a single figure. (ZIP) [file pone.0051263.s004.zip › Cephalocordata/Epigonichthyidae/15_150/Asymmetron_lucayanum_NC_006464_Asymmetron_inferum_NC_009774.pdf]

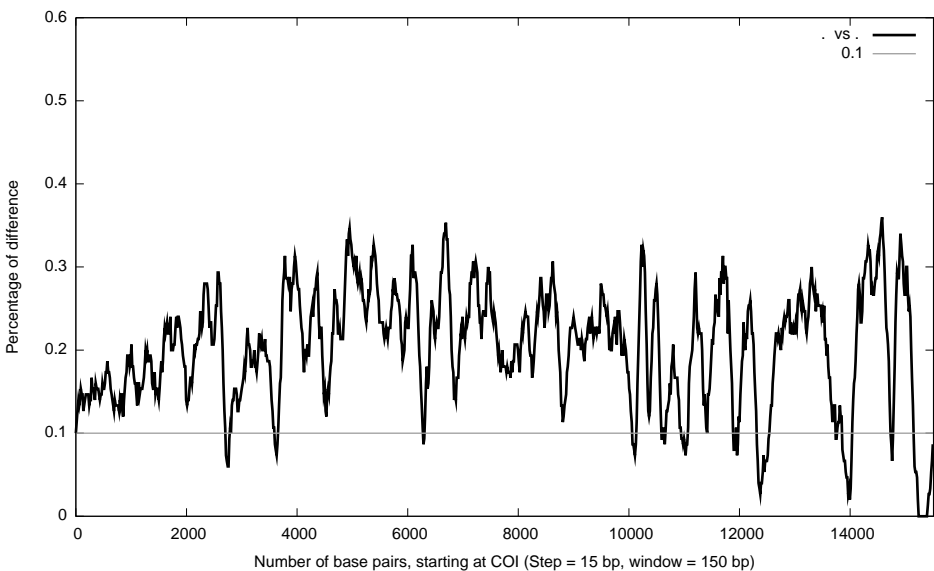

Supplement: File S4 — Sliding window analyses for Sauropsida, Aves, Hemichordata, Coelacanthimorpha, Dipnoi, Chondrichthyes and Cephalochordata. For each family, the folder contains the aligned sequences as well as the sliding window analyses by species pair and for all species pair on a single figure. (ZIP) [file pone.0051263.s004.zip › Cephalocordata/Epigonichthyidae/15_150/Asymmetron_lucayanum_NC_006464_Asymmetron_sp._A_TK-2007_NC_009775.pdf]

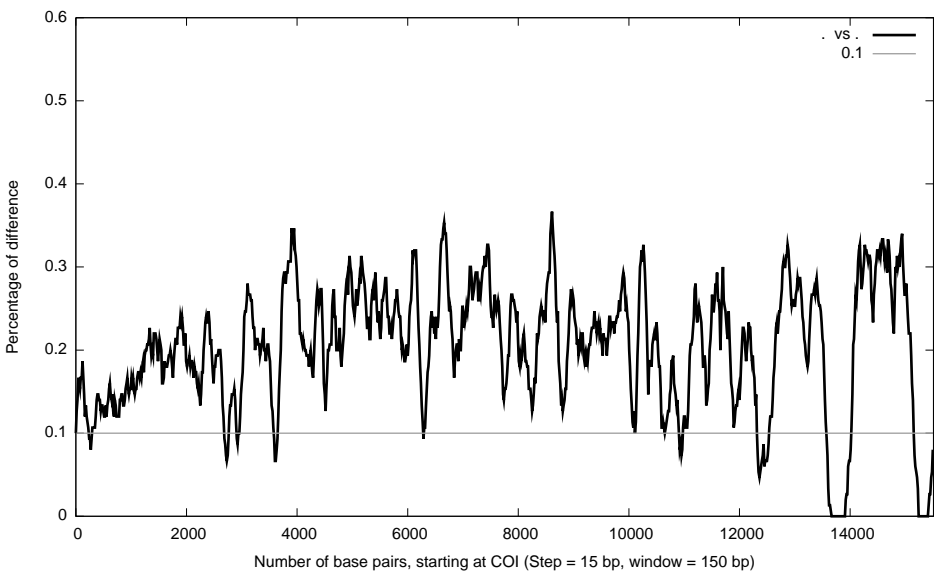

Supplement: File S4 — Sliding window analyses for Sauropsida, Aves, Hemichordata, Coelacanthimorpha, Dipnoi, Chondrichthyes and Cephalochordata. For each family, the folder contains the aligned sequences as well as the sliding window analyses by species pair and for all species pair on a single figure. (ZIP) [file pone.0051263.s004.zip › Cephalocordata/Epigonichthyidae/15_150/Asymmetron_sp._A_TK-2007_NC_009775_Asymmetron_inferum_NC_009774.pdf]

# Epigonichthyidae

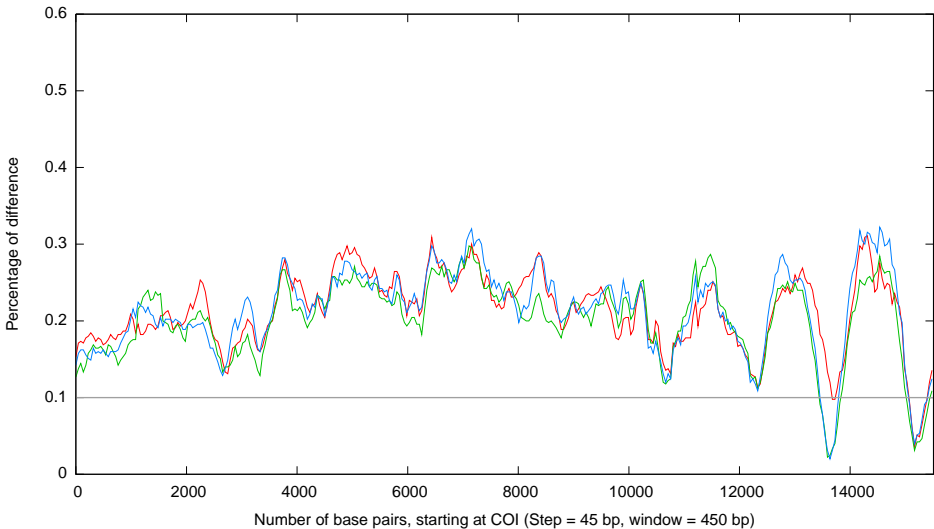

Supplement: File S4 — Sliding window analyses for Sauropsida, Aves, Hemichordata, Coelacanthimorpha, Dipnoi, Chondrichthyes and Cephalochordata. For each family, the folder contains the aligned sequences as well as the sliding window analyses by species pair and for all species pair on a single figure. (ZIP) [file pone.0051263.s004.zip › Cephalocordata/Epigonichthyidae/45_450/allCurves.pdf]

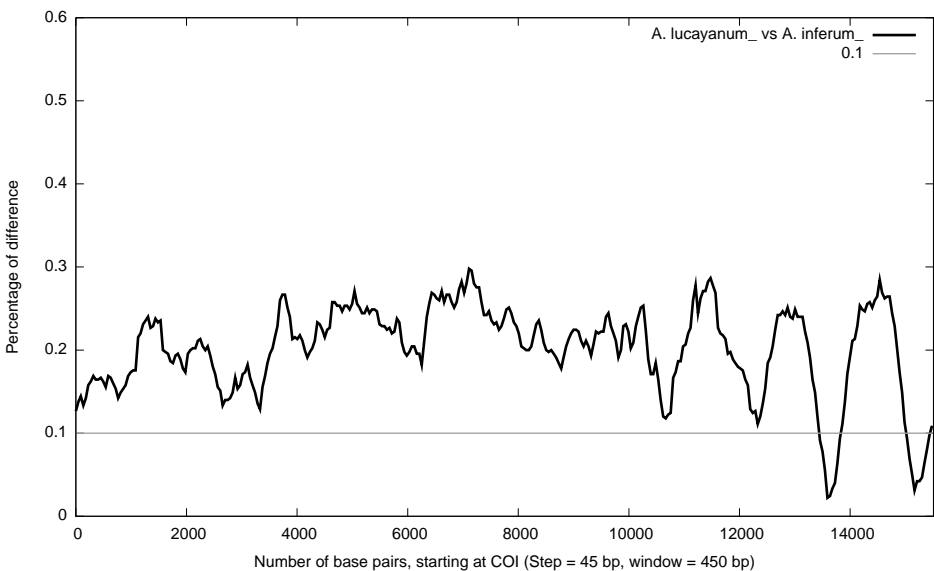

Supplement: File S4 — Sliding window analyses for Sauropsida, Aves, Hemichordata, Coelacanthimorpha, Dipnoi, Chondrichthyes and Cephalochordata. For each family, the folder contains the aligned sequences as well as the sliding window analyses by species pair and for all species pair on a single figure. (ZIP) [file pone.0051263.s004.zip › Cephalocordata/Epigonichthyidae/45_450/Asymmetron_lucayanum_NC_006464_Asymmetron_inferum_NC_009774.pdf]

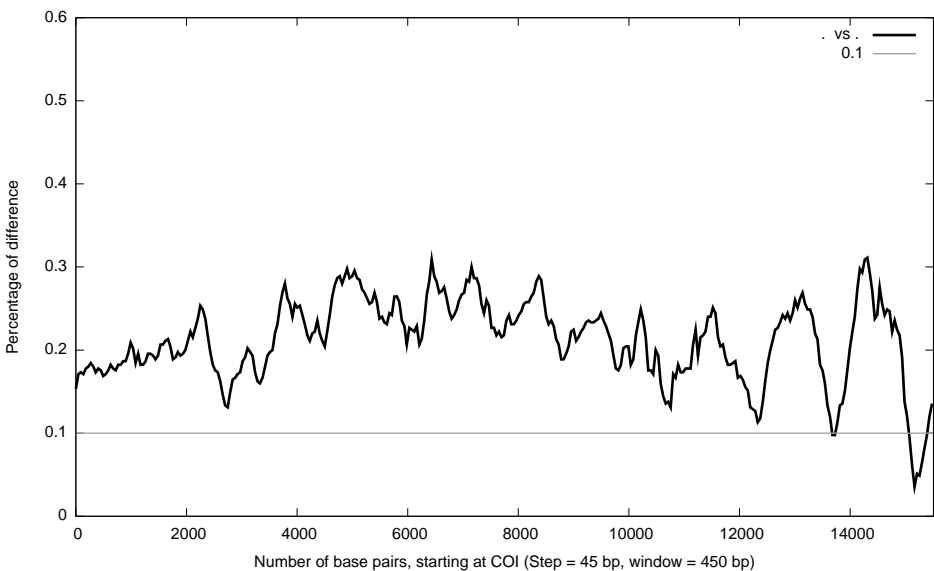

Supplement: File S4 — Sliding window analyses for Sauropsida, Aves, Hemichordata, Coelacanthimorpha, Dipnoi, Chondrichthyes and Cephalochordata. For each family, the folder contains the aligned sequences as well as the sliding window analyses by species pair and for all species pair on a single figure. (ZIP) [file pone.0051263.s004.zip › Cephalocordata/Epigonichthyidae/45_450/Asymmetron_lucayanum_NC_006464_Asymmetron_sp._A_TK-2007_NC_009775.pdf]

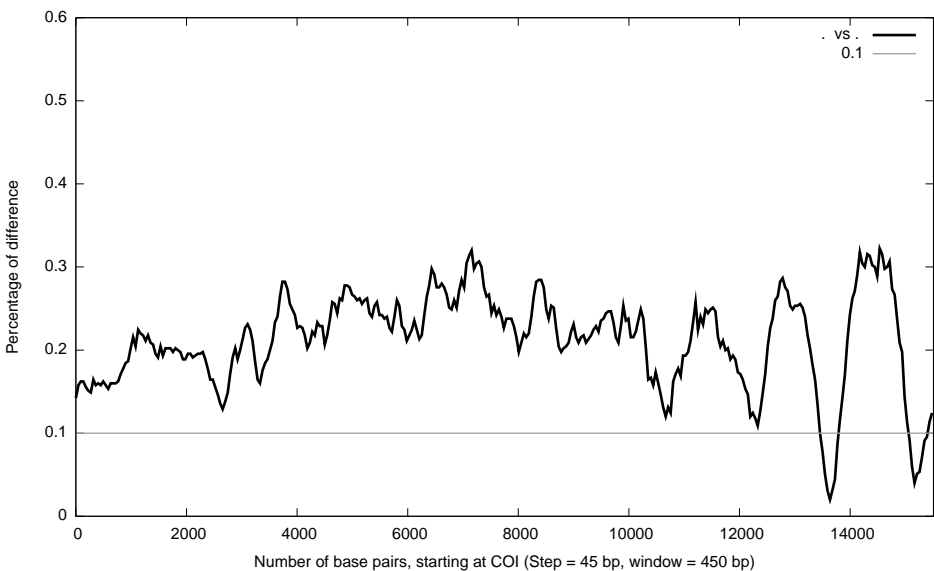

Supplement: File S4 — Sliding window analyses for Sauropsida, Aves, Hemichordata, Coelacanthimorpha, Dipnoi, Chondrichthyes and Cephalochordata. For each family, the folder contains the aligned sequences as well as the sliding window analyses by species pair and for all species pair on a single figure. (ZIP) [file pone.0051263.s004.zip › Cephalocordata/Epigonichthyidae/45_450/Asymmetron_sp._A_TK-2007_NC_009775_Asymmetron_inferum_NC_009774.pdf]

# Chimaeridae

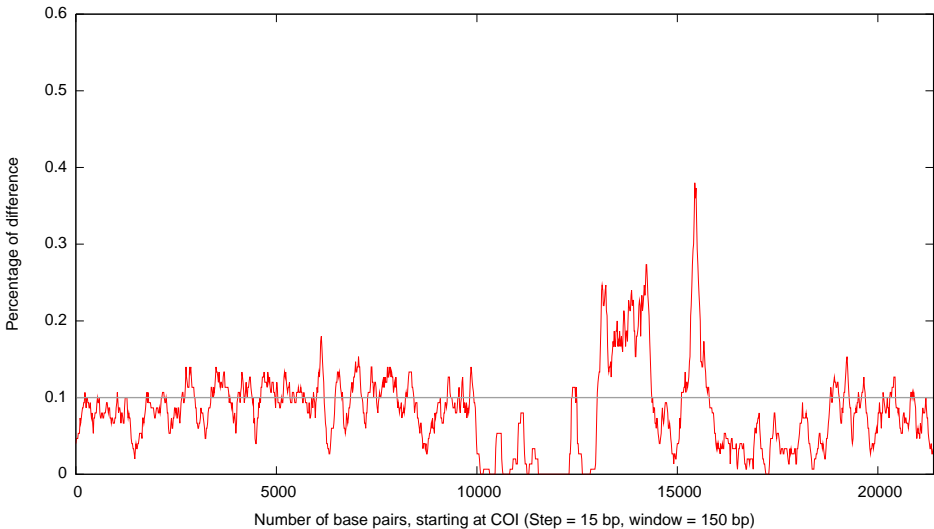

Supplement: File S4 — Sliding window analyses for Sauropsida, Aves, Hemichordata, Coelacanthimorpha, Dipnoi, Chondrichthyes and Cephalochordata. For each family, the folder contains the aligned sequences as well as the sliding window analyses by species pair and for all species pair on a single figure. (ZIP) [file pone.0051263.s004.zip › Chondrichthyes/Chimaeridae/15_150/allCurves.pdf]

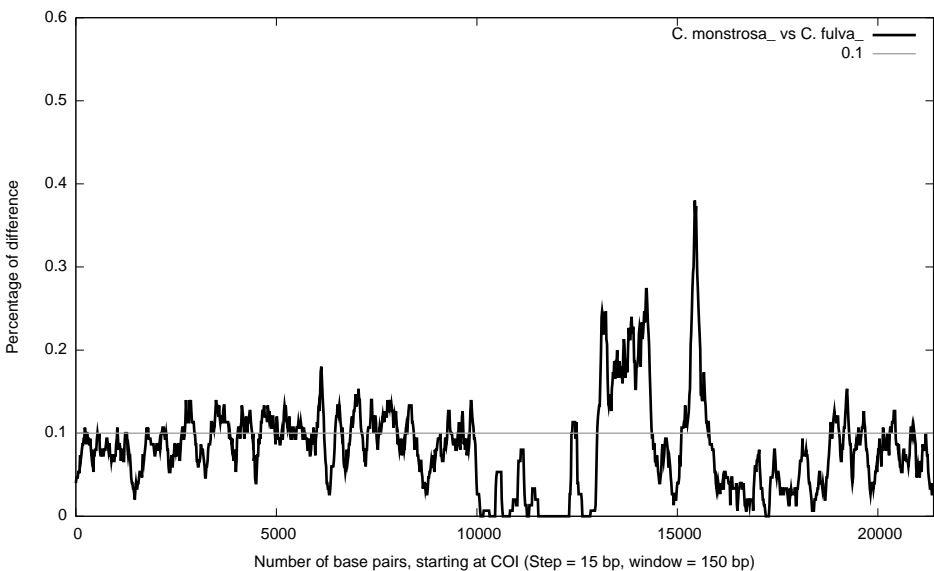

Supplement: File S4 — Sliding window analyses for Sauropsida, Aves, Hemichordata, Coelacanthimorpha, Dipnoi, Chondrichthyes and Cephalochordata. For each family, the folder contains the aligned sequences as well as the sliding window analyses by species pair and for all species pair on a single figure. (ZIP) [file pone.0051263.s004.zip › Chondrichthyes/Chimaeridae/15_150/Chimaera_monstrosa_NC_003136_Chimaera_fulva_NC_014288.pdf]

# Chimaeridae

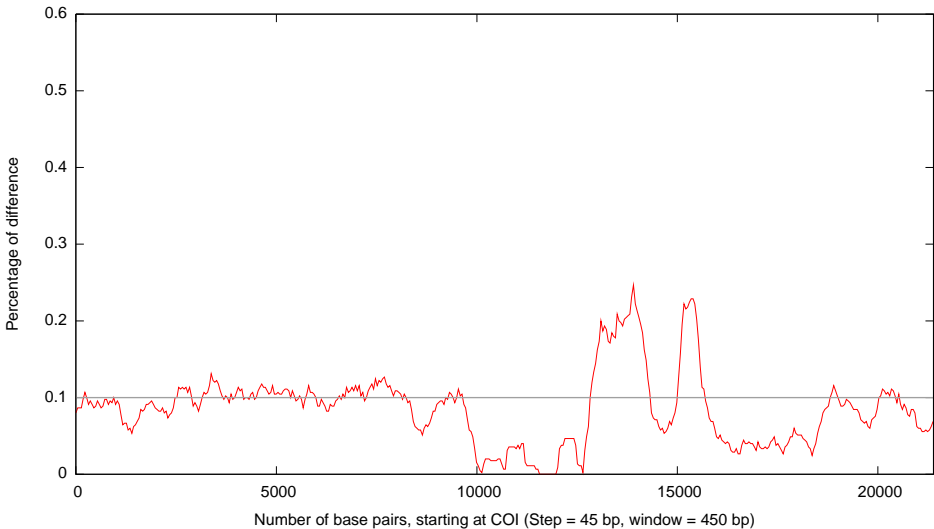

Supplement: File S4 — Sliding window analyses for Sauropsida, Aves, Hemichordata, Coelacanthimorpha, Dipnoi, Chondrichthyes and Cephalochordata. For each family, the folder contains the aligned sequences as well as the sliding window analyses by species pair and for all species pair on a single figure. (ZIP) [file pone.0051263.s004.zip › Chondrichthyes/Chimaeridae/45_450/allCurves.pdf]

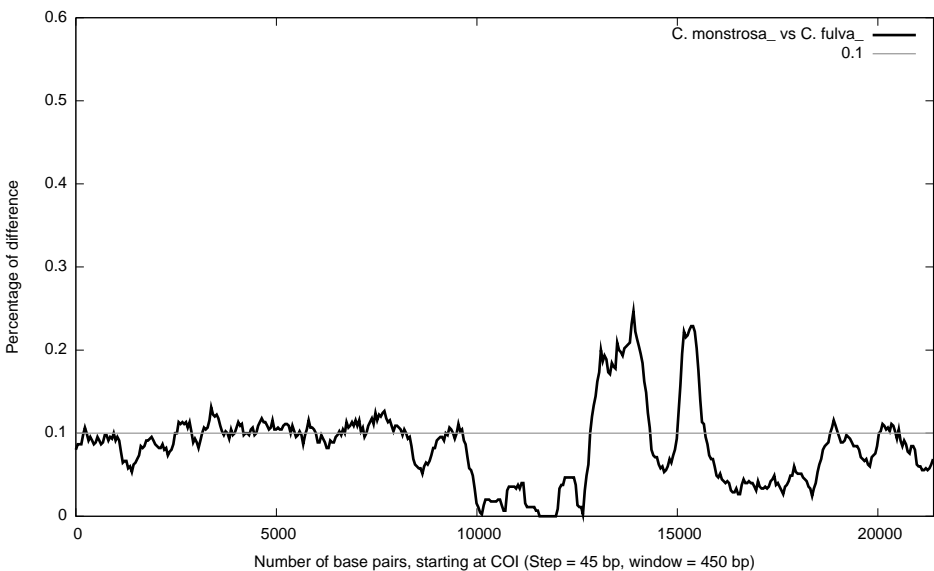

Supplement: File S4 — Sliding window analyses for Sauropsida, Aves, Hemichordata, Coelacanthimorpha, Dipnoi, Chondrichthyes and Cephalochordata. For each family, the folder contains the aligned sequences as well as the sliding window analyses by species pair and for all species pair on a single figure. (ZIP) [file pone.0051263.s004.zip › Chondrichthyes/Chimaeridae/45_450/Chimaera_monstrosa_NC_003136_Chimaera_fulva_NC_014288.pdf]

# Coelacanthidae

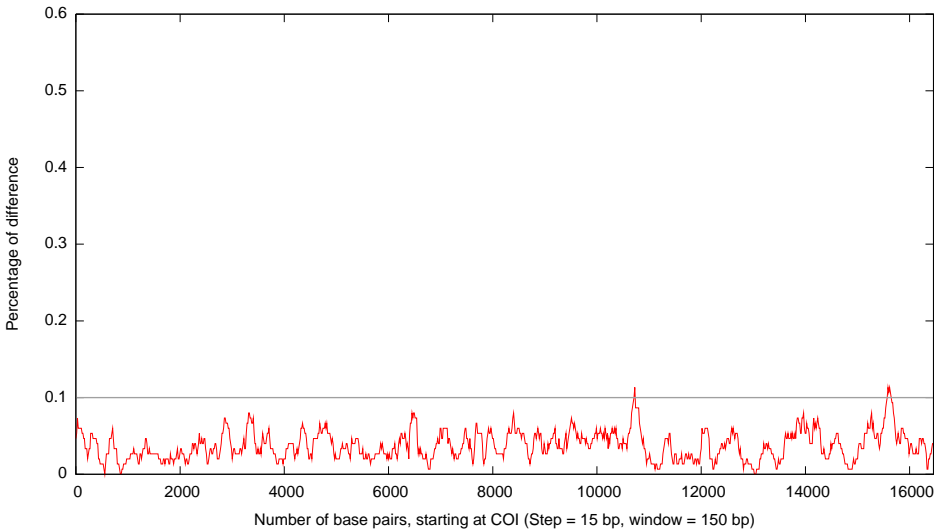

Supplement: File S4 — Sliding window analyses for Sauropsida, Aves, Hemichordata, Coelacanthimorpha, Dipnoi, Chondrichthyes and Cephalochordata. For each family, the folder contains the aligned sequences as well as the sliding window analyses by species pair and for all species pair on a single figure. (ZIP) [file pone.0051263.s004.zip › Coelacanthimorpha/Coelacanthidae/15_150/allCurves.pdf]

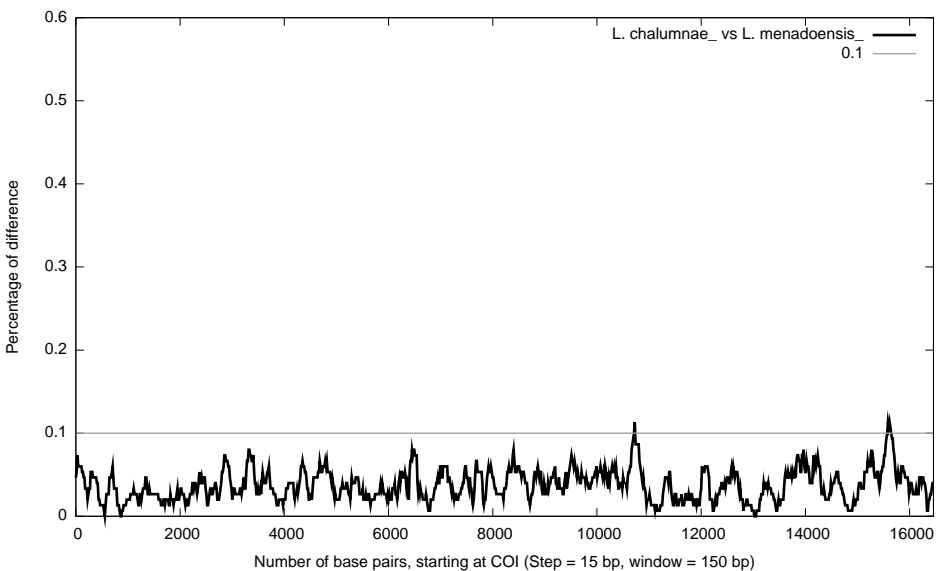

Supplement: File S4 — Sliding window analyses for Sauropsida, Aves, Hemichordata, Coelacanthimorpha, Dipnoi, Chondrichthyes and Cephalochordata. For each family, the folder contains the aligned sequences as well as the sliding window analyses by species pair and for all species pair on a single figure. (ZIP) [file pone.0051263.s004.zip › Coelacanthimorpha/Coelacanthidae/15_150/Latimeria_chalumnae_NC_001804_Latimeria_menadoensis_NC_006921.pdf]

# Coelacanthidae

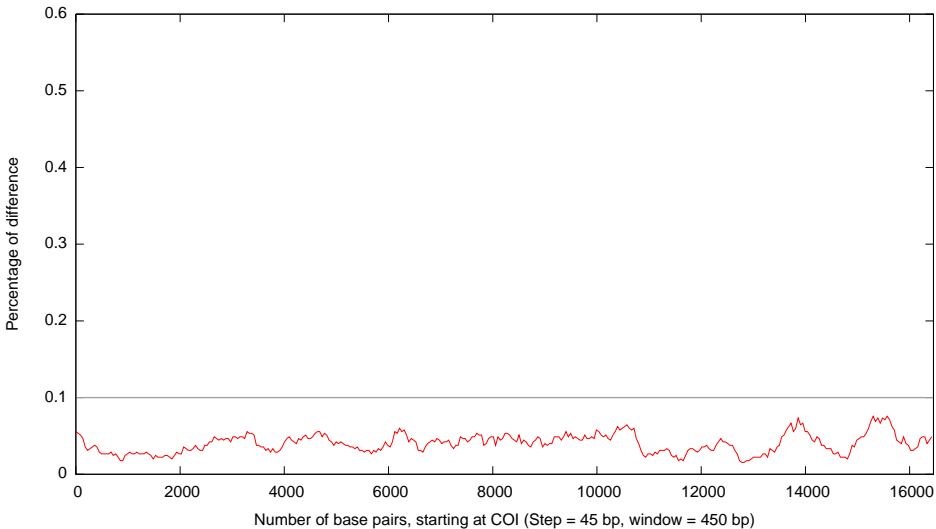

Supplement: File S4 — Sliding window analyses for Sauropsida, Aves, Hemichordata, Coelacanthimorpha, Dipnoi, Chondrichthyes and Cephalochordata. For each family, the folder contains the aligned sequences as well as the sliding window analyses by species pair and for all species pair on a single figure. (ZIP) [file pone.0051263.s004.zip › Coelacanthimorpha/Coelacanthidae/45_450/allCurves.pdf]

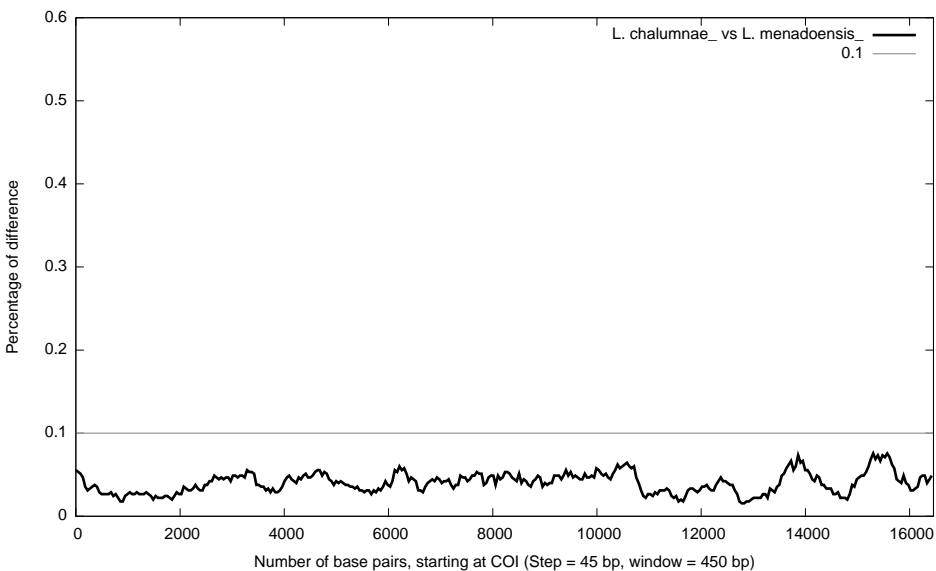

Supplement: File S4 — Sliding window analyses for Sauropsida, Aves, Hemichordata, Coelacanthimorpha, Dipnoi, Chondrichthyes and Cephalochordata. For each family, the folder contains the aligned sequences as well as the sliding window analyses by species pair and for all species pair on a single figure. (ZIP) [file pone.0051263.s004.zip › Coelacanthimorpha/Coelacanthidae/45_450/Latimeria_chalumnae_NC_001804_Latimeria_menadoensis_NC_006921.pdf]

# Protopteridae

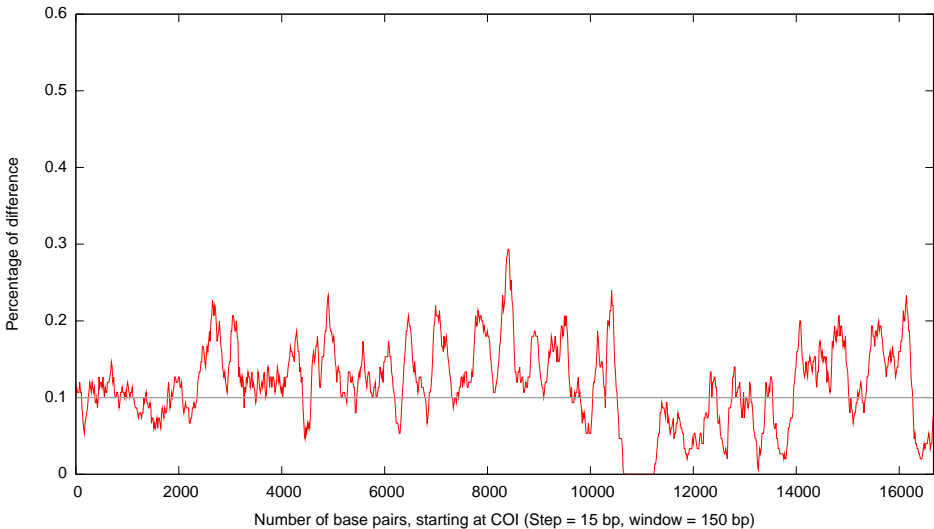

Supplement: File S4 — Sliding window analyses for Sauropsida, Aves, Hemichordata, Coelacanthimorpha, Dipnoi, Chondrichthyes and Cephalochordata. For each family, the folder contains the aligned sequences as well as the sliding window analyses by species pair and for all species pair on a single figure. (ZIP) [file pone.0051263.s004.zip › Dipnoi/Protopteridae/15_150/allCurves.pdf]

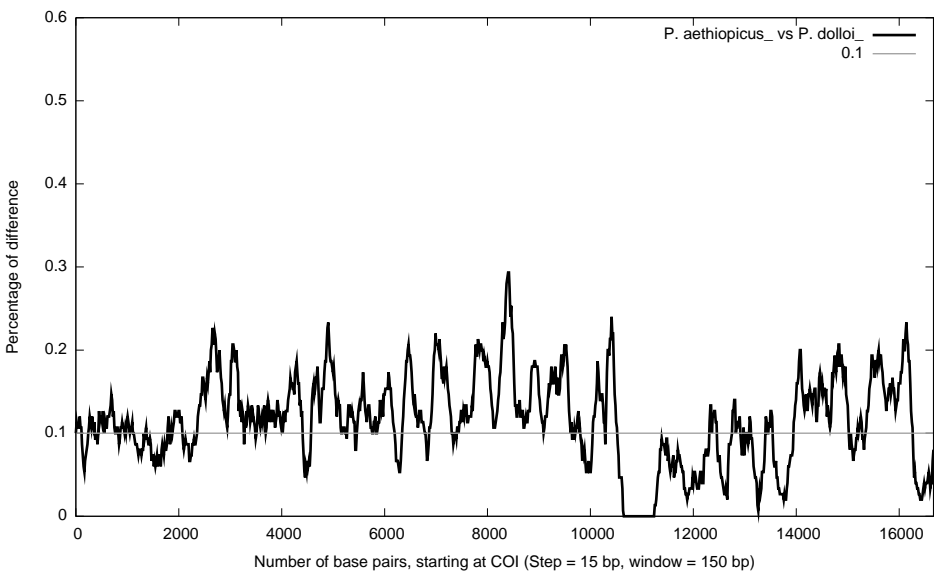

Supplement: File S4 — Sliding window analyses for Sauropsida, Aves, Hemichordata, Coelacanthimorpha, Dipnoi, Chondrichthyes and Cephalochordata. For each family, the folder contains the aligned sequences as well as the sliding window analyses by species pair and for all species pair on a single figure. (ZIP) [file pone.0051263.s004.zip › Dipnoi/Protopteridae/15_150/Protopterus_aethiopicus_NC_014764_Protopterus_dolloi_NC_001708.pdf]

# Protopteridae

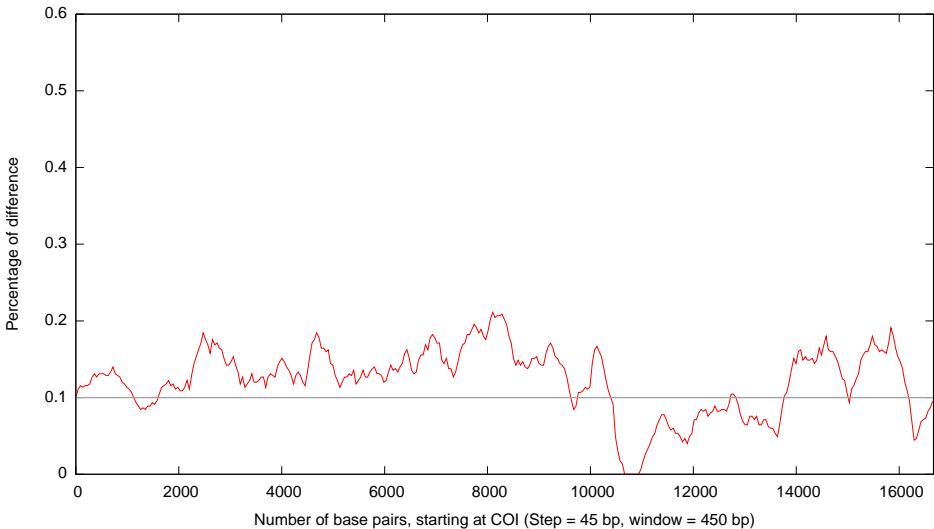

Supplement: File S4 — Sliding window analyses for Sauropsida, Aves, Hemichordata, Coelacanthimorpha, Dipnoi, Chondrichthyes and Cephalochordata. For each family, the folder contains the aligned sequences as well as the sliding window analyses by species pair and for all species pair on a single figure. (ZIP) [file pone.0051263.s004.zip › Dipnoi/Protopteridae/45_450/allCurves.pdf]

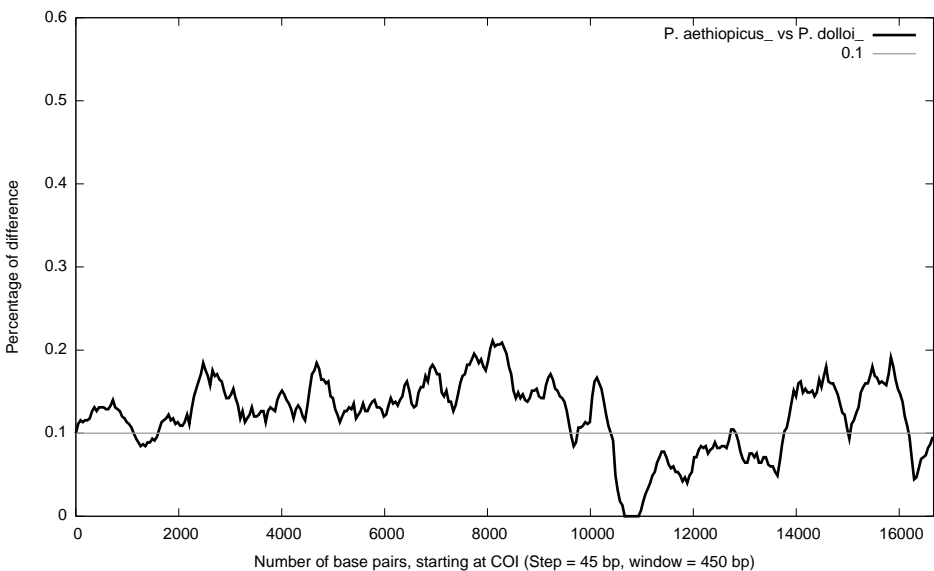

Supplement: File S4 — Sliding window analyses for Sauropsida, Aves, Hemichordata, Coelacanthimorpha, Dipnoi, Chondrichthyes and Cephalochordata. For each family, the folder contains the aligned sequences as well as the sliding window analyses by species pair and for all species pair on a single figure. (ZIP) [file pone.0051263.s004.zip › Dipnoi/Protopteridae/45_450/Protopterus_aethiopicus_NC_014764_Protopterus_dolloi_NC_001708.pdf]

# Ptychoderidae

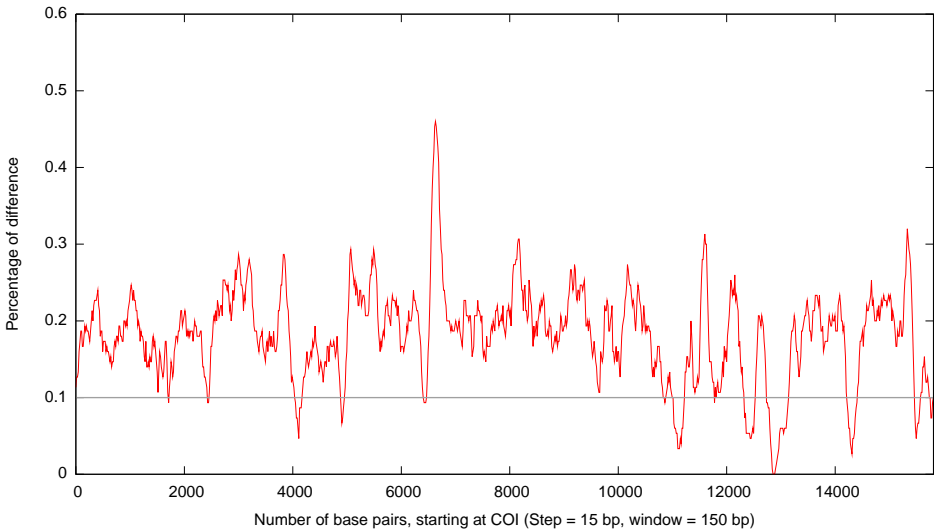

Supplement: File S4 — Sliding window analyses for Sauropsida, Aves, Hemichordata, Coelacanthimorpha, Dipnoi, Chondrichthyes and Cephalochordata. For each family, the folder contains the aligned sequences as well as the sliding window analyses by species pair and for all species pair on a single figure. (ZIP) [file pone.0051263.s004.zip › Hemichordata/Ptychoderidae/15_150/allCurves.pdf]

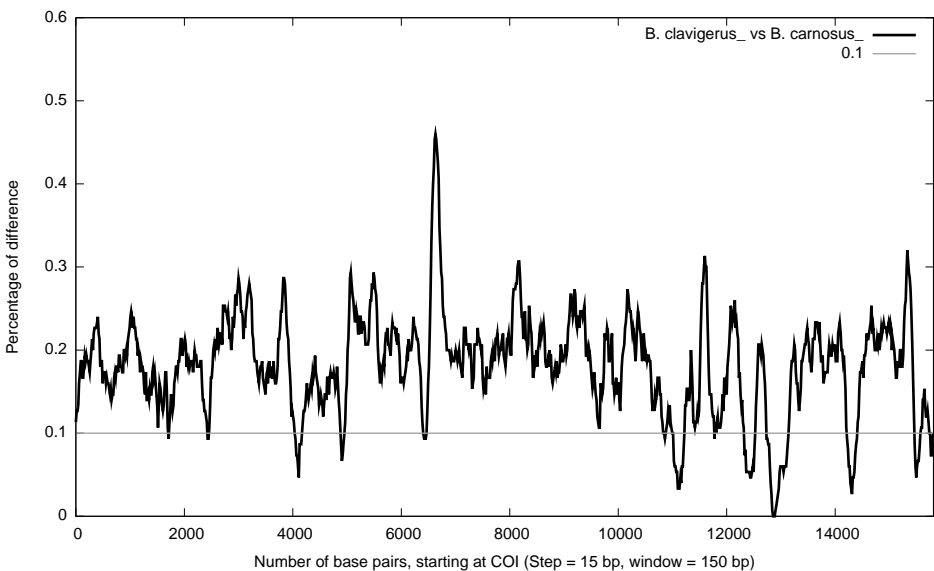

Supplement: File S4 — Sliding window analyses for Sauropsida, Aves, Hemichordata, Coelacanthimorpha, Dipnoi, Chondrichthyes and Cephalochordata. For each family, the folder contains the aligned sequences as well as the sliding window analyses by species pair and for all species pair on a single figure. (ZIP) [file pone.0051263.s004.zip › Hemichordata/Ptychoderidae/15_150/Balanoglossus_clavigerus_NC_013877_Balanoglossus_carnosus_NC_001887.pdf]

# Ptychoderidae

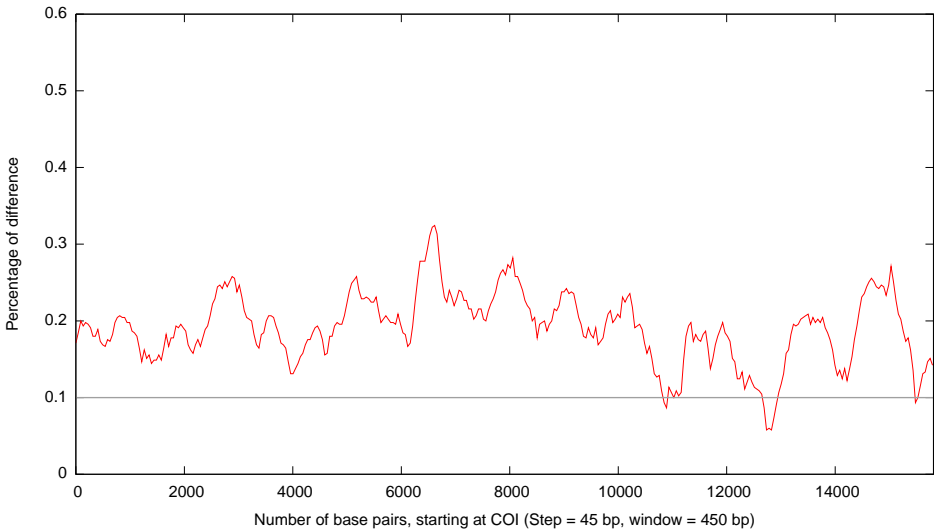

Supplement: File S4 — Sliding window analyses for Sauropsida, Aves, Hemichordata, Coelacanthimorpha, Dipnoi, Chondrichthyes and Cephalochordata. For each family, the folder contains the aligned sequences as well as the sliding window analyses by species pair and for all species pair on a single figure. (ZIP) [file pone.0051263.s004.zip › Hemichordata/Ptychoderidae/45_450/allCurves.pdf]

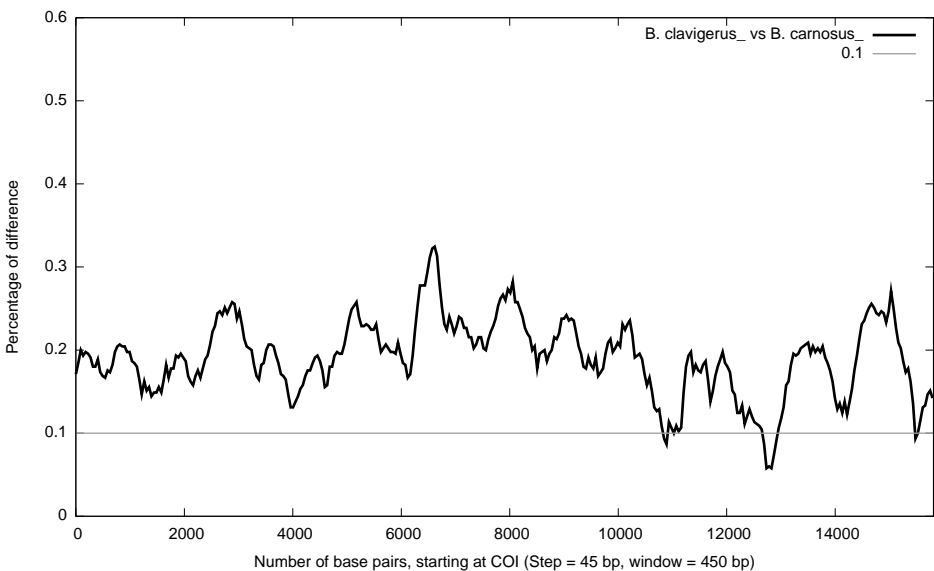

Supplement: File S4 — Sliding window analyses for Sauropsida, Aves, Hemichordata, Coelacanthimorpha, Dipnoi, Chondrichthyes and Cephalochordata. For each family, the folder contains the aligned sequences as well as the sliding window analyses by species pair and for all species pair on a single figure. (ZIP) [file pone.0051263.s004.zip › Hemichordata/Ptychoderidae/45_450/Balanoglossus_clavigerus_NC_013877_Balanoglossus_carnosus_NC_001887.pdf]

# Accipitridae

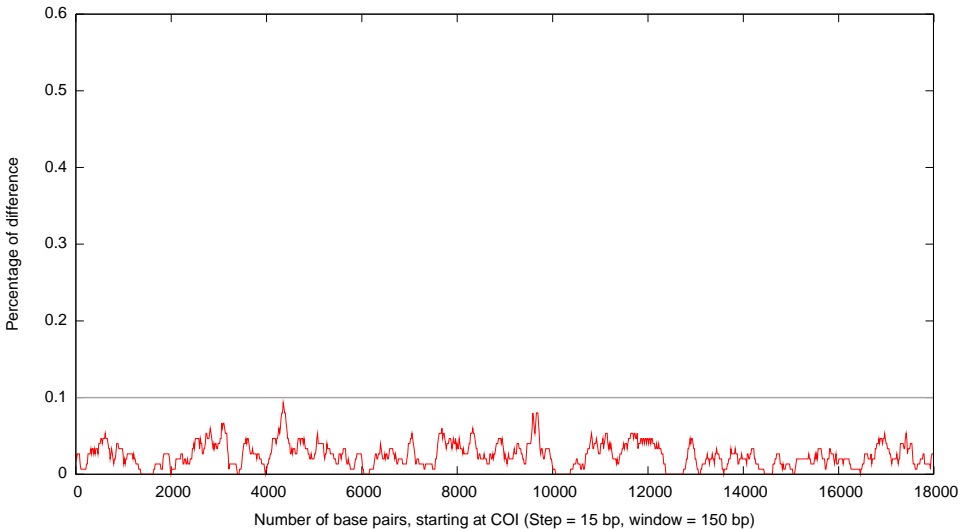

Supplement: File S4 — Sliding window analyses for Sauropsida, Aves, Hemichordata, Coelacanthimorpha, Dipnoi, Chondrichthyes and Cephalochordata. For each family, the folder contains the aligned sequences as well as the sliding window analyses by species pair and for all species pair on a single figure. (ZIP) [file pone.0051263.s004.zip › Sauropsida & Aves/Accipitridae/15_150/allCurves.pdf]

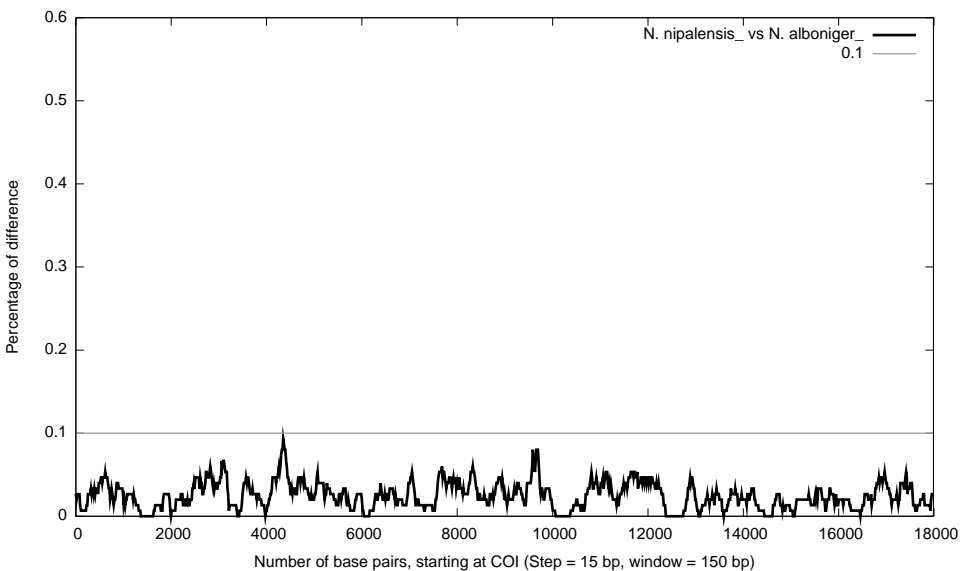

Supplement: File S4 — Sliding window analyses for Sauropsida, Aves, Hemichordata, Coelacanthimorpha, Dipnoi, Chondrichthyes and Cephalochordata. For each family, the folder contains the aligned sequences as well as the sliding window analyses by species pair and for all species pair on a single figure. (ZIP) [file pone.0051263.s004.zip › Sauropsida & Aves/Accipitridae/15_150/Nisaetus_nipalensis_NC_007598_Nisaetus_alboniger_NC_007599.pdf]

# Accipitridae

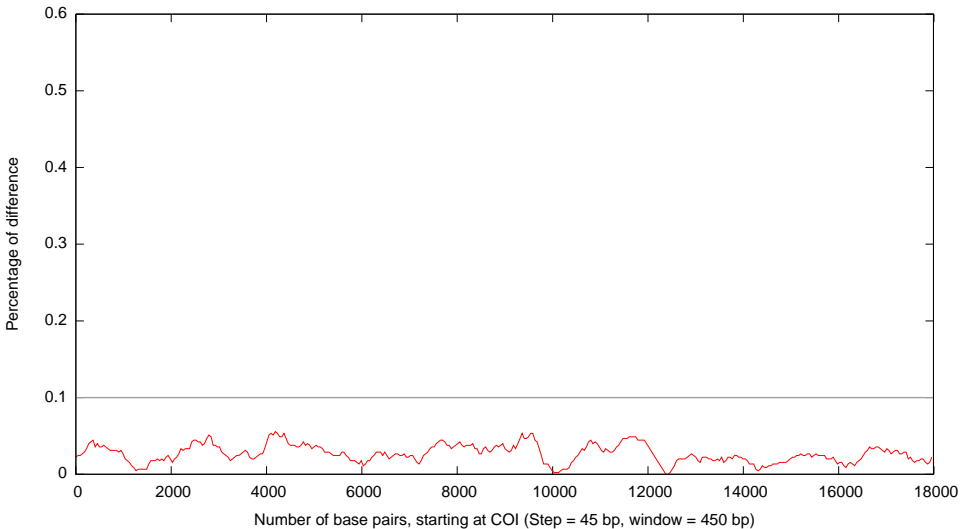

Supplement: File S4 — Sliding window analyses for Sauropsida, Aves, Hemichordata, Coelacanthimorpha, Dipnoi, Chondrichthyes and Cephalochordata. For each family, the folder contains the aligned sequences as well as the sliding window analyses by species pair and for all species pair on a single figure. (ZIP) [file pone.0051263.s004.zip › Sauropsida & Aves/Accipitridae/45_450/allCurves.pdf]

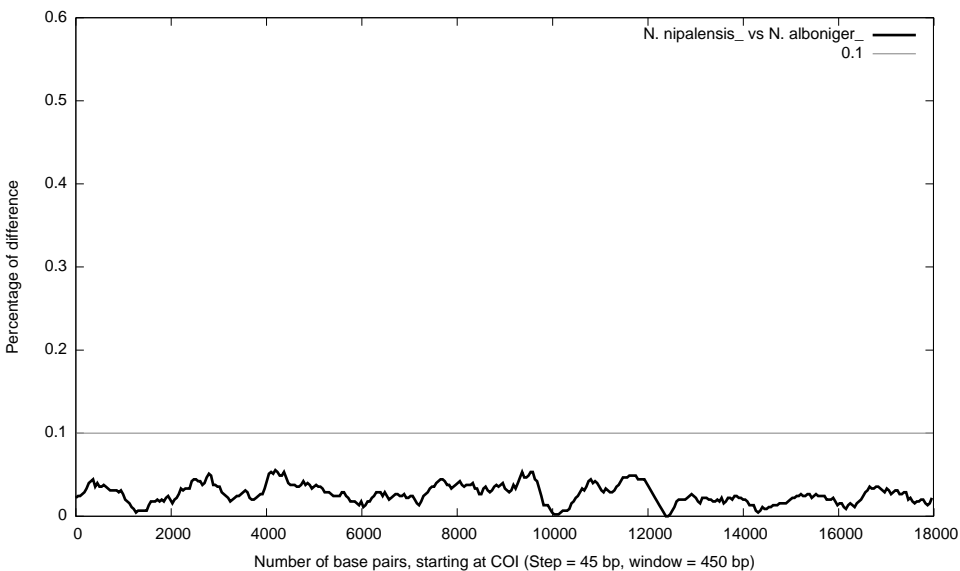

Supplement: File S4 — Sliding window analyses for Sauropsida, Aves, Hemichordata, Coelacanthimorpha, Dipnoi, Chondrichthyes and Cephalochordata. For each family, the folder contains the aligned sequences as well as the sliding window analyses by species pair and for all species pair on a single figure. (ZIP) [file pone.0051263.s004.zip › Sauropsida & Aves/Accipitridae/45_450/Nisaetus_nipalensis_NC_007598_Nisaetus_alboniger_NC_007599.pdf]

# Anatidae

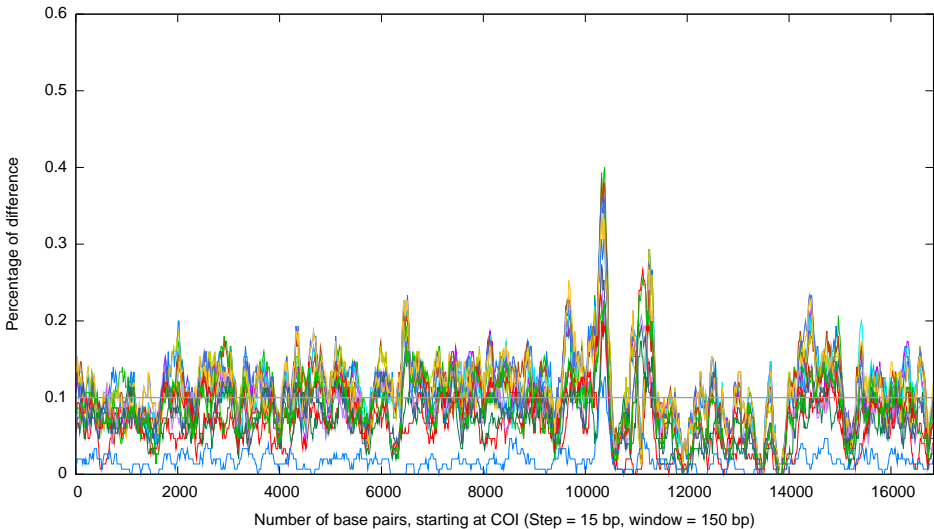

Supplement: File S4 — Sliding window analyses for Sauropsida, Aves, Hemichordata, Coelacanthimorpha, Dipnoi, Chondrichthyes and Cephalochordata. For each family, the folder contains the aligned sequences as well as the sliding window analyses by species pair and for all species pair on a single figure. (ZIP) [file pone.0051263.s004.zip › Sauropsida & Aves/Anatidae/15_150/allCurves.pdf]

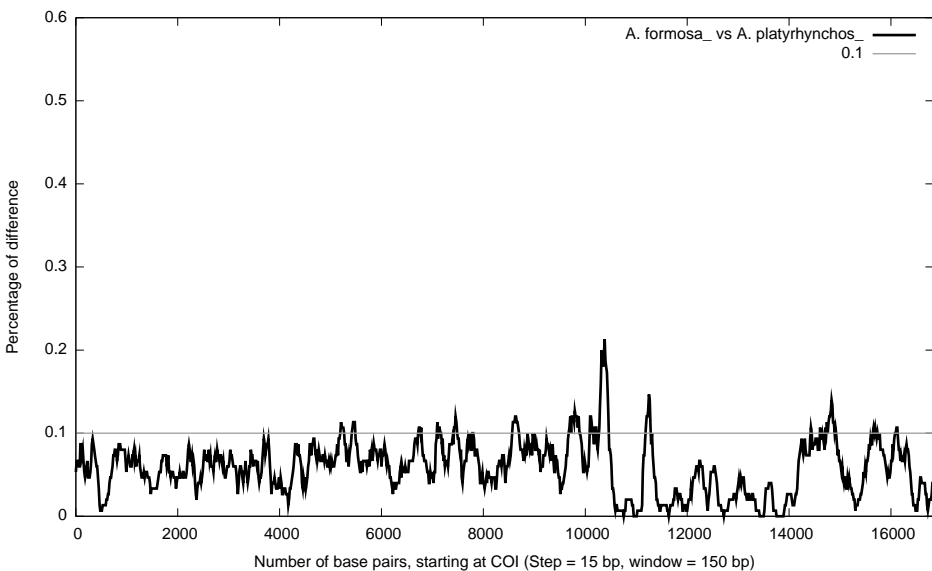

Supplement: File S4 — Sliding window analyses for Sauropsida, Aves, Hemichordata, Coelacanthimorpha, Dipnoi, Chondrichthyes and Cephalochordata. For each family, the folder contains the aligned sequences as well as the sliding window analyses by species pair and for all species pair on a single figure. (ZIP) [file pone.0051263.s004.zip › Sauropsida & Aves/Anatidae/15_150/Anas_formosa_NC_015482_Anas_platyrhynchos_NC_009684.pdf]

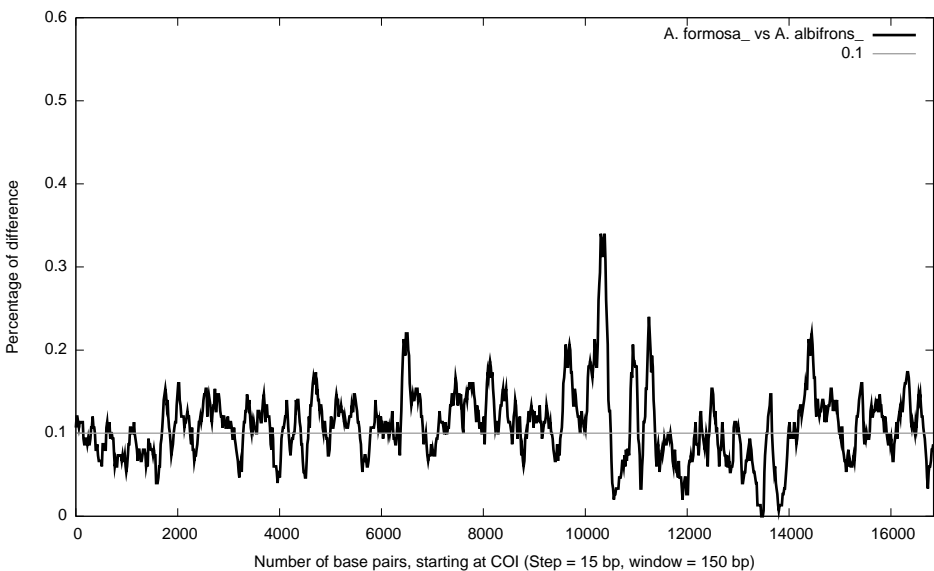

Supplement: File S4 — Sliding window analyses for Sauropsida, Aves, Hemichordata, Coelacanthimorpha, Dipnoi, Chondrichthyes and Cephalochordata. For each family, the folder contains the aligned sequences as well as the sliding window analyses by species pair and for all species pair on a single figure. (ZIP) [file pone.0051263.s004.zip › Sauropsida & Aves/Anatidae/15_150/Anas_formosa_NC_015482_Anser_albifrons_NC_004539.pdf]

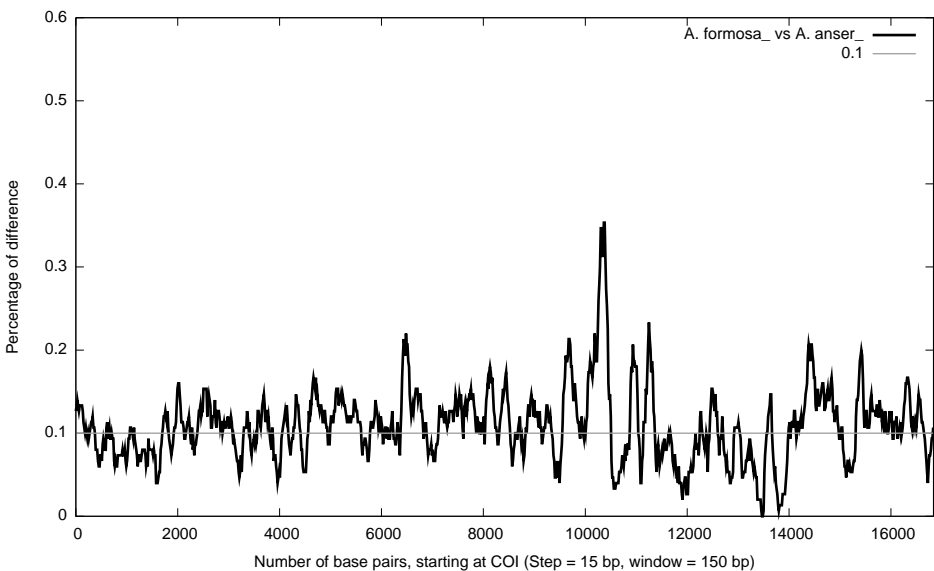

Supplement: File S4 — Sliding window analyses for Sauropsida, Aves, Hemichordata, Coelacanthimorpha, Dipnoi, Chondrichthyes and Cephalochordata. For each family, the folder contains the aligned sequences as well as the sliding window analyses by species pair and for all species pair on a single figure. (ZIP) [file pone.0051263.s004.zip › Sauropsida & Aves/Anatidae/15_150/Anas_formosa_NC_015482_Anser_anser_NC_011196.pdf]
