# Supplementary figures and images for: Conveniently Pre-Tagged and Pre-Packaged: Extended Molecular Identification and Metagenomics Using Complete Metazoan Mitochondrial Genomes (part 3 of 3)
Source: PLoS One. 2012 Dec 14;7(12):e51263. doi: 10.1371/journal.pone.0051263 (PMC3522660; doi:10.1371/journal.pone.0051263)

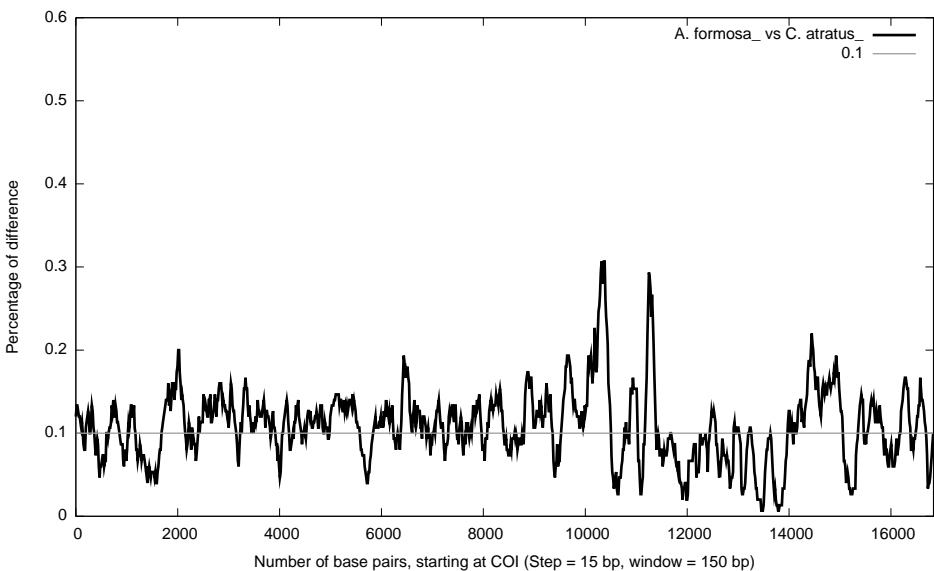

Supplement: File S4 — Sliding window analyses for Sauropsida, Aves, Hemichordata, Coelacanthimorpha, Dipnoi, Chondrichthyes and Cephalochordata. For each family, the folder contains the aligned sequences as well as the sliding window analyses by species pair and for all species pair on a single figure. (ZIP) [file pone.0051263.s004.zip › Sauropsida & Aves/Anatidae/15_150/Anas_formosa_NC_015482_Cygnus_atratus_NC_012843.pdf]

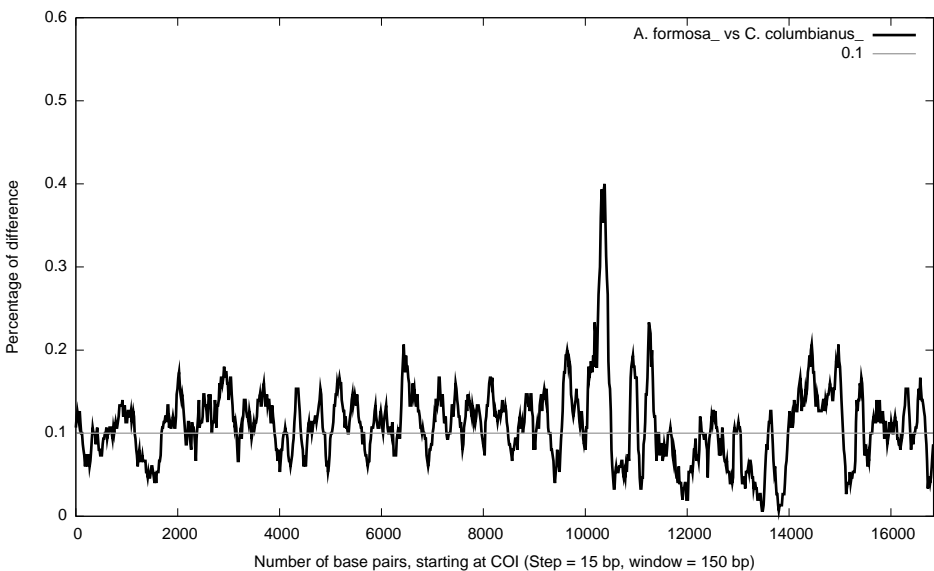

Supplement: File S4 — Sliding window analyses for Sauropsida, Aves, Hemichordata, Coelacanthimorpha, Dipnoi, Chondrichthyes and Cephalochordata. For each family, the folder contains the aligned sequences as well as the sliding window analyses by species pair and for all species pair on a single figure. (ZIP) [file pone.0051263.s004.zip › Sauropsida & Aves/Anatidae/15_150/Anas_formosa_NC_015482_Cygnus_columbianus_NC_007691.pdf]

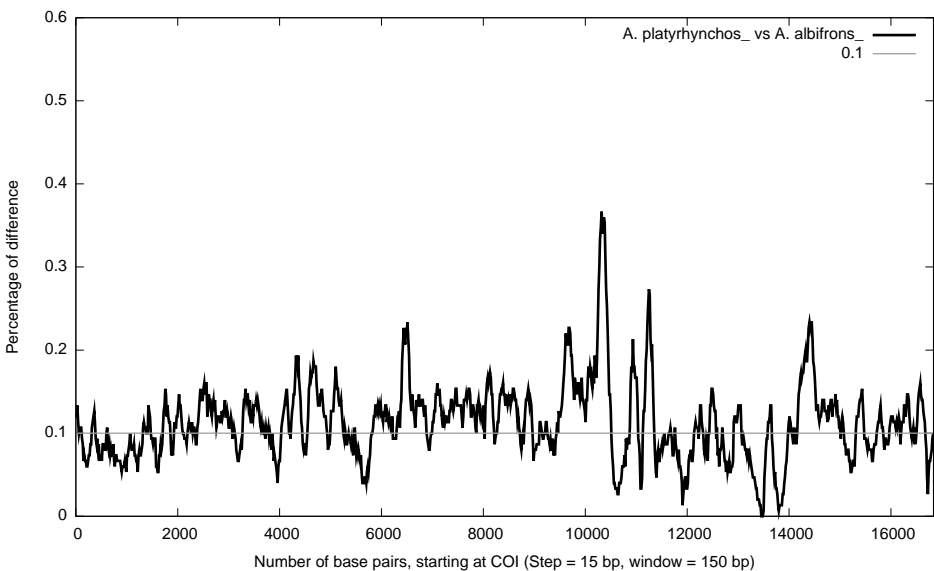

Supplement: File S4 — Sliding window analyses for Sauropsida, Aves, Hemichordata, Coelacanthimorpha, Dipnoi, Chondrichthyes and Cephalochordata. For each family, the folder contains the aligned sequences as well as the sliding window analyses by species pair and for all species pair on a single figure. (ZIP) [file pone.0051263.s004.zip › Sauropsida & Aves/Anatidae/15_150/Anas_platyrhynchos_NC_009684_Anser_albifrons_NC_004539.pdf]

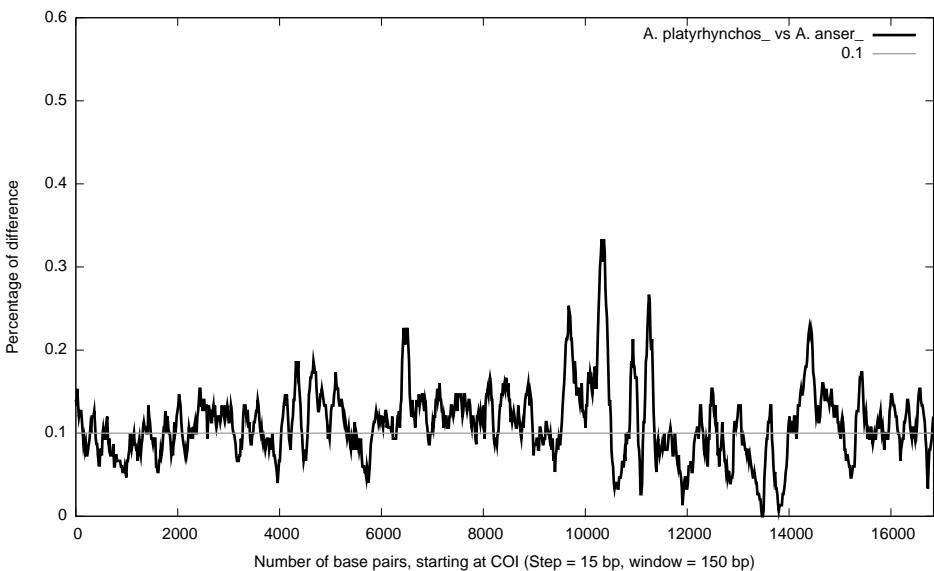

Supplement: File S4 — Sliding window analyses for Sauropsida, Aves, Hemichordata, Coelacanthimorpha, Dipnoi, Chondrichthyes and Cephalochordata. For each family, the folder contains the aligned sequences as well as the sliding window analyses by species pair and for all species pair on a single figure. (ZIP) [file pone.0051263.s004.zip › Sauropsida & Aves/Anatidae/15_150/Anas_platyrhynchos_NC_009684_Anser_anser_NC_011196.pdf]

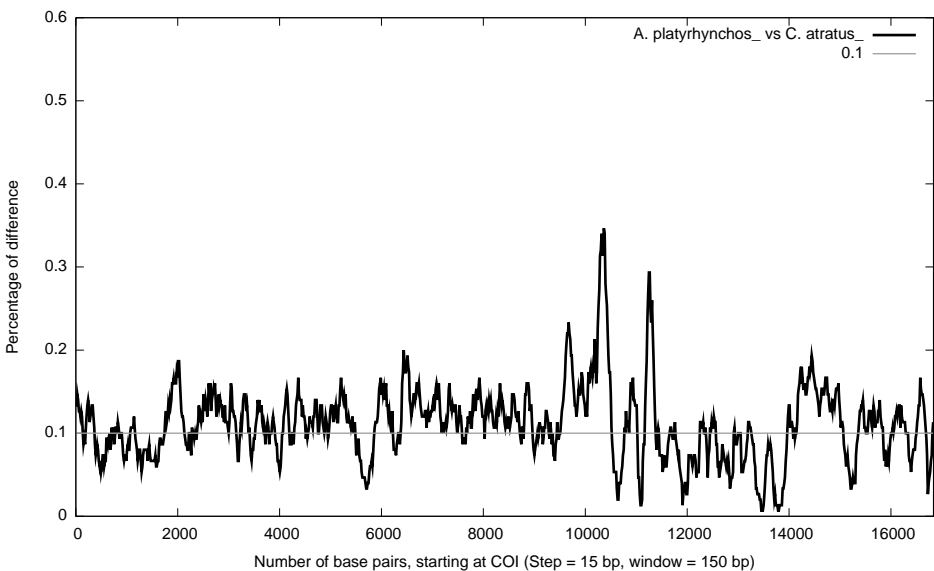

Supplement: File S4 — Sliding window analyses for Sauropsida, Aves, Hemichordata, Coelacanthimorpha, Dipnoi, Chondrichthyes and Cephalochordata. For each family, the folder contains the aligned sequences as well as the sliding window analyses by species pair and for all species pair on a single figure. (ZIP) [file pone.0051263.s004.zip › Sauropsida & Aves/Anatidae/15_150/Anas_platyrhynchos_NC_009684_Cygnus_atratus_NC_012843.pdf]

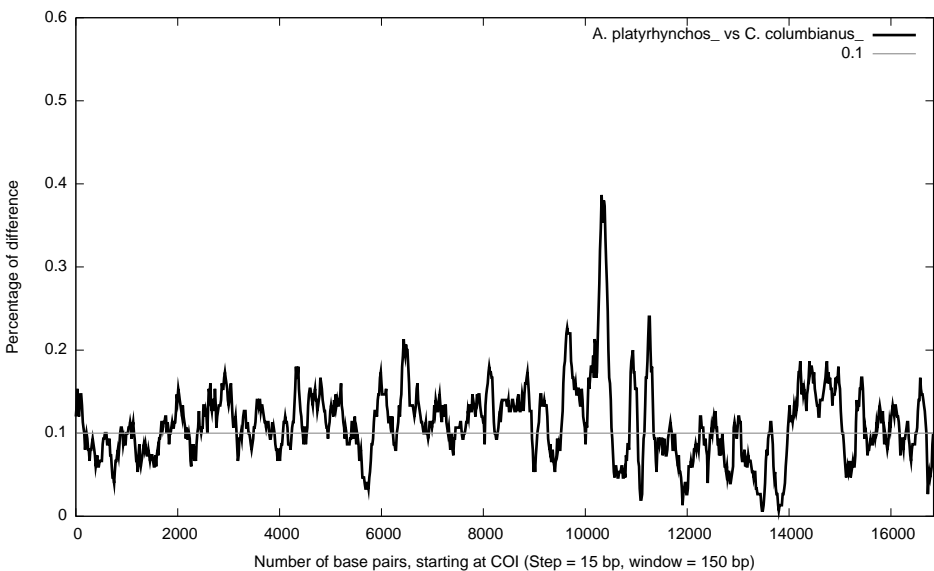

Supplement: File S4 — Sliding window analyses for Sauropsida, Aves, Hemichordata, Coelacanthimorpha, Dipnoi, Chondrichthyes and Cephalochordata. For each family, the folder contains the aligned sequences as well as the sliding window analyses by species pair and for all species pair on a single figure. (ZIP) [file pone.0051263.s004.zip › Sauropsida & Aves/Anatidae/15_150/Anas_platyrhynchos_NC_009684_Cygnus_columbianus_NC_007691.pdf]

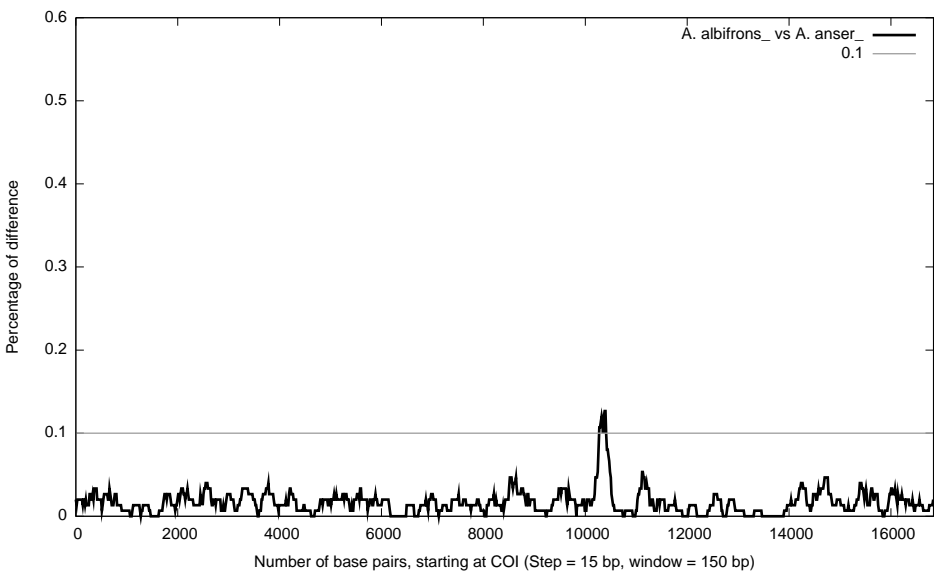

Supplement: File S4 — Sliding window analyses for Sauropsida, Aves, Hemichordata, Coelacanthimorpha, Dipnoi, Chondrichthyes and Cephalochordata. For each family, the folder contains the aligned sequences as well as the sliding window analyses by species pair and for all species pair on a single figure. (ZIP) [file pone.0051263.s004.zip › Sauropsida & Aves/Anatidae/15_150/Anser_albifrons_NC_004539_Anser_anser_NC_011196.pdf]

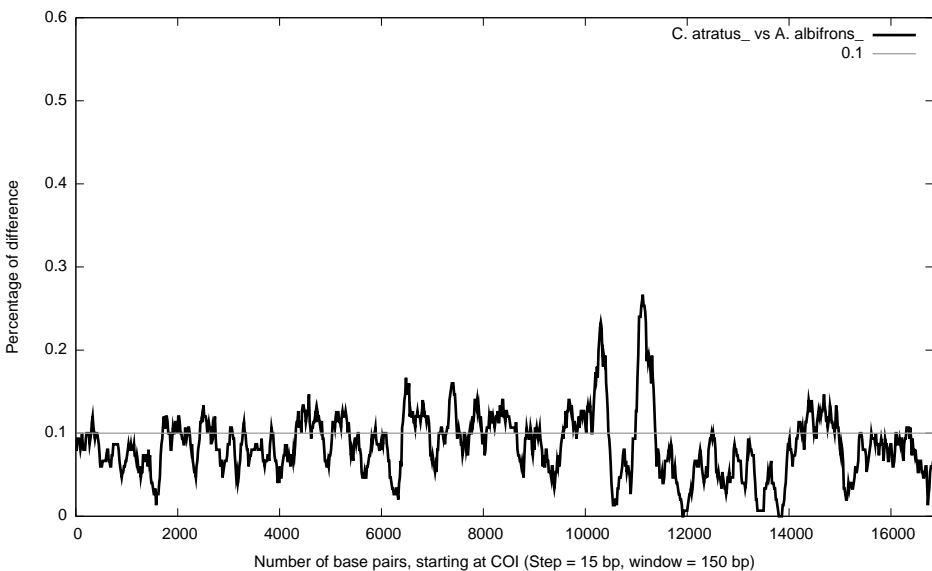

Supplement: File S4 — Sliding window analyses for Sauropsida, Aves, Hemichordata, Coelacanthimorpha, Dipnoi, Chondrichthyes and Cephalochordata. For each family, the folder contains the aligned sequences as well as the sliding window analyses by species pair and for all species pair on a single figure. (ZIP) [file pone.0051263.s004.zip › Sauropsida & Aves/Anatidae/15_150/Cygnus_atratus_NC_012843_Anser_albifrons_NC_004539.pdf]

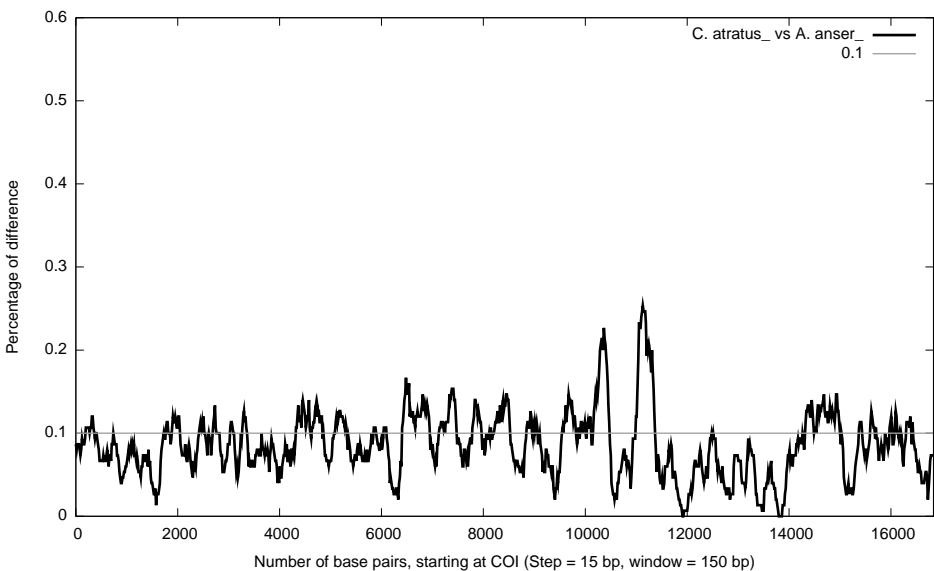

Supplement: File S4 — Sliding window analyses for Sauropsida, Aves, Hemichordata, Coelacanthimorpha, Dipnoi, Chondrichthyes and Cephalochordata. For each family, the folder contains the aligned sequences as well as the sliding window analyses by species pair and for all species pair on a single figure. (ZIP) [file pone.0051263.s004.zip › Sauropsida & Aves/Anatidae/15_150/Cygnus_atratus_NC_012843_Anser_anser_NC_011196.pdf]

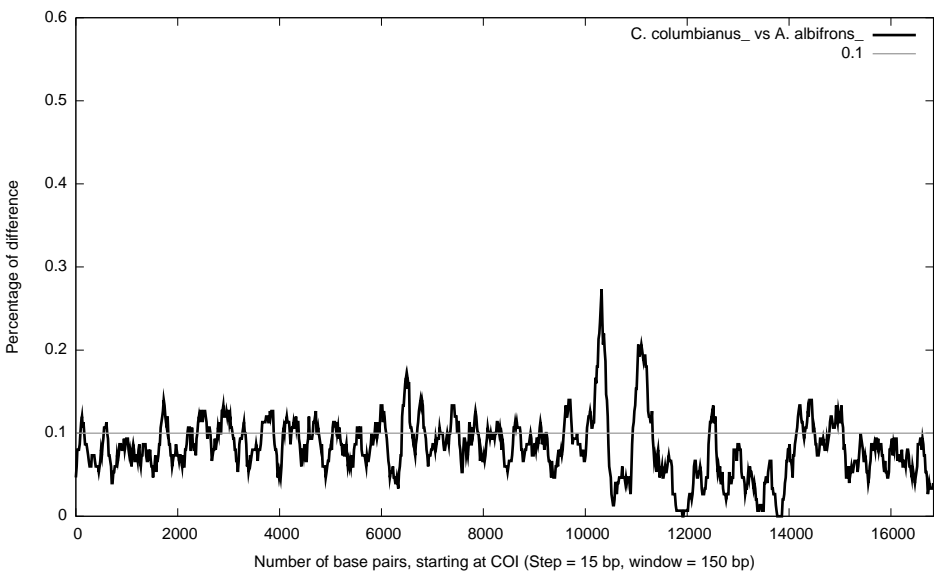

Supplement: File S4 — Sliding window analyses for Sauropsida, Aves, Hemichordata, Coelacanthimorpha, Dipnoi, Chondrichthyes and Cephalochordata. For each family, the folder contains the aligned sequences as well as the sliding window analyses by species pair and for all species pair on a single figure. (ZIP) [file pone.0051263.s004.zip › Sauropsida & Aves/Anatidae/15_150/Cygnus_columbianus_NC_007691_Anser_albifrons_NC_004539.pdf]

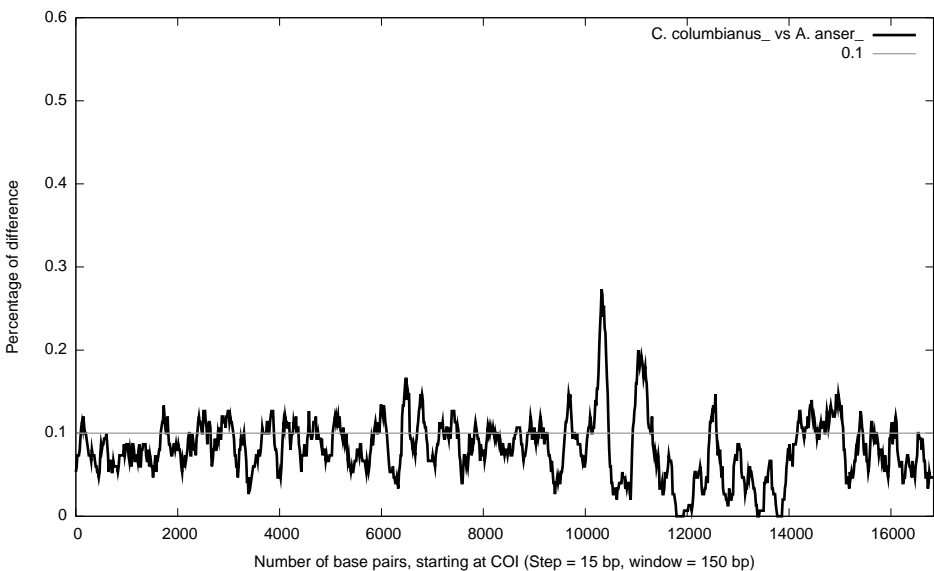

Supplement: File S4 — Sliding window analyses for Sauropsida, Aves, Hemichordata, Coelacanthimorpha, Dipnoi, Chondrichthyes and Cephalochordata. For each family, the folder contains the aligned sequences as well as the sliding window analyses by species pair and for all species pair on a single figure. (ZIP) [file pone.0051263.s004.zip › Sauropsida & Aves/Anatidae/15_150/Cygnus_columbianus_NC_007691_Anser_anser_NC_011196.pdf]

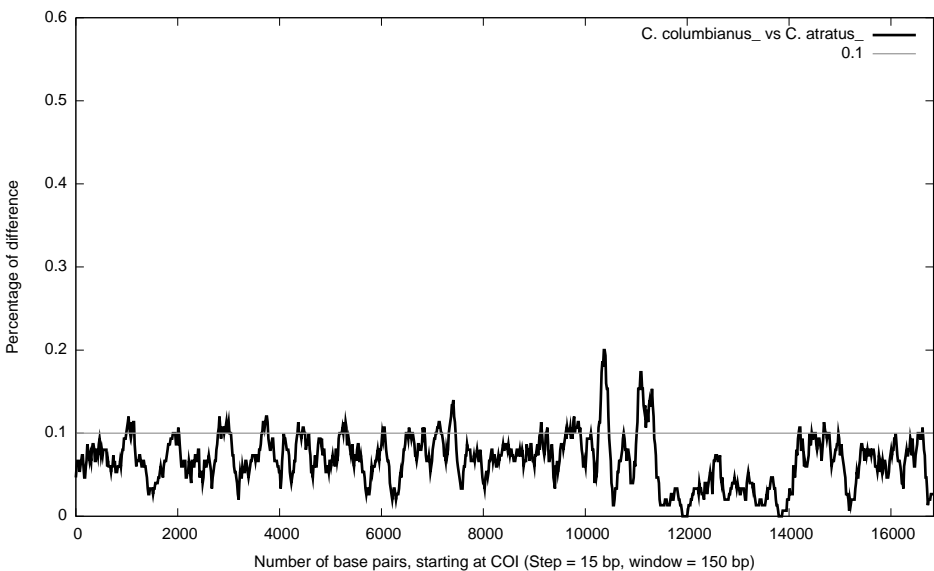

Supplement: File S4 — Sliding window analyses for Sauropsida, Aves, Hemichordata, Coelacanthimorpha, Dipnoi, Chondrichthyes and Cephalochordata. For each family, the folder contains the aligned sequences as well as the sliding window analyses by species pair and for all species pair on a single figure. (ZIP) [file pone.0051263.s004.zip › Sauropsida & Aves/Anatidae/15_150/Cygnus_columbianus_NC_007691_Cygnus_atratus_NC_012843.pdf]

# Anatidae

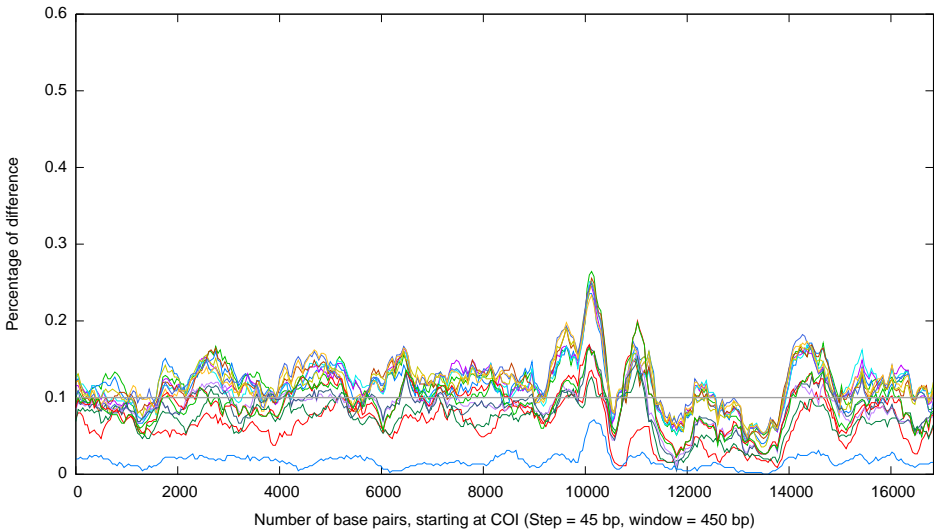

Supplement: File S4 — Sliding window analyses for Sauropsida, Aves, Hemichordata, Coelacanthimorpha, Dipnoi, Chondrichthyes and Cephalochordata. For each family, the folder contains the aligned sequences as well as the sliding window analyses by species pair and for all species pair on a single figure. (ZIP) [file pone.0051263.s004.zip › Sauropsida & Aves/Anatidae/45_450/allCurves.pdf]

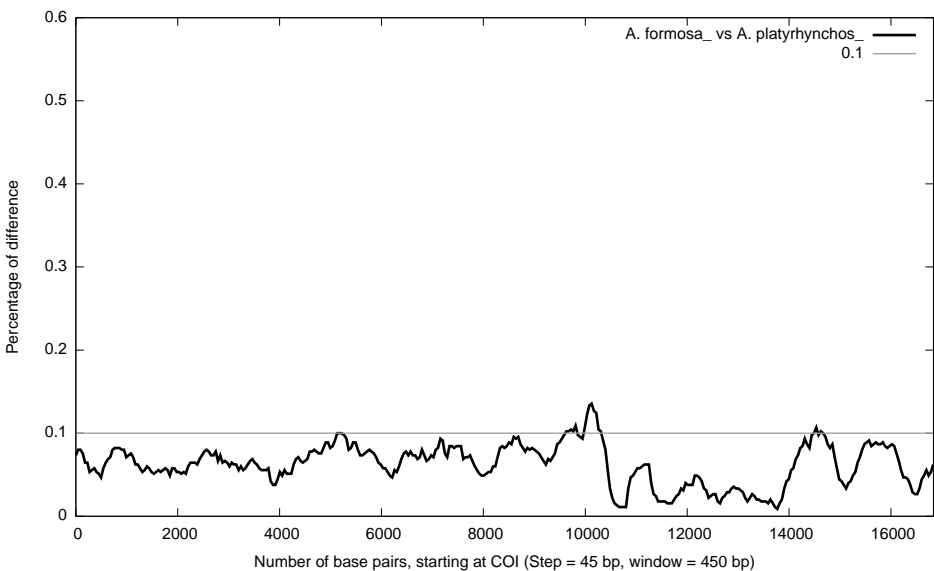

Supplement: File S4 — Sliding window analyses for Sauropsida, Aves, Hemichordata, Coelacanthimorpha, Dipnoi, Chondrichthyes and Cephalochordata. For each family, the folder contains the aligned sequences as well as the sliding window analyses by species pair and for all species pair on a single figure. (ZIP) [file pone.0051263.s004.zip › Sauropsida & Aves/Anatidae/45_450/Anas_formosa_NC_015482_Anas_platyrhynchos_NC_009684.pdf]

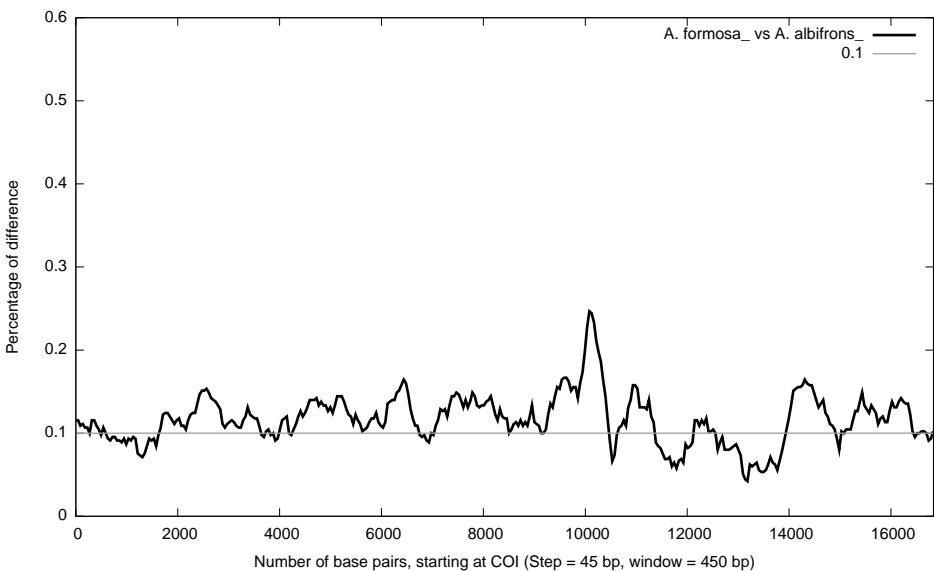

Supplement: File S4 — Sliding window analyses for Sauropsida, Aves, Hemichordata, Coelacanthimorpha, Dipnoi, Chondrichthyes and Cephalochordata. For each family, the folder contains the aligned sequences as well as the sliding window analyses by species pair and for all species pair on a single figure. (ZIP) [file pone.0051263.s004.zip › Sauropsida & Aves/Anatidae/45_450/Anas_formosa_NC_015482_Anser_albifrons_NC_004539.pdf]

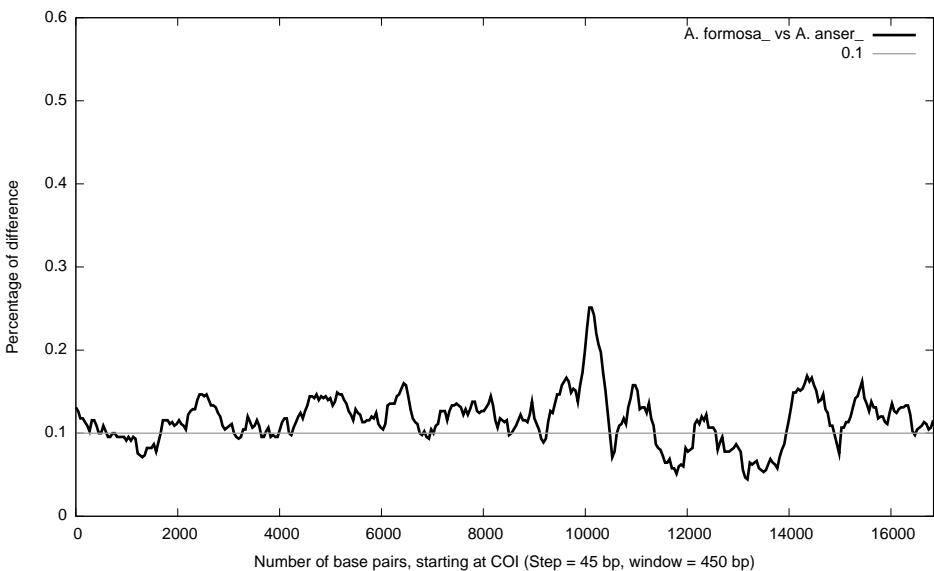

Supplement: File S4 — Sliding window analyses for Sauropsida, Aves, Hemichordata, Coelacanthimorpha, Dipnoi, Chondrichthyes and Cephalochordata. For each family, the folder contains the aligned sequences as well as the sliding window analyses by species pair and for all species pair on a single figure. (ZIP) [file pone.0051263.s004.zip › Sauropsida & Aves/Anatidae/45_450/Anas_formosa_NC_015482_Anser_anser_NC_011196.pdf]

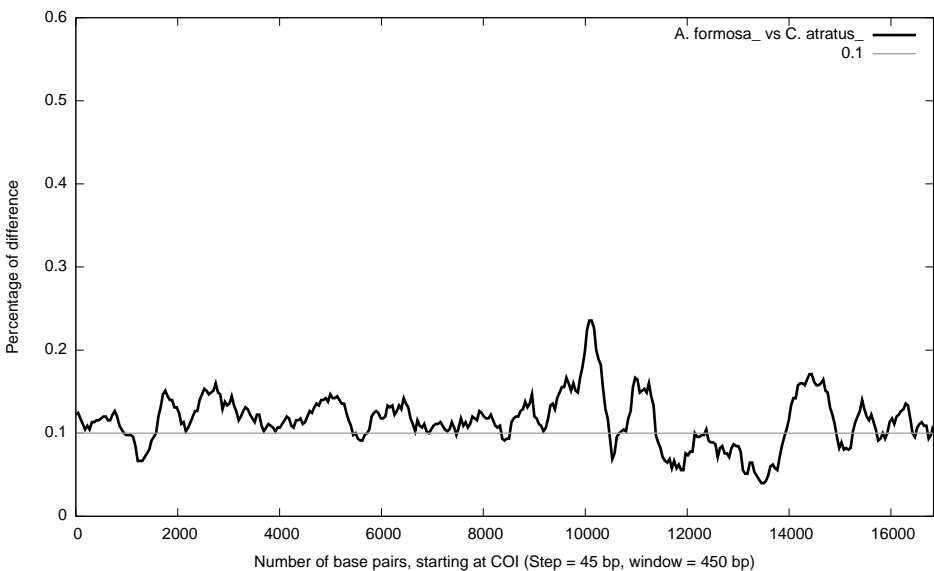

Supplement: File S4 — Sliding window analyses for Sauropsida, Aves, Hemichordata, Coelacanthimorpha, Dipnoi, Chondrichthyes and Cephalochordata. For each family, the folder contains the aligned sequences as well as the sliding window analyses by species pair and for all species pair on a single figure. (ZIP) [file pone.0051263.s004.zip › Sauropsida & Aves/Anatidae/45_450/Anas_formosa_NC_015482_Cygnus_atratus_NC_012843.pdf]

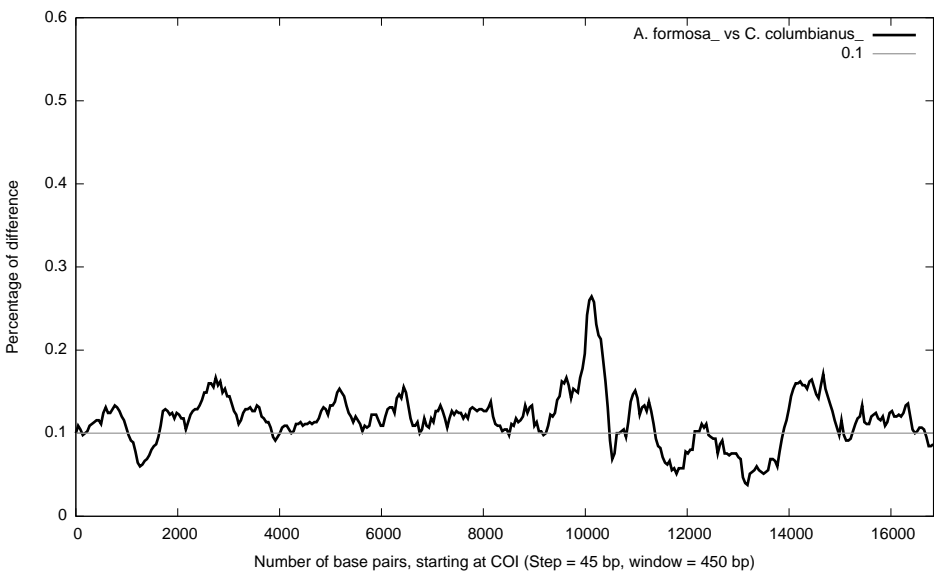

Supplement: File S4 — Sliding window analyses for Sauropsida, Aves, Hemichordata, Coelacanthimorpha, Dipnoi, Chondrichthyes and Cephalochordata. For each family, the folder contains the aligned sequences as well as the sliding window analyses by species pair and for all species pair on a single figure. (ZIP) [file pone.0051263.s004.zip › Sauropsida & Aves/Anatidae/45_450/Anas_formosa_NC_015482_Cygnus_columbianus_NC_007691.pdf]

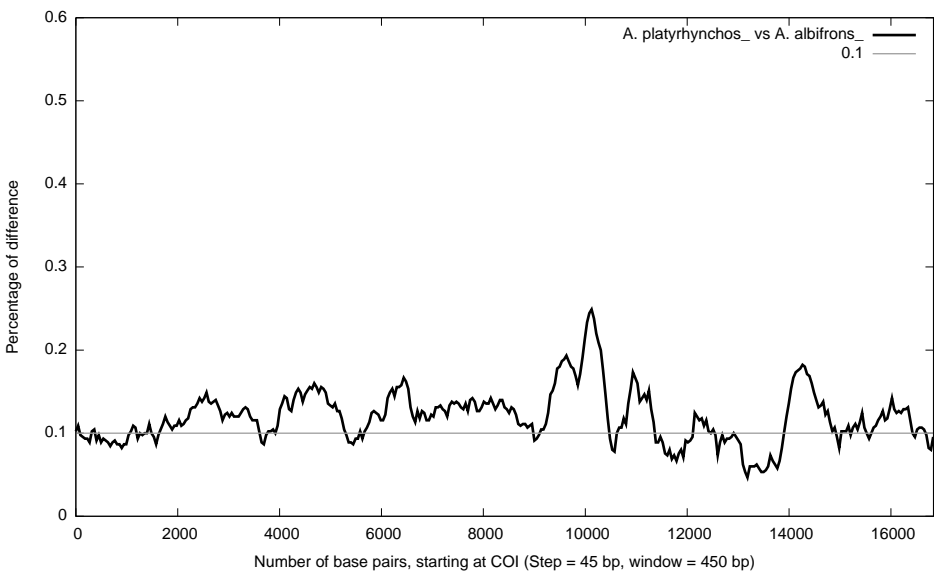

Supplement: File S4 — Sliding window analyses for Sauropsida, Aves, Hemichordata, Coelacanthimorpha, Dipnoi, Chondrichthyes and Cephalochordata. For each family, the folder contains the aligned sequences as well as the sliding window analyses by species pair and for all species pair on a single figure. (ZIP) [file pone.0051263.s004.zip › Sauropsida & Aves/Anatidae/45_450/Anas_platyrhynchos_NC_009684_Anser_albifrons_NC_004539.pdf]

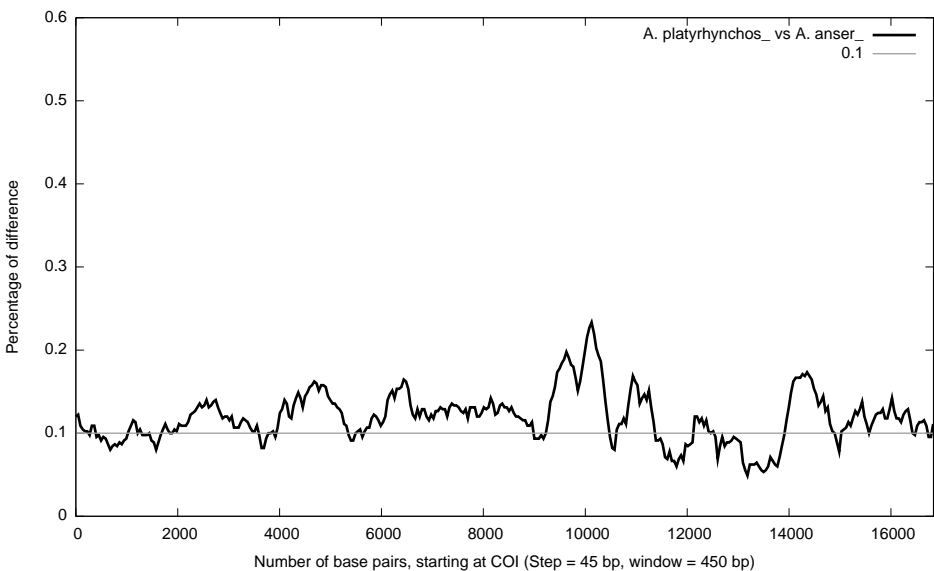

Supplement: File S4 — Sliding window analyses for Sauropsida, Aves, Hemichordata, Coelacanthimorpha, Dipnoi, Chondrichthyes and Cephalochordata. For each family, the folder contains the aligned sequences as well as the sliding window analyses by species pair and for all species pair on a single figure. (ZIP) [file pone.0051263.s004.zip › Sauropsida & Aves/Anatidae/45_450/Anas_platyrhynchos_NC_009684_Anser_anser_NC_011196.pdf]

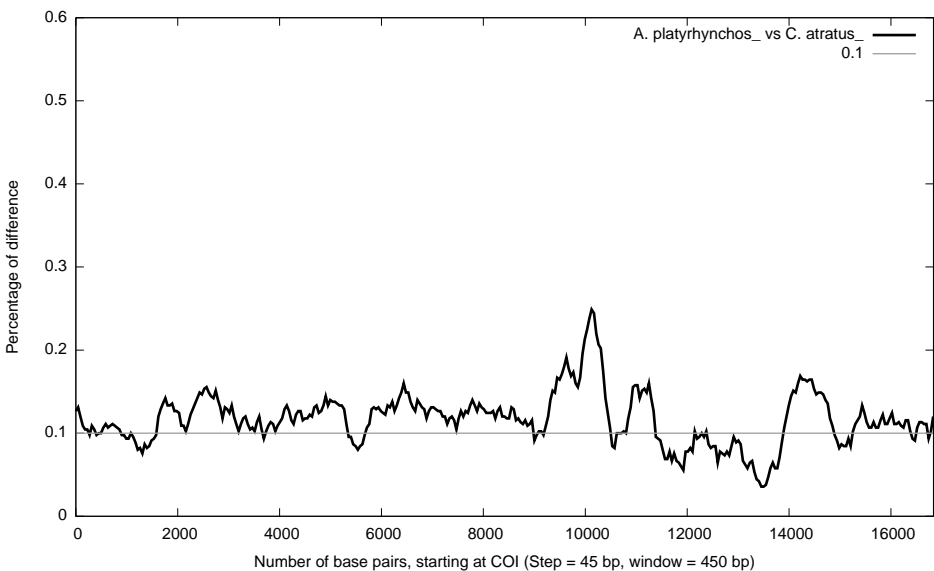

Supplement: File S4 — Sliding window analyses for Sauropsida, Aves, Hemichordata, Coelacanthimorpha, Dipnoi, Chondrichthyes and Cephalochordata. For each family, the folder contains the aligned sequences as well as the sliding window analyses by species pair and for all species pair on a single figure. (ZIP) [file pone.0051263.s004.zip › Sauropsida & Aves/Anatidae/45_450/Anas_platyrhynchos_NC_009684_Cygnus_atratus_NC_012843.pdf]

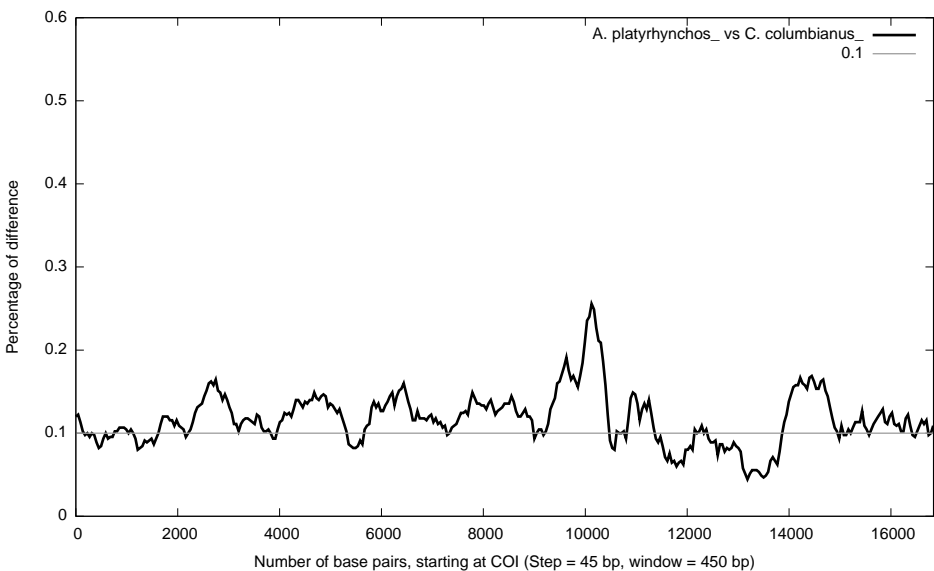

Supplement: File S4 — Sliding window analyses for Sauropsida, Aves, Hemichordata, Coelacanthimorpha, Dipnoi, Chondrichthyes and Cephalochordata. For each family, the folder contains the aligned sequences as well as the sliding window analyses by species pair and for all species pair on a single figure. (ZIP) [file pone.0051263.s004.zip › Sauropsida & Aves/Anatidae/45_450/Anas_platyrhynchos_NC_009684_Cygnus_columbianus_NC_007691.pdf]

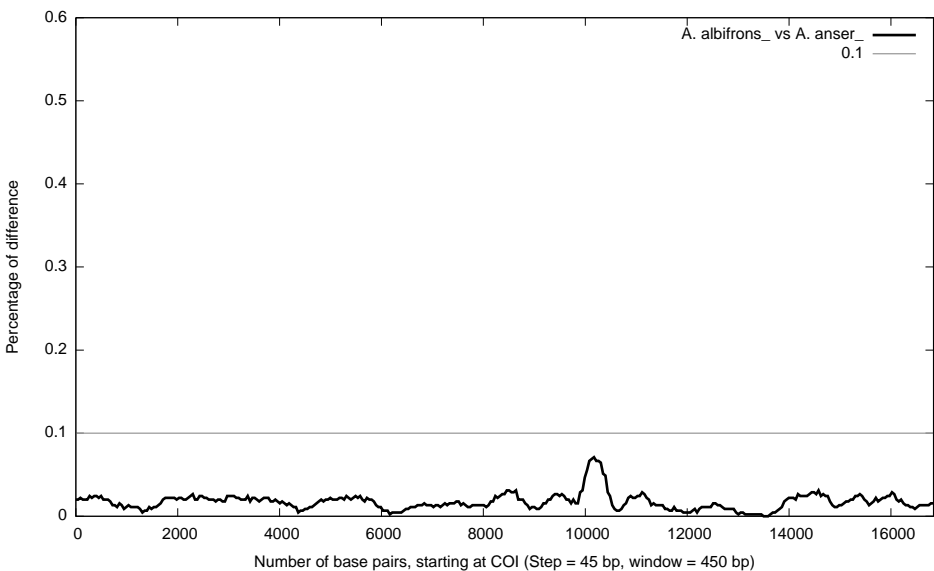

Supplement: File S4 — Sliding window analyses for Sauropsida, Aves, Hemichordata, Coelacanthimorpha, Dipnoi, Chondrichthyes and Cephalochordata. For each family, the folder contains the aligned sequences as well as the sliding window analyses by species pair and for all species pair on a single figure. (ZIP) [file pone.0051263.s004.zip › Sauropsida & Aves/Anatidae/45_450/Anser_albifrons_NC_004539_Anser_anser_NC_011196.pdf]

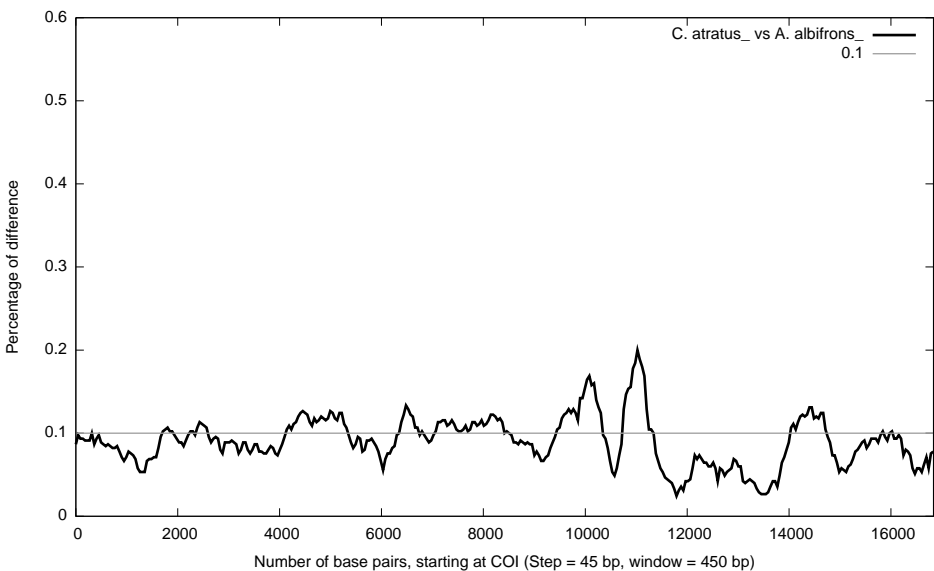

Supplement: File S4 — Sliding window analyses for Sauropsida, Aves, Hemichordata, Coelacanthimorpha, Dipnoi, Chondrichthyes and Cephalochordata. For each family, the folder contains the aligned sequences as well as the sliding window analyses by species pair and for all species pair on a single figure. (ZIP) [file pone.0051263.s004.zip › Sauropsida & Aves/Anatidae/45_450/Cygnus_atratus_NC_012843_Anser_albifrons_NC_004539.pdf]

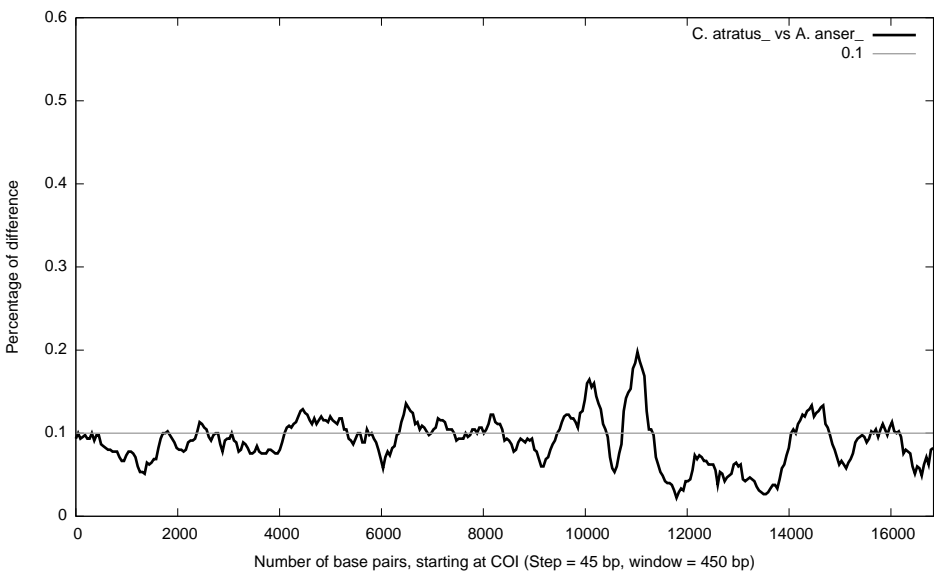

Supplement: File S4 — Sliding window analyses for Sauropsida, Aves, Hemichordata, Coelacanthimorpha, Dipnoi, Chondrichthyes and Cephalochordata. For each family, the folder contains the aligned sequences as well as the sliding window analyses by species pair and for all species pair on a single figure. (ZIP) [file pone.0051263.s004.zip › Sauropsida & Aves/Anatidae/45_450/Cygnus_atratus_NC_012843_Anser_anser_NC_011196.pdf]

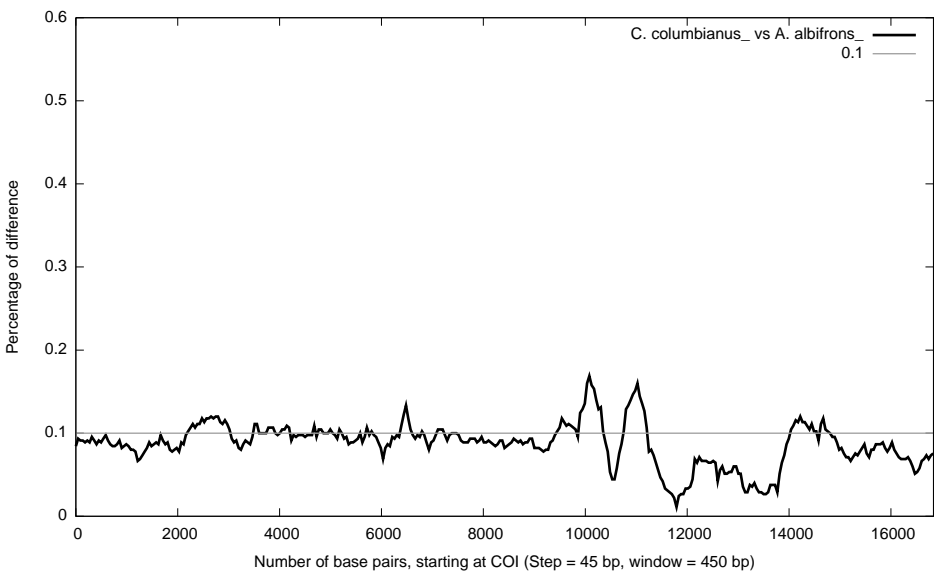

Supplement: File S4 — Sliding window analyses for Sauropsida, Aves, Hemichordata, Coelacanthimorpha, Dipnoi, Chondrichthyes and Cephalochordata. For each family, the folder contains the aligned sequences as well as the sliding window analyses by species pair and for all species pair on a single figure. (ZIP) [file pone.0051263.s004.zip › Sauropsida & Aves/Anatidae/45_450/Cygnus_columbianus_NC_007691_Anser_albifrons_NC_004539.pdf]

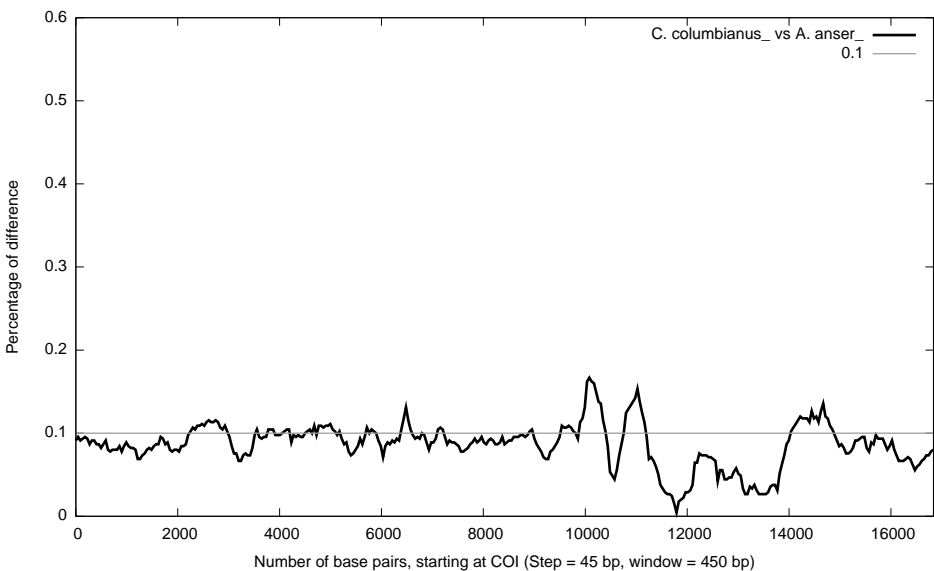

Supplement: File S4 — Sliding window analyses for Sauropsida, Aves, Hemichordata, Coelacanthimorpha, Dipnoi, Chondrichthyes and Cephalochordata. For each family, the folder contains the aligned sequences as well as the sliding window analyses by species pair and for all species pair on a single figure. (ZIP) [file pone.0051263.s004.zip › Sauropsida & Aves/Anatidae/45_450/Cygnus_columbianus_NC_007691_Anser_anser_NC_011196.pdf]

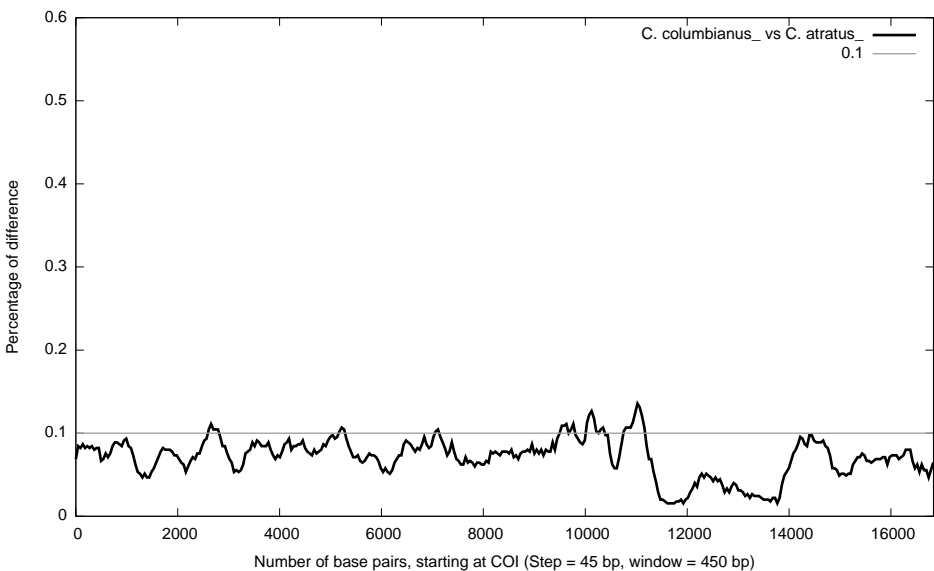

Supplement: File S4 — Sliding window analyses for Sauropsida, Aves, Hemichordata, Coelacanthimorpha, Dipnoi, Chondrichthyes and Cephalochordata. For each family, the folder contains the aligned sequences as well as the sliding window analyses by species pair and for all species pair on a single figure. (ZIP) [file pone.0051263.s004.zip › Sauropsida & Aves/Anatidae/45_450/Cygnus_columbianus_NC_007691_Cygnus_atratus_NC_012843.pdf]
